# Supplementary material for: Safety and immunogenicity of a phase 1/2 randomized clinical trial of a quadrivalent, mRNA-based seasonal influenza vaccine (mRNA-1010) in healthy adults: interim analysis
Source: Nat Commun. 2023 Jun 19;14:3631. doi: 10.1038/s41467-023-39376-7 (PMC10279702; doi:10.1038/s41467-023-39376-7)
Supplement: Supplementary file 1 — Supplementary Information [file 41467_2023_39376_MOESM1_ESM.pdf]

Supplement

METHODS

Inclusion and Exclusion Criteria for Part 1

Each participant was required to have met the following criteria for enrollment into Part 1 of the study:

- An adult, ≥18 years of age at the time of consent, who was in good health, in the opinion of the Investigator, based on review of medical history and physical examination performed at screening
- Understood and was willing and physically able to comply with protocol-mandated follow-up, including all procedures, in the opinion of the Investigator
- Provided written informed consent for participation in this study, including all evaluations and procedures as specified in the protocol
- Had a body mass index of 18 kg/m^2 to 35 kg/m^2 (inclusive) at screening
- If female and not of childbearing potential, could be enrolled in the study. A follicle-stimulating hormone (FSH) level may be measured at the discretion of the Investigator to confirm postmenopausal status.
- If female and of childbearing potential, met all the following criteria to be enrolled in the study:
    - Had a negative pregnancy test at screening and on the day of vaccination (Day 1)
    - Had practiced adequate contraception or abstained from all activities that could result in pregnancy for at least 28 days prior to Day 1. Adequate female contraception was defined as consistent and correct use of a US Food and Drug Administration (FDA)-approved contraceptive method in accordance with the product label
    - Had agreed to continue adequate contraception through 3 months following vaccine administration

    - Was not currently breastfeeding

Participants who met any of the following criteria were excluded from Part 1 of the study:

- Had significant exposure to someone with laboratory-confirmed severe acute respiratory syndrome coronavirus 2 (SARS-CoV-2) infection, coronavirus disease 2019 (COVID-19), or influenza-like illness (ILI) in the past 14 days prior to screening, as defined by the Centers for Disease Control and Prevention (CDC) as close contact with someone who has COVID-19
- Had a positive SARS-CoV-2 reverse transcription-polymerase chain reaction (RT-PCR) or antigen test in the 10 days prior to screening
    - A participant who had a positive SARS-CoV-2 serological test in the 10 days prior to screening but was otherwise asymptomatic and did not have a positive SARS-CoV-2 RT-PCR or antigen test at screening could still be enrolled if they met the study eligibility criteria
    - A participant who had a positive SARS-CoV-2 RT-PCR or antigen test in the 10 days prior to screening could be enrolled 10 days after symptom onset or 10 days after the date of the first positive SARS-CoV-2 RT-PCR test without repeat testing, provided the participant was asymptomatic and met the study eligibility criteria, as well as the CDC recommendation for duration of isolation and precautions
    - Additionally, a participant who met all study eligibility criteria (note fourth exclusion criterion bullet) at screening but later tested positive for SARS-CoV-2 at Visit 1 would continue participation in the study
- Had clinical screening laboratory values (white blood cell count, hemoglobin, platelets, alanine aminotransferase, aspartate aminotransferase, creatinine, alkaline phosphatase, and total bilirubin) > Grade 1

- Was acutely ill or febrile (body temperature ≥38.0°C/100.4°F) 72 hours prior to or at screening or Day 1. Participants meeting this criterion could be rescheduled within the 28-day screening window and would retain their initially assigned participant number
- Had a pre-existing medical condition that was not stable, at the discretion of the Investigator. A stable medical condition was defined as disease not requiring significant change in therapy or hospitalization for worsening disease during the 2 months before enrollment. Participants could be rescreened if they were not medically stable at screening. Medical conditions included (but were not limited to) the following:
    - Uncontrolled hypertension or systolic blood pressure >150 mm Hg or diastolic blood pressure >90 mm Hg at screening
    - Congestive heart failure
    - Unstable angina or exacerbation of coronary artery disease within 6 months before the day of vaccination (Day 1) that required cardiac intervention or new cardiac medications to control symptoms
    - Diabetes requiring the use of medicine (injectable or oral) or not controlled with diet
    - Chronic obstructive pulmonary disease, asthma requiring daily use of a bronchodilator or inhaled/systemic corticosteroids, or other chronic lung diseases such as pulmonary fibrosis
- Had a medical, psychiatric, or occupational condition that could pose additional risk as a result of participation or that could interfere with safety assessments or interpretation of results according to the Investigator's judgment
- Had a current or previous diagnosis of immunocompromising condition, immune-mediated disease, or other immunosuppressive condition

- Had received systemic immunosuppressants or immune-modifying drugs for >14 days in total within 6 months prior to screening (for corticosteroids ≥10 mg/day of prednisone or equivalent) or was anticipating the need for immunosuppressive treatment at any time during participation in the study
- Had a history of anaphylaxis, urticaria, or other significant adverse reaction requiring medical intervention after receipt of a vaccine or any of the components contained in the investigational vaccine
- Had a history of coagulopathy or bleeding disorder considered a contraindication to intramuscular injection or phlebotomy
- Had received or planned to receive any licensed vaccine ≤28 days prior to the vaccination (Day 1) or planned to receive a licensed vaccine within 28 days after vaccination, with the exception of vaccines authorized or approved for the prevention of COVID-19 (regardless of type of vaccine) that become available to participants during the study. Efforts were made to space study vaccination and COVID-19 vaccination by at least 7 and preferably 14 days, but COVID-19 vaccination should not have been delayed
- Had received a seasonal influenza vaccine or any other investigational influenza vaccine after January 1, 2021
- Had received systemic immunoglobulins or blood products within 3 months prior to screening or had plans to receive them during the study
- Had a diagnosis of malignancy within the previous 10 years (excluding nonmelanoma skin cancer)
- Had donated ≥450 mL of blood products within 28 days prior to screening or planned to donate blood products during the study

- Had participated in an interventional clinical study within 28 days prior to screening based on the medical history interview or planned to do so while participating in this study
- Was an immediate family member or household member of study personnel, study site staff, or Sponsor personnel

Inclusion and Exclusion Criteria for Part 2

Each participant was required to have met the following criteria for enrollment into Part 2 of the study:

- An adult, ≥18 years of age at the time of consent, who in the opinion of the Investigator, was medically stable based on review of medical history and physical examination performed at screening. Medically stable was defined as disease not requiring significant change in therapy or hospitalization for worsening disease during the 3 months before enrollment. Participants could be rescreened if they were not medically stable at screening
- Understood and was willing and physically able to comply with protocol-mandated follow-up, including all procedures, in the opinion of the Investigator
- Provided written informed consent for participation in this study, including all evaluations and procedures as specified in the protocol
- If female and not of childbearing potential, could be enrolled in the study. An FSH level may be measured at the discretion of the Investigator to confirm postmenopausal status
- If female and of childbearing potential, met all the following criteria to be enrolled in the study:
    - Had a negative pregnancy test at screening and on the day of vaccination (Day 1)
    - Had practiced adequate contraception or abstained from all activities that could result in pregnancy for at least 28 days prior to Day 1

- Had agreed to continue adequate contraception through 3 months following vaccine administration. Adequate female contraception was defined as consistent and correct use of an FDA-approved contraceptive method in accordance with the product label
- Was not currently breastfeeding

Participants who met any of the following criteria were excluded from Part 2 of the study:

- Had close contact to someone with SARS-CoV-2 infection or COVID-19 as defined by the CDC in the past 14 days prior to screening, unless the participant had been fully vaccinated for COVID-19
- Was acutely ill or febrile (body temperature ≥38.0°C/100.4°F) 72 hours prior to or at screening or Day 1. Participants meeting this criterion could be rescheduled within the 28-day screening window and would retain their initially assigned participant number
- Had a medical, psychiatric, or occupational condition, or history of substance abuse that may pose additional risk as a result of participation or that could interfere with safety assessments or interpretation of results according to the Investigator's judgment
- Had a current or previous diagnosis of immunocompromising/immunosuppressive condition, immune-mediated disease requiring immune-modifying therapy, asplenia, or recurrent severe infections (HIV-positive participants on antiretroviral therapy with cluster of differentiation 4 count ≥350 cells/mm^3 and HIV-RNA ≤500 copies/mL within the past 12 months were permitted)
- Had received systemic immunosuppressants or immune-modifying drugs for >14 days in total within 6 months prior to screening (for corticosteroids ≥10 mg/day of prednisone or equivalent) or was anticipating the need for systemic immunosuppressive treatment at any time during participation in the study

- Had a history of anaphylaxis, urticaria, or other significant adverse reaction requiring medical intervention after receipt of a vaccine or any of the components contained in the investigational vaccine or the comparator vaccine, which is an egg-based influenza vaccine
- Had a coagulopathy or bleeding disorder considered a contraindication to intramuscular injection or phlebotomy
- Had received or planned to receive any licensed vaccine ≤28 days prior to vaccination (Day 1) or planned to receive a licensed vaccine within 28 days after vaccination, with the exception of vaccines authorized or approved for the prevention of COVID-19 (regardless of type of vaccine) that become available to participants during the study. Efforts were made to space study vaccination and COVID-19 vaccination by at least 7 and preferably 14 days, but COVID-19 vaccination should not have been delayed
- Had received a seasonal influenza vaccine or any other investigational influenza vaccine within 6 months prior to screening
- Had tested positive for influenza by CDC-recommended testing methods within 6 months prior to screening
- Had received systemic immunoglobulins or blood products within 3 months prior to screening or had plans to receive them during the study
- Had donated ≥450 mL of blood products within 28 days prior to screening or planned to donate blood products during the study
- Had participated in an interventional clinical study within 28 days prior to screening based on the medical history interview or planned to do so while participating in this study
- Was an immediate family member or household member of study personnel, study site staff, or Sponsor personnel

Blinding

This was an observer-blind study, with the Investigator, study staff, study participants, site monitors, and Sponsor personnel blinded to the vaccine administered until the study database was locked and unblinded. A limited number of unblinded personnel were assigned to vaccine accountability procedures and prepared the study vaccines, while an unblinded medically qualified study site personnel administered vaccination. Unblinded site monitors were assigned responsibilities to ensure sites were following all proper vaccination accountability, preparation, and administration procedures. The planned interim analysis was performed by an independent, unblinded statistical and programming team and did not communicate results to blinded Investigators, study site staff, clinical monitors, or participants. The Data Safety Monitoring Board reviewed unblinded interim analysis data provided by the independent unblinded statistician to safeguard the interests of the study participants.

Vaccine Strains

In Part 1, mRNA-1010–encoded influenza strains included A/Wisconsin/588/2019(H1N1)pdm09, A/Hong Kong/45/2019(H3N2), B/Washington/02/2019 (B/Victoria lineage), and B/Phuket/3073/2013 (B/Yamagata lineage). In Part 2, mRNA-1010–encoded influenza strains included A/Wisconsin/588/2019(H1N1)pdm09, A/Cambodia/e0826360/2020(H3N2), B/Washington/02/2019 (B/Victoria lineage), and B/Phuket/3073/2013 (B/Yamagata lineage). Afluria influenza strains included A/Victoria/2570/2019 IVR-215 (an A/Victoria/2570/2019 [H1N1]pdm09–like virus), A/Cambodia/e0826360/2020 IVR-224 (an A/Cambodia/e0826360/2020 [H3N2]–like virus), B/Victoria/705/2018 BVR-11 (a B/Washington/02/2019–like virus), and B/Phuket/3073/2013 BVR-1B (a B/Phuket/3073/2013–like virus).

Hemagglutination Inhibition (HAI) Assay

Sera were tested by HAI assay using standard methods[1]. In brief, serum samples were treated with receptor destroying enzyme (RDE) to remove non-specific inhibitors of hemagglutination. Eleven 2-fold serial dilutions of treated serum samples were prepared in duplicate 96-well plates. Respective influenza virus, at a target working dilution of 4 HA units per well (per World Health Organization recommendations), was added to the serum dilutions and incubated for 60 minutes at room temperature. Guinea pig red blood cell (RBC) suspension (0.75%) was added to the serum-virus mixture and incubated for 60 minutes at room temperature. Following RBC incubation, plates were read and the results recorded; geometric mean titer (GMT) was calculated from duplicate plate readings. The HAI titer was defined as the last dilution at which agglutination of RBCs was inhibited.

Statistical Analyses

The sample sizes for Part 1 and Part 2 were not based on formal statistical hypothesis testing. In Part 1, it was expected that with 45 participants in each vaccine group, there was an approximately 90% probability to observe at least 1 participant with an adverse event (AE; if the true incidence rate of AEs was 5%) or an approximately 99% probability if the true incidence rate was 10%. For Part 2, a sample size of 150 participants in each vaccine group had at least a 95% probability to observe at least 1 participant with an AE if the true incidence rate was 2%; if the true incidence rate was 3%, then the probability would be 99%.

In Part 2 of the study, an analysis of covariance model was used with hemagglutination inhibition (HAI) titers at Day 29 as a dependent variable and a group variable as the fixed variable. The GMT of mRNA-1010 at Day 29 was estimated by the geometric least square mean (GLSM) from the model. The geometric mean ratio (GMTr; ratio of GMT) was estimated by the ratio of GLSM from the

model. The corresponding 2-sided 95% confidence interval (CI) was calculated to assess the difference in immune response between mRNA-1010 compared with the active comparator at Day 29.

REFERENCES

1 Nachbagauer, R. *et al.* A universal influenza virus vaccine candidate confers protection against pandemic H1N1 infection in preclinical ferret studies. *NPJ Vaccines* **2**, 26, doi:10.1038/s41541-017-0026-4 (2017).

Supplement Figure 1. Study designs of Part 1 and Part 2.

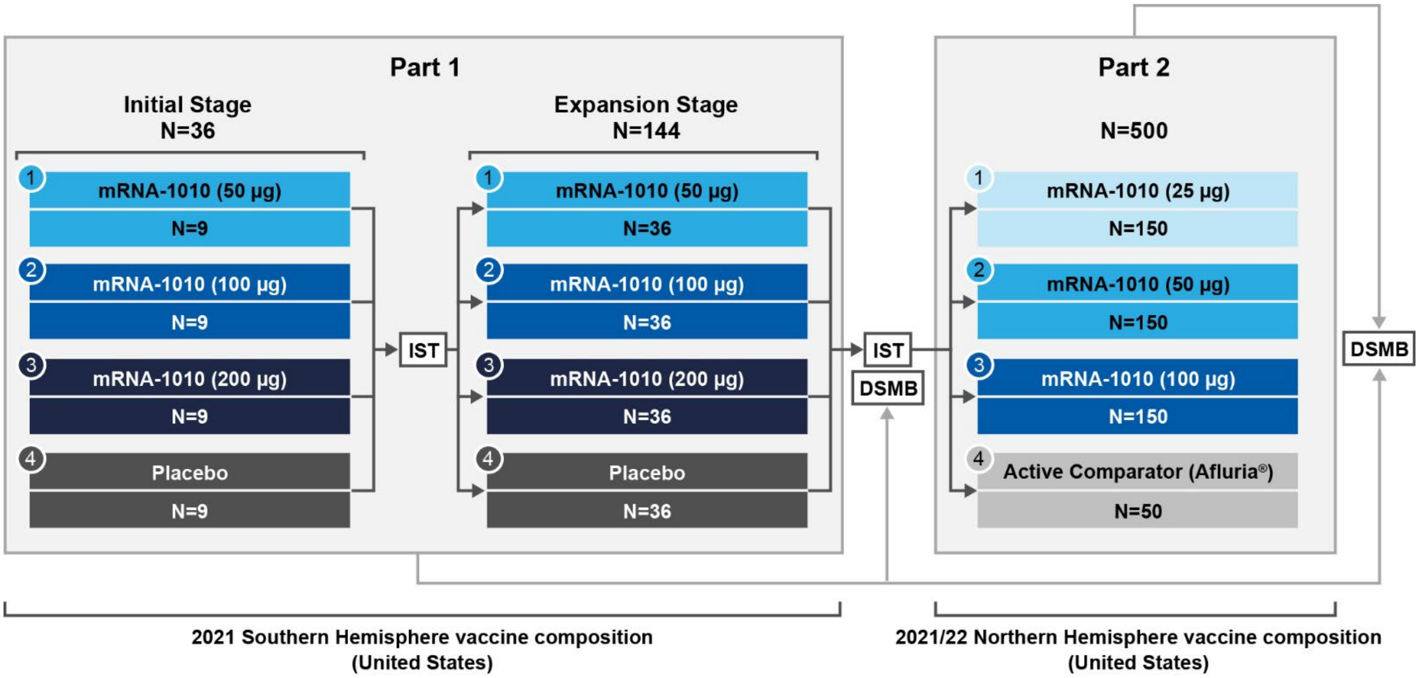

DSMB, Data and Safety Monitoring Board; IST, internal safety team.

Supplement Figure 2. Summary of solicited local and systemic adverse reactions within 7 days after vaccination by participant age group in Part 1. Percentages of (a) local or (b) systemic adverse reactions are based on participants in the solicited safety population. Number of participants in the placebo group were 20 (18-49 years) and 24 (≥50 years); number of participants in the mRNA-1010 groups were 23 (50 µg), 21 (100 µg), and 23 (200 µg) for 18-49 years, and 22 (50 µg), 25 (100 µg), and 21 (200 µg) for ≥50 years. mRNA, messenger RNA.

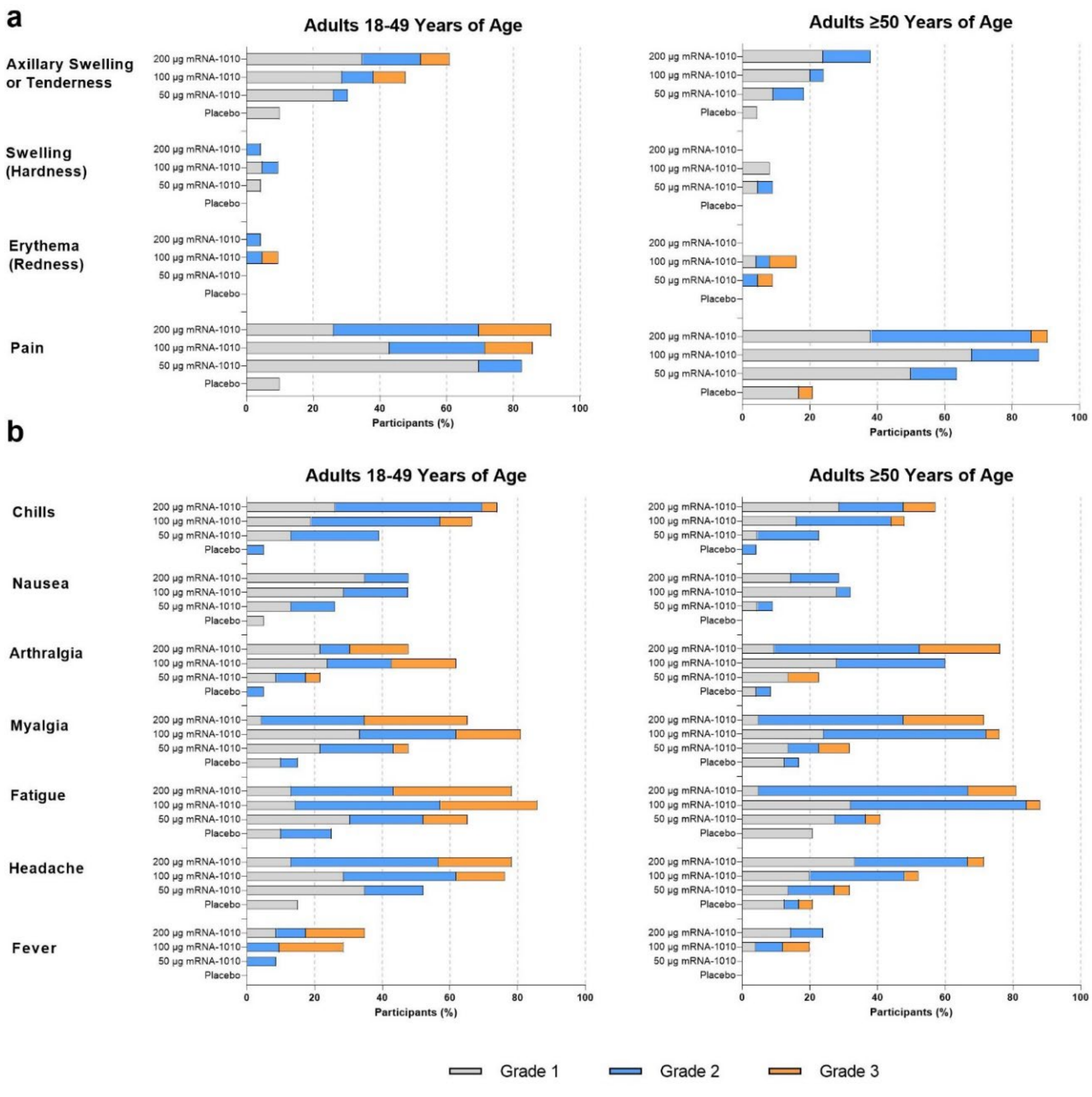

Supplement Figure 3. Summary of solicited local and systemic adverse reactions within 7 days after vaccination by participant age group in Part 2. Percentages of (a) local or (b) systemic adverse reactions are based on participants in the solicited safety population. Number of participants in the Alfuria group were 21 (18-49 years), 19 (50-64 years), and 13 (⩾65 years); number of participants in the mRNA-1010 groups were 60 (25 µg), 57 (50 µg), and 58 (100 µg) for 18-49 years; 58 (25 µg), 58 (50 µg), and 57 (100 µg), for 50-64 years; and 31 (25 µg), 31 (50 µg), and 30 (100 µg) for ≥65 years). mRNA, messenger RNA.

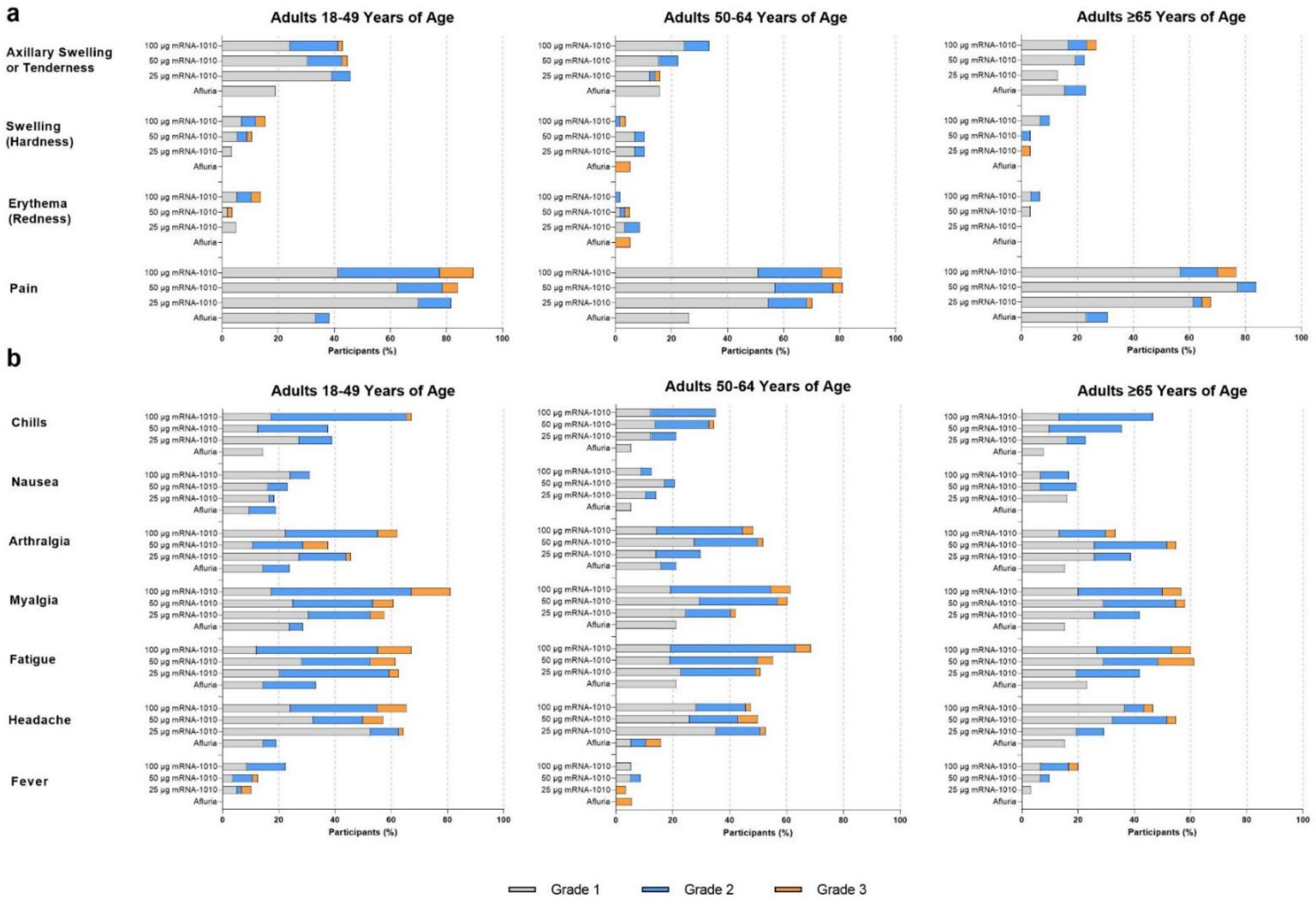

Supplement Figure 4. GMTs and GMFRs of anti-hemagglutinin antibodies for vaccine-matched seasonal influenza strains in adults ⩾18 years of age in Part 2. Hemagglutination inhibition GMTs with associated 95% CIs against seasonal influenza A strains (A/Wisconsin/588/2019[H1N1]pdm09 and A/Cambodia/e0826360/2020[H3N2]) or influenza B strains (B/Washington/02/2019 [B/Victoria lineage] and B/Phuket/3073/2013 [B/Yamagata lineage]) are shown at Day 1 (baseline) and Day 29 (28 days after vaccination) among participants in the per-protocol population for Part 2. Dots correspond to participant-level titers; number of participants were 52 (Afluria), 143 (mRNA-1010 25 µg), 138 (mRNA-1010 50 µg), and 142 (mRNA-1010 100 µg). GMFRs at Day 29 from Day 1 are shown above each Day 29 bar plot. LLOQs were 10 for each influenza strain; ULOQs were 6400 (H1N1 and B/Yamagata), 1280 (H3N2), or 3200 (B/Victoria). HAI GMTs <LLOQ were replaced by 0.5 x LLOQ and values >ULOQ were converted to ULOQ CI, confidence interval; HAI, hemagglutination inhibition; GMFR, geometric mean fold-rise; GMT, geometric mean titer; LLOQ, lower limit of quantification; mRNA, messenger RNA; ULOQ, upper limit of quantification.

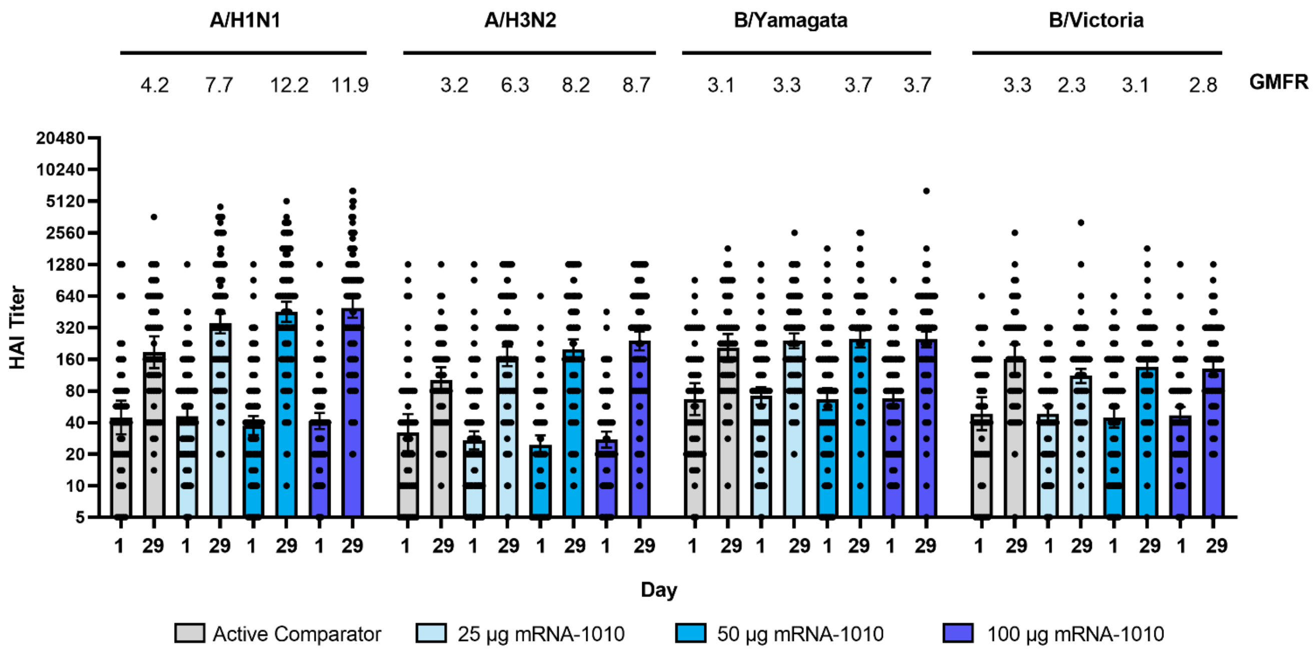

Supplement Figure 5. Ratios of GMTs of anti-hemagglutinin antibodies after vaccination with mRNA-1010 compared with Afluria in adults ≥18 years of age in Part 2. Ratios of HAI GMTs against vaccine-matched seasonal influenza strains (A/Wisconsin/588/2019[H1N1]pdm09, A/Cambodia/e0826360/2020[H3N2], B/Washington/02/2019 [B/Victoria lineage], and B/Phuket/3073/2013 [B/Yamagata lineage]) at 28 days after vaccination with mRNA-1010 compared with Afluria are shown for participants in the per-protocol population for Part 2. Number of participants were 52 (Afluria), 143 (mRNA-1010 25 µg), 138 (mRNA-1010 50 µg), and 142 (mRNA-1010 100 µg). Horizontal dotted line indicates a GMT ratio of 1, which reflects comparable GMTs between mRNA-1010 and Afluria. Error bars represent 95% CI. CI, confidence interval; HAI, hemagglutination inhibition; GMT, geometric mean titer; LLOQ, lower limit of quantification; mRNA, messenger RNA; ULOQ, upper limit of quantification.

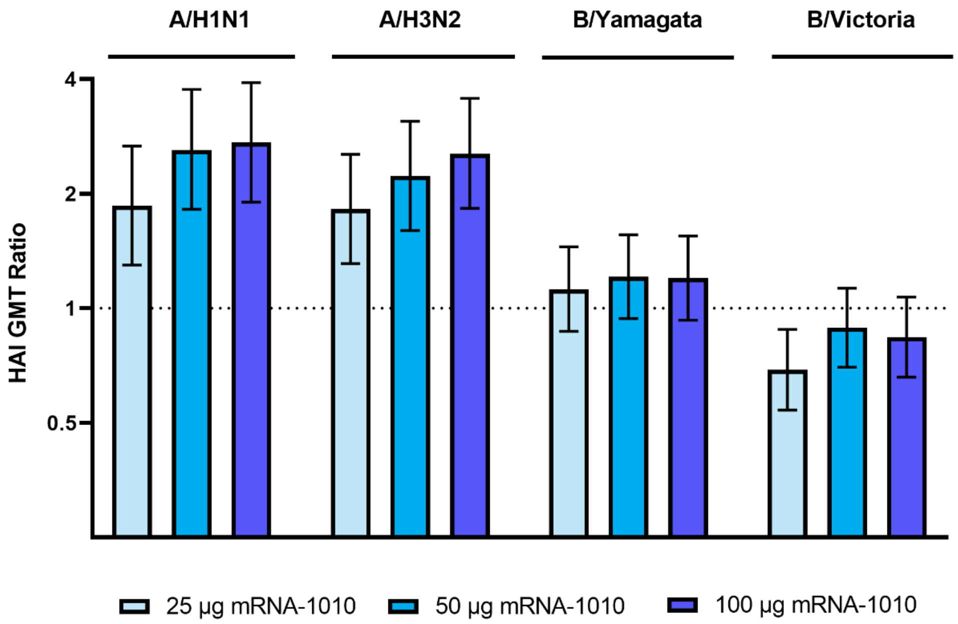

Supplement Table 1. Participant baseline demographics by study part (safety population)

	Part 1								Part 2											
	18-49 Years of Age				≥50 Years of Age				18-49 Years of Age				50-64 Years of Age				≥65 Years of Age			
	**Placebo (n=21)**	**50 µg mRNA-1010 (n=23)**	**100 µg mRNA-1010 (n=21)**	**200 µg mRNA-1010 (n=23)**	**Placebo (n=24)**	**50 µg mRNA-1010 (n=22)**	**100 µg mRNA-1010 (n=25)**	**200 µg mRNA-1010 (n=21)**	**Afluria (n=21)**	**25 µg mRNA-1010 (n=60)**	**50 µg mRNA-1010 (n=58)**	**100 µg mRNA-1010 (n=59)**	**Afluria (n=20)**	**25 µg mRNA-1010 (n=59)**	**50 µg mRNA-1010 (n=58)**	**100 µg mRNA-1010 (n=57)**	**Afluria (n=13)**	**25 µg mRNA-1010 (n=31)**	**50 µg mRNA-1010 (n=31)**	**100 µg mRNA-1010 (n=31)**
**Age, y**																				
Mean ± SD	38.1 ±8.3	35.2 ±9.6	37.0 ±10.0	37.3 ±8.3	64.2 ±10.9	61.8 ±7.3	65.0 ±8.7	61.5 ±9.4	35.9 ±8.7	34.3 ±9.5	34.5 ±8.9	32.4 ±9.0	56.0 ±4.0	56.2 ±3.8	55.1 ±3.8	56.3 ±4.3	73.5 ±5.4	70.5 ±4.5	71.1 ±4.4	71.2 ±5.6
Median (range)	40.0 (18-49)	38.0 (18-49)	40.0 (19-49)	39.0 (19-48)	61.0 (50-90)	62.0 (51-74)	64.0 (51-83)	59.0 (51-89)	37.0 (20-49)	34.0 (18-49)	36.0 (19-49)	32.0 (18-47)	55.0 (50-64)	56.0 (50-64)	55.0 (50-64)	55.0 (50-64)	72.0 (66-86)	70.0 (65-86)	71.0 (65-81)	70.0 (65-82)
**Sex, n (%)**																				
Male	13 (61.9)	13 (56.5)	11 (52.4)	6 (26.1)	9 (37.5)	13 (59.1)	10 (40.0)	5 (23.8)	9 (42.9)	27 (45.0)	25 (43.1)	26 (44.1)	11 (55.0)	25 (42.4)	29 (50.0)	20 (35.1)	4 (30.8)	10 (32.3)	13 (41.9)	15 (48.4)
Female	8 (38.1)	10 (43.5)	10 (47.6)	17 (73.9)	15 (62.5)	9 (40.9)	15 (60.0)	16 (76.2)	12 (57.1)	33 (55.0)	33 (56.9)	33 (55.9)	9 (45.0)	34 (57.6)	29 (50.0)	37 (64.9)	9 (69.2)	21 (67.7)	18 (58.1)	16 (51.6)
**Race, n (%)**																				
White	16 (76.2)	19 (82.6)	16 (76.2)	15 (65.2)	22 (91.7)	19 (86.4)	25 (100)	17 (81.0)	15 (71.4)	46 (76.7)	45 (77.6)	48 (81.4)	16 (80.0)	50 (84.7)	44 (75.9)	45 (78.9)	13 (100)	28 (90.3)	27 (87.1)	26 (83.9)
Black/African American	4 (19.0)	3 (13.0)	2 (9.5)	6 (26.1)	1 (4.2)	2 (9.1)	0	4 (19.0)	3 (14.3)	8 (13.3)	11 (19.0)	7 (11.9)	3 (15.0)	5 (8.5)	5 (8.6)	8 (14.0)	0	3 (9.7)	2 (6.5)	3 (9.7)
Asian	1 (4.8)	1 (4.3)	1 (4.8)	0	0	0	0	0	0	2 (3.3)	1 (1.7)	1 (1.7)	0	0	1 (1.7)	1 (1.8)	0	0	1 (3.2)	0
American Indian or Alaska Native	0	0	0	0	0	0	0	0	0	1 (1.7)	1 (1.7)	0	0	0	1 (1.7)	0	0	0	0	0
Native Hawaiian or	0	0	0	0	0	0	0	0	0	0	0	0	0	1 (1.7)	0	0	0	0	0	0

Other Pacific Islander																				
Multiracial	0	0	0	0	0	1 (4.5)	0	0	2 (9.5)	2 (3.3)	0	1 (1.7)	0	1 (1.7)	2 (3.4)	0	0	0	0	0
Other	0	0	1 (4.8)	0	0	0	0	0	0	1 (1.7)	0	0	0	1 (1.7)	2 (3.4)	0	0	0	0	0
Not Reported	0	0	1 (4.8)	2 (8.7)	1 (4.2)	0	0	0	0	0	0	0	1 (5.0)	0	0	1 (1.8)	0	0	0	0
Unknown	0	0	0	0	0	0	0	0	0	0	0	0	0	0	0	0	0	0	0	0
Missing	0	0	0	0	0	0	0	0	1 (4.8)	0	0	2 (3.4)	0	1 (1.7)	3 (5.2)	2 (3.5)	0	0	1 (3.2)	2 (6.5)
**Ethnicity, n (%)**																				
Hispanic or Latino	4 (19.0)	7 (30.4)	5 (23.8)	4 (17.4)	1 (4.2)	5 (22.7)	4 (16.0)	4 (19.0)	5 (23.8)	12 (20.0)	20 (34.5)	17 (28.8)	5 (25.0)	12 (20.3)	10 (17.2)	9 (15.8)	2 (15.4)	4 (12.9)	2 (6.5)	3 (9.7)
Not Hispanic or Latino	17 (81.0)	16 (69.6)	16 (76.2)	18 (78.3)	22 (91.7)	17 (77.3)	21 (84.0)	17 (81.0)	16 (76.2)	47 (78.3)	38 (65.5)	40 (67.8)	15 (75.0)	47 (79.7)	47 (81.0)	47 (82.5)	11 (84.6)	27 (87.1)	29 (93.5)	28 (90.3)
Not Reported	0	0	0	1 (4.3)	0	0	0	0	0	0	0	1 (1.7)	0	0	1 (1.7)	1 (1.8)	0	0	0	0
Unknown	0	0	0	0	1 (4.2)	0	0	0	0	1 (1.7)	0	1 (1.7)	0	0	0	0	0	0	0	0
**Weight, kg**																				
Mean ± SD	84.8 ±16.3	82.8 ±17.1	78.9 ±13.5	76.5 ±14.8	78.6 ±14.6	84.4 ±15.9	81.0 ±15.1	75.8 ±10.0	91.2 ±35.5	90.6 ±24.5	91.7 ±24.6	93.0 ±25.9	84.0 ±22.1	91.5 ±21.1	90.2 ±22.0	90.1 ±28.9	83.7 ±15.9	81.0 ±18.4	86.4 ±20.8	84.9 ±19.2
**Height, cm**																				
Mean ± SD	172.0 ±9.4	172.1 ±9.5	169.4 ±8.1	166.6 ±8.7	168.9 ±8.4	170.1 ±10.8	169.6 ±10.4	165.9 ±7.9	169.9 ±12.9	169.8 ±10.4	168.6 ±10.0	170.9 ±10.7	169.2 ±10.9	169.1 ±8.3	171.5 ±10.0	168.6 ±10.2	163.2 ±10.0	167.3 ±9.4	165.4 ±10.1	167.0 ±10.3
**BMI, kg/m²**																				
Mean ± SD	28.6 ±4.5	27.8 ±4.4	27.4 ±3.6	27.5 ±4.3	27.4 ±4.1	29.1 ±4.2	28.1 ±4.1	27.6 ±3.9	31.0 ±10.1	31.5 ±8.5	32.4 ±8.8	31.8 ±8.6	29.2 ±6.4	31.9 ±6.6	30.7 ±7.4	31.5 ±8.7	31.4 ±4.9	28.8 ±5.6	31.3 ±5.3	30.5 ±6.8

**Received seasonal influenza vaccine, n (%)***																				
Yes	7 (33.3)	7 (30.4)	7 (33.3)	6 (26.1)	12 (50.0)	11 (50.0)	14 (56.0)	14 (66.7)	7 (33.3)	15 (25.0)	13 (22.4)	17 (28.8)	4 (20.0)	20 (33.9)	16 (27.6)	17 (29.8)	8 (61.5)	15 (48.4)	14 (45.2)	19 (61.3)
No	13 (61.9)	16 (69.6)	13 (61.9)	17 (73.9)	9 (37.5)	9 (40.9)	8 (32.0)	6 (28.6)	14 (66.7)	45 (75.0)	45 (77.6)	42 (71.2)	16 (80.0)	39 (66.1)	42 (72.4)	40 (70.2)	5 (38.5)	16 (51.6)	17 (54.8)	12 (38.7)
Unknown	1 (4.8)	0	1 (4.8)	0	3 (12.5)	2 (9.1)	3 (12.0)	1 (4.8)	0	0	0	0	0	0	0	0	0	0	0	0

BMI, body mass index; mRNA, messenger RNA; SD, standard deviation.

*Received seasonal influenza vaccine from August 2020 to December 2020.

Supplement Table 2. Summary of Influenza antibody responses by HAI through Day 29 by age group and study part (per-protocol population)

	Part 1								Part 2											
	18-49 Years of Age				≥50 Years of Age				18-49 Years of Age				50-64 Years of Age				≥65 Years of Age			
	Placebo (n=21)	50 µg mRNA-1010 (n=23)	100 µg mRNA-1010 (n=21)	200 µg mRNA-1010 (n=23)	Placebo (n=24)	50 µg mRNA-1010 (n=22)	100 µg mRNA-1010 (n=25)	200 µg mRNA-1010 (n=21)	Afluria (n=21)	25 µg mRNA-1010 (n=60)	50 µg mRNA-1010 (n=58)	100 µg mRNA-1010 (n=59)	Afluria (n=20)	25 µg mRNA-1010 (n=59)	50 µg mRNA-1010 (n=58)	100 µg mRNA-1010 (n=57)	Afluria (n=13)	25 µg mRNA-1010 (n=31)	50 µg mRNA-1010 (n=31)	100 µg mRNA -1010 (n=31)
**Influenza A H1N1 Antibodies by HAI Assay**																				
**Baseline (Day 1)**																				
n[a]	19	22	21	22	22	21	24	19	20	55	53	58	20	57	54	54	12	31	31	30
GMT (95% CI)	65.4 (34.5-123.8)	55.6 (30.3-102.3)	51.2 (32.6-80.2)	59.2 (36.4-96.4)	46.0 (25.9-81.9)	53.8 (33.6-86.2)	30.4 (20.8-44.5)	48.8 (26.1-91.0)	45.1 (25.0-81.4)	55.5 (42.3-72.8)	39.2 (27.6-55.6)	57.6 (43.3-76.6)	47.6 (23.8-95.3)	36.7 (27.1-49.7)	28.7 (20.8-39.4)	34.9 (26.2-46.6)	38.8 (17.3-87.3)	48.9 (34.0-70.2)	54.7 (34.9-85.7)	29.2 (21.5-39.8)
**Day 29**																				
n[b]	19	22	21	22	22	21	24	19	20	55	53	58	20	57	54	54	12	31	31	30
GMT (95% CI)	66.6 (34.5-128.9)	537.9 (265.4-1090.4)	572.9 (288.0-1139.7)	785.7 (419.9-1470.0)	52.3 (30.7-89.1)	309.6 (173.8-551.4)	219.9 (130.8-369.7)	436.3 (200.1-951.3)	246.7 (129.2-471.0)	591.8 (428.5-817.3)	613.7 (434.9-866.0)	807.2 (592.8-1099.1)	171.3 (102.9-285.3)	268.3 (194.7-369.9)	418.8 (295.7-593.1)	418.0 (301.7-579.1)	134.5 (59.2-305.9)	228.1 (133.2-390.5)	311.7 (187.5-518.4)	253.9 (157.7-408.8)
GMFR (95% CI)	1.0 (0.8-1.3)	9.7 (5.3-17.7)	11.2 (6.2-20.2)	13.3 (8.6-20.6)	1.1 (0.9-1.5)	5.8 (3.3-10.1)	7.2 (4.6-11.4)	8.9 (4.6-17.5)	5.5 (2.3-13.0)	10.7 (7.9-14.5)	15.7 (9.8-24.9)	14.0 (10.2-19.3)	3.6 (2.3-5.7)	7.3 (5.2-10.2)	14.6 (10.1-21.1)	12.0 (8.7-16.4)	3.5 (1.6-7.7)	4.7 (2.9-7.5)	5.7 (3.5-9.4)	8.7 (5.6-13.4)
Seroconversion,[c] n[d] (%) [95% CI]	0 [0.0-17.7]	17 (77.3) [54.6-92.2]	19 (90.5) [69.6-98.8]	21 (95.5) [77.2-99.9]	2 (9.1) [1.1-29.2]	12 (57.1) [34.0-78.2]	18 (75.0) [53.3-90.2]	16 (84.2) [60.4-96.6]	17 (85.0) [62.1-96.8]	48 (87.3) [75.5-94.7]	48 (90.6) [79.3-96.9]	50 (86.2) [74.6-93.9]	9 (45.0) [23.1-68.5]	39 (68.4) [54.8-80.1]	47 (87.0) [75.1-94.6]	49 (90.7) [79.7-96.9]	5 (41.7) [15.2-72.3]	16 (51.6) [33.1-69.9]	22 (71.0) [52.0-85.8]	23 (76.7) [57.7-90.1]
**Influenza A H3N2 Antibodies by HAI Assay**																				
**Baseline (Day 1)**																				

n[a]	19	22	21	22	22	21	24	19	20	55	53	58	20	57	54	54	12	31	31	30
GMT (95% CI)	69.0 (40.4-118.0)	66.1 (40.3-108.2)	59.4 (41.9-84.0)	90.6 (51.6-159.1)	43.2 (24.9-74.9)	41.3 (25.3-67.4)	42.9 (27.3-67.4)	60.8 (32.1-115.0)	22.6 (13.5-37.6)	33.9 (24.7-46.7)	26.5 (19.0-36.9)	46.2 (36.1-59.1)	28.7 (15.8-52.3)	20.9 (14.8-29.5)	18.8 (13.4-26.2)	17.8 (13.5-23.4)	69.2 (18.9-253.9)	28.9 (19.5-42.9)	33.5 (20.1-55.7)	21.9 (15.0-32.1)
**Day 29**																				
n[b]	19	22	21	22	22	21	24	19	20	55	53	58	20	57	54	54	12	31	31	30
GMT (95% CI)	81.3 (45.1-146.6)	529.7 (350.5-800.4)	371.0 (208.3-660.9)	674.2 (424.8-1070.0)	41.2 (24.5-69.6)	262.6 (162.1-425.4)	277.1 (191.8-400.4)	413.1 (253.5-673.0)	113.1 (78.0-164.1)	255.0 (185.3-350.9)	279.0 (203.1-383.3)	404.1 (319.5-511.0)	84.3 (55.5-128.2)	144.3 (102.9-202.4)	163.1 (114.3-232.8)	170.5 (116.9-248.8)	116.5 (47.0-288.7)	111.9 (69.2-181.0)	158.3 (97.5-257.1)	160.0 (102.0-250.8)
GMFR (95% CI)	1.2 (0.6-2.2)	8.0 (5.1-12.7)	6.2 (3.5-11.3)	7.4 (4.9-11.4)	1.0 (0.9-1.1)	6.4 (3.9-10.4)	6.5 (4.3-9.8)	6.8 (3.9-12.0)	5.01 (2.9-8.6)	7.5 (5.4-10.5)	10.5 (7.6-14.6)	8.8 (6.8-11.3)	2.9 (1.8-4.8)	6.9 (4.6-10.5)	8.7 (6.1-12.5)	9.6 (6.7-13.7)	1.7 (0.6-5.1)	3.9 (2.7-5.7)	4.7 (3.3-6.9)	7.3 (5.0-10.6)
Seroconversion,[c] n[d] (%) [95% CI]	1 (5.3) [0.1-26.0]	18 (81.8) [59.7-94.8]	16 (76.2) [52.8-91.8]	17 (77.3) [54.6-92.2]	0 [0.0-15.4]	17 (81.0) [58.1-94.6]	18 (75.0) [53.3-90.2]	14 (73.7) [48.8-90.9]	10 (50.0) [27.2-72.8]	42 (76.4) [63.0-86.8]	43 (81.1) [68.0-90.6]	48 (82.8) [70.6-91.4]	8 (40.0) [19.1-64.0]	40 (70.2) [56.6-81.6]	38 (70.4) [56.4-82.0]	42 (77.8) [64.4-88.0]	3 (25.0) [5.5-57.2]	12 (38.7) [21.9-57.8]	18 (58.1) [39.1-75.5]	22 (73.3) [54.1-87.7]
**Influenza B/Victoria Lineage Antibodies by HAI Assay**																				
**Baseline (Day 1)**																				
n[a]	19	22	21	22	22	21	24	18	20	55	53	58	20	57	54	54	12	31	31	30
GMT (95% CI)	143.3 (82.5-248.8)	113.1 (65.7-194.6)	109.4 (66.6-179.8)	136.7 (89.7-208.2)	93.5 (51.7-169.1)	131.3 (83.4-206.6)	81.1 (49.3-133.3)	139.6 (70.5-276.4)	33.7 (18.8-60.1)	44.8 (32.8-61.3)	30.4 (21.2-43.6)	36.5 (27.3-48.8)	68.4 (38.2-122.5)	50.7 (38.0-67.7)	55.1 (40.5-75.0)	53.0 (40.3-69.7)	50.4 (20.3-125.5)	51.7 (36.9-72.5)	59.8 (34.9-102.5)	60.6 (38.9-94.5)
**Day 29**																				
n[b]	19	22	21	22	22	21	24	19	20	55	53	58	20	57	54	54	12	31	31	30
GMT (95% CI)	126.1 (75.7-210.2)	260.7 (168.5-403.3)	179.6 (117.5-274.5)	313.2 (217.9-450.1)	99.6 (54.2-183.2)	215.3 (145.2-319.2)	168.7 (100.6-282.9)	247.8 (157.7-389.4)	160.1 (91.9-278.6)	99.7 (78.3-127.0)	119.1 (93.2-152.2)	115.1 (89.2-148.5)	183.9 (123.5-273.9)	131.4 (100.1-172.5)	181.9 (140.5-235.6)	149.1 (117.8-188.7)	127.1 (47.0-343.6)	97.9 (72.4-132.4)	104.6 (61.9-176.9)	128.6 (80.9-204.5)
GMFR (95% CI)	0.9 (0.6-1.4)	2.3 (1.7-3.1)	1.6 (1.0-2.7)	2.3 (1.6-3.2)	1.1 (0.9-1.2)	1.6 (1.3-2.1)	2.1 (1.4-3.1)	1.8 (1.0-3.1)	4.8 (2.0-11.1)	2.2 (1.8-2.8)	3.9 (3.0-5.2)	3.2 (2.6-3.8)	2.7 (1.6-4.5)	2.6 (2.1-3.2)	3.3 (2.5-4.3)	2.8 (2.3-3.5)	2.5 (0.8-8.0)	1.9 (1.5-2.4)	1.8 (1.4-2.1)	2.1 (1.7-2.7)

Seroconversion,[c] n[d] (%) [95% CI]	1 (5.3) [0.1-26.0]	4 (18.2) [5.2-40.3]	4 (19.0) [5.5-41.9]	6 (27.3) [10.7-50.2]	0 [0.0-15.4]	2 (9.5) [1.2-30.4]	3 (12.5) [2.7-32.4]	4 (22.2) [6.1-45.6]	8 (40.0) [19.1-64.0]	10 (18.2) [9.1-30.9]	21 (39.6) [26.5-54.0]	25 (43.9) [30.7-57.6]	6 (30.0) [11.9-54.3]	18 (31.6) [19.9-45.2]	21 (38.9) [25.9-53.1]	21 (38.9) [25.9-53.1]	3 (25.0) [5.5-57.2]	5 (16.1) [5.5-33.7]	4 (12.9) [3.6-29.8]	6 (20.0) [7.7-38.6]
**Influenza B/Yamagata Lineage Antibodies by HAI Assay**																				
**Baseline (Day 1)**																				
n[a]	19	22	21	22	22	21	24	19	20	55	53	58	20	57	54	54	12	31	31	30
GMT (95% CI)	157.1 (79.7-309.6)	159.9 (91.9-278.1)	137.8 (75.1-252.7)	181.5 (99.4-331.7)	85.2 (42.2-171.8)	95.9 (58.2-158.2)	57.4 (33.7-97.6)	86.0 (42.4-174.6)	95.1 (52.0-173.9)	89.0 (65.0-121.9)	60.0 (41.4-86.9)	79.1 (59.7-104.7)	61.6 (36.4-104.3)	63.1 (45.1-88.1)	59.1 (41.0-85.4)	65.1 (48.3-87.8)	42.3 (18.3-98.0)	61.2 (42.0-89.1)	100.0 (55.9-178.8)	54.1 (35.0-83.5)
**Day 29**																				
n[b]	19	22	21	22	22	21	24	19	20	55	53	58	20	57	54	54	12	31	31	30
GMT (95% CI)	143.3 (77.1-266.5)	467.1 (295.0-739.5)	383.7 (201.9-729.2)	610.5 (417.4-892.9)	78.8 (41.2-150.5)	304.5 (195.3-474.9)	279.5 (178.3-438.3)	325.8 (211.3-502.4)	387.0 (239.1-626.3)	314.0 (245.7-401.4)	309.7 (223.8-428.5)	309.9 (237.4-404.7)	165.6 (102.3-268.2)	206.6 (156.2-273.2)	226.3 (174.0-294.4)	255.7 (201.8-324.0)	100.9 (63.5-160.2)	191.4 (137.0-267.4)	204.6 (131.2-319.0)	151.0 (94.2-242.2)
GMFR (95% CI)	0.9 (0.7-1.2)	2.9 (2.0-4.2)	2.8 (1.8-4.2)	3.4 (1.9-5.9)	0.9 (0.8-1.0)	3.2 (2.1-4.8)	4.9 (3.0-8.0)	3.8 (2.3-6.2)	4.1 (2.1-8.0)	3.5 (2.7-4.7)	5.2 (3.6-7.4)	3.9 (3.0-5.1)	2.7 (1.7-4.3)	3.3 (2.6-4.1)	3.8 (3.0-5.0)	3.9 (3.2-4.9)	2.4 (1.2-4.7)	3.1 (2.1-4.6)	2.1 (1.5-2.8)	2.8 (2.0-3.9)
Seroconversion,[c] n[d] (%) [95% CI]	1 (5.3) [0.1-26.0]	9 (40.9) [20.7-63.7]	9 (42.9) [21.8-66.0]	12 (54.5) [32.2-75.6]	0 [0.0-15.4]	9 (42.9) [21.8-66.0]	13 (54.2) [32.8-74.5]	9 (47.4) [24.5-71.1]	8 (40.0) [19.1-64.0]	22 (40.0) [27.0-54.1]	30 (56.6) [42.3-70.2]	35 (60.3) [46.6-73.0]	5 (25.0) [8.7-49.1]	27 (47.4) [34.0-61.0]	29 (53.7) [39.6-67.4]	27 (50.0) [36.1-63.9]	4 (33.3) [9.9-65.1]	11 (35.5) [19.2-54.6]	7 (22.6) [9.6-41.1]	11 (36.7) [19.9-56.1]

The per-protocol population comprised all randomly assigned participants who received the vaccination and who did not have influenza infection at baseline through Day 29 and had no major protocol deviations that could impact the immune response.

CI, confidence interval; GMFR, geometric mean fold rise; GMT, geometric mean titer; HA, hemagglutinin; HAI, hemagglutination inhibition; LLOQ, lower limit of quantification; mRNA, messenger RNA.

[a]Number of subjects with non-missing baseline (Day 1) anti-HA antibody data.

[b]Number of subjects with non-missing anti-HA antibody data at the corresponding visit.

[c]Seroconversion at a subject level is defined as a Day 29 titer ≥1:40 if baseline is <1:10 or a 4-fold or greater rise if baseline is ≥1:10 in anti-HA antibodies.

[d]Number of subjects meeting the criterion at the corresponding visit. Percentage is based on the number of subjects with non-missing baseline (Day 1) anti-HA antibody (n^b rows).

CLINICAL STUDY PROTOCOL

**Protocol Title:**	A Phase 1/2, Randomized, Stratified, Observer-Blind, Dose-Ranging Study to Evaluate the Safety, Reactogenicity, and Immunogenicity of mRNA-1010 Seasonal Influenza Vaccine in Healthy Adults 18 Years and Older
**Protocol Number:**	mRNA-1010-P101
**Sponsor Name:**	ModernaTX, Inc.
**Legal Registered Address:**	200 Technology Square<br>Cambridge, MA 02139
**Sponsor Contact and Medical Monitor:**	[redacted]<br>ModernaTX, Inc.<br>200 Technology Square<br>Cambridge, MA 02139<br>Telephone: [redacted]<br>e-mail: [redacted]
**Regulatory Agency Identifier Number(s):**	IND 27460
**Amendment Number:**	4
**Date of Amendment 4:**	03 Feb 2022
**Date of Amendment 3:**	21 Oct 2021
**Date of Amendment 2:**	19 Aug 2021
**Date of Amendment 1:**	22 Jul 2021
**Date of Original Protocol:**	19 Apr 2021

CONFIDENTIAL

All financial and nonfinancial support for this study will be provided by ModernaTX, Inc. The concepts and information contained in this document or generated during the study are considered proprietary and may not be disclosed in whole or in part without the expressed written consent of ModernaTX, Inc. The study will be conducted according to the *International Council for Harmonisation (ICH) of Technical Requirements for Registration of Pharmaceuticals for Human Use, E6(R2) Good Clinical Practice (GCP) Guidance.*

PROTOCOL APPROVAL – SPONSOR SIGNATORY

Study Title: A Phase 1/2, Randomized, Stratified, Observer-Blind, Dose-Ranging Study to Evaluate the Safety, Reactogenicity, and Immunogenicity of mRNA-1010 Seasonal Influenza Vaccine in Healthy Adults 18 Years and Older

Protocol Number: mRNA-1010-P101

Amendment Number: 4

Approval Date: 03 Feb 2022

Protocol accepted and approved by:

See e-Signature and date at the end of the document

[redacted]<br>ModernaTX, Inc.<br>200 Technology Square<br>Cambridge, MA 02139<br>Telephone: [redacted]	Date

DECLARATION OF INVESTIGATOR

I have read and understood all sections of the protocol titled A Phase 1/2, Randomized, Stratified, Observer-Blind, Dose-Ranging Study to Evaluate the Safety, Reactogenicity, and Immunogenicity of mRNA-1010 Seasonal Influenza Vaccine in Healthy Adults 18 Years and Older and the most recent version of the investigator's brochure.

I agree to supervise all aspects of the protocol and to conduct the clinical investigation in accordance with the current protocol, the *International Council for Harmonisation (ICH) of Technical Requirements for Registration of Pharmaceuticals for Human Use, E6(R2) Good Clinical Practice (GCP) Guidance*, and all applicable government regulations. I will not make changes to the protocol before consulting with ModernaTX, Inc. or implement protocol changes without institutional review board (IRB) approval except to eliminate an immediate risk to participants.

I agree to administer study treatment only to participants under my personal supervision or the supervision of a subinvestigator. I will not supply study treatment to any person not authorized to receive it. I also agree that persons debarred from conducting or working on clinical studies by any court or regulatory agency will not be allowed to conduct or work on studies for the Sponsor or a partnership in which the Sponsor is involved. I will immediately disclose it in writing to the Sponsor if any person who is involved in the study is debarred, or if any proceeding for debarment is pending, or, to the best of my knowledge, threatened.

I will not disclose confidential information contained in this document, including participant information, to anyone other than the recipient study staffs and members of the IRB. I agree to ensure that this information will not be used for any purpose other than the evaluation or conduct of the clinical investigation without prior written consent from ModernaTX, Inc. I will not disclose information regarding this clinical investigation or publish results of the investigation without authorization from ModernaTX, Inc.

The signature below provides the necessary assurance that this study will be conducted according to all stipulations of the protocol, including statements regarding confidentiality, and according to local legal and regulatory requirements, United States federal regulations, and ICH E6(R2) GCP guidelines.

____________________________________ ____________________

Signature of Principal Investigator Date

____

Printed Name of Principal Investigator

Protocol Amendment Summary of Changes

DOCUMENT HISTORY	
**Document**	**Date**
Amendment 4	03 Feb 2022
Amendment 3	21 Oct 2021
Amendment 2	19 Aug 2021
Amendment 1	22 Jul 2021
Original Protocol	19 Apr 2021

Amendment 4, 03 Feb 2022 (Current Amendment)

Main Rationale for the Amendment:

The purpose of this amendment is to add the Phase 2 Extension part to test 3 dose levels of mRNA-1010 ([redacted]; Vaccination Groups 1 to 3).

The summary of changes table provided here describes the major changes made in Amendment 4 relative to Amendment 3, including the sections modified and the corresponding rationales. As applicable, the synopsis of Amendment 4 has been modified to correspond to changes in the body of the protocol. Minor editorial and/or formatting changes are not provided in this summary table.

Summary of Major Changes From Protocol Amendment 3 to Protocol Amendment 4:

Section # and Name	Description of Change	Brief Rationale
Synopsis: Overall Study Design; Section 4.1 (General Design); Section 4.2 (Scientific Rationale for Study Design); Section 6.1 (Investigational Products Administered)	• Added Phase 2 Extension part to the study to test dose levels of [redacted] or a single dose of a licensed quadrivalent seasonal influenza vaccine (active comparator).	• Based on interim analysis of the Phase 1/2 study, vaccination with mRNA-1010 elicited HAI antibodies at all dose levels tested. In the Phase 2 Extension part of the study, the Sponsor intends to test lower dose levels of [redacted] Thus, the Phase 2 Extension part will evaluate the immunogenicity of mRNA-1010 at proposed total dose levels of [redacted] in addition to an active comparator.
Synopsis: Number of Participants; Section 4.1 (General Design); Section 6.2 (Randomization); Section 9.3 (Sample Size Determination)	• Updated to describe enrollment and randomization of approximately 200 participants into the Phase 2 Extension part.	• Added per the addition of the Phase 2 Extension part.

Section # and Name	Description of Change	Brief Rationale
Synopsis: Study Eligibility Criteria; Section 5.2 (Phase 2 Northern Hemisphere and Phase 2 Extension)	• Updated to include inclusion and exclusion criteria for the Phase 2 Extension part.	• Added per the addition of the Phase 2 Extension part.
Synopsis: Study Treatments; Section 6 (Study Treatment)	• Updated to specify that the vaccine to be tested for the Phase 2 Extension part will be the same as the vaccine tested in the Phase 2 NH part.	• Added per the addition of the Phase 2 Extension part.
Synopsis: Procedures and Assessments; Section 8.1 (Safety Assessments and Procedures)	• Updated to reflect procedures and assessments in the Phase 2 Extension part.	• Added per the addition of the Phase 2 Extension part.
Synopsis: Statistical Methods (Sample Size); Section 9.3 (Sample Size Determination)	• Updated to describe the sample size for each group within the Phase 2 Extension part.	• Added per the addition of the Phase 2 Extension part.
Synopsis: Immunogenicity Analyses; Section 9.5.3 (Immunogenicity Analyses)	• Updated to define the rate of seroconversion for the Phase 2 NH part and to describe immunogenicity analyses in the Phase 2 Extension part.	• Updated for clarity and (Part 2 NH) and per the addition of the Phase 2 Extension part.
Section 1.2 (Schema), Figure 3	• Added this figure to present the study schema for the Phase 2 Extension part.	• Added per the addition of the Phase 2 Extension part.
Section 1.3 (Schedule of Events), Table 3	• Added this table as the Phase 2 Extension part Schedule of Events.	• Added per the addition of the Phase 2 Extension part.
Section 2.3.1 (Known Potential Benefits)	• Updated to describe potential benefit for participants enrolled in the Phase 2 Extension part.	• Added per the addition of the Phase 2 Extension part.
Section 2.3.3 (Overall Benefit/Risk Conclusion); Section 4.3 (Choice of Vaccine Dose)	• Updated to include rationale for doses in the Phase 2 Extension part.	• Added per the addition of the Phase 2 Extension part.
Section 3.2 (Phase 2 Northern Hemisphere and Phase 2 Extension)	• Updated to indicate objectives and endpoints for the Phase 2 Extension part are the same as those for the Phase 2 NH part.	• Added per the addition of the Phase 2 Extension part.
Section 6.3 (Preparation/Handling/ Storage/Accountability)	• Updated to describe investigational product that will be provided in the Phase 2 Extension part.	• Added per the addition of the Phase 2 Extension part.
Section 6.5.2 (Concomitant Medications and Therapies)	• Clarified and updated the timing of seasonal influenza vaccine that is considered concomitant therapy.	• Updated for clarity.
Section 6.6 (Intervention After End of the Study)	• Updated to indicate that no investigational product will be	• Updated for clarity.

Section # and Name	Description of Change	Brief Rationale
	provided to participants after the end of study.	
Section 7.1.2 (Pause Rules for the Phase 2 Northern Hemisphere and the Phase 2 Extension Parts of the Study	• Updated to indicate that pausing rules apply to both Phase 2 parts of the study.	• Added per the addition of the Phase 2 Extension part.
Section 8.1.1 (Use of Electronic Diaries)	• Removed language indicating that participants have a limited window on the following day to complete assessments for the previous day.<br>• Removed language specifically describing the treatment of new safety information reported during safety telephone calls or at site visits that was not already captured in the eDiary.	• Updated for practicality and clarity.
Section 8.1.6 (Assessments for Respiratory Viral Infections)	• Added description of NP swab sampling of participants in the Phase 2 Extension parts with signs or symptoms of CDC-defined ILI or of COVID-19).	• Updated for clarity.
Section 8.4.3 (Solicited Adverse Reactions)	• Added clarification of recording solicited adverse reactions.	• Updated for clarity.
Section 8.9 (Biomarkers)	• Updated to indicate that biomarker assessments will be evaluated in the Phase 2 Extension part.	• Added per the addition of the Phase 2 Extension part.

Abbreviations: COVID-19 = coronavirus disease 2019; HAI = hemagglutination inhibition; mRNA = messenger RNA; NH = Northern Hemisphere; NP = nasopharyngeal swab.

1. PROTOCOL SUMMARY

1.1. Synopsis

Name of Sponsor/Company: ModernaTX, Inc.

Name of Investigational Product: mRNA-1010

Name of Active Ingredient: mRNA-1010

Protocol Title: A Phase 1/2, Randomized, Stratified, Observer-Blind, Dose-Ranging Study to Evaluate the Safety, Reactogenicity, and Immunogenicity of mRNA-1010 Seasonal Influenza Vaccine in Healthy Adults 18 Years and Older

Protocol Number: mRNA-1010-P101; Amendment 4

Study Period: Approximately 18 months

Phase of Development: Phase 1/2

Objectives and Endpoints:

The objectives and endpoints for the Phase 1/2 part of the study are presented in the table below.

Objectives	Endpoints
Primary	
• To evaluate the safety and reactogenicity of 3 dose levels (50, 100, and 200 µg) of mRNA-1010 vaccine administered as a single dose	• Frequency and grade of each solicited local and systemic reactogenicity AR during a 7-day follow-up period after vaccination<br>• Frequency and severity of any unsolicited AEs during the 28-day follow-up period after vaccination<br>• Frequency of any SAEs, AESIs, and MAAEs from Day 1 through Day 181/EoS<br>• Safety laboratory abnormalities through 7 days after vaccination
• To evaluate the humoral immunogenicity of 3 dose levels (50, 100, and 200 µg) of mRNA-1010 vaccine administered as a single dose against vaccine-matched influenza A and B strains at Day 29	• GMT and GMFR at Day 29 compared with Day 1 (baseline) and percentage of participants with seroconversion, defined as a Day 29 titer ≥ 1:40 if baseline is < 1:10 or a 4-fold or greater rise if baseline is ≥ 1:10 in anti-HA antibodies measured by HAI assay

Secondary	
• To evaluate the humoral immunogenicity of 3 dose levels (50, 100, and 200 µg) of mRNA-1010 vaccine administered as a single dose against vaccine-matched influenza A and B strains at all evaluable humoral immunogenicity time points	• GMT and GMFR of anti-HA antibodies as measured by HAI or MN assays at all evaluable humoral immunogenicity time points compared with Day 1 (baseline)
Exploratory (May be Performed)	
[redacted]	

Abbreviations: AE = adverse event; AESI = adverse event of special interest; AR = adverse reaction; EoS = end of study; Fc = fragment crystallizable; GMFR = geometric mean fold rise; GMT = geometric mean titer; HA = hemagglutinin; HAI = hemagglutination inhibition; ILI = influenza-like illness; MAAE = medically attended adverse event; MN = microneutralization; RT-PCR = reverse transcription polymerase chain reaction; SAE = serious adverse event.

The objectives and endpoints for the Phase 2 Northern Hemisphere (NH) and the Phase 2 Extension parts of the study are presented in the table below.

Objectives	Endpoints
Primary	

• To evaluate the humoral immunogenicity of mRNA-1010 vaccine relative to that of an active comparator against vaccine-matched influenza A and B strains at Day 29	• GMT at Day 29 as measured by HAI<br>• Proportion of participants reaching seroconversion at Day 29 as measured by HAI
• To evaluate the safety and reactogenicity of mRNA-1010 vaccine	• Frequency and grade of each solicited local and systemic reactogenicity ARs during a 7-day follow-up period after vaccination<br>• Frequency and severity of any unsolicited AEs during the 28-day follow-up period after vaccination<br>• Frequency of any SAEs, AESIs, and MAAEs from Day 1 through Day 181/EoS
Secondary	
• To evaluate the humoral immunogenicity of each vaccine group against vaccine-matched influenza A and B strains at Day 29	• The frequency of participants with HAI seroconversion and the frequency of participants with an HAI titer ≥ 1:40 at Day 29<br>• GMFR comparing Day 29 to Day 1 (baseline) as measured by HAI
Exploratory (May be Performed)	
[redacted]	[redacted]

Abbreviations: AE = adverse event; AESI = adverse event of special interest; AR = adverse reaction; CDC = Centers for Disease Control and Prevention; EoS = end of study; GMFR = geometric mean fold rise; GMT = geometric mean titer;

HA = hemagglutinin; HAI = hemagglutination inhibition; ILI = influenza-like illness; MAAE = medically attended adverse event; MN = microneutralization; RT-PCR = reverse transcription polymerase chain reaction; SAE = serious adverse event.

Overall Study Design: This first-in-human study is a Phase 1/2, randomized, observer-blind, dose-ranging study to evaluate the safety, reactogenicity, and immunogenicity of mRNA-1010 vaccine in adult participants ≥ 18 years of age. The study comprises 3 parts: Phase 1/2, Phase 2 NH, and Phase 2 Extension.

The Phase 1/2 part of the study will be a randomized, observer-blind, dose-ranging, placebo-controlled study to evaluate the safety, reactogenicity, and immunogenicity of mRNA-1010 vaccine in healthy adult participants ≥ 18 years of age. The Phase 2 NH and Phase 2 Extension parts of the study will be randomized, observer-blind, dose-ranging, active-controlled studies to evaluate the immunogenicity, reactogenicity, and safety of mRNA-1010 vaccine in medically stable adults 18 years and older.

All participants in each part of the study will participate in a screening period (up to 28 days before Day 1), treatment period (single dose of vaccine on Day 1), and a follow-up period (up to 6 months after vaccination).

In the Phase 1/2 part of the study, the mRNA-1010 vaccine to be tested includes messenger RNAs (mRNAs) that encode for the surface glycoprotein hemagglutinins (HAs) of the following influenza virus strains recommended by the World Health Organization (WHO) for 2020-2021 Southern Hemisphere (SH) cell- or recombinant-based vaccines:

- A/Wisconsin/588/2019(H1N1)pdm09
- A/Hong Kong/45/2019(H3N2)
- B/Washington/02/2019 (B/Victoria lineage)
- B/Phuket/3073/2013 (B/Yamagata lineage)

In the Phase 2 NH and Phase 2 Extension parts of the study, the mRNA-1010 vaccine to be tested, includes mRNAs that encode for the surface glycoprotein HAs of the following influenza virus strains recommended by the WHO for 2021-2022 NH cell- or recombinant-based vaccines:

- A/Wisconsin/588/2019(H1N1)pdm09
- A/Cambodia/e0826360/2020 (H3N2)
- B/Washington/02/2019 (B/Victoria lineage)
- B/Phuket/3073/2013 (B/Yamagata lineage)

Different dose levels of mRNA-1010 vaccine will be evaluated. Placebo will be included in the Phase 1/2 part and an active comparator (licensed quadrivalent seasonal influenza vaccine) will

be included in the Phase 2 NH and Phase 2 Extension parts. In the Phase 1/2 part, each vaccination group will have 2 stages: initial stage and expansion stage.

The tables below list the vaccination groups and dose levels that will be evaluated in each part of the study, as well as the total number of study participants in each study part and overall.

Phase 1/2

				Number of Participants		
				Phase 1/2		
Vaccination Group	Investigational Product	mRNA or HA (µg)	Total Dose (µg)	Initial Stage	Expansion Stage	Total
1	mRNA-1010	12.5	50	9	36	45
2	mRNA-1010	25	100	9	36	45
3	mRNA-1010	50	200	9	36	45
4	Placebo	–	–	9	36	45
**Total**				**36**	**144**	**180**

Abbreviations: HA = hemagglutinin; mRNA = messenger RNA.

Phase 2 NH

				Number of Participants
Vaccination Group	Investigational Product	mRNA or HA (µg)	Total Dose (µg)	Phase 2 NH
1	mRNA-1010	6.25	25	150
2	mRNA-1010	12.5	50	150
3	mRNA-1010	25	100	150
4	Active comparator	15	60	50
**Total**				**500**

Abbreviations: HA = hemagglutinin; mRNA = messenger RNA; NH = Northern Hemisphere.

Phase 2 Extension

			Number of Participants
Vaccination Group	Investigational Product	Total Dose (µg)	Phase 2 Extension
1	mRNA-1010	[redacted]	50
2	mRNA-1010	[redacted]	50
3	mRNA-1010	[redacted]	50
4	Active comparator	60	50
**Total**			**200**

Abbreviations: HA = hemagglutinin; mRNA = messenger RNA.
Note: Mass percent in mRNA-1010 is 25% for each of the 4 HAs.

Total Number of Participants

Study Part	Number of Participants
Phase 1/2	180
Phase 2 NH	500
Phase 2 Extension	200
**Total**	**880**

Abbreviations: NH = Northern Hemisphere.

Healthy (Phase 1/2) and medically stable (Phase 2 NH and Phase 2 Extension) adults ≥ 18 years of age will be screened and enrolled. For the Phase 2 NH and Phase 2 Extension parts, participants with chronic diseases requiring ongoing medical intervention within the last 3 months prior to enrollment as well as those with immunocompromising conditions or medications will be excluded.

In the Phase 1/2 part, approximately 180 participants will be randomly assigned in a 1:1:1:1 ratio to receive mRNA-1010 50 µg, mRNA-1010 100 µg, mRNA-1010 200 µg, or placebo, with approximately 45 participants randomly assigned to each vaccination group. A total of 36 participants (9 participants in each vaccination group) will be randomly assigned in the initial stage of the study. The internal safety team (IST) will perform a blinded review of all safety data up to 7 days after vaccination from the 36 participants in the initial stage. After the IST confirms that no pause rules have been met in the 36 participants in the initial stage, enrollment will begin in the expansion stage. A total of 144 participants (36 participants in each vaccination group) will be randomly assigned in the expansion stage. Randomization in the expansion stage will be stratified by age (18 to < 50 years versus ≥ 50 years) and will be balanced across the 2 age groups within each vaccination group.

The Phase 2 NH part of the study will enroll and randomize approximately 500 participants in a 3:3:3:1 ratio to 1 of 4 vaccination groups to receive a single dose of mRNA-1010 at different dose levels (25, 50, or 100 µg total mRNA) or a single dose of a licensed quadrivalent seasonal influenza vaccine (active comparator). Randomization to individual vaccination groups will proceed with parallel randomization among the 3 dose levels of mRNA-1010 (25, 50, and 100 total mRNA; Vaccination Groups 1 to 3) and the active comparator (Vaccination Group 4). Randomization will be stratified by age categories (18 to < 50 years, 50 to < 65 years, or ≥ 65 years) and vaccination status in the previous flu season (received or not received).

The Phase 2 Extension part of the study will enroll and randomize approximately 200 participants in a 1:1:1:1 ratio to 1 of 4 vaccination groups to receive a single dose of mRNA-1010 at different dose levels [redacted] or a single dose of a licensed quadrivalent seasonal influenza vaccine (active comparator). Randomization to

individual vaccination groups will proceed with parallel randomization among the 3 dose levels of mRNA-1010 ([redacted]; Vaccination Groups 1 to 3) and the active comparator (Vaccination Group 4). Randomization will be stratified by age categories (18 to < 50 years, or ≥ 50 years).

The Phase 1/2 part of the study comprises 6 scheduled study site visits: screening, Day 1, Day 8, Day 29 (Month 1), Day 57 (Month 2), and Day 181 (Month 6). The Phase 2 NH and Phase 2 Extension parts of the study comprise 5 scheduled study site visits: Screening, Day 1, Day 29 (Month 1), Day 91 (Month 3), and Day 181 (Month 6). The Phase 2 Extension part also contains an optional study site visit on Day 4 for blood sample collection. There are also scheduled safety phone calls to collect medically attended adverse events (MAAEs), adverse events (AEs) leading to withdrawal from study participation, serious AEs (SAEs), AEs of special interest (AESIs), information about concomitant medications associated with these events, and information about receipt of nonstudy vaccinations temporally associated with these events. These phone calls are scheduled monthly from Day 91 through Day 151 in the Phase 1/2 part and on Days 8 and 15 in the Phase 2 NH and Phase 2 Extension parts of the study.

The study duration will be approximately 7 months for each participant enrolled in each part of the study: a screening period of up to 1 month and a study period of 6 months that includes a single dose of vaccine on Day 1. The participant's final visit (ie, end of study [EoS]) will be on Day 181 (Month 6), six months after the study vaccination.

All participants in each part of the study will be followed for safety and reactogenicity. On Day 1, participants will be instructed how to document, and report solicited adverse reactions (ARs) and influenza-like illness (ILI) symptoms (for Phase 2 NH part only) with a provided electronic diary (eDiary). Solicited ARs will be assessed for 7 days (the day of injection and the following 6 days) after the study vaccination, and unsolicited AEs will be assessed for 28 days after the study vaccination; SAEs, AESIs, and MAAEs will be assessed from Day 1 throughout the study.

All participants in the Phase 1/2 part of the study will provide nasopharyngeal (NP) swab samples before the injection on Day 1 for assessment of asymptomatic infection with respiratory pathogens, including influenza virus and severe acute respiratory syndrome coronavirus 2 (SARS-CoV-2). A study site visit or a consultation (unscheduled study visit) will be arranged within 24 hours or as soon as possible to collect an NP swab sample if participants experience symptoms consistent with the United States (US) Centers for Disease Control and Prevention (CDC) definition of ILI and/or coronavirus disease 2019 (COVID-19) during the course of the study.

For the Phase 2 NH part, as part of the safety assessments, active and passive surveillance for ILI will be conducted between Day 1 through Day 181/EoS. For active surveillance, participants will be instructed to report whether ILI symptoms have been experienced, 3 to 4 times weekly from Day 1 through Day 29 and twice weekly from Day 30 through Day 181/EoS, via eDiary or telephone calls. If symptoms occur, participants will be directed to return to the study site as soon as possible, but no later than 72 hours after the onset of symptoms, for medical evaluation and an NP swab. Participants will be contacted by the study site if they have missed reporting in the eDiary. If there is no response to an eDiary prompt for 2 consecutive entries, the study site staff will contact the participant by telephone. For passive surveillance, participants will be instructed to report symptoms of ILI any time from Day 1 through Day 181/EoS. Participants in the Phase 2 NH part who manifest protocol-defined ILI between Day 1 through Day 29 will be evaluated by real-time reverse transcription polymerase chain reaction (RT-PCR) testing of an NP specimen for influenza (and other respiratory pathogens). An NP swab specimen for pathogens, including influenza virus and SARS-CoV-2 will be collected any time from Day 1 through Day 181/EoS if participants have protocol-defined ILI or symptoms suggestive of COVID-19 or other upper or lower respiratory infection at the Investigator's discretion.

For the Phase 2 Extension part, a study site visit or a consultation (unscheduled study visit) will be arranged within 72 hours after the onset of symptoms to collect an NP swab sample if participants experience symptoms consistent with the US CDC definition of ILI and/or COVID-19 during the course of the study.

All participants in the Phase 1/2 part of the study will provide a blood specimen before injection on Day 1 and additional blood specimens through the next 6 months for immunogenicity (on Days 8, 29, and 181/EoS). In the Phase 2 NH and Phase 2 Extension parts, all participants will provide a blood specimen for immunogenicity assessments before injection on Day 1 and then on Day 29. In addition, blood samples from a subset of participants at Days 91 and 181/EoS may be used for additional assessments. The Phase 2 Extension part also contains an optional study site visit on Day 4 for blood sample collection for potential biomarker analysis. Furthermore, blood samples for safety or other medical concerns may be collected according to the Investigator's judgment at scheduled study site visits during each part of the study.

Participants may experience AEs that necessitate an unscheduled visit. There may also be situations in which the Investigator asks a participant to report for an unscheduled visit following the report of an AE. Additional examinations may be conducted at these visits as necessary to ensure the safety and well-being of participants during the study. Electronic case report forms should be completed for each unscheduled visit.

The study will be observer-blind as to which investigational product (IP; mRNA-1010 or placebo in the Phase 1/2 part; mRNA-1010 or the active comparator in the Phase 2 NH and Phase 2 Extension parts) or dose level is administered. Participants will receive the IP by 0.5-mL intramuscular (IM) injection on Day 1. All participants, study site staff involved in participant assessment, and Sponsor personnel (or its designees) will be blinded to individual dosing assignment until the study database is locked and unblinded. Preparation of the IP for administration will be conducted on site by an unblinded staff member who has no role in the observation or assessment of study participants.

For the Phase 1/2 part, there is 1 planned interim analysis (IA), which will include safety and immunogenicity data of all participants (all vaccination groups) after completion of the Day 29 Visit. For the Phase 2 NH and Phase 2 Extension parts, an IA of safety (reactogenicity and immunogenicity as applicable) will be performed after participants have completed the Day 29 Visit for each part of the study.

Immunizations for the Phase 1/2 part of the study are planned during the NH summer and all participants will be recommended to receive a licensed 2021-22 NH seasonal influenza vaccine during their participation in the study after completion of the Day 29 Visit. Immunizations for the Phase 2 NH part of the study are planned during the NH influenza season. Immunizations for the Phase 2 Extension part are planned during the NH spring.

Safety Oversight: Safety monitoring for this study will include blinded study team members, inclusive of, at a minimum, the Sponsor’s medical monitor, medical monitor of the contract research organization (CRO), a blinded IST, and an unblinded Data and Safety Monitoring Board (DSMB). The study team will conduct ongoing blinded safety reviews during the study and will be responsible for notifying the IST and DSMB of potential safety signal events or the triggering of pause rules.

The IST will consist of at least 2 Sponsor physicians who will be voting members not directly involved in the clinical development of the study. For the initial stage in the Phase 1/2 part of the study, the IST will conduct a blinded review of all available safety data after at least 36 participants (9 participants per vaccination group) have completed their Day 8 Visit. Vaccination of the remaining participants in the expansion stage will continue in each vaccination group if no pause rules have been met and the safety and tolerability are acceptable. Prior to the start of the Phase 2 NH part of the study, the IST will also conduct a blinded review of available safety data from the Phase 1/2 part. Finally, the IST will also conduct ad hoc reviews as requested by the study medical monitor and the study team.

An independent unblinded DSMB will be used throughout the conduct of this study. This committee will be composed of independent members with relevant therapeutic and/or

biostatistical expertise to allow for the ongoing unblinded review of safety data from this study population. Safety data will be reviewed according to intervals defined in the DSMB charter and will also occur as needed when study stopping or pausing criteria are met, or as otherwise requested by the study team and/or IST.

Details regarding the IST and DSMB composition, responsibilities, procedures, and frequency of data review will be defined in their respective charters.

Study Duration: Approximately 7 months for each participant in each part of the study.

Number of Participants: Approximately 180 participants will be enrolled and randomized in the Phase 1/2 part of the study. The Phase 2 NH part will enroll and randomize approximately 500 participants. The Phase 2 Extension part will enroll and randomize approximately 200 participants. There will be a total of 880 participants in the entire study.

Study Eligibility Criteria

Phase 1/2 Inclusion Criteria:

Each participant must meet all of the following criteria at the Screening Visit and at Day 1, unless noted otherwise, to be enrolled in the study:

1. Is an adult, ≥ 18 years of age at the time of consent (Screening Visit), who is in good health, in the opinion of the Investigator, based on review of medical history and physical examination performed at screening.
2. Understands and is willing and physically able to comply with protocol-mandated follow-up, including all procedures, in the opinion of the Investigator.
3. Has provided written informed consent for participation in this study, including all evaluations and procedures as specified in this protocol.
4. Has a body mass index of 18 kg/m^2 to 35 kg/m^2 (inclusive) at the Screening Visit.
5. If female and is not of childbearing potential, may be enrolled in the study. A follicle-stimulating hormone (FSH) level may be measured at the discretion of the Investigator to confirm postmenopausal status.
6. If female and of childbearing potential, meets all of the following criteria to be enrolled in the study:
    - Has a negative pregnancy test at the Screening Visit and on the day of vaccination (Day 1).

- Has practiced adequate contraception or has abstained from all activities that could result in pregnancy for at least 28 days prior to Day 1. Adequate female contraception is defined as consistent and correct use of a Food and Drug Administration (FDA)-approved contraceptive method in accordance with the product label.
- Has agreed to continue adequate contraception through 3 months following vaccine administration.
- Is not currently breastfeeding.

Phase 1/2 Exclusion Criteria:

Participants meeting any of the following criteria at the Screening Visit or at Day 1, unless noted otherwise, will be excluded from the study:

1. Has had significant exposure to someone with laboratory-confirmed SARS-CoV-2 infection, COVID-19, or ILI in the past 14 days prior to the Screening Visit, as defined by the CDC as a close contact with someone who has COVID-19.
2. Has a positive SARS-CoV-2 RT-PCR or antigen test in the past 10 days prior to the Screening Visit.
   a. A participant who has a positive SARS-CoV-2 serological test in the past 10 days prior to the Screening Visit but is otherwise asymptomatic and does not have a positive SARS-CoV-2 RT-PCR or antigen test at the Screening Visit may still be enrolled if they meet the study eligibility criteria.
   b. A participant who has a positive SARS-CoV-2 RT-PCR or antigen test in the past 10 days prior to the Screening Visit may be enrolled 10 days after symptom onset or 10 days after the date of the first positive SARS-CoV-2 RT-PCR test without repeat testing, provided the participant is asymptomatic and meets the study eligibility criteria as well as the CDC recommendation for duration of isolation and precautions.
   c. Additionally, a participant who meets all study eligibility criteria (note exclusion criterion No. 4) at Screening Visit but later tests positive for SARS-CoV-2 at Visit 1 will continue participation in the study.
3. Has clinical screening laboratory values (white blood cell count, hemoglobin, platelets, alanine aminotransferase, aspartate aminotransferase, creatinine, alkaline phosphatase, and total bilirubin) > Grade 1.
4. Is acutely ill or febrile (body temperature ≥ 38.0°C/100.4°F) 72 hours prior to or at the Screening Visit or Day 1. Participants meeting this criterion may be rescheduled within

the 28-day screening window and will retain their initially assigned participant number.

5. Has a pre-existing medical condition that is not stable, at the discretion of the Investigator. A stable medical condition is defined as disease not requiring significant change in therapy or hospitalization for worsening disease during the 2 months before enrollment. Participants may be rescreened if they are not medically stable at the Screening Visit. Medical conditions include (but are not limited to) the following:
    a. Uncontrolled hypertension or systolic blood pressure (BP) > 150 mm Hg or diastolic BP > 90 mm Hg at the Screening Visit.
    b. Congestive heart failure.
    c. Unstable angina or exacerbation of coronary artery disease within 6 months before the day of vaccination (Day 1) requiring cardiac intervention or new cardiac medications to control symptoms.
    d. Diabetes requiring the use of medicine (injectable or oral) or not controlled with diet.
    e. Chronic obstructive pulmonary disease, asthma requiring daily use of a bronchodilator or inhaled/systemic corticosteroids, or other chronic lung diseases such as pulmonary fibrosis.
6. Has a medical, psychiatric, or occupational condition that may pose additional risk as a result of participation or that could interfere with safety assessments or interpretation of results according to the Investigator's judgment.
7. Has a current or previous diagnosis of immunocompromising condition, immune-mediated disease, or other immunosuppressive condition.
8. Has received systemic immunosuppressants or immune-modifying drugs for > 14 days in total within 6 months prior to Screening Visit (for corticosteroids ≥ 10 mg/day of prednisone or equivalent) or is anticipating the need for immunosuppressive treatment at any time during participation in the study.
9. Has a history of anaphylaxis, urticaria, or other significant AR requiring medical intervention after receipt of a vaccine or any of the components contained in the IP.
10. Has a history of coagulopathy or bleeding disorder considered a contraindication to IM injection or phlebotomy.
11. Has received or plans to receive any licensed vaccine ≤ 28 days prior to the IP injection (Day 1) or plans to receive a licensed vaccine within 28 days after the IP injection, with the exception of vaccines authorized or approved for the prevention of COVID-19

(regardless of type of vaccine) that become available to participants during the study. Efforts should be made to space study vaccination and COVID-19 vaccination by at least 7 and preferably 14 days, but COVID-19 vaccination should not be delayed.

12. Has received a seasonal influenza vaccine or any other investigational influenza vaccine after 01 Jan 2021.
13. Has received systemic immunoglobulins or blood products within 3 months prior to the Screening Visit or plans to receive them during the study.
14. Has a diagnosis of malignancy within the previous 10 years (excluding nonmelanoma skin cancer).
15. Has donated ≥ 450 mL of blood products within 28 days prior to the Screening Visit or plans to donate blood products during the study.
16. Has participated in an interventional clinical study within 28 days prior to the Screening Visit based on the medical history interview or plans to do so while participating in this study.
17. Is an immediate family member or household member of study personnel, study site staff, or Sponsor personnel.

Phase 2 NH and Phase 2 Extension Inclusion Criteria:

Each participant must meet all of the following criteria at the Screening Visit and at Day 1, unless noted otherwise, to be enrolled in this study:

1. Is an adult, ≥ 18 years of age at the time of consent (Screening Visit), who in the opinion of the Investigator, is medically stable based on review of medical history and physical examination performed at screening. Medically stable is defined as disease not requiring significant change in therapy or hospitalization for worsening disease during the 3 months before enrollment. Participants may be rescreened if they are not medically stable at the Screening Visit.
2. Understands and is willing and physically able to comply with protocol-mandated follow-up, including all procedures, in the opinion of the Investigator.
3. Has provided written informed consent for participation in this study, including all evaluations and procedures as specified in this protocol.
4. If female and is not of childbearing potential, may be enrolled in the study. An FSH level may be measured at the discretion of the Investigator to confirm postmenopausal status (Section 11.2).

5. If female and of childbearing potential, meets all of the following criteria to be enrolled in the study:
   - Has a negative pregnancy test at the Screening Visit and on the day of vaccination (Day 1).
   - Has practiced adequate contraception or has abstained from all activities that could result in pregnancy for at least 28 days prior to Day 1. Adequate female contraception is defined as consistent and correct use of an FDA-approved contraceptive method in accordance with the product label.
   - Has agreed to continue adequate contraception through 3 months following vaccine administration.
   - Is not currently breastfeeding.

Phase 2 NH Exclusion Criteria:

Participant meeting any of the following criteria at the Screening Visit or at Day 1, unless noted otherwise, will be excluded from the study:

1. Has had close contact to someone with SARS-CoV-2 infection or COVID-19 as defined by the CDC in the past 14 days prior to the Screening Visit, unless the participant has been fully vaccinated for COVID-19.
2. Is acutely ill or febrile (body temperature ≥ 38.0°C/100.4°F) 72 hours prior to or at the Screening Visit or Day 1. Participants meeting this criterion may be rescheduled within the 28-day screening window and will retain their initially assigned participant number.
3. Has a medical, psychiatric, occupational condition, or history of substance abuse that may pose additional risk as a result of participation or that could interfere with safety assessments or interpretation of results according to the Investigator's judgment.
4. Has a current or previous diagnosis of immunocompromising/immunosuppressive condition, immune-mediated disease requiring immune-modifying therapy, asplenia, recurrent severe infections (human immunodeficiency virus [HIV]-positive participants on antiretroviral therapy with cluster of differentiation [CD] 4 count ≥ 350 cells/mm^3 and HIV-RNA ≤ 500 copies/mL within the past 12 months are permitted).
5. Has received systemic immunosuppressants or immune-modifying drugs for > 14 days in total within 6 months prior to Screening Visit (for corticosteroids ≥ 10 mg/day of

prednisone or equivalent) or is anticipating the need for systemic immunosuppressive treatment at any time during participation in the study.

6. Has a history of anaphylaxis, urticaria, or other significant AR requiring medical intervention after receipt of a vaccine or any of the components contained in the mRNA-1010 or the comparator vaccine, which is an egg-based influenza vaccine.
7. Has coagulopathy or bleeding disorder considered a contraindication to IM injection or phlebotomy.
8. Has received or plans to receive any licensed vaccine ≤ 28 days prior to the IP injection (Day 1) or plans to receive a licensed vaccine within 28 days after the IP injection, with the exception of vaccines authorized or approved for the prevention of COVID-19 (regardless of type of vaccine) that become available to participants during the study. Efforts should be made to space study vaccination and COVID-19 vaccination by at least 7 and preferably 14 days, but COVID-19 vaccination should not be delayed.
9. Has received a seasonal influenza vaccine or any other investigational influenza vaccine within 6 months prior to the Screening Visit.
10. Had tested positive for influenza by CDC-recommended testing methods within 6 months prior to the Screening Visit.
11. Has received systemic immunoglobulins or blood products within 3 months prior to the Screening Visit or plans to receive them during the study.
12. Has donated ≥ 450 mL of blood products within 28 days prior to the Screening Visit or plans to donate blood products during the study.
13. Has participated in an interventional clinical study within 28 days prior to the Screening Visit based on the medical history interview or plans to do so while participating in this study.
14. Is an immediate family member or household member of study personnel, study site staff, or Sponsor personnel.

Phase 2 Extension Exclusion Criteria:

Participant meeting any of the following criteria at the Screening Visit or at Day 1, unless noted otherwise, will be excluded from the study:

1. Has had close contact to someone with SARS-CoV-2 infection or COVID-19 as defined by the CDC in the past 10 days prior to the Screening Visit.

2. Is acutely ill or febrile (body temperature ≥ 38.0°C/100.4°F) 72 hours prior to or at the Screening Visit or Day 1. Participants meeting this criterion may be rescheduled within the 28-day screening window and will retain their initially assigned participant number.

3. Has a medical, psychiatric, occupational condition, or history of substance abuse that may pose additional risk as a result of participation or that could interfere with safety assessments or interpretation of results according to the Investigator's judgment.

4. Has a current or previous diagnosis of immunocompromising/immunosuppressive condition, immune-mediated disease requiring immune-modifying therapy, asplenia, recurrent severe infections (HIV-positive participants on antiretroviral therapy with CD 4 count ≥ 350 cells/mm^3 and HIV-RNA ≤ 500 copies/mL within the past 365 days are permitted).

5. Has received systemic immunosuppressants or immune-modifying drugs for > 14 days in total within 180 days prior to Screening Visit (for corticosteroids ≥ 10 mg/day of prednisone or equivalent) or is anticipating the need for systemic immunosuppressive treatment at any time during participation in the study.

6. Has a history of anaphylaxis, urticaria, or other significant AR requiring medical intervention after receipt of a vaccine or any of the components contained in the mRNA-1010 or the comparator vaccine, which is an egg-based influenza vaccine.

7. Has coagulopathy or bleeding disorder considered a contraindication to IM injection or phlebotomy.

8. Has received or plans to receive any licensed vaccine ≤ 28 days prior to the IP injection (Day 1) or plans to receive a licensed vaccine within 28 days after the IP injection, with the exception of vaccines authorized or approved for the prevention of COVID-19 (regardless of type of vaccine) that become available to participants during the study. Efforts should be made to space study vaccination and COVID-19 vaccination by at least 7 and preferably 14 days, but COVID-19 vaccination should not be delayed.

9. Has received a seasonal influenza vaccine or any other investigational influenza vaccine within 180 days prior to the randomization visit.

10. Had tested positive for influenza by CDC-recommended testing methods within 180 days prior to the Screening Visit.

11. Has received systemic immunoglobulins or blood products within 90 days prior to the Screening Visit or plans to receive them during the study.

12. Has donated ≥ 450 mL of blood products within 28 days prior to the Screening Visit or plans to donate blood products during the study.
13. Has participated in an interventional clinical study within 28 days prior to the Screening Visit based on the medical history interview or plans to do so while participating in this study.
14. Is an immediate family member or household member of study personnel, study site staff, or Sponsor personnel.

Study Treatments

Investigational Product, Dosage, and Mode of Administration:

Investigational products in the Phase 1/2 part of the study are the mRNA-1010 vaccine candidate and placebo. Investigational products administered in the Phase 2 NH and Phase 2 Extension parts of the study are the mRNA-1010 vaccine candidate and the licensed quadrivalent seasonal influenza vaccine (active comparator).

In the Phase 1/2 part of the study, the mRNA-1010 vaccine to be tested includes mRNAs that encode for the surface glycoprotein HAs of the following influenza virus strains recommended by the WHO for 2020-2021 SH cell- or recombinant-based vaccines:

- A/Wisconsin/588/2019(H1N1)pdm09
- A/Hong Kong/45/2019(H3N2)
- B/Washington/02/2019 (B/Victoria lineage)
- B/Phuket/3073/2013 (B/Yamagata lineage)

In the Phase 2 NH and Phase 2 Extension parts of the study, the mRNA-1010 vaccine to be tested includes mRNAs that encode for the surface glycoprotein HAs of the following influenza virus strains recommended by the WHO for 2021-2022 NH cell- or recombinant-based vaccines:

- A/Wisconsin/588/2019(H1N1)pdm09
- A/Cambodia/e0826360/2020 (H3N2)
- B/Washington/02/2019 (B/Victoria lineage)
- B/Phuket/3073/2013 (B/Yamagata lineage)

mRNA-1010 is formulated in lipid nanoparticles composed of 4 lipids (1 proprietary and 3 commercially available): [redacted]

[redacted] The mRNA-1010 injection is provided as sterile liquid for injection and is a white to off-white dispersion at a concentration of 0.5 mg/mL (in the Phase 1/2 part) or 0.4 mg/mL (in the Phase 2 NH and Phase 2 Extension parts) in 20 mM Tris buffer containing 87 mg/mL sucrose and 10.7 mM sodium acetate at pH 7.5.

In each part of the study, the mRNA-1010 vaccine will be administered as a single 0.5-mL IM injection at total dose levels ranging from 6.25 to 200 µg to participants according to the vaccination group assignment.

The placebo (administered in Phase 1/2 part only) is 0.9% Sodium Chloride (normal saline) Injection, which meets the criteria of the US Pharmacopeia.

The active comparator vaccine (administered in Phase 2 NH and Phase 2 Extension parts only) will be administered as a single 0.5 mL IM injection to participants per the vaccination group assignment.

Procedures and Assessments:

Safety Assessments:

Safety assessments will include monitoring and recording of the following for each participant:

- Solicited local and systemic ARs that occur during the 7 days following vaccine administration (ie, the day of injection and 6 subsequent days). Solicited ARs will be recorded daily using eDiaries.
- Unsolicited AEs observed or reported during the 28 days following vaccine administration (ie, the day of injection and 27 subsequent days).
- AEs leading to discontinuation from study participation from Day 1 through Day 181/EoS or withdrawal from the study.
- MAAEs from Day 1 through Day 181/EoS or withdrawal from the study.
- SAEs and AESIs from Day 1 through Day 181/EoS or withdrawal from the study.
- Results of safety laboratory tests (Phase 1/2 part only).
- Vital sign measurements.
- Physical examination findings.
- Assessments for causes of respiratory infections as needed from Day 1 through Day 181/EoS in the Phase 1/2 and Phase 2 Extension parts of the study or withdrawal from the study. Active and passive surveillance for ILI (and assessments for causes of respiratory infections as needed) will be conducted from Day 1 through Day 181/EoS in the Phase 2 NH part of the study.
- Details of all pregnancies in female participants will be collected after the study vaccination on Day 1 and until the end of their participation in the study.
- Optional blood sample collection on Day 4 in the Phase 2 Extension part.

Immunogenicity Assessments:

The following immunogenicity assessments are planned:

- Serum antibody level as measured by hemagglutination inhibition (HAI) assay (primary, secondary, and exploratory endpoints).
- Serum neutralizing antibody level as measured by microneutralization assay or similar method (secondary [Phase 1/2 part only] and exploratory endpoints).

[redacted]

Statistical Methods:

Hypothesis:

No formal hypotheses will be tested.

Sample Size:

The sample size for this study is not driven by statistical assumptions for formal hypothesis testing. The number of proposed participants is considered sufficient to provide a descriptive summary of the safety and immunogenicity of different dose levels of mRNA-1010.

For the Phase 1/2 part of the study, approximately 180 participants will be randomly assigned in this study, with approximately 45 participants in each vaccination group. With 45 participants receiving an investigational vaccine, there is a probability of approximately 90% to observe at least 1 participant with an AE, if the true incidence rate of the AE is 5%; if the true incidence rate is 10%, then the probability to observe it will be approximately 99%.

For the Phase 2 NH part of the study, approximately 500 participants will be randomly assigned in a 3:3:3:1 ratio to the mRNA-1010 25, 50, 100 µg groups, or the Active Comparator Group. A total of 450 participants will receive the mRNA-1010 vaccine (150 participants at each dose level). A sample size of 150 participants at each dose level has at least a 95% probability to observe at least 1 participant with an AE, if the true incidence rate is 2%; if the true incidence rate is 3%, then the probability to observe it will be 99%.

For the Phase 2 Extension part of the study, approximately 200 participants will be randomly assigned in a 1:1:1:1 ratio to the mRNA-1010 [redacted] groups, or the Active Comparator Group. A total of 150 participants will receive the mRNA-1010 vaccine (50 participants at each dose level). With 50 participants receiving an investigational vaccine, there is a probability of approximately 92% to observe at least 1 participant with an AE, if the true incidence rate of the AE is 5%; if the true incidence rate is 10%, then the probability to observe it will be approximately 99%.

Analysis Sets:

Set	Description
Randomization Set	The Randomization Set consists of all participants who are randomly assigned.

Full Analysis Set (FAS)[1]	The FAS consists of all randomly assigned participants who receive the IP.
Per-Protocol (PP) Set[2]	The PP Set consists of all participants in the FAS who comply with the injection schedule, comply with the timings of immunogenicity blood sampling to have a baseline and at least 1 postinjection assessment, do not have influenza infection at baseline through Day 29 (as documented by PCR testing), and have no major protocol deviations that impact the immune response.
Safety Set[3]	The Safety Set consists of all randomly assigned participants who receive the IP.
Solicited Safety Set[4]	The Solicited Safety Set consists of all participants in the Safety Set who contribute any solicited AR data.

Abbreviations: AR = adverse reaction; IP = investigational product; PCR = polymerase chain reaction; RT-PCR = reverse transcription polymerase chain reaction.

1 For the FAS, participants will be analyzed according to the group to which they were randomized.
2 The PP Set will be used as the primary analysis set for analyses of immunogenicity unless otherwise specified. Participants will be analyzed according to the group to which they were randomized.
3 The Safety Set will be used for all analyses of safety, except for the solicited ARs. Participants will be included in the vaccination group corresponding to what they actually received.
4 The Solicited Safety Set will be used for the analyses of solicited ARs and participants will be included in the vaccination group corresponding to what they actually received.

Safety Analyses:

All safety analyses will be based on the Safety Set, except summaries of solicited ARs, which will be based on the Solicited Safety Set. All safety analyses will be provided by vaccination group. Participants will be included in the vaccination group corresponding to what they actually received.

Safety and reactogenicity will be assessed by clinical review of all relevant parameters, including solicited ARs (local and systemic ARs), unsolicited AEs (including any clinical safety laboratory abnormalities [Phase 1/2 part only]), treatment-related AEs, severe AEs, SAEs, MAAEs, AEs leading to withdrawal from study participation, AESIs, vital sign measurements, and physical examination findings.

The number and percentage of participants with any solicited local AR, solicited systemic AR, during the 7-day follow-up period after the injection will be summarized. A 2-sided 95% exact confidence interval (CI) using the Clopper-Pearson method will also be provided for the percentage of participants with any solicited AR.

The number and percentage of participants with unsolicited AEs, treatment-related AEs, severe AEs, SAEs, AESIs, MAAEs, and AEs leading to withdrawal from study participation will be summarized. Unsolicited AEs will be coded according to the Medical Dictionary for Regulatory

Activities (MedDRA) for AR terminology and presented by MedDRA system organ class and preferred term.

Solicited ARs will be coded according to the MedDRA for AR terminology. The toxicity grading scale for healthy adult and adolescent volunteers enrolled in preventive vaccine clinical trials will be used in this study with modification for rash, solicited ARs, and vital signs. Unsolicited AEs will be presented by MedDRA system organ class and preferred term.

The number of events of unsolicited AEs/SAEs, AESIs, and MAAEs will be reported in summary tables accordingly. For all other safety parameters, descriptive summary statistics will be provided.

Assessment of safety laboratory tests will be done for the Phase 1/2 part of the study only. The number and percentage of participants who have chemistry and hematology results below or above the normal laboratory ranges will be tabulated by time point. For treatment-emergent safety laboratory test results, the raw values and change from baseline values will be summarized by vaccination group and visit at each time point.

For all parts of the study, vital sign results will also be presented.

Immunogenicity Analyses:

Immunogenicity analyses in each part of the study will be reported based on the Per-Protocol Set and provided by vaccination group, unless otherwise specified.

Phase 1/2

For the immunogenicity endpoints, geometric mean of specific antibody titers with corresponding 95% CI at each time point and geometric mean fold rise (GMFR) of specific antibody titers with corresponding 95% CI at each postbaseline time point over preinjection baseline at Day 1 will be provided by vaccination group. The 95% CIs will be calculated based on the t-distribution of the log 2-transformed values and then back transformed to the original scale. Descriptive summary statistics, including median, minimum, and maximum, will also be provided.

For the calculation of geometric mean titers (GMTs), antibody titers reported as below the lower limit of quantification (LLOQ) will be replaced by 0.5 × LLOQ. Values that are greater than the upper limit of quantification (ULOQ) will be converted to the ULOQ.

Seroconversion rate from baseline will be provided with a 2-sided 95% CI using the Clopper-Pearson method at each postbaseline time point. Rate of seroconversion is defined as the proportion of participants with either a prevaccination HAI titer < 1:10 and a postvaccination

HAI titer ≥ 1:40 or a prevaccination HAI titer ≥ 1:10 and a minimum 4-fold rise in the postvaccination HAI antibody titer.

Phase 2 NH and Phase 2 Extension

Immune responses, as measured by GMT and seroconversion rate in the mRNA-1010 groups based on Day 29 HAI titers, will be compared with that of participants receiving active comparator for all 4 strains.

An analysis of covariance model will be carried out with HAI titers at Day 29 as a dependent variable and a group variable as the fixed variable. The GMT of the mRNA-1010 at Day 29 will be estimated by the geometric least square mean (GLSM) from the model. The geometric mean ratio (GMTr; ratio of GMT) will be estimated by the ratio of GLSM from the model. The corresponding 2-sided 95% CI will be provided to assess the difference in immune response between the mRNA-1010 compared with the active comparator at Day 29.

The number and percentage of participants with seroconversion due to vaccination will be provided with 2-sided 95% CI using the Clopper-Pearson method at Day 29. The seroconversion rate difference with 95% CI at Day 29 will be provided.

In addition, the GMT of HAI titers with corresponding 95% CI will be provided at each time point. The 95% CIs will be calculated based on the t-distribution of the log 2-transformed values and then back transformed to the original scale. The GMFR of HAI titers with corresponding 95% CI at each postbaseline time point over baseline will be provided. Descriptive summary statistics, including median, minimum, and maximum, will also be provided.

For summarizations of GMTs, antibody titers reported as LLOQ will be replaced by 0.5 × LLOQ. Values that are greater than ULOQ will be converted to the ULOQ.

Rate of seroconversion is defined as (1) if LLOQ is 1:10, the proportion of participants with either a prevaccination HAI titer < 1:10 and a postvaccination HAI titer ≥ 1:40 or a prevaccination HAI titer ≥ 1:10 and a minimum 4-fold rise in the postvaccination HAI antibody titer; or (2) if LLOQ is greater than 1:10, the proportion of participants with either a prevaccination HAI titer < LLOQ and a postvaccination HAI ≥ 4 times of LLOQ, or a prevaccination HAI ≥ LLOQ and a minimum 4-fold rise in the postvaccination HAI .

The HAI titers at Day 29, GMTr at Day 29, and the difference in seroconversion rate at Day 29 may also be performed with the Full Analysis Set as supplementary analyses.

Phase 2 Extension

Immune responses, as measured by GMT and seroconversion rate in the mRNA-1010 groups based on Day 29 HAI titers, will be compared with that of participants receiving active comparator for all 4 strains.

The number and percentage of participants with seroconversion due to vaccination will be provided with 2-sided 95% CI using the Clopper-Pearson method at Day 29. The seroconversion rate difference with 95% CI at Day 29 will be provided.

For summarizations of GMTs, antibody titers reported as LLOQ will be replaced by 0.5 × LLOQ. Values that are greater than ULOQ will be converted to the ULOQ. The definition of rate of seroconversion will be the same definition as the rate of seroconversion in Phase 2 NH.

Study Analyses

A Day 29 IA is planned in this study for the Phase 1/2, Phase 2 NH, and Phase 2 Extension parts separately. The IA for the Phase 1/2 part will be performed after all participants have completed the Day 29 Visit. All relevant data to the IA will be cleaned (ie, data that are as clean as possible).

For the Phase 2 NH and the Phase 2 Extension parts, an IA of safety (reactogenicity and immunogenicity as applicable) will be performed after participants have completed the Day 29 Visit for each part of the study. All data relevant to the IA through the Day 29 Visit will be cleaned (ie, data that are as clean as possible). An interim study report may be generated, in which case a formal database lock will be performed to support the interim study report.

The IAs will be performed by a separate team of unblinded programmers and statisticians. The analyses will be presented by vaccination groups. Except for a limited number of Sponsor and CRO personnel who will be unblinded to perform the IA, the study site staff, Investigators, study monitors, and participants will remain blinded until after the final database lock for final analysis.

The final analysis of safety, reactogenicity, and immunogenicity will be performed after all participants have completed all planned study procedures. The results of this analysis will be presented in a final clinical study report, including individual listings.

1.2. Schema

Figure 1 Phase 1/2 - Study Schema

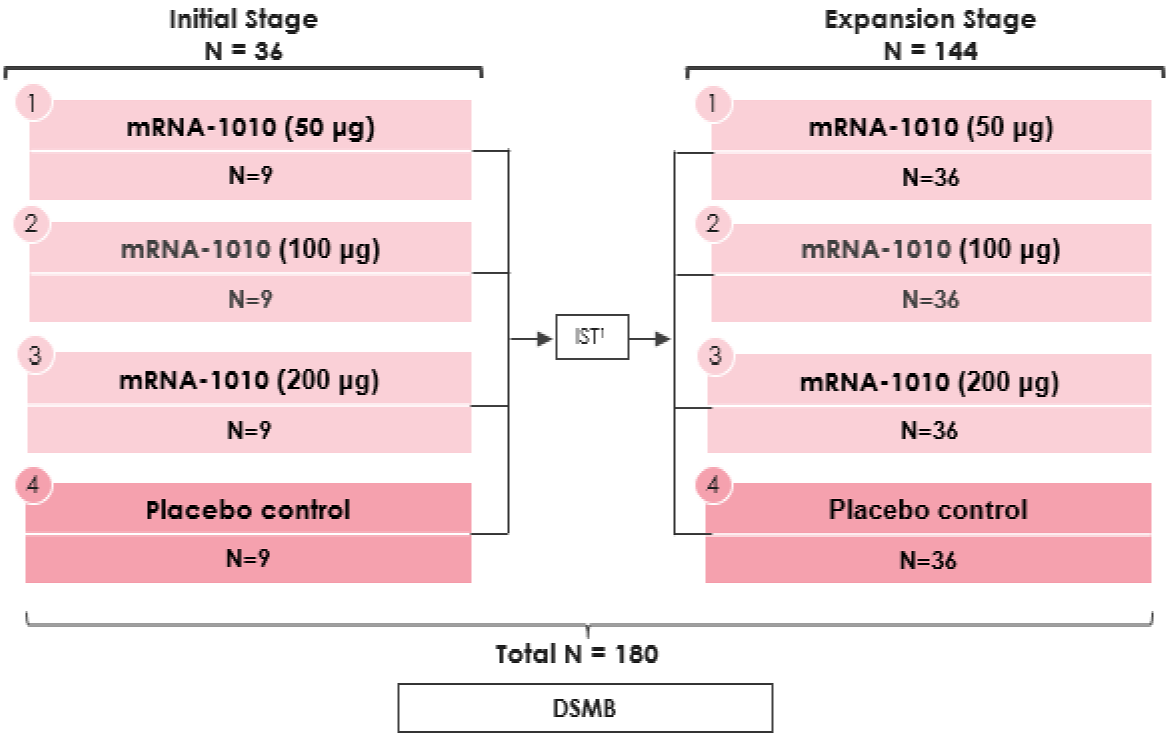

Abbreviations: DSMB = Data and Safety Monitoring Board; IST = internal safety team; N = number of participants.
[1] Expansion from the initial stage to the expansion stage is triggered by the IST.

Figure 2 Phase 2 Northern Hemisphere - Study Schema

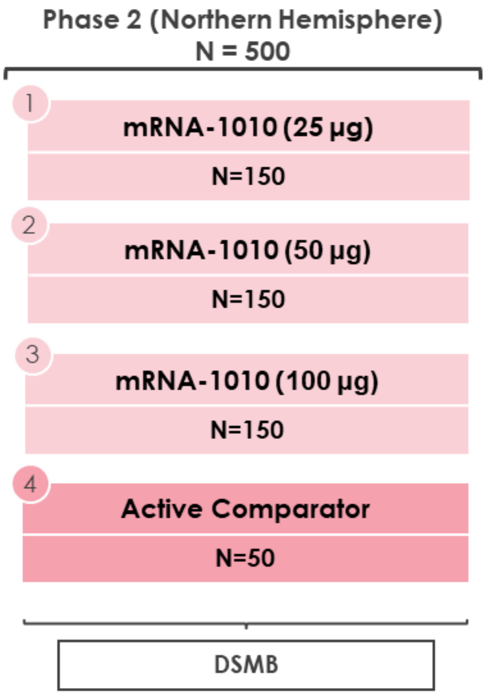

Abbreviations: DSMB = Data and Safety Monitoring Board; N = number of participants.

Figure 3 Phase 2 Extension - Study Schema

[redacted]

Abbreviations: DSMB = Data and Safety Monitoring Board; N = number of participants.

1.3. Schedule of Events

Table 1 Phase 1/2 - Schedule of Events

Visit Number	SCRN[1]	1	2	3	4	5, 6, and 7	8	Unscheduled
**Type of Visit**	**C**	**C**	**C**	**C**	**C**	**SC**	**C**	**C**
**Month Time Point**	**NA**	**M0**	**M0**	**M1**	**M2**	**M3-M5**	**M6**	**M0-M6**
**Study Visit Day**	**-**	**D1 (Baseline)**	**D8**	**D29**	**D57**	**D91, D121, D151**	**D181/EoS**	**NA**
**Window Allowance (Days)**	**-28**	**-**	**–2 or +1**	**±2**	**±5**	**±5**	**±14**	**NA**
ICF, demographics, concomitant medications, medical history	X	-	-	-	-	-	-	-
Inclusion/exclusion criteria	X	X	-	-	-	-	-	-
Blood collection for safety laboratory samples[2]	X	-	X	-	-	-	-	-
Physical examination[3]	X	-	-	-	-	-	-	-
Vital signs[4]	X	X	-	-	-	-	-	-
Pregnancy testing[5]	X	X	-	-	-	-	-	-
Randomization	-	X	-	-	-	-	-	-
Study vaccination (including 60-minute postdosing observation period)	-	X[6]	-	-	-	-	-	-
Blood collection for humoral immunogenicity[7]	-	X	X	X	-	-	X	-
[redacted]								
NP swab for virus detection[8]	-	X	-	-	-	-	-	X
eDiary activation for recording solicited ARs (7 days)[9]	-	X	-	-	-	-	-	-
Review of eDiary	-	-	X	-	-	-	-	-
Follow-up safety calls[10]	-	-	-	-	-	X	-	-
Recording of unsolicited AEs	-	X	X	X	-	-	-	-

**Visit Number**	**SCRN**[1]	**1**	**2**	**3**	**4**	**5, 6, and 7**	**8**	**Unscheduled**
**Type of Visit**	**C**	**C**	**C**	**C**	**C**	**SC**	**C**	**C**
**Month Time Point**	**NA**	**M0**	**M0**	**M1**	**M2**	**M3-M5**	**M6**	**M0-M6**
**Study Visit Day**	**-**	**D1 (Baseline)**	**D8**	**D29**	**D57**	**D91, D121, D151**	**D181/EoS**	**NA**
**Window Allowance (Days)**	**-28**	**-**	**–2 or +1**	**±2**	**±5**	**±5**	**±14**	**NA**
Recording of MAAEs and concomitant medications relevant to or for the treatment of the MAAE[11]	-	X	X	X	X	X	X	X
Recording of SAEs, AESIs, and concomitant medications relevant to or for the treatment of the SAE and/or AESI[11]	-	X	X	X	X	X	X	X
Recording of concomitant medications and nonstudy vaccinations[11]	X	X	X	X	X	X	X	X
Phase 1/2 completion	-	-	-	-	-	-	X	-

Abbreviations: AE = adverse event; AESI = adverse event of special interest; ALT = alanine aminotransferase; AR = adverse reaction; AST = aspartate aminotransferase; C = clinic visit (ie, study site visit); COVID-19 = coronavirus disease 2019; D = day; eCRF = electronic case report form; eDiary = electronic diary; EoS = end of study; FSH = follicle-stimulating hormone; ICF = informed consent form; ILI = influenza-like illness; IM = intramuscular; IP = investigational product; M = month; MAAE = medically attended adverse event; NA = not applicable; NP = nasopharyngeal; RT-PCR = reverse transcription polymerase chain reaction; SAE = serious adverse event; SARS-CoV-2 = severe acute respiratory syndrome coronavirus 2; SC = safety call (phone or contact by electronic means); SCRN = screening; WBC = white blood cell.

[1] The Screening Visit and Day 1 Visit cannot be completed on the same day. Additionally, the Screening Visit may be performed over multiple visits if within the 28-day screening window.

[2] Safety laboratory tests: WBC count, hemoglobin, platelets, ALT, AST, total bilirubin, alkaline phosphatase, and creatinine.

[3] Physical examination: A full physical examination, including height and weight, will be performed at screening. Symptom-directed physical examinations may be performed at other time points at the discretion of the Investigator. Any clinically significant finding identified during a study visit should be reported as an MAAE.

[4] Vital sign measurement: systolic and diastolic blood pressure, heart rate, respiratory rate, and body temperature. Participants will be seated for at least 5 minutes before all measurements are taken. Vital signs will be collected at the Screening Visit and on the day of vaccination, once before and at least 1 hour after the vaccination. Vital signs will be collected at other study site visits only in conjunction with a symptom-directed physical examination. The preferred route of temperature assessment is oral. Participants must not eat or drink anything hot or cold within 10 minutes before oral temperature is taken. If any of the vital sign measurements meet the toxicity grading criteria for clinical abnormalities of Grade 3 or greater, the abnormal value and grade will be documented in the AE section of the eCRF (unless there is another known cause of the abnormality that would result in an AE classification). The Investigator will continue to monitor the participant with additional assessments until the vital sign value has reached the reference range, returns to the vital sign value at baseline, is considered stable or until the Investigator determines that follow-up is no longer medically necessary.

[5] A point-of-care urine pregnancy test will be performed at the Screening Visit and before the vaccine dose on Day 1. At the discretion of the Investigator, a pregnancy test either via blood or point-of-care urine can be performed at any time. The FSH level may be measured at the Screening Visit as necessary and at the discretion of the Investigator to confirm postmenopausal status.

[6] A 50-µg dose of mRNA-1010 will be administered for Vaccination Group 1, a 100-µg dose of mRNA-1010 will be administered for Vaccination Group 2, a 200-µg dose of mRNA-1010 will be administered for Vaccination Group 3, and placebo will be administered for Vaccination Group 4. All participants will receive a single 0.5-mL IM injection of the IP.

[7] Samples for humoral and [redacted] immunogenicity (and safety laboratory samples) must be collected prior to receipt of vaccination on Day 1. [redacted]

[8] An NP swab specimen will be collected on Day 1 prior to vaccination to assess for asymptomatic infection with respiratory pathogens, including influenza virus and SARS-CoV-2. An NP swab will also be collected at any time during the study through study completion if any signs or symptoms or an MAAE suggesting COVID-19 or ILI occur, to confirm the diagnosis by RT-PCR. For signs or symptoms during the study, a participant will be instructed to contact the study site to have an NP swab collected for testing for respiratory pathogens at an unscheduled visit. The NP swabs may be collected as part of a home visit in lieu of a study site visit.

[9] The eDiary entries will be recorded by the participant at approximately 1 hour after vaccination while at the study site under the supervision of the study site staff. Participants will continue to record data in the eDiary for solicited AEs each day after they leave the study site, preferably in the evening and at the same time each day, on the day of injection, and for 6 days following injection. Any solicited AR that continues beyond Day 7 will be reported until it is no longer reported but only until 28 days after vaccination. Adverse reactions recorded in diaries beyond Day 7 should be reviewed either during the next scheduled telephone call or at the next study site visit.

[10] Trained study personnel will call all participants to collect information relating to any AEs, MAAEs, AEs leading to withdrawal from study participation, SAEs, AESIs, information on concomitant medications associated with those events, and any nonstudy vaccinations.

[11] All nonstudy vaccinations will be recorded through Day 181/EoS. All concomitant medications will be recorded through 28 days after vaccination; all concomitant medications relevant to or for the treatment of an SAE, AESI, or MAAE will be recorded from Day 1 through Day 181/EoS.

Table 2 Phase 2 Northern Hemisphere - Schedule of Events

Visit Number	SCRN[1]	1	2	3	4	5	6	Unscheduled
Type of Visit/Contact	C	C	SC	SC	C	C	C	C
Month Time Point	NA	M0	M0	M0	M1	M3	M6	M0-M6
Study Visit Day	-	D1 (Baseline)	D8	D15	D29	D91	D181/EoS	NA
Window Allowance (Days)	-28	-	±3	±3	-7 to +3	±5	±14	NA
ICF, demographics, concomitant medications, medical history[2]	X	-	-	-	-	-	-	-
Inclusion/exclusion criteria	X	X	-	-	-	-	-	-
Physical examination[3]	X	-	-	-	-	-	-	-
Vital signs[4]	X	X	-	-	-	-	-	-
Pregnancy testing[5]	X	X	-	-	-	-	-	-
Randomization	-	X	-	-	-	-	-	-
Study vaccination (including 60-minute postdosing observation period)	-	X[6]	-	-	-	-	-	-
Blood collection for humoral immunogenicity (and exploratory assessments)[7]	-	X	-	-	X	X	X	-
Optional blood collection for pharmacogenomics	-	X	-	-	-	-	-	-
NP swab for virus detection[8]	-	-	-	-	-	-	-	X
eDiary activation for recording solicited ARs (7 days)[9]	-	X	-	-	-	-	-	-
Review of eDiary for solicited ARs	-	-	X	-	-	-	-	-
eDiary activation for active and passive surveillance[10]	-	X	-	-	-	-	-	-
eDiary active surveillance collection of symptoms of ILI[11]	-	3 to 4 times weekly				Twice weekly		-

Visit Number	SCRN[1]	1	2	3	4	5	6	Unscheduled
**Type of Visit/Contact**	**C**	**C**	**SC**	**SC**	**C**	**C**	**C**	**C**
**Month Time Point**	**NA**	**M0**	**M0**	**M0**	**M1**	**M3**	**M6**	**M0-M6**
**Study Visit Day**	**-**	**D1 (Baseline)**	**D8**	**D15**	**D29**	**D91**	**D181/EoS**	**NA**
**Window Allowance (Days)**	**-28**	**-**	**±3**	**±3**	**-7 to +3**	**±5**	**±14**	**NA**
eDiary passive surveillance collection of symptoms of ILI[11]	-	X						-
Review of eDiary[12]	-	Review participant-recorded ILI starting on Day 1 through Day 181/EoS						
Follow-up safety call	-	-	X	X	-	-	-	-
Recording of unsolicited AEs	-	X	X	X	X	-	-	-
Recording of MAAEs and concomitant medications relevant to or for the treatment of the MAAE[13]	-	X	X	X	X	X	X	X
Recording of SAEs, AESIs, and concomitant medications relevant to or for the treatment of the SAE and/or AESI[13]	-	X	X	X	X	X	X	X
Recording of concomitant medications and nonstudy vaccinations[14]	X	X	X	X	X	X	X	X
Study completion	-	-	-	-	-	-	X[15]	-

Abbreviations: AE = adverse event; AESI = adverse event of special interest; AR = adverse reaction; C = clinic visit (ie, study site visit); COVID-19 = coronavirus disease 2019; D = day; eCRF = electronic case report; form; eDiary = electronic diary; EoS = end of study; FSH = follicle-stimulating hormone; ICF = informed consent form; ILI = influenza-like illness; IM = intramuscular; M = month; MAAE = medically attended adverse event; NA = not applicable; NP = nasopharyngeal swab; SAE = serious adverse event; SARS-CoV-2 = severe acute respiratory syndrome coronavirus 2; SC = safety call (phone or contact by electronic means); SCRN = screening.

[1] The Screening Visit and Day 1 Visit may be performed on the same day or a different day. Additionally, the Screening Visit may be performed over multiple visits if within the 28-day screening window.

[2] Verbal confirmation of medical history is acceptable.

[3] Physical examination: A full physical examination, including height and weight, will be performed at screening; symptom-directed physical examinations will be performed at all other clinic visits (ie, study site visits). Interim physical examinations will be performed at the discretion of the Investigator. Any clinically significant finding identified by a healthcare professional during study visits should be reported as an MAAE.

[4] Vital sign measurement: Systolic and diastolic blood pressure, heart rate, respiratory rate, and body temperature. The preferred route of temperature assessment is oral. Participants must not eat or drink anything hot or cold within 10 minutes before oral temperature is taken. Participants will be seated for at least 5 minutes before all measurements are taken. Vital signs will be collected on the day of vaccination, once before vaccination and approximately 1 hour after vaccination. Vital signs may be collected at other study site visits in conjunction with a symptom-directed physical examination. If any of the vital sign measurements meet the toxicity grading criteria for clinical

abnormalities of Grade 3 or greater, the abnormal value and grade will be documented in the AE section of the eCRF (unless there is another known cause of the abnormality that would result in an AE classification). The Investigator will continue to monitor the participant with additional assessments until the vital sign value has reached the reference range, returns to the vital sign value at baseline, is considered stable or until the Investigator determines that follow-up is no longer medically necessary.

[5] A point-of-care urine pregnancy test will be performed at the Screening Visit (and before the vaccine dose on Day 1 if Day 1 is not on the same day as the Screening Visit). At the discretion of the Investigator, a pregnancy test either via blood or point-of-care urine can be performed at any time. The FSH level may be measured at the Screening Visit as necessary and at the discretion of the Investigator, to confirm postmenopausal status.

[6] See Table 8 for dose levels and vaccination groups. All participants will be randomized to receive a single 0.5-mL IM injection.

[7] Samples for humoral immunogenicity (and exploratory assessments) must be collected prior to receipt of vaccination on Day 1. A subset of samples collected will be assessed for humoral immunogenicity (and exploratory assessments).

[8] An NP swab specimen for pathogens, including influenza virus and SARS-CoV-2 will be collected any time from Day 1 through Day 181/EoS if participants have protocol-defined ILI or symptoms suggestive of COVID-19 or other upper or lower respiratory infection at the Investigator's discretion. If participants experience these symptoms/signs, they will be instructed to contact the study site to have an NP swab collected for testing. The NP swabs may be collected as part of a home visit in lieu of a study site visit.

[9] The eDiary entries will be recorded by all participants at approximately 1 hour after vaccination while at the study site under the supervision of the study site staff. Participants will continue to record data in the eDiary for solicited ARs each day after they leave the study site, preferably in the evening and at the same time each day, on the day of vaccination, and the 6 days following vaccination. Any solicited AR that is ongoing beyond Day 7 will be reported until it is no longer reported but only until 28 days after vaccination. Adverse reactions recorded in the eDiary beyond Day 7 should be reviewed either during the next scheduled call or study site visit, or during an unscheduled visit.

[10] The eDiary will be activated for collection of ILI symptoms.

[11] Passive Surveillance: Participants will be instructed to report symptoms of ILI any time from Day 1 through Day 181/EoS; Active Surveillance: Participants will be instructed to report whether ILI symptoms have been experienced, 3 to 4 times weekly from Day 1 through Day 29 and twice weekly from Day 30 through Day 181/EoS, via eDiary or telephone calls. If symptoms occur, participants will be directed to return to the study site as soon as possible, but no later than 72 hours after the onset of symptoms for medical evaluation and an NP swab.

[12] Review of eDiary for recording of symptoms of ILI.

[13] Trained study personnel will call all participants to collect information relating to any MAAEs, AEs leading to study discontinuation, SAEs, AESIs, information on concomitant medications associated with those events, and any nonstudy vaccinations.

[14] All nonstudy vaccinations will be recorded through Day 181/EoS. All concomitant medications will be recorded through 28 days after vaccination; all concomitant medications relevant to or for the treatment of an SAE, AESI, or MAAE will be recorded from Day 1 through Day 181/EoS.

[15] Participants who develop ILI will be followed through 30 days from the onset of ILI even if Day 30 is beyond Day 181/EoS.

Table 3 Phase 2 Extension - Schedule of Events

Visit Number	SCRN[1]	1	2	3	4	5	6	7	Unscheduled
**Type of Visit/Contact**	**C**	**C**	**C**	**SC**	**SC**	**C**	**C**	**C**	**C**
**Month Time Point**	**NA**	**M0**	**M0**	**M0**	**M0**	**M1**	**M3**	**M6**	**M0-M6**
**Study Visit Day**	**-**	**D1 (Baseline)**	**D4**	**D8**	**D15**	**D29**	**D91**	**D181/EoS**	**NA**
**Window Allowance (Days)**	**-28**	**-**	**-2**	**±3**	**±3**	**-7 to +3**	**±5**	**±14**	**NA**
ICF, demographics, concomitant medications, medical history[2]	X	-	-	-	-	-	-	-	-
Inclusion/exclusion criteria	X	X	-	-	-	-	-	-	-
Physical examination[3]	X	-	-	-	-	-	-	-	-
Vital signs[4]	X	X	-	-	-	-	-	-	-
Pregnancy testing[5]	X	X	-	-	-	-	-	-	-
Randomization	-	X	-	-	-	-	-	-	-
Study vaccination (including 60-minute postdosing observation period)	-	X[6]	-	-	-	-	-	-	-
Blood collection for humoral immunogenicity (and exploratory assessments)[7]	-	X	-	-	-	X	X	X	-
Optional blood collection for pharmacogenomics	-	X	-	-	-	-	-	-	-
Optional blood collection for potential biomarker analysis[8]	-	-	X	-	-	-	-	-	-
NP swab for virus detection[9]	-	-	-	-	-	-	-	-	X
eDiary activation for recording solicited ARs (7 days)[10]	-	X	-	-	-	-	-	-	-
Review of eDiary for solicited ARs	-	-	-	X	-	-	-	-	-
Follow-up safety call	-	-	-	X	X	-	-	-	-
Recording of unsolicited AEs	-	X	-	X	X	X	-	-	-

**Visit Number**	**SCRN[1]**	**1**	**2**	**3**	**4**	**5**	**6**	**7**	**Unscheduled**
**Type of Visit/Contact**	**C**	**C**	**C**	**SC**	**SC**	**C**	**C**	**C**	**C**
**Month Time Point**	**NA**	**M0**	**M0**	**M0**	**M0**	**M1**	**M3**	**M6**	**M0-M6**
**Study Visit Day**	**-**	**D1 (Baseline)**	**D4**	**D8**	**D15**	**D29**	**D91**	**D181/EoS**	**NA**
**Window Allowance (Days)**	**-28**	**-**	**-2**	**±3**	**±3**	**-7 to +3**	**±5**	**±14**	**NA**
Recording of MAAEs and concomitant medications relevant to or for the treatment of the MAAE[11]	-	X	-	X	X	X	X	X	X
Recording of SAEs, AESIs, and concomitant medications relevant to or for the treatment of the SAE and/or AESI[11]	-	X	-	X	X	X	X	X	X
Recording of concomitant medications and nonstudy vaccinations[12]	X	X	-	X	X	X	X	X	X
Study completion	-	-	-	-	-	-	-	X[13]	-

Abbreviations: AE = adverse event; AESI = adverse event of special interest; AR = adverse reaction; C = clinic visit (ie, study site visit); COVID-19 = coronavirus disease 2019; D = day; eCRF = electronic case report; form; eDiary = electronic diary; EoS = end of study; FSH = follicle-stimulating hormone; ICF = informed consent form; ILI = influenza-like illness; IM = intramuscular; M = month; MAAE = medically attended adverse event; NA = not applicable; NP = nasopharyngeal swab; SAE = serious adverse event; SARS-CoV-2 = severe acute respiratory syndrome coronavirus 2; SC = safety call (phone or contact by electronic means); SCRN = screening.

1 The Screening Visit and Day 1 Visit may be performed on the same day or a different day. Additionally, the Screening Visit may be performed over multiple visits if within the 28-day screening window.

2 Verbal confirmation of medical history is acceptable.

3 Physical examination: A full physical examination, including height and weight, will be performed at screening; symptom-directed physical examinations will be performed at all other clinic visits (ie, study site visits). Interim physical examinations will be performed at the discretion of the Investigator. Any clinically significant finding identified by a healthcare professional during study visits should be reported as an MAAE.

4 Vital sign measurement: Systolic and diastolic blood pressure, heart rate, respiratory rate, and body temperature. The preferred route of temperature assessment is oral. Participants must not eat or drink anything hot or cold within 10 minutes before oral temperature is taken. Participants will be seated for at least 5 minutes before all measurements are taken. Vital signs will be collected on the day of vaccination, once before vaccination and approximately 1 hour after vaccination. Vital signs may be collected at other study site visits in conjunction with a symptom-directed physical examination. If any of the vital sign measurements meet the toxicity grading criteria for clinical abnormalities of Grade 3 or greater, the abnormal value and grade will be documented in the AE section of the eCRF (unless there is another known cause of the abnormality that would result in an AE classification). The Investigator will continue to monitor the participant with additional assessments until the vital sign value has reached the reference range, returns to the vital sign value at baseline, is considered stable or until the Investigator determines that follow-up is no longer medically necessary.

5 A point-of-care urine pregnancy test will be performed at the Screening Visit (and before the vaccine dose on Day 1 if Day 1 is not on the same day as the Screening Visit). At the discretion of the Investigator, a pregnancy test either via blood or point-of-care urine can be performed at any time. The FSH level may be measured at the Screening Visit as necessary and at the discretion of the Investigator, to confirm postmenopausal status.

6 See Table 9 for dose levels and vaccination groups. All participants will be randomized to receive a single 0.5-mL IM injection.

[7] Samples for humoral immunogenicity (and exploratory assessments) must be collected prior to receipt of vaccination on Day 1. A subset of samples collected will be assessed for humoral immunogenicity (and exploratory assessments).
[8] Biomarker plasma and biomarker serum samples will be stored for potential future biomarker assessment.
[9] An NP swab specimen for pathogens, including influenza virus and SARS-CoV-2 will be collected any time from Day 1 through Day 181/EoS if participants experience symptoms consistent with the US CDC definition of ILI and/or symptoms suggestive of COVID-19 or other upper or lower respiratory infection at the Investigator's discretion. If participants experience these symptoms/signs, they will be instructed to contact the study site to have an NP swab collected for testing. The NP swabs may be collected as part of a home visit in lieu of a study site visit. In the event that NP swabs during ILI cannot be collected, any available influenza and/or SARS-CoV-2 testing results performed outside of the study should be captured in the eCRF.
[10] The eDiary entries will be recorded by all participants at approximately 1 hour after vaccination while at the study site under the supervision of the study site staff. Participants will continue to record data in the eDiary for solicited ARs each day after they leave the study site, preferably in the evening and at the same time each day, on the day of vaccination, and the 6 days following vaccination. Any solicited AR that is ongoing beyond Day 7 will be reported until it is no longer reported but only until 28 days after vaccination. Adverse reactions recorded in the eDiary beyond Day 7 should be reviewed either during the next scheduled call or study site visit, or during an unscheduled visit.
[11] Trained study personnel will call all participants to collect information relating to any MAAEs, AEs leading to study discontinuation, SAEs, AESIs, information on concomitant medications associated with those events, and any nonstudy vaccinations.
[12] All nonstudy vaccinations will be recorded through Day 181/EoS. All concomitant medications will be recorded through 28 days after vaccination; all concomitant medications relevant to or for the treatment of an SAE, AESI, or MAAE will be recorded from Day 1 through Day 181/EoS.
[13] Participants who develop ILI will be followed through 30 days from the onset of ILI even if Day 30 is beyond Day 181/EoS.

TABLE OF CONTENTS

CLINICAL STUDY PROTOCOL 1
PROTOCOL APPROVAL – SPONSOR SIGNATORY 2
DECLARATION OF INVESTIGATOR 3
PROTOCOL AMENDMENT SUMMARY OF CHANGES 4
1. PROTOCOL SUMMARY 7
1.1. Synopsis 7
1.2. Schema 31
1.3. Schedule of Events 33
TABLE OF CONTENTS 42
LIST OF TABLES 46
LIST OF FIGURES 47
LIST OF ABBREVIATIONS AND DEFINITION OF TERMS 48
2. INTRODUCTION 51
2.1. Study Rationale 51
2.2. Background and Overview 51
2.2.1. mRNA-1010 51
2.2.2. Nonclinical Studies 52
2.2.3. Clinical Studies 53
2.3. Benefit/Risk Assessment 53
2.3.1. Known Potential Benefits 53
2.3.2. Risks From Study Participation and Their Mitigation 54
2.3.3. Overall Benefit/Risk Conclusion 55
3. OBJECTIVES AND ENDPOINTS 56
3.1. Phase 1/2 56
3.2. Phase 2 Northern Hemisphere and Phase 2 Extension 57
4. STUDY DESIGN 60
4.1. General Design 60
4.2. Scientific Rationale for Study Design 66
4.3. Choice of Vaccine Dose 67
4.4. End of Study Definition 68

5. STUDY POPULATION 69
5.1. Phase 1/2 69
5.1.1. Inclusion Criteria 69
5.1.2. Exclusion Criteria 70
5.2. Phase 2 Northern Hemisphere and Phase 2 Extension 72
5.2.1. Inclusion Criteria 72
5.2.2. Phase 2 Northern Hemisphere Exclusion Criteria 73
5.2.3. Phase 2 Extension Exclusion Criteria 74
5.3. Lifestyle Restrictions 75
5.4. Screen Failures 76
6. STUDY TREATMENT 77
6.1. Investigational Products Administered 77
6.2. Randomization 79
6.2.1. Phase 1/2 79
6.2.2. Phase 2 Northern Hemisphere 79
6.2.3. Phase 2 Extension 79
6.3. Preparation/Handling/Storage/Accountability 79
6.3.1. Preparation of Investigational Product 79
6.3.2. Investigational Product Administration 80
6.3.3. Investigational Product Delivery and Receipt 80
6.3.4. Investigational Product Packaging and Labeling 81
6.3.5. Investigational Product Storage 81
6.3.6. Investigational Product Accountability 82
6.3.7. Investigational Product Handling and Disposal 82
6.4. Investigational Product Compliance 82
6.5. Prior and Concomitant Medications 82
6.5.1. Prior Medications and Therapies 82
6.5.2. Concomitant Medications and Therapies 83
6.5.3. Concomitant Medications and Vaccines That May Lead to the Elimination of a Participant From Per-Protocol Analyses 84
6.6. Intervention After End of the Study 84
7. DELAY OR DISCONTINUATION OF STUDY INTERVENTION AND PARTICIPANT DISCONTINUATION/WITHDRAWAL 85

7.1. Pause Rules 85
7.1.1. Pause Rules for the Phase 1/2 Part of the Study 85
7.1.2. Pause Rules for the Phase 2 Northern Hemisphere and the Phase 2 Extension Parts of the Study 86
7.2. Criteria for Delay or Withholding of Study Vaccination 87
7.3. Participant Discontinuation/Withdrawal From the Study 88
7.4. Lost to Follow-Up 89
8. STUDY ASSESSMENTS AND PROCEDURES 90
8.1. Safety Assessments and Procedures 91
8.1.1. Use of Electronic Diaries 91
8.1.2. Safety Telephone Calls 93
8.1.3. Safety Laboratory Assessments 93
8.1.4. Vital Sign Measurements 94
8.1.5. Physical Examinations 94
8.1.6. Assessments for Respiratory Viral Infections 95
8.2. Immunogenicity Assessments 96
8.3. Efficacy Assessments 97
8.4. Safety Definitions and Related Procedures 97
8.4.1. Adverse Event 97
8.4.2. Serious Adverse Events 98
8.4.3. Solicited Adverse Reactions 99
8.4.4. Medically Attended Adverse Events 101
8.4.5. Anaphylaxis 102
8.4.6. Influenza-Like Illness 103
8.4.7. Adverse Events of Special Interest 103
8.4.8. Recording and Follow-Up of Pregnancy 104
8.4.9. Eliciting and Documenting Adverse Events 104
8.4.10. Assessment of Intensity 105
8.4.11. Assessment of Causality 105
8.4.12. Reporting Adverse Events 106
8.4.13. Reporting Serious Adverse Events 106
8.4.14. Reporting of Adverse Events of Special Interest 107

8.4.15. Time Period and Frequency for Collecting Adverse Event and Serious Adverse Event Information ... 107
8.4.16. Method of Detecting Adverse Events and Serious Adverse Events ... 108
8.4.17. Follow-Up of Adverse Events and Serious Adverse Events ... 109
8.4.18. Regulatory Reporting Requirements for Serious Adverse Events ... 109
8.5. Safety Monitoring ... 109
8.6. Treatment of Overdose ... 109
8.7. Pharmacokinetics ... 109
8.8. Pharmacodynamics ... 109
8.9. Biomarkers ... 110
8.10. Health Economics ... 110
9. STATISTICAL ANALYSIS PLAN ... 111
9.1. Blinding and Responsibility for Analyses ... 111
9.1.1. Breaking the Blind ... 112
9.2. Statistical Hypotheses ... 112
9.3. Sample Size Determination ... 112
9.4. Analysis Sets ... 114
9.5. Statistical Methods ... 114
9.5.1. Baseline Characteristics and Demographics ... 114
9.5.2. Safety Analyses ... 114
9.5.3. Immunogenicity Analyses ... 116
9.5.4. Exploratory Analyses ... 118
9.5.5. Subgroup Analyses ... 118
9.6. Study Analyses ... 118
10. REFERENCES ... 119
11. SUPPORTING DOCUMENTATION AND OPERATIONAL CONSIDERATIONS ... 123
11.1. APPENDIX 1: Study Governance Considerations ... 124
11.1.1. Regulatory and Ethical Considerations ... 124
11.1.2. Study Monitoring ... 124
11.1.3. Audits and Inspections ... 125
11.1.4. Financial Disclosure ... 126
11.1.5. Recruitment Procedures ... 126

11.1.6. Informed Consent Process 126
11.1.7. Protocol Amendments 127
11.1.8. Protocol Deviations 128
11.1.9. Data Protection 128
11.1.10. Sample Retention and Future Biomedical Research 129
11.1.11. Safety Oversight 129
11.1.12. Dissemination of Clinical Study Data 130
11.1.13. Data Quality Assurance and Quality Control 130
11.1.14. Data Collection and Management 131
11.1.15. Source Documents 132
11.1.16. Retention of Records 132
11.1.17. Study and Site Closure 133
11.1.18. Publication Policy 133
11.2. APPENDIX 2: Contraceptive Guidance 134
11.3. APPENDIX 3: Adverse Events of Special Interest Terms 136
11.4. APPENDIX 4: CDC Working Case Definition of Pericarditis, Myocarditis, and Myopericarditis Occurring After Receipt of COVID-19 mRNA Vaccines 137
11.5. APPENDIX 5: Protocol Amendment History 139

LIST OF TABLES

Table 1 Phase 1/2 - Schedule of Events 33
Table 2 Phase 2 Northern Hemisphere - Schedule of Events 36
Table 3 Phase 2 Extension - Schedule of Events 39
Table 4 Phase 1/2 - Study Objectives and Endpoints 56
Table 5 Phase 2 Northern Hemisphere and Phase 2 Extension - Study Objectives and Endpoints 58
Table 6 Vaccination Groups and Dose Levels 61
Table 7 Phase 1/2 - Summary of Vaccination Groups 78
Table 8 Phase 2 NH - Summary of Vaccination Groups 78
Table 9 Phase 2 Extension - Summary of Vaccination Groups 78
Table 10 Phase 1/2 Pause Rule Criteria, Events, and Thresholds - Single Event 85

Table 11 Phase 1/2 Pause Rule Criteria, Events, and Thresholds – Proportion of Participants 86
Table 12 Phase 2 Northern Hemisphere and Phase 2 Extension Pause Rule Criteria, Events, and Thresholds - Single Event 87
Table 13 Solicited Adverse Reactions and Grades 100
Table 14 Analysis Sets 114
Table 15 Analysis Strategy for Safety Parameters 116
Table 16 Adverse Events of Special Interest 136
Table 17 Case Definitions of Probable and Confirmed Myocarditis, Pericarditis, and Myopericarditis 137

LIST OF FIGURES

Figure 1 Phase 1/2 - Study Schema....31
Figure 2 Phase 2 Northern Hemisphere - Study Schema 32
Figure 3 Phase 2 Extension - Study Schema....32

LIST OF ABBREVIATIONS AND DEFINITION OF TERMS

The following abbreviations and terms are used in this study protocol.

Abbreviation or Specialist Term	Definition
AE	adverse event
AESI	adverse event of special interest
ALT	alanine aminotransferase
AR	adverse reaction
AST	aspartate aminotransferase
BMI	body mass index
BP	blood pressure
CD	cluster of differentiation
CDC	United States Centers for Disease Control and Prevention
CFR	Code of Federal Regulations
CI	confidence interval
COVID-19	coronavirus disease 2019
CRO	contract research organization
CSR	clinical study report
DSMB	Data and Safety Monitoring Board
[redacted]	[redacted]
eCRF	electronic case report form
EDC	electronic data capture
eDiary	electronic diary
EoS	end of study
FDA	Food and Drug Administration
FIH	first in human
FSH	follicle-stimulating hormone
GCP	Good Clinical Practice
GLSM	geometric least square mean
GMFR	geometric mean fold rise

Abbreviation or Specialist Term	Definition
GMT	geometric mean titer
GMTr	geometric mean titer ratio
HA	hemagglutinin
HAI	hemagglutination inhibition
HCP	healthcare practitioner
HIV	human immunodeficiency virus
HRT	hormone replacement therapy
IA	interim analysis
IB	investigator’s brochure
ICF	informed consent form
ICH	International Council for Harmonisation
IgG	immunoglobulin G
ILI	influenza-like illness
IM	intramuscular
IND	investigational new drug
IP	investigational product
IRB	institutional review board
IRT	interactive response technology
IST	internal safety team
LLOQ	lower limit of quantification
LNP	lipid nanoparticle
LTFU	lost to follow-up
MAAE	medically attended adverse event
MedDRA	Medical Dictionary for Regulatory Activities
mRNA	messenger RNA
NH	Northern Hemisphere
NP	nasopharyngeal

Abbreviation or Specialist Term	Definition
[redacted]	[redacted]
PP	per protocol
QA	quality assurance
RT-PCR	reverse transcription polymerase chain reaction
SAE	serious adverse event
SAP	statistical analysis plan
SH	Southern Hemisphere
SARS-CoV-2	severe acute respiratory syndrome coronavirus 2
[redacted]	[redacted]
SoE	schedule of events
ULOQ	upper limit of quantification
US	United States
USP	United States Pharmacopeia
WBC	white blood cell
WHO	World Health Organization

2. INTRODUCTION

2.1. Study Rationale

Seasonal influenza viruses are estimated by the World Health Organization (WHO) to cause 3 to 5 million cases of severe illness and up to 650,000 deaths each year, which is a severe challenge to public health (WHO 2018). Influenza epidemics occur each year and follow a seasonal circulation pattern with increased cases during the winter months in the Northern Hemisphere (NH) and Southern Hemisphere (SH) (Riedel et al 2019). Since influenza viruses continuously change through a process termed antigenic drift, the circulating viruses are actively monitored by a worldwide monitoring network coordinated by the WHO (Monto 2018). An expert panel recommends certain influenza virus strains for vaccine manufacturing, based on observed circulation patterns and antigenic changes, twice a year (once for NH and once for SH). Influenza A and B viruses are the most relevant influenza viruses for human infection. Therefore, current vaccine recommendations include 1 influenza A H1N1 strain, 1 influenza A H3N2 strain, and 2 influenza B strains (covering the B/Victoria and B/Yamagata lineages).

Currently licensed seasonal influenza virus vaccines rarely exceed 60% overall effectiveness and are less effective during the years when the circulating viruses do not match the strains selected for the vaccine antigens (CDC 2020a). Influenza vaccines based on messenger RNA (mRNA) technology could provide several benefits compared with current vaccines, including the ability to respond to strain changes more quickly; avoidance of mutations that may be acquired during vaccine production in eggs or cell culture; stronger immune responses; and improved protection in older adults (Rockman et al 2020).

2.2. Background and Overview

ModernaTX, Inc. (Sponsor) has developed a rapid response proprietary vaccine platform based on the mRNA delivery system. The platform is based on the principle and observations that cells in vivo can take up mRNA, translate it, and then express protein viral antigen(s) on the cell surface. The delivered mRNA does not enter the cellular nucleus or interact with the genome, is nonreplicating, and is expressed transiently.

Two mRNA vaccines against the severe acute respiratory syndrome coronavirus 2 (SARS-CoV-2), including Pfizer Inc.'s BNT162b2 (Polack et al 2020) and the Sponsor's mRNA-1273 (NCT04283461, NCT04405076, and NCT04470427) have been issued emergency use authorizations by the United States (US) Food and Drug Administration (FDA).

2.2.1. mRNA-1010

The Sponsor is using its mRNA-based platform to develop a novel lipid nanoparticle (LNP)-encapsulated mRNA-based vaccine against diseases caused by influenza virus types A

and B. The proposed development candidate mRNA-1010 is a quadrivalent vaccine containing mRNAs that encode for the hemagglutinins (HAs) of the 4 strains recommended by the WHO for cell- or recombinant-based vaccines. Equal amounts of mRNAs that encode for membrane-bound wild-type versions of each of the 4 different strains will be used for the HA components. The mRNA-1010 development candidate will be administered as a single intramuscular (IM) injection and aims to elicit protection from all seasonal influenza viruses covered by the vaccine.

The Sponsor is conducting this first-in-human (FIH) Phase 1/2 study of mRNA-1010, a seasonal influenza vaccine, to establish preliminary safety, reactogenicity, and immunogenicity data to support the initiation of a Phase 3 program for this vaccine. The design of this study will include an immunogenicity objective for the HA component, using HA inhibition (HAI) as a correlate of protection against influenza illness. The rationale for this approach is based on the established precedent of using HA-based immunologic correlates for clinical assessment and licensure of influenza vaccines (DHHS 2007a; Dunning et al 2016; EMA 2016). In this FIH study, the safety, reactogenicity, and immunogenicity of mRNA-1010 vaccine will be compared with placebo (Phase 1/2 part) and with an active comparator (Phase 2 NH and Phase 2 Extension parts). The vaccine administration in the Phase 1/2 part and the Phase 2 Extension part of the study are planned outside the NH influenza season. In the Phase 2 NH part, the vaccine administration is planned during the NH influenza season.

2.2.2. Nonclinical Studies

Using individually formulated HA-encoding mRNAs (preclinical material), 2 mouse immunogenicity studies were performed. The HA sequences used for preclinical formulation were from wild-type HAs.

Both studies used a prime/boost regimen with a 3-week interval. Immunized mice were bled on Day 21 (3 weeks after the prime dose) and Day 36 (2 weeks after the boost dose), and immunoglobulin G (IgG) antibody titers were determined with an enzyme-linked immunosorbent assay using recombinant HA proteins.

The first study compared mice immunized with individual mRNAs or a combination of all 4 HA mRNAs at 2 different doses (2 or 0.4 µg of each mRNA), based on the following strains recommended for the 2020-2021 NH influenza season:

- A/Hawaii/70/2019(H1N1)pdm09
- A/Hong Kong/45/2019(H3N2)
- B/Washington/02/2019 (B/Victoria lineage)
- B/Phuket/3073/2013 (B/Yamagata lineage)

High IgG antibody titers were observed against all HAs following immunization, with both individual mRNA constructs and mRNA combinations after a single dose. A dose response and booster effect were observed for most mRNA constructs (individual and combinations).

The second mouse immunogenicity study aimed to confirm that the 2021 SH composition is similarly immunogenic with the 2020-2021 NH composition. The compositions differed only for the H1N1 strain; the SH composition included A/Wisconsin/588/2019(H1N1)pdm09 instead of the A/Hawaii/70/2019(H1N1)pdm09 strain.

No difference in immunogenicity (HA IgG antibody titers) based on the strain/mRNA construct used for immunization (A/Hawaii/70/2019 in the NH composition versus A/Wisconsin/588/2019 in the SH composition) was observed, suggesting that the mRNA platform will support annual strain updates.

Additional studies to assess protection of mice from challenge with mouse-adapted H1N1 and H3N2 viruses after a single immunization and a study to assess immunogenicity and protection from H1N1 challenge after 2 immunizations in ferrets are currently ongoing. Results will be available in the planned investigational new drug (IND) application.

A detailed review of the nonclinical experience with mRNA-1010 vaccine will be provided in the investigator's brochure (IB).

2.2.3. Clinical Studies

No clinical studies with mRNA-1010 have been performed to date.

2.3. Benefit/Risk Assessment

2.3.1. Known Potential Benefits

The following benefits may accrue to participants who receive the mRNA-1010 vaccine:

- The mRNA-1010 vaccine may be effective against seasonal influenza strains as defined by the WHO for the 2020-2021 SH flu season (Phase 1/2) or against the seasonal influenza strains as defined by the WHO for the 2021-2022 NH flu season (Phase 2 NH and Phase 2 Extension).
- Participants will have a baseline (Day 1) evaluation for respiratory pathogens (Phase 1/2 part), including influenza virus and SARS-CoV-2, and ongoing surveillance for influenza-like illness (ILI) and/or coronavirus disease 2019 (COVID-19) throughout the study (Phase 1/2, Phase 2 NH, and Phase 2 Extension).
- The study will contribute to the development of a potentially efficacious vaccine against seasonal influenza.

2.3.2. Risks From Study Participation and Their Mitigation

Adverse events (AEs) ranging from immediate mild allergic reactions (eg, urticaria) to systemic allergic reactions (eg, anaphylaxis) may occur following any vaccination. Systemic allergic reactions are very rare and are estimated to occur once per 450,000 vaccinations for vaccines that do not contain allergens such as gelatin or egg protein (Zent et al 2002). Since the authorization of the mRNA-1273 vaccine for COVID-19, the US Centers for Disease Control and Prevention (CDC) estimate of the rate of anaphylaxis based on reporting in the Vaccine Adverse Event Reporting System is approximately 2.5 cases/million doses administered (Shimabukuro et al 2021). As a precaution, all participants will remain under observation at the study site for at least 60 minutes after injection.

Vasovagal syncope (fainting) can occur before or after any vaccination, is usually triggered by pain or anxiety associated with the injection and is not related to the substance injected. Therefore, it is important that standard precautions and procedures are followed to avoid injury from fainting.

Intramuscular injection with other mRNA vaccines manufactured by the Sponsor containing the proprietary [redacted] lipid formulation have commonly resulted in transient and self-limiting local inflammatory reactions. These typically included pain, erythema (redness), or swelling (hardness) at the injection site, which were mostly mild to moderate in severity and usually occurred within 24 hours of injection. Laboratory abnormalities (including increases in liver function tests and serum lipase levels) following injection have been observed in early phase clinical studies with similar mRNA-based vaccines. These abnormalities were without clinical symptoms or signs and returned toward baseline (Day 1) values over time.

In a recently completed Phase 3 study of mRNA-1273 vaccine for COVID-19 in 30,420 healthy adults, the most commonly reported local reactions included pain, swelling, and erythema at the injection site. Most of these reactions were Grade 1 or 2 in severity and resolved within 3 to 4 days of onset. The most commonly reported systemic reactions were headache, myalgia, arthralgia, fatigue, chills, and fever. In most cases, the reactions resolved spontaneously within several days (Baden et al 2021).

Similarly, safety results from Phase 1 studies conducted by the Sponsor on 2 mRNA vaccines containing the HA glycoproteins from the H10N8 and H7N9 avian influenza viruses were well tolerated, although the Sponsor's LNP/mRNA platform has since been updated (eg, [redacted] is used in mRNA-1010 and mRNA-1273; Feldman et al 2019).

There have been very rare reports of myocarditis and pericarditis occurring after vaccination with COVID-19 mRNA vaccines. The majority of the cases have been reported in young males shortly after the second dose of the vaccine. These are typically mild cases and individuals tend to recover

within a short time following standard treatment and rest. Investigators and study participants should be alert to the signs and symptoms of myocarditis and pericarditis (Gargano et al 2021).

2.3.3. Overall Benefit/Risk Conclusion

Three dose levels (50, 100, and 200 µg of total mRNA) of mRNA-1010 vaccine administered as a single IM injection will be evaluated in the Phase 1/2 part of the study. The safety, reactogenicity, and immunogenicity of the 3 dose levels of mRNA-1010 will be compared with placebo. The study schema for the Phase 1/2 part of the study is provided in Figure 1.

In the Phase 1/2 part of the study, each vaccination group will have 2 stages: initial stage and expansion stage. A total of 36 participants (9 participants in each vaccination group) will be randomly assigned in the initial stage in parallel fashion. Enrollment and vaccination in the expansion stage will begin only after an internal safety team (IST) performs a blinded review of all available safety data up to 7 days after vaccination from the 36 participants in the initial stage and determines that no pause rules have been met. As an additional safeguard, the vaccine administration in the Phase 1/2 part of this FIH study is planned outside the NH influenza season.

In the Phase 2 NH part, the three dose levels of mRNA-1010 vaccine to be evaluated are 25, 50, and 100 µg of total mRNA based on interim analysis (IA) of the Phase 1/2 part. In addition, the Phase 2 NH part of the study will also include a composition change of mRNA-1010 (strain update for the 2021-2022 NH season), as well as the inclusion of a licensed active comparator. In this part, vaccine administration is planned during the NH influenza season. The study schema for the Phase 2 NH part of the study is provided in Figure 2.

In the Phase 2 Extension part, the 3 dose levels of mRNA-1010 vaccine to be evaluated are [redacted] of total mRNA based on IA of the Phase 1/2 part. The Phase 2 Extension part of the study will include the same composition of mRNA-1010 as the Phase 2 NH part. In this part, vaccine administration is planned outside the NH influenza season. The study schema for the Phase 2 Extension part of the study is provided in Figure 3.

Serological data from all participants will be used to infer vaccine immunogenicity. All participants will be followed up for 6 months after the study vaccination. Safety will be monitored throughout the study (Section 8.4).

Considering the nonclinical data for the mRNA-1010 vaccine and the safety data for the other mRNA vaccines manufactured by the Sponsor to date that contain the proprietary [redacted] lipid formulation, the Sponsor considers the potential benefits of participation to exceed the risks.

3. OBJECTIVES AND ENDPOINTS

3.1. Phase 1/2

The objectives of the Phase 1/2 part of the study and the endpoints associated with each objective are provided in Table 4.

Table 4 Phase 1/2 - Study Objectives and Endpoints

Objectives	Endpoints
Primary	
• To evaluate the safety and reactogenicity of 3 dose levels (50, 100, and 200 µg) of mRNA-1010 vaccine administered as a single dose	• Frequency and grade of each solicited local and systemic reactogenicity AR during a 7-day follow-up period after vaccination<br>• Frequency and severity of any unsolicited AEs during the 28-day follow-up period after vaccination<br>• Frequency of any SAEs, AESIs, and MAAEs from Day 1 through Day 181/EoS<br>• Safety laboratory abnormalities through 7 days after vaccination
• To evaluate the humoral immunogenicity of 3 dose levels (50, 100, and 200 µg) of mRNA-1010 vaccine administered as a single dose against vaccine-matched influenza A and B strains at Day 29	• GMT and GMFR at Day 29 compared with Day 1 (baseline) and percentage of participants with seroconversion, defined as a Day 29 titer ≥ 1:40 if baseline is < 1:10 or a 4-fold or greater rise if baseline is ≥ 1:10 in anti-HA antibodies measured by HAI assay
Secondary	
• To evaluate the humoral immunogenicity of 3 dose levels (50, 100, and 200 µg) of mRNA-1010 vaccine administered as a single dose against vaccine-matched influenza A and B strains at all evaluable humoral immunogenicity time points	• GMT and GMFR of anti-HA antibodies as measured by HAI or MN assays at all evaluable humoral immunogenicity time points compared with Day 1 (baseline)

Objectives	Endpoints
Exploratory (May be Performed)	
[redacted]	

Abbreviations: AE = adverse event; AESI = adverse event of special interest; AR = adverse reaction; EoS = end of study; Fc = fragment crystallizable; GMFR = geometric mean fold rise; GMT = geometric mean titer; HA = hemagglutinin; HAI = hemagglutination inhibition; ILI = influenza-like illness; MAAE = medically attended adverse event; MN = microneutralization; RT-PCR = reverse transcription polymerase chain reaction; SAE = serious adverse event.

3.2. Phase 2 Northern Hemisphere and Phase 2 Extension

The objectives of the Phase 2 NH and Phase 2 Extension parts of the study and the endpoints associated with each objective are provided in Table 5.

Table 5 Phase 2 Northern Hemisphere and Phase 2 Extension - Study Objectives and Endpoints

Objectives	Endpoints
Primary	
• To evaluate the humoral immunogenicity of mRNA-1010 vaccine relative to that of an active comparator against vaccine-matched influenza A and B strains at Day 29	• GMT at Day 29 as measured by HAI<br>• Proportion of participants reaching seroconversion at Day 29 as measured by HAI
• To evaluate the safety and reactogenicity of mRNA-1010 vaccine	• Frequency and grade of each solicited local and systemic reactogenicity ARs during a 7-day follow-up period after vaccination<br>• Frequency and severity of any unsolicited AEs during the 28-day follow-up period after vaccination<br>• Frequency of any SAEs, AESIs, and MAAEs from Day 1 through Day 181/EoS
Secondary	
• To evaluate the humoral immunogenicity of each vaccine group against vaccine-matched influenza A and B strains at Day 29	• The frequency of participants with HAI seroconversion and the frequency of participants with an HAI titer ≥ 1:40 at Day 29<br>• GMFR comparing Day 29 to Day 1 (baseline) as measured by HAI

Objectives	Endpoints
Exploratory (May be Performed)	
[redacted]	

Abbreviations: AE = adverse event; AESI = adverse event of special interest; AR = adverse reaction; CDC = Centers for Disease Control and Prevention; EoS = end of study; GMFR = geometric mean fold rise; GMT = geometric mean titer; HA = hemagglutinin; HAI = hemagglutination inhibition; ILI = influenza-like illness; MAAE = medically attended adverse event; MN = microneutralization; RT-PCR = reverse transcription polymerase chain reaction; SAE = serious adverse event.

4. STUDY DESIGN

4.1. General Design

This FIH study is a Phase 1/2, randomized, observer-blind, dose-ranging study to evaluate the safety, reactogenicity, and immunogenicity of mRNA-1010 vaccine in adult participants ≥ 18 years of age. The study comprises 3 parts: Phase 1/2, Phase 2 NH, and Phase 2 Extension.

The Phase 1/2 part of the study will be a randomized, observer-blind, dose-ranging, placebo-controlled study to evaluate the safety, reactogenicity, and immunogenicity of mRNA-1010 vaccine in healthy adult participants ≥ 18 years of age. The Phase 2 NH and Phase 2 Extension parts of the study will be randomized, observer-blind, dose-ranging, active-controlled studies to evaluate the immunogenicity, reactogenicity, and safety of mRNA-1010 vaccine in medically stable adults 18 years and older.

All participants in each part of the study will participate in a screening period (up to 28 days before Day 1), treatment period (single dose of vaccine on Day 1), and a follow-up period (up to 6 months after vaccination).

In the Phase 1/2 part of the study, the mRNA-1010 vaccine to be tested includes mRNAs that encode for the surface glycoprotein HAs of the following influenza virus strains recommended by the WHO for 2020-2021 SH cell- or recombinant-based vaccines:

- A/Wisconsin/588/2019(H1N1)pdm09
- A/Hong Kong/45/2019(H3N2)
- B/Washington/02/2019 (B/Victoria lineage)
- B/Phuket/3073/2013 (B/Yamagata lineage)

In the Phase 2 NH and Phase 2 Extension parts of the study, the mRNA-1010 vaccine to be tested, includes mRNAs that encode for the surface glycoprotein HAs of the following influenza virus strains recommended by the WHO for 2021-2022 NH cell- or recombinant-based vaccines:

- A/Wisconsin/588/2019(H1N1)pdm09
- A/Cambodia/e0826360/2020 (H3N2)
- B/Washington/02/2019 (B/Victoria lineage)
- B/Phuket/3073/2013 (B/Yamagata lineage)

Different dose levels of mRNA-1010 vaccine will be evaluated. Placebo will be included in the Phase 1/2 part and an active comparator (licensed quadrivalent seasonal influenza vaccine) will be included in the Phase 2 NH and Phase 2 Extension parts.

In the Phase 1/2 part, each vaccination group will have 2 stages: initial stage and expansion stage. The schema for the Phase 1/2, Phase 2 NH, and Phase 2 Extension parts of the study are presented in Figure 1, Figure 2, and Figure 3, respectively. Table 6 (Parts A, B, and C) lists the vaccination groups and dose levels that will be evaluated in each part of the study; Part D lists the total number of study participants in each study part and overall.

Table 6 Vaccination Groups and Dose Levels

A) Phase 1/2

				Number of Participants		
				Phase 1/2		
Vaccination Group	Investigational Product	mRNA or HA (µg)	Total Dose (µg)	Initial Stage	Expansion Stage	Total
1	mRNA-1010	12.5	50	9	36	45
2	mRNA-1010	25	100	9	36	45
3	mRNA-1010	50	200	9	36	45
4	Placebo	–	–	9	36	45
**Total**				**36**	**144**	**180**

Abbreviations: HA = hemagglutinin; mRNA = messenger RNA.

B) Phase 2 NH

				Number of Participants
Vaccination Group	Investigational Product	mRNA or HA (µg)	Total Dose (µg)	Phase 2 NH
1	mRNA-1010	6.25	25	150
2	mRNA-1010	12.5	50	150
3	mRNA-1010	25	100	150
4	Active comparator	15	60	50
**Total**				**500**

Abbreviations: HA = hemagglutinin; mRNA = messenger RNA; NH = Northern Hemisphere.

C) Phase 2 Extension

			Number of Participants
**Vaccination Group**	**Investigational Product**	**Total Dose (μg)**	**Phase 2 Extension**
1	mRNA-1010	[redacted]	50
2	mRNA-1010	[redacted]	50
3	mRNA-1010	[redacted]	50
4	Active comparator	60	50
**Total**			**200**

Abbreviations: HA = hemagglutinin; mRNA = messenger RNA.
Note: Mass percent in mRNA-1010 is 25% for each of the 4 HAs.

D) Total Number of Participants

**Study Part**	**Number of Participants**
Phase 1/2	180
Phase 2 NH	500
Phase 2 Extension	200
**Total**	**880**

Abbreviations: NH = Northern Hemisphere.

Healthy (Phase 1/2) and medically stable (Phase 2 NH and Phase 2 Extension) adults ≥ 18 years of age will be screened and enrolled. For the Phase 2 NH and Phase 2 Extension parts, participants with chronic diseases requiring ongoing medical intervention within the last 3 months prior to enrollment as well as those with immunocompromising conditions or medications will be excluded. The complete lists of inclusion and exclusion criteria for the Phase 1/2, Phase 2 NH, and Phase 2 Extension parts of the study are provided in Section 5.1 (Phase 1/2) and Section 5.2 (Phase 2 NH and Phase 2 Extension).

In the Phase 1/2 part, approximately 180 participants will be randomly assigned in a 1:1:1:1 ratio to receive mRNA-1010 50 μg, mRNA-1010 100 μg, mRNA-1010 200 μg, or placebo, with approximately 45 participants randomly assigned to each vaccination group. A total of 36 participants (9 participants in each vaccination group) will be randomly assigned in the initial stage of the study. The IST will perform a blinded review of all safety data up to 7 days after vaccination from the 36 participants in the initial stage. After the IST confirms that no pause rules (Section 7.1) have been met in the 36 participants in the initial stage, enrollment will begin in the expansion stage. A total of 144 participants (36 participants in each vaccination group) will be randomly assigned in the expansion stage of the study. Randomization in the expansion stage will be stratified by age (18 to < 50 years versus ≥ 50 years) and will be balanced across the 2 age groups within each vaccination group.

The Phase 2 NH part of the study will enroll and randomize approximately 500 participants in a 3:3:3:1 ratio to 1 of 4 vaccination groups to receive a single dose of mRNA-1010 at different dose levels (25, 50, or 100 µg total mRNA) or a single dose of a licensed quadrivalent seasonal influenza vaccine (active comparator). Randomization to individual vaccination groups will proceed with parallel randomization among the 3 dose levels of mRNA-1010 (25, 50, and 100 µg total mRNA; Vaccination Groups 1 to 3) and the active comparator (Vaccination Group 4). Randomization will be stratified by age categories (18 to < 50 years, 50 to < 65 years, or ≥ 65 years) and vaccination status in the previous flu season (received or not received).

The Phase 2 Extension part of the study will enroll and randomize approximately 200 participants in a 1:1:1:1 ratio to 1 of 4 vaccination groups to receive a single dose of mRNA-1010 at different dose levels [redacted] or a single dose of a licensed quadrivalent seasonal influenza vaccine (active comparator). Randomization to individual vaccination groups will proceed with parallel randomization among the 3 dose levels of mRNA-1010 ([redacted]; Vaccination Groups 1 to 3) and the active comparator (Vaccination Group 4). Randomization will be stratified by age categories (18 to < 50 years, or ≥ 50 years).

Table 1, Table 2, and Table 3 display the Schedule of Events (SoE) for the Phase 1/2, Phase 2 NH, and Phase 2 Extension parts of the study, respectively. The Phase 1/2 part of the study comprises 6 scheduled study site visits: Screening, Day 1, Day 8, Day 29 (Month 1), Day 57 (Month 2), and Day 181 (Month 6). The Phase 2 NH and Phase 2 Extension parts of the study comprise 5 scheduled study site visits: Screening, Day 1, Day 29 (Month 1), Day 91 (Month 3), and Day 181 (Month 6). The Phase 2 Extension part also contains an optional study site visit on Day 4 for blood sample collection. There are also scheduled safety phone calls to collect medically attended AEs (MAAEs), AEs leading to withdrawal from study participation, serious AEs (SAEs), AEs of special interest (AESIs), information about concomitant medications associated with these events, and to collect information about receipt of nonstudy vaccinations temporally associated with these events. These phone calls are scheduled monthly from Day 91 through Day 151 during the Phase 1/2 part and on Days 8 and 15 during the Phase 2 NH and Phase 2 Extension parts of the study.

The study duration will be approximately 7 months for each participant enrolled in each part of the study: a screening period of up to 1 month and a study period of 6 months that includes a single dose of vaccine on Day 1. The participant’s final visit will be on Day 181 (Month 6), six months after the study vaccination.

All participants in each part of the study will be followed for safety and reactogenicity. On Day 1, participants will be instructed how to document, and report solicited adverse reactions (ARs) and ILI symptoms (for Phase 2 NH part only) with a provided electronic diary (eDiary) (Section 8.1.1). Solicited ARs will be assessed for 7 days (the day of injection and the following 6 days) after the

study vaccination, and unsolicited AEs will be assessed for 28 days after the study vaccination; SAEs, AESIs, and MAAEs will be assessed from Day 1 throughout the study.

All participants in the Phase 1/2 part of the study will provide nasopharyngeal (NP) swab samples before the injection on Day 1 for assessment of asymptomatic infection with respiratory pathogens, including influenza virus and SARS-CoV-2. A study site visit or a consultation (unscheduled study visit) will be arranged within 24 hours or as soon as possible to collect an NP swab sample if participants experience symptoms consistent with the CDC definition of ILI (Section 8.4.6) and/or COVID-19 during the course of the study.

For the Phase 2 NH part, as part of the safety assessments, active and passive surveillance for ILI will be conducted between Day 1 through Day 181/end of study (EoS). For active surveillance, participants will be instructed to report whether ILI symptoms have been experienced, 3 to 4 times weekly from Day 1 through Day 29 and twice weekly from Day 30 through Day 181/EoS, via eDiary or telephone calls. If symptoms occur, participants will be directed to return to the study site as soon as possible, but no later than 72 hours after the onset of symptoms, for medical evaluation and an NP swab. Participants will be contacted by the study site if they have missed reporting in the eDiary. If there is no response to an eDiary prompt for 2 consecutive entries, the study site staff will contact the participant by telephone. For passive surveillance, participants will be instructed to report symptoms of ILI any time from Day 1 through Day 181/EoS. Participants in the Phase 2 NH part who manifest protocol-defined ILI (Section 8.4.6) between Day 1 through Day 29 will be evaluated by real-time reverse transcription polymerase chain reaction (RT-PCR) testing of an NP specimen for influenza (and other respiratory pathogens). An NP swab specimen for pathogens, including influenza virus and SARS-CoV-2 will be collected any time from Day 1 through Day 181/EoS if participants have protocol-defined ILI or symptoms suggestive of COVID-19 or other upper or lower respiratory infection at the Investigator's discretion.

For the Phase 2 Extension part, a study site visit or a consultation (unscheduled study visit) will be arranged within 72 hours after the onset of symptoms to collect an NP swab sample if participants experience symptoms consistent with the US CDC definition of ILI and/or COVID-19 during the course of the study.

All participants in the Phase 1/2 part of the study will provide a blood specimen before injection on Day 1 and additional blood specimens through the next 6 months for immunogenicity (on Days 8, 29, and 181/EoS). In the Phase 2 NH and Phase 2 Extension parts, all participants will provide a blood specimen for immunogenicity assessments before injection on Day 1 and then on Day 29. In addition, blood samples from a subset of participants at Days 91 and 181/EoS may be used for additional assessments. The Phase 2 Extension part also contains an optional study site visit on Day 4 for blood sample collection for potential biomarker analysis. Furthermore, blood

samples for safety or other medical concerns may be collected according to the Investigator's judgment at scheduled study site visits during each part of the study.

Participants may experience AEs that necessitate an unscheduled visit. There may also be situations in which the Investigator asks a participant to report for an unscheduled visit following the report of an AE. Additional examinations may be conducted at these visits as necessary to ensure the safety and well-being of participants during the study. Electronic case report forms (eCRFs) should be completed for each unscheduled visit.

The study will be observer-blind as to which investigational product (IP; mRNA-1010 or placebo in the Phase 1/2 part; mRNA-1010 or the active comparator in the Phase 2 NH and Phase 2 Extension parts) or dose level is administered. Participants will receive the IP by 0.5-mL IM injection on Day 1. All participants, study site staff involved in participant assessment, and Sponsor personnel (or its designees) will be blinded to individual dosing assignment until the study database is locked and unblinded. Details regarding blinding are provided in Section 9.1 and the study Data Blinding Plan. Preparation of IP for administration will be conducted on site by an unblinded staff member who has no role in the observation or assessment of study participants.

Safety monitoring for this study will include blinded study team members, inclusive of, at a minimum, the Sponsor's medical monitor, medical monitor of the contract research organization (CRO), a blinded IST, and an unblinded Data and Safety Monitoring Board (DSMB). The study team will conduct ongoing blinded safety reviews during the study and will be responsible for notifying the IST and DSMB of potential safety signal events or the triggering of pause rules (Section 7.1).

For the initial stage in the Phase 1/2 part of the study, the IST will conduct a blinded review of all available safety data after at least 36 participants (9 participants per vaccination group) have completed their Day 8 Visit. Vaccination of the remaining participants in the expansion stage will continue in each vaccination group if no pause rules have been met and the safety and tolerability are acceptable. Prior to the start of the Phase 2 NH part of the study, the IST will also conduct a blinded review of available safety data from the Phase 1/2 part. Finally, the IST will also conduct ad hoc reviews as requested by the study medical monitor and the study team. An independent unblinded DSMB will be used throughout the conduct of this study. This committee will be composed of independent members with relevant therapeutic and/or biostatistical expertise to allow for the ongoing unblinded review of safety data from this study population. Safety data will be reviewed according to intervals defined in the DSMB charter and will also occur as needed when study stopping or pausing criteria are met, or as otherwise requested by the study team and/or IST. See Section 11.1.11 for details on the IST and DSMB constituted in this study.

For the Phase 1/2 part, there is 1 planned IA, which will include safety and immunogenicity data of all participants (all vaccination groups) after completion of the Day 29 Visit. For the Phase 2 NH and Phase 2 Extension parts, an IA of safety (reactogenicity and immunogenicity as applicable) will be performed after participants have completed the Day 29 Visit for each part of the study. See Section 9.6 for details.

Immunizations for the Phase 1/2 part of the study are planned during the NH summer and all participants will be recommended to receive a licensed 2021-2022 NH seasonal influenza vaccine during their participation in the study after completion of the Day 29 Visit. Immunizations for the Phase 2 NH part of the study are planned during the NH influenza season. Immunizations for the Phase 2 Extension part are planned during the NH spring.

4.2. Scientific Rationale for Study Design

This study is designed as an observer-blind study. In the Phase 1/2 part of the study, participants in Vaccination Groups 1 to 3 will receive mRNA-1010 vaccine and those in Vaccination Group 4 will receive placebo. The placebo will be used for descriptive comparisons of safety and immunogenicity. In the Phase 2 NH part, participants in Vaccination Groups 1 to 3 will receive mRNA-1010 vaccine and those in Vaccination Group 4 will receive a licensed quadrivalent seasonal influenza vaccine in order to assess noninferior immunogenicity. In the Phase 2 Extension part, participants in Vaccination Groups 1 to 3 will receive mRNA-1010 vaccine and those in Vaccination Group 4 will receive a licensed quadrivalent seasonal influenza vaccine in order to compare immunogenicity.

In this observer-blind study, all participants, study site staff involved in participant assessment, and Sponsor personnel (or its designees) will be blinded to participant vaccine allocation. A limited number of Sponsor and/or CRO personnel will be unblinded to conduct safety data analyses for the DSMB safety data reviews (as described in the DSMB charter) and perform the IA. Unblinded study personnel, who will not participate in any other aspect of the study, will perform IP accountability, dose preparation, and IP administration.

In the Phase 1/2 part, all participants will provide NP swab samples before the injection on Day 1 for assessment of asymptomatic infection with respiratory pathogens, including influenza virus and SARS-CoV-2, as influenza or COVID-19 symptoms may confound reactogenicity assessments. Furthermore, if a participant experiences an MAAE or any signs or symptoms suggesting ILI and/or COVID-19, an additional NP swab sample will be collected to confirm the diagnosis via RT-PCR. In the Phase 2 NH and Phase 2 Extension parts, the NP swab samples for assessment of infection with respiratory pathogens, including influenza virus and SARS-CoV-2, will be collected anytime from Day 1 through Day 181/EoS if the participants have protocol-defined ILI (Phase 2 NH) or CDC-defined ILI (Phase 2 Extension) or symptoms

suggestive of COVID-19 or other upper or lower respiratory infection at the Investigator's discretion. Participants who manifest ILI between Day 1 through Day 29 will be evaluated by RT-PCR testing of an NP specimen for influenza (and other respiratory pathogens). Additionally, in all parts of the study, clinical information may be collected to evaluate the severity of the clinical case.

4.3. Choice of Vaccine Dose

No clinical studies of mRNA-1010 have been completed to date.

Previous studies of the Sponsor's mRNA/LNP [redacted] platform have assessed dose levels as high as 250 µg for mRNA-1273, a SARS-CoV-2 vaccine (Jackson et al 2020, NCT04283461), and mRNA-1893, a Zika vaccine (unpublished data, NCT04064905). Additionally, doses up to 300 µg of the multivalent mRNA-1647, a cytomegalovirus vaccine (unpublished data, NCT03382405), and the multivalent mRNA-1653, a combination vaccine against human metapneumovirus and parainfluenza virus type 3 (unpublished data, NCT03392389), have been tested in Phase 1 studies. The vaccines were generally well tolerated at these dose levels.

The Sponsor has previously evaluated the immunogenicity of 2 mRNA vaccines containing the HA glycoprotein from the H10N8 and H7N9 influenza viruses. Although differences in the mRNA/LNP platform and the administration schedule (2 doses were given 3 weeks apart) limit extrapolation of the safety data, these mRNA vaccines against H10N8 and H7N9 influenza viruses elicited robust humoral immune responses and were well tolerated up to dose levels of 100 µg (for each HA type; Feldman et al 2019).

The FIH Phase 1/2 clinical study of mRNA-1010 vaccine will evaluate the immunogenicity of mRNA-1010 at proposed total dose levels of 50 µg (12.5 µg/mRNA), 100 µg (25 µg/mRNA), and 200 µg (50 µg/mRNA), administered as a single IM injection in healthy adults ≥ 18 years of age. Based on IA results of the Phase 1/2 study, the 200 µg dose of mRNA-1010 did not meet the Sponsor's desired reactogenicity profile. In the Phase 2 NH part of the study, the Sponsor intends to test a lower dose level of 25 µg in lieu of 200 µg, keeping the previous dose levels of 50 and 100 µg unchanged. Thus, the Phase 2 NH part will evaluate the immunogenicity of mRNA-1010 at proposed total dose levels of 25 µg (6.25 µg/mRNA), 50 µg (12.5 µg/mRNA), and 100 µg (25 µg/mRNA), administered as a single IM injection in medically stable adults ≥ 18 years of age. Based on IA of the Phase 1/2 study, vaccination with mRNA-1010 elicited HAI antibodies at all dose levels tested. In the Phase 2 Extension part of the study, the Sponsor intends to test lower dose levels of [redacted] of mRNA-1010, and to keep the Phase 2 NH mRNA-1010 dose level of [redacted] and the active comparator unchanged. Thus, the Phase 2 Extension part will evaluate the immunogenicity of mRNA-1010 at proposed total dose levels of [redacted] administered as a single IM injection in medically stable adults ≥ 18 years of age.

4.4. End of Study Definition

For each part of the study, participants are considered to have completed the study if they complete the final visit on Day 181 (Month 6), six months after the study vaccination on Day 1.

The study will be considered ended at the completion of the last visit of the last participant in the Phase 2 Extension part of the study or the last scheduled procedure as shown in the SoE (Table 3) for the last participant in the Phase 2 Extension part of the study.

5. STUDY POPULATION

Prospective approval of protocol deviations to recruitment and enrollment criteria, also known as protocol waivers or exemptions, is not permitted.

5.1. Phase 1/2

5.1.1. Inclusion Criteria

Each participant must meet all of the following criteria at the Screening Visit and at Day 1, unless noted otherwise, to be enrolled in this study:

1. Is an adult, ≥ 18 years of age at the time of consent (Screening Visit), who is in good health, in the opinion of the Investigator, based on review of medical history and physical examination performed at screening.
2. Understands and is willing and physically able to comply with protocol-mandated follow-up, including all procedures, in the opinion of the Investigator.
3. Has provided written informed consent for participation in this study, including all evaluations and procedures as specified in this protocol.
4. Has a body mass index (BMI) of 18 kg/m^2 to 35 kg/m^2 (inclusive) at the Screening Visit.
5. If female and is not of childbearing potential, may be enrolled in the study. The definition of a female of childbearing potential is provided in Appendix 2 (Section 11.2). A follicle-stimulating hormone (FSH) level may be measured at the discretion of the Investigator to confirm postmenopausal status.
6. If female and of childbearing potential, meets all of the following criteria to be enrolled in the study:
   - Has a negative pregnancy test at the Screening Visit and on the day of vaccination (Day 1).
   - Has practiced adequate contraception (Section 11.2) or has abstained from all activities that could result in pregnancy for at least 28 days prior to Day 1. Adequate female contraception is defined as consistent and correct use of an FDA-approved contraceptive method in accordance with the product label.
   - Has agreed to continue adequate contraception through 3 months following vaccine administration.
   - Is not currently breastfeeding.

5.1.2. Exclusion Criteria

Participant meeting any of the following criteria at the Screening Visit or at Day 1, unless noted otherwise, will be excluded from the study:

1. Has had significant exposure to someone with laboratory-confirmed SARS-CoV-2 infection, COVID-19, or ILI in the past 14 days prior to the Screening Visit, as defined by the CDC as close contact with someone who has COVID-19 (CDC 2021a).
2. Has a positive SARS-CoV-2 RT-PCR or antigen test in the past 10 days prior to the Screening Visit.
   a. A participant who has a positive SARS-CoV-2 serological test in the past 10 days prior to the Screening Visit but is otherwise asymptomatic and does not have a positive SARS-CoV-2 RT-PCR or antigen test at the Screening Visit may still be enrolled if they meet the study eligibility criteria.
   b. A participant who has a positive SARS-CoV-2 RT-PCR or antigen test in the past 10 days prior to the Screening Visit may be enrolled 10 days after symptom onset or 10 days after the date of the first positive SARS-CoV-2 RT-PCR test without repeat testing, provided the participant is asymptomatic and meets the study eligibility criteria as well as the CDC recommendation for duration of isolation and precautions (CDC 2021b).
   c. Additionally, a participant who meets all study eligibility criteria (note exclusion criterion No. 4) at the Screening Visit but later tests positive for SARS-CoV-2 at Visit 1 will continue participation in the study.
3. Has clinical screening laboratory values (white blood cell [WBC] count, hemoglobin, platelets, alanine aminotransferase [ALT], aspartate aminotransferase [AST], creatinine, alkaline phosphatase, and total bilirubin) > Grade 1.
4. Is acutely ill or febrile (body temperature ≥ 38.0°C/100.4°F) 72 hours prior to or at the Screening Visit or Day 1. Participants meeting this criterion may be rescheduled within the 28-day screening window and will retain their initially assigned participant number.
5. Has a pre-existing medical condition that is not stable, at the discretion of the Investigator. A stable medical condition is defined as disease not requiring significant change in therapy or hospitalization for worsening disease during the 2 months before enrollment. Participants may be rescreened if they are not medically stable at the Screening Visit. Medical conditions include (but are not limited to) the following:
   a. Uncontrolled hypertension or systolic blood pressure (BP) > 150 mm Hg or diastolic BP > 90 mm Hg at the Screening Visit.

   b. Congestive heart failure.
   c. Unstable angina or exacerbation of coronary artery disease within 6 months before the day of vaccination (Day 1) requiring cardiac intervention or new cardiac medications to control symptoms.
   d. Diabetes requiring the use of medicine (injectable or oral) or not controlled with diet.
   e. Chronic obstructive pulmonary disease, asthma requiring daily use of a bronchodilator or inhaled/systemic corticosteroids, or other chronic lung diseases such as pulmonary fibrosis.

6. Has a medical, psychiatric, or occupational condition that may pose additional risk as a result of participation or that could interfere with safety assessments or interpretation of results according to the Investigator's judgment.
7. Has a current or previous diagnosis of immunocompromising condition, immune-mediated disease, or other immunosuppressive condition.
8. Has received systemic immunosuppressants or immune-modifying drugs for > 14 days in total within 6 months prior to Screening Visit (for corticosteroids ≥ 10 mg/day of prednisone or equivalent) or is anticipating the need for immunosuppressive treatment at any time during participation in the study.
9. Has a history of anaphylaxis, urticaria, or other significant AR requiring medical intervention after receipt of a vaccine or any of the components contained in the IP.
10. Has a history of coagulopathy or bleeding disorder considered a contraindication to IM injection or phlebotomy.
11. Has received or plans to receive any licensed vaccine ≤ 28 days prior to the IP injection (Day 1) or plans to receive a licensed vaccine within 28 days after the IP injection, with the exception of vaccines authorized or approved for the prevention of COVID-19 (regardless of type of vaccine) that become available to participants during the study. Efforts should be made to space study vaccination and COVID-19 vaccination by at least 7 and preferably 14 days, but COVID-19 vaccination should not be delayed.
12. Has received a seasonal influenza vaccine or any other investigational influenza vaccine after 01 Jan 2021.
13. Has received systemic immunoglobulins or blood products within 3 months prior to the Screening Visit or plans to receive them during the study.
14. Has a diagnosis of malignancy within the previous 10 years (excluding nonmelanoma skin cancer).

15. Has donated ≥ 450 mL of blood products within 28 days prior to the Screening Visit or plans to donate blood products during the study.
16. Has participated in an interventional clinical study within 28 days prior to the Screening Visit based on the medical history interview or plans to do so while participating in this study.
17. Is an immediate family member or household member of study personnel, study site staff, or Sponsor personnel.

5.2. Phase 2 Northern Hemisphere and Phase 2 Extension

5.2.1. Inclusion Criteria

Each participant must meet all of the following criteria at the Screening Visit and at Day 1, unless noted otherwise, to be enrolled in this study:

1. Is an adult, ≥ 18 years of age at the time of consent (Screening Visit), who in the opinion of the Investigator, is medically stable based on review of medical history and physical examination performed at screening. Medically stable is defined as disease not requiring significant change in therapy or hospitalization for worsening disease during the 3 months before enrollment. Participants may be rescreened if they are not medically stable at the Screening Visit.
2. Understands and is willing and physically able to comply with protocol-mandated follow-up, including all procedures, in the opinion of the Investigator.
3. Has provided written informed consent for participation in this study, including all evaluations and procedures as specified in this protocol.
4. If female and is not of childbearing potential, may be enrolled in the study. The definition of a female of childbearing potential is provided in Appendix 2 (Section 11.2). An FSH level may be measured at the discretion of the Investigator to confirm postmenopausal status.
5. If female and of childbearing potential, meets all of the following criteria to be enrolled in the study:
   - Has a negative pregnancy test at the Screening Visit and on the day of vaccination (Day 1).
   - Has practiced adequate contraception (Section 11.2) or has abstained from all activities that could result in pregnancy for at least 28 days prior to Day 1.

- Has agreed to continue adequate contraception through 3 months following vaccine administration. Adequate female contraception is defined as consistent and correct use of an FDA-approved contraceptive method in accordance with the product label.
- Is not currently breastfeeding.

5.2.2. Phase 2 Northern Hemisphere Exclusion Criteria

Participant meeting any of the following criteria at the Screening Visit or at Day 1, unless noted otherwise, will be excluded from the study:

1. Has had close contact to someone with SARS-CoV-2 infection or COVID-19 as defined by the CDC (CDC 2021a) in the past 14 days prior to the Screening Visit, unless the participant has been fully vaccinated for COVID-19.
2. Is acutely ill or febrile (body temperature ≥ 38.0°C/100.4°F) 72 hours prior to or at the Screening Visit or Day 1. Participants meeting this criterion may be rescheduled within the 28-day screening window and will retain their initially assigned participant number.
3. Has a medical, psychiatric, occupational condition, or history of substance abuse that may pose additional risk as a result of participation or that could interfere with safety assessments or interpretation of results according to the Investigator's judgment.
4. Has a current or previous diagnosis of immunocompromising/immunosuppressive condition, immune-mediated disease requiring immune-modifying therapy, asplenia, recurrent severe infections (human immunodeficiency virus [HIV]-positive participants on antiretroviral therapy with cluster of differentiation [CD] 4 count ≥ 350 cells/mm^3 and HIV-RNA ≤ 500 copies/mL within the past 12 months are permitted).
5. Has received systemic immunosuppressants or immune-modifying drugs for > 14 days in total within 6 months prior to the Screening Visit (for corticosteroids ≥ 10 mg/day of prednisone or equivalent) or is anticipating the need for systemic immunosuppressive treatment at any time during participation in the study.
6. Has a history of anaphylaxis, urticaria, or other significant AR requiring medical intervention after receipt of a vaccine or any of the components contained in the mRNA-1010 or the comparator vaccine, which is an egg-based influenza vaccine.
7. Has coagulopathy or bleeding disorder considered a contraindication to IM injection or phlebotomy.
8. Has received or plans to receive any licensed vaccine ≤ 28 days prior to the IP injection (Day 1) or plans to receive a licensed vaccine within 28 days after the IP vaccine injection, with the exception of vaccines authorized or approved for the prevention of

COVID-19 (regardless of type of vaccine) that become available to participants during the study. Efforts should be made to space study vaccination and COVID-19 vaccination by at least 7 and preferably 14 days, but COVID-19 vaccination should not be delayed.

9. Has received a seasonal influenza vaccine or any other investigational influenza vaccine within 6 months prior to the Screening Visit.
10. Had tested positive for influenza by CDC-recommended testing methods (CDC 2020c) within 6 months prior to the Screening Visit.
11. Has received systemic immunoglobulins or blood products within 3 months prior to the Screening Visit or plans to receive them during the study.
12. Has donated ≥ 450 mL of blood products within 28 days prior to the Screening Visit or plans to donate blood products during the study.
13. Has participated in an interventional clinical study within 28 days prior to the Screening Visit based on the medical history interview or plans to do so while participating in this study.
14. Is an immediate family member or household member of study personnel, study site staff, or Sponsor personnel.

5.2.3. Phase 2 Extension Exclusion Criteria

Participant meeting any of the following criteria at the Screening Visit or at Day 1, unless noted otherwise, will be excluded from the study:

1. Has had close contact to someone with SARS-CoV-2 infection or COVID-19 as defined by the CDC in the past 10 days prior to the Screening Visit.
2. Is acutely ill or febrile (body temperature ≥ 38.0°C/100.4°F) 72 hours prior to or at the Screening Visit or Day 1. Participants meeting this criterion may be rescheduled within the 28-day screening window and will retain their initially assigned participant number.
3. Has a medical, psychiatric, occupational condition, or history of substance abuse that may pose additional risk as a result of participation or that could interfere with safety assessments or interpretation of results according to the Investigator's judgment.
4. Has a current or previous diagnosis of immunocompromising/immunosuppressive condition, immune-mediated disease requiring immune-modifying therapy, asplenia, recurrent severe infections (HIV-positive participants on antiretroviral therapy with CD 4 count ≥ 350 cells/mm^3 and HIV-RNA ≤ 500 copies/mL within the past 365 days are permitted).

5. Has received systemic immunosuppressants or immune-modifying drugs for > 14 days in total within 180 days prior to Screening Visit (for corticosteroids ≥ 10 mg/day of prednisone or equivalent) or is anticipating the need for systemic immunosuppressive treatment at any time during participation in the study.
6. Has a history of anaphylaxis, urticaria, or other significant AR requiring medical intervention after receipt of a vaccine or any of the components contained in the mRNA-1010 or the comparator vaccine, which is an egg-based influenza vaccine.
7. Has coagulopathy or bleeding disorder considered a contraindication to IM injection or phlebotomy.
8. Has received or plans to receive any licensed vaccine ≤ 28 days prior to the IP injection (Day 1) or plans to receive a licensed vaccine within 28 days after the IP injection, with the exception of vaccines authorized or approved for the prevention of COVID-19 (regardless of type of vaccine) that become available to participants during the study. Efforts should be made to space study vaccination and COVID-19 vaccination by at least 7 and preferably 14 days, but COVID-19 vaccination should not be delayed.
9. Has received a seasonal influenza vaccine or any other investigational influenza vaccine within 180 days prior to the randomization visit.
10. Had tested positive for influenza by CDC-recommended testing methods within 180 days prior to the Screening Visit.
11. Has received systemic immunoglobulins or blood products within 90 days prior to the Screening Visit or plans to receive them during the study.
12. Has donated ≥ 450 mL of blood products within 28 days prior to the Screening Visit or plans to donate blood products during the study.
13. Has participated in an interventional clinical study within 28 days prior to the Screening Visit based on the medical history interview or plans to do so while participating in this study.
14. Is an immediate family member or household member of study personnel, study site staff, or Sponsor personnel.

5.3. Lifestyle Restrictions

Participants must not eat or drink anything hot or cold within 10 minutes before oral temperature is taken. Participants in the study should defer vaccination with licensed seasonal influenza vaccine until after completion of their Day 29 Visit, if seasonal influenza vaccine is available and they choose to receive it.

5.4. Screen Failures

Screen failures are defined as participants who consent to participate in the clinical study but are not subsequently randomly assigned to treatment. A minimum set of screen failure information is required to ensure transparent reporting of screen failures to meet the Consolidated Standards of Reporting Trials publishing requirements and to respond to queries from regulatory authorities. Minimum information includes date of informed consent, demography, reason(s) for screen failure, and eligibility criteria.

6. STUDY TREATMENT

6.1. Investigational Products Administered

Investigational products in the Phase 1/2 part of the study are the mRNA-1010 vaccine candidate and placebo and those administered in the Phase 2 NH and Phase 2 Extension parts of the study are the mRNA-1010 vaccine candidate and licensed quadrivalent seasonal influenza vaccine (active comparator).

In the Phase 1/2 part of the study, the mRNA-1010 vaccine to be tested includes mRNAs that encode for the surface glycoprotein HAs of the following influenza virus strains recommended by the WHO for 2020-2021 SH cell- or recombinant-based vaccines:

- A/Wisconsin/588/2019(H1N1)pdm09
- A/Hong Kong/45/2019(H3N2)
- B/Washington/02/2019 (B/Victoria lineage)
- B/Phuket/3073/2013 (B/Yamagata lineage)

In the Phase 2 NH and Phase 2 Extension parts of the study, the mRNA-1010 vaccine to be tested, includes mRNAs that encode for the surface glycoprotein HAs of the following influenza virus strains recommended by the WHO for 2021-2022 NH cell- or recombinant-based vaccines:

- A/Wisconsin/588/2019(H1N1)pdm09
- A/Cambodia/e0826360/2020 (H3N2)
- B/Washington/02/2019 (B/Victoria lineage)
- B/Phuket/3073/2013 (B/Yamagata lineage)

mRNA-1010 is formulated in LNPs composed of 4 lipids (1 proprietary and 3 commercially available): [redacted] The mRNA-1010 injection is provided as sterile liquid for injection and is a white to off-white dispersion at a concentration of 0.5 mg/mL (in the Phase 1/2 part) or 0.4 mg/mL (in the Phase 2 NH and Phase 2 Extension parts) in 20 mM Tris buffer containing 87 mg/mL sucrose and 10.7 mM sodium acetate at pH 7.5.

In each part of the study, the mRNA-1010 vaccine will be administered as a single 0.5-mL IM injection at total dose levels ranging from 6.25 to 200 µg to participants according to the vaccination group assignment (Table 7, Table 8, and Table 9). The placebo (administered in the

Phase 1/2 part only) is 0.9% Sodium Chloride (normal saline) Injection, which meets the criteria of the US Pharmacopeia (USP) (Table 7).

Table 7 Phase 1/2 - Summary of Vaccination Groups

Vaccination Group	Investigational Product Administered	Dose	Number of Participants
1	mRNA-1010	50 µg	45
2	mRNA-1010	100 µg	45
3	mRNA-1010	200 µg	45
4	Placebo	–	45

The active comparator vaccine (administered in the Phase 2 NH and Phase 2 Extension parts only) will be administered as a single 0.5-mL IM injection to participants per the vaccination group assignment (Table 8 and Table 9).

Table 8 Phase 2 NH - Summary of Vaccination Groups

Vaccination Group	Investigational Product Administered	Dose	Number of Participants
1	mRNA-1010	25 µg	150
2	mRNA-1010	50 µg	150
3	mRNA-1010	100 µg	150
4	Licensed quadrivalent seasonal influenza vaccine (active comparator)	60 µg	50

Table 9 Phase 2 Extension - Summary of Vaccination Groups

Vaccination Group	Investigational Product Administered	Dose	Number of Participants
1	mRNA-1010	[redacted]	50
2	mRNA-1010	[redacted]	50
3	mRNA-1010	[redacted]	50
4	Licensed quadrivalent seasonal influenza vaccine (active comparator)	60 µg	50

6.2. Randomization

6.2.1. Phase 1/2

The Sponsor's Biostatistics Department or designee will generate the randomized allocation schedule(s) for vaccination group assignment. Overall, approximately 180 participants will be randomized in a 1:1:1:1 ratio to receive either mRNA-1010 50 µg, mRNA-1010 100 µg, mRNA-1010 200 µg, or placebo, with approximately 45 participants randomly assigned to each vaccination group. A total of 36 participants (9 participants in each vaccination group) will be randomly assigned in the initial stage of the study. A total of 144 participants (36 participants in each vaccination group) will be randomly assigned in the expansion stage of the study. Randomization in the expansion stage will be stratified by age (18 to < 50 years versus ≥ 50 years) and will be balanced across the 2 age groups within each vaccination group.

6.2.2. Phase 2 Northern Hemisphere

The Sponsor's Biostatistics Department or designee will generate the randomized allocation schedule(s) for vaccination group assignment. Overall, approximately 500 participants will be randomly assigned in a 3:3:3:1 ratio to receive either mRNA-1010 25 µg, mRNA-1010 50 µg, mRNA-1010 100 µg, or licensed quadrivalent seasonal influenza vaccine (active comparator), with approximately 150 participants randomly assigned to each mRNA-1010 vaccination group and 50 participants to the Active Comparator Group. Randomization will be stratified by age (18 to < 50 years, 50 to < 65 years, or ≥ 65 years) and vaccination status in the previous flu season (received or not received).

6.2.3. Phase 2 Extension

The Sponsor's Biostatistics Department or designee will generate the randomized allocation schedule(s) for vaccination group assignment. Overall, approximately 200 participants will be randomly assigned in a 1:1:1:1 ratio to receive either [redacted] or licensed quadrivalent seasonal influenza vaccine (active comparator), with approximately 50 participants randomly assigned to each mRNA-1010 vaccination group and 50 participants to the Active Comparator Group. Randomization will be stratified by age (18 to < 50 years or ≥ 50 years), with at least 30% of participants in each dose group being ≥ 50 years of age.

6.3. Preparation/Handling/Storage/Accountability

6.3.1. Preparation of Investigational Product

In the study, the IP will be prepared for each participant based on the vaccination group. For the Phase 1/2 part, the mRNA-1010 vaccine injection will have a volume of 0.5 mL and will contain

mRNA-1010 at a dose of 50 µg (Vaccination Group 1), 100 µg (Vaccination Group 2), or 200 µg (Vaccination Group 3). For the Phase 2 NH part, the mRNA-1010 vaccine injection will have a volume of 0.5 mL and will contain mRNA-1010 at a dose of 25 µg (Vaccination Group 1), 50 µg (Vaccination Group 2), or 100 µg (Vaccination Group 3). For the Phase 2 Extension part, the mRNA-1010 vaccine injection will have a volume of 0.5 mL and will contain mRNA-1010 at a dose of [redacted] (Vaccination Group 1), [redacted] (Vaccination Group 2), or [redacted] (Vaccination Group 3). For Vaccination Group 4 in the Phase 1/2 part of the study, placebo will be administered at a volume of 0.5 mL. For Vaccination Group 4 in the Phase 2 NH part of the study, the active comparator will be administered at a volume of 0.5 mL. For Vaccination Group 4 in the Phase 2 Extension part of the study, the active comparator will be administered at a volume of 0.5 mL. The IP (mRNA-1010, placebo, and active comparator) preparation instructions are detailed in the Pharmacy Manual.

6.3.2. Investigational Product Administration

The IP (mRNA-1010 or placebo during the Phase 1/2 part and mRNA-1010 or the active comparator during the Phase 2 NH and Phase 2 Extension parts) will be administered as a single IM injection into the deltoid muscle on Day 1. Preferably, the IP should be administered into the nondominant arm.

Participants will be monitored for a minimum of 60 minutes after administration of the IP. Assessments will include vital sign measurements and monitoring for local or systemic reactions as shown in the SoE for Phase 1/2 (Table 1), Phase 2 NH (Table 2), and Phase 2 Extension (Table 3) parts of the study.

The study site will be appropriately staffed with individuals with basic cardiopulmonary resuscitation training/certification. Either onsite resuscitation equipment and personnel or appropriate protocols for the rapid transport of a participant to a resuscitation area or facility are required.

6.3.3. Investigational Product Delivery and Receipt

The Sponsor or designee is responsible for the following:

- Supplying the IP (mRNA-1010, placebo, and active comparator).
- Confirming the appropriate labeling of the IP for clinical study use, so that it complies with the US legal requirements.

The Investigator is responsible for acknowledging receipt of the IP by a designated staff member at the site, which includes the following:

- Confirming that the IP was received in good condition.

- Confirming that the temperature during shipment from the Sponsor to the Investigator's designated storage location was appropriate.
- Confirming that the Sponsor has authorized the IP for use.
- Ensuring the appropriate dose level of the IP is properly prepared using aseptic technique.

Further description of the IP and corresponding instructions for the receipt, storage, preparation, administration, accountability, and destruction are described in the Pharmacy Manual.

6.3.4. Investigational Product Packaging and Labeling

The Sponsor will provide the Investigator (via the study site pharmacy) with adequate quantities of the IP. Sterile mRNA-1010 for the Phase 1/2 part is packaged in 2R glass vials with a 0.6-mL fill volume and that for the Phase 2 NH and Phase 2 Extension parts is packaged in 2R glass vials with a 0.8-mL fill volume. The IP will have all required labeling per regulations and will be supplied to the pharmacy in an unblinded manner. The Sponsor or Sponsor's designee will supply the 0.9% Sodium Chloride Injection, USP for use as both placebo (Phase 1/2) and diluent for the mRNA-1010 vaccine (as applicable) as well as the active comparator (Phase 2 NH and Phase 2 Extension). The 0.9% Sodium Chloride Injection, USP bears a commercial label and does not contain study-specific identification.

The IP will be packaged and labeled in accordance with the standard operating procedures of the Sponsor or of its designee, Code of Federal Regulations (CFR) Title 21 Good Manufacturing Practice guidelines, International Council for Harmonisation (ICH) Good Clinical Practice (GCP) guidelines, guidelines for Quality System Regulations, and applicable regulations.

6.3.5. Investigational Product Storage

mRNA-1010 must be stored at -60°C to -90°C (-76°F to -130°F) in a secure area with limited access and must be protected from moisture and light until it is prepared for administration (Section 6.3.1). The freezer should have automated temperature recording and a 24-hour alert system in place that allows for rapid response in case of freezer malfunction. There must be an available backup freezer. The freezer must be connected to a backup generator. In addition, for IP accountability, staff are required to keep a temperature log to establish a record of compliance with these storage conditions. The study site is responsible for reporting any IP that was not temperature controlled during shipment or storage. Such IP will be retained for inspection by the monitor and disposed of according to approved methods.

The 0.9% Sodium Chloride Injection USP should be stored at 20°C to 25°C (68°F to 77°F) in a restricted access area.

The active comparator should be stored in its original container and in accordance with the instructions in the Pharmacy Manual.

6.3.6. Investigational Product Accountability

The Investigator is responsible for ensuring that the IP accountability staff maintain accurate records in an IP accountability log of receipt of all IPs, site IP inventory, IP dispensing, IP injections, and return to the Sponsor or alternative disposition of used and unused IP vials.

A site monitor will review the inventory and accountability log during site visits and at completion of the study. Additional details are provided in the Pharmacy Manual.

6.3.7. Investigational Product Handling and Disposal

A site monitor will reconcile the IP inventory during study conduct and at EoS for compliance. Once fully reconciled at the site at EoS, unused IP will be destroyed onsite or, if necessary, returned to the sending depot for destruction.

6.4. Investigational Product Compliance

The IP will be administered at the study site under direct observation of medically qualified study staff, and IP administration will be appropriately recorded (date and time) in the eCRF. Qualified staff will confirm that the participant has received the entire dose of the IP. If a participant does not receive the IP, the reason for the missed dose will be recorded. Data will be reconciled with site accountability records to assess compliance.

The study site staff are responsible for ensuring that participants comply with the allowed study visit windows. If a participant misses a visit, every effort should be made to contact the participant and complete a visit within the defined visit window specified in the SoE (Table 1, Table 2, and Table 3). If a participant does not complete a visit within the time window, that visit will be classified as a missed visit and the participant will continue with subsequent scheduled study visits. All safety requirements of the missed visit will be captured and included in the subsequent visit.

6.5. Prior and Concomitant Medications

6.5.1. Prior Medications and Therapies

Information about prior medications (including any prescription or over-the-counter medications, vaccines, or blood products) taken by the participant within the 28 days before providing informed consent (or as designated in the inclusion/exclusion requirements) will be recorded in the participant's eCRF.

6.5.2. Concomitant Medications and Therapies

At each study visit, study site staff must question the participant regarding any medications taken and nonstudy vaccinations received by the participant and record the following information in the eCRF:

- All nonstudy vaccinations administered within the period starting 28 days before the IP injection and through Day 181/EoS.
- Any seasonal influenza vaccine administered since September 2020 (Phase 2 NH) or since September 2021 (Phase 2 Extension).
- Any authorized or investigational COVID-19 vaccine at any time before the IP injection and through Day 181/EoS.
- All concomitant medications taken through 28 days after the IP injection. Antipyretics and analgesics taken prophylactically (ie, taken in the absence of any symptoms in anticipation of an injection reaction) will be recorded as such.
- Systemic steroids (≥ 10 mg/day of prednisone or equivalent), immunosuppressants, immune-modifying drugs, immunoglobulins, and/or blood products administered at any time during the study period after the IP injection.
- Any concomitant medications relevant to or for the treatment of an SAE, AESI, or an MAAE from Day 1 through Day 181/EoS.
- The participant will be asked in the eDiary if they have taken any antipyretic or analgesic to treat or prevent fever or pain within 7 days after the study vaccination, including the day of injection. Reported antipyretic or analgesic medications should be recorded in the source document by the study site staff during the study visits after vaccination or via other participant interactions (eg, telephone calls).

Concomitant medications (including vaccinations) will be coded using the WHODrug Global.

If a participant takes a prohibited drug therapy, the Investigator and the CRO’s medical monitor will make a joint decision about continuing or withholding the study vaccination of the participant based on the time the medication was administered, the drug’s pharmacology and pharmacokinetics, and whether use of the medication will compromise the participant’s safety or interpretation of the data. It is the Investigator’s responsibility to ensure that details regarding the concomitant medications are adequately recorded in the eCRF.

6.5.3. Concomitant Medications and Vaccines That May Lead to the Elimination of a Participant From Per-Protocol Analyses

The use of the following concomitant medications and/or vaccines will not require withdrawal of the participant from the study but may determine a participant's evaluability in the Per-Protocol (PP) analysis (see Table 14 for definition of PP Set):

- Any investigational or nonregistered product (drug or vaccine) other than the IP used during the study period.
- Immunosuppressants or other immune-modifying drugs administered chronically (ie, more than 14 days in total) during the study period. For corticosteroids, this will mean that ≥ 10 mg/day of prednisone or equivalent is not permitted. Inhaled, nasal, and topical steroids are allowed.
- Long-acting immune-modifying drugs administered at any time during the study period (eg, infliximab).
- An authorized or licensed vaccine administered within 28 days after the study vaccination, except for any authorized or approved COVID-19 vaccine that was administered more than 14 days before or after the study vaccination (exclusion criterion No. 11; Section 5.1.2).
- Immunoglobulins and/or any blood products administered during the study period.

6.6. Intervention After End of the Study

The IP will not be available to participants after the EoS.

7. DELAY OR DISCONTINUATION OF STUDY INTERVENTION AND PARTICIPANT DISCONTINUATION/WITHDRAWAL

7.1. Pause Rules

Study pause rules will be continuously monitored during all periods of the study by the Investigators, IST, and DSMB (as warranted). If the Investigator, IST, or DSMB request that the study be paused due to a safety concern, further study vaccination in the affected vaccination group and at higher dose levels will be suspended, but all other planned procedures relating to safety, reactogenicity, and immunogenicity assessments will continue as described in the protocol. The Sponsor will notify the Center for Biologics Evaluation and Research within 48 hours in the event of a study pause.

7.1.1. Pause Rules for the Phase 1/2 Part of the Study

7.1.1.1. Pause Rules Based on the Occurrence of a Single Event and Adjudicated by the Data and Safety Monitoring Board

During the Phase 1/2 part of the study, the occurrence of any of the events listed in Table 10 regardless of the vaccination group, will result in immediate suspension of dosing and enrollment in the vaccination group and at higher dose levels. An unscheduled DSMB will be convened to assess specific data concerns and to make recommendations.

Table 10 Phase 1/2 Pause Rule Criteria, Events, and Thresholds - Single Event

Pause Rule	Event	Number of Participants
1	Any SAE that cannot be reasonably attributed to a cause other than study vaccination	≥ 1
2	Any Grade 4 AE[a] that cannot be reasonably attributed to a cause other than study vaccination	≥ 1
3	Any systemic immediate hypersensitivity reaction within 60 minutes after the study vaccination	≥ 1

Abbreviations: AE = adverse event; AR = adverse reaction; FDA = Food and Drug Administration; SAE = serious adverse event; US = United States.

[a] Grade 4 AE includes any Grade 4 solicited local or systemic AR and any Grade 4 laboratory abnormality. Grading of laboratory parameters will be based on the US FDA Guidance for Industry "Toxicity grading scale for healthy adult and adolescent volunteers enrolled in preventive vaccine clinical trials" (DHHS 2007b).

7.1.1.2. Pause Rules Based on the Occurrence of Events in a Proportion of Participants

During the Phase 1/2 part of the study, the occurrence of safety events that will pause study dosing based on defined threshold levels, which are aggregate incidences relative to the number of exposed participants within a vaccination group, are summarized in Table 11.

Table 11 Phase 1/2 Pause Rule Criteria, Events, and Thresholds – Proportion of Participants

Pause Rule	Event	Number or Percentage of Participants[a]
4	Any Grade 3 solicited local AR lasting more than 48 hours, within the 7-day postvaccination period	≥ 2[b] participants per group in the initial stage or ≥ 20% of participants in the expansion stage
5	Any Grade 3 solicited systemic AR lasting more than 48 hours (24 hours for fever) that cannot be reasonably attributed to a cause other than vaccination, within the 7-day postvaccination period	≥ 2[b] participants per group in the initial stage or ≥ 20% of participants in the expansion stage
6	Any severe unsolicited AE in a vaccination group that cannot be reasonably attributed to a cause other than vaccination<br>OR<br>Any Grade 3 laboratory abnormality[c] in a vaccination group that cannot be reasonably attributed to a cause other than vaccination	≥ 2[b] participants per group in the initial stage or ≥ 20% of participants in the expansion stage

Abbreviations: AE = adverse event; AR = adverse reaction; FDA = Food and Drug Administration; MedDRA = Medical Dictionary for Regulatory Activities; US = United States.

a The rate of AEs and laboratory abnormalities will be computed based on the number of exposed participants who have provided safety data (ie, have completed a postdosing visit for assessment of safety).

b For the first 9 participants in each vaccination group, the pause rule will be considered to be met if 2 of the first 9 participants experience the same solicited AR or the same MedDRA preferred term unsolicited AE or laboratory abnormality. The calculation for ≥ 20% of participants in the expansion stage includes participants in the initial stage and the expansion stage as the total number of participants in the denominator.

c Grading of laboratory parameters will be based on the US FDA Guidance for Industry "Toxicity grading scale for healthy adult and adolescent volunteers enrolled in preventive vaccine clinical trials" (DHHS 2007b).

If a pause is triggered in the study, each participant's study site visits will continue until EoS. If a pause affects a participant's vaccination visit, the window for that participant's vaccination visit will be suspended until the pause is lifted and vaccination can resume. Once the pause is lifted, vaccination should be reinstated as soon as possible.

If a participant is in the screening period for more than 28 days as the result of a pause, the participant may be rescreened for study eligibility (and will receive a new screening number) as long as the participant continues to provide consent to participate in the study.

7.1.2. Pause Rules for the Phase 2 Northern Hemisphere and the Phase 2 Extension Parts of the Study

During the Phase 2 parts of the study, the occurrence of any of the events listed in Table 12 regardless of the vaccination group, will result in immediate suspension of dosing and enrollment

in the vaccination group and at higher dose levels. An unscheduled DSMB will be convened to assess specific data concerns and to make recommendations.

Table 12 Phase 2 Northern Hemisphere and Phase 2 Extension Pause Rule Criteria, Events, and Thresholds - Single Event

Pause Rule	Event	Number of Participants
1	Any SAE that cannot be reasonably attributed to a cause other than study vaccination	≥ 1
2	Any Grade 4 solicited local or systemic AR that cannot be reasonably attributed to a cause other than study vaccination	≥ 1
3	Any systemic immediate hypersensitivity reaction within 60 minutes after the study vaccination	≥ 1

Abbreviations: AR = adverse reaction; SAE = serious adverse event.

7.2. Criteria for Delay or Withholding of Study Vaccination

Body temperature must be measured at the dosing visit before vaccine administration. The following events constitute criteria for delay of injection, and if either of these events occur at the time scheduled for dosing, the participant may be injected at a later date within the time window specified in the SoE (Table 1, Table 2, and Table 3):

- Acute moderate or severe infection with or without fever at the time of dosing.
- Fever, defined as body temperature ≥ 38.0°C (100.4°F) at the time of dosing.
- Receipt of COVID-19 vaccination within 7 to 14 days of planned Day 1.

Participants with a minor illness without fever, as assessed by the Investigator, can be vaccinated. Participants with a fever ≥ 38.0°C (100.4°F) will be contacted within the time window acceptable for participation and reevaluated for eligibility. If the Investigator determines that the participant's health on the day of dosing temporarily precludes injection, the visit should be rescheduled within the allowed interval for that visit.

The Investigator, in consultation with the Sponsor's medical monitor, may withhold the IP injection if the participant meets any of the following criteria:

- Becomes pregnant.
- Develops symptoms or conditions listed in the exclusion criteria.
- Experiences a clinically significant change in clinical laboratory test results, vital sign measurements, or general condition that, in the judgment of the Investigator, requires withholding of vaccine.

The reason(s) for withholding the injection will be recorded in the eCRF.

If a participant takes a prohibited drug therapy, the Investigator could delay the IP injection within the visit window or withhold the IP injection based on a joint decision of the Investigator and the CRO's medical monitor (Section 6.5.3).

7.3. Participant Discontinuation/Withdrawal From the Study

Participants who withdraw or are withdrawn from the study will not be replaced unless otherwise stated in the protocol.

A "withdrawal" from the study refers to a situation wherein a participant does not return for the final visit planned in the protocol.

Participants can withdraw consent and withdraw from the study at any time, for any reason, without prejudice to further treatment the participant may need to receive. The Investigator will request that the participant complete all study procedures pending at the time of withdrawal.

If a participant desires to withdraw from the study because of an AE, the Investigator will attempt to obtain agreement to follow-up with the participant until the event is considered resolved or stable and will then complete the EoS section of the eCRF.

Information related to the withdrawal will be documented in the eCRF. The Investigator will document whether the decision to withdraw a participant from the study was made by the participant or by the Investigator, as well as which of the following possible reasons was responsible for withdrawal:

- AE (specify)
- SAE (specify)
- Solicited AR or reactogenicity event (specify)
- Death
- Lost to follow-up (LTFU)
- Physician decision (specify)
- Pregnancy
- Protocol deviation
- Study terminated by Sponsor
- Withdrawal of consent by participant (specify)
- Other (specify)

Participants who are withdrawn from the study because of AEs (including SAEs, solicited ARs, or reactogenicity events) must be clearly distinguished from participants who are withdrawn for other reasons. Investigators will follow-up with participants who are withdrawn from the study as a result of an AE, SAE, solicited AR, or reactogenicity event until resolution of the event.

A participant withdrawing from the study may request destruction of any samples taken and not tested, and the Investigator must document this in the site study records.

If the participant withdraws consent for disclosure of future information, the Sponsor may retain and continue to use any data collected before such a withdrawal of consent (Section 11.1.6).

The Sponsor will continue to retain and use all research results that have already been collected for the study evaluation, unless the participant has requested destruction of these samples. All biological samples that have already been collected may be retained and analyzed at a later date (or as permitted by local regulations).

7.4. Lost to Follow-Up

A participant will be considered LTFU if he or she repeatedly fails to return for scheduled visits without stating an intention to withdraw consent and is unable to be contacted by the study site. The following actions must be taken if a participant fails to return to the study site for a required study visit:

- The site must attempt to contact the participant and reschedule the missed visit as soon as possible, counsel the participant on the importance of maintaining the assigned visit schedule, and ascertain whether the participant wishes to and/or should continue in the study.
- Before a participant is deemed LTFU, the Investigator or designee must make every effort to regain contact with the participant (where possible, 3 telephone calls and, if necessary, a certified letter to the participant's last known mailing address or local equivalent methods). These contact attempts (eg, dates of telephone calls and registered letters) should be documented in the participant's medical record.
- A participant who continues to be unreachable or continues to be noncompliant with study visits or procedures will be considered to have withdrawn from the study.
- A participant should not be considered LTFU until due diligence has been completed.

8. STUDY ASSESSMENTS AND PROCEDURES

Before performing any study procedures, all potential participants will sign an informed consent form (ICF; Section 11.1.6). Participants will undergo study procedures at the time points specified in the SoE (Table 1, Table 2, and Table 3). A participant can also be seen for an unscheduled visit at any time during the study. Reasons for an unscheduled visit may include, but are not limited to, reactogenicity issues, symptoms of potential ILI, and/or COVID-19, or new or ongoing AEs. The site also has the discretion to make reminder telephone calls or send text messages to inform the participant about visits, review eDiary requirements, or follow-up on ongoing or outstanding issues.

In accordance with "FDA Guidance on Conduct of Clinical Trials of Medical Products during COVID-19 Public Health Emergency" (DHHS 2020), Investigators may convert study site visits to home visits or telemedicine visits with the approval of the Sponsor. Such action should be taken to protect the safety and well-being of participants and study site staff or to comply with state or municipal mandates.

General considerations for study assessments and procedures include the following:

- Protocol waivers or exemptions are not allowed. The study procedures and their timing must be followed as presented in the SoE (Table 1, Table 2, and Table 3). Adherence to the study design requirements is essential and required for study conduct.
- Immediate safety concerns should be discussed with the Sponsor immediately upon occurrence or awareness to determine if the participant should continue participation in the study.
- All screening evaluations must be completed and reviewed to confirm that potential participants meet all eligibility criteria. The Investigator will maintain a screening log to record details of all participants screened and to confirm eligibility or record reasons for screening failure, as applicable.
- The Screening Visit and Day 1 Visit cannot be completed on the same day for the Phase 1/2 part but may be completed on the same day for the Phase 2 NH and Phase 2 Extension parts. Additionally, the Screening Visit may be performed over multiple visits if within the 28-day screening window.

8.1. Safety Assessments and Procedures

Safety assessments will include monitoring and recording of the following for each participant, according to the SoE (Table 1, Table 2, and Table 3):

- Solicited local and systemic ARs (Section 8.4.3) that occur during the 7 days following vaccine administration (ie, the day of injection and 6 subsequent days). Solicited ARs will be recorded daily using eDiaries (Section 8.1.1).
- Unsolicited AEs observed or reported during the 28 days following vaccine administration (ie, the day of injection and 27 subsequent days). Unsolicited AEs are defined in Section 8.4.1.
- AEs leading to discontinuation from study participation from Day 1 through Day 181/EoS or withdrawal from the study.
- MAAEs from Day 1 through Day 181/EoS or withdrawal from the study.
- SAEs and AESIs from Day 1 through Day 181/EoS or withdrawal from the study.
- Results of safety laboratory tests (Phase 1/2 only; Section 8.1.3).
- Vital sign measurements (Section 8.1.4).
- Physical examination findings (Section 8.1.5).
- Assessments for causes of respiratory infections as needed from Day 1 through Day 181/EoS in the Phase 1/2 and Phase 2 Extension parts of the study or withdrawal from the study (Section 8.1.6). Active and passive surveillance for ILI (and assessments for causes of respiratory infections as needed) will be conducted from Day 1 through Day 181/EoS in the Phase 2 NH part of the study.
- Details of all pregnancies in female participants will be collected after the study vaccination on Day 1 and until the end of their participation in the study (Section 8.4.8).
- Optional blood sample collection on Day 4 in the Phase 2 Extension part.

8.1.1. Use of Electronic Diaries

At the time of consent, the participants must confirm they will be willing to complete an eDiary using either an application downloaded to their own device or using a device that is provided at the time of enrollment. Before enrollment on Day 1, the participants will be instructed to download the eDiary application or will be provided an eDiary device to record solicited ARs (Section 8.4.3). Participants in the Phase 2 NH part of the study will also use the eDiary to record any ILI symptoms

(as part of active and passive surveillance) that they experience after vaccination from Day 1 through Day 181/EoS.

Participants will be instructed on Day 1 on thermometer usage to measure body temperature, ruler usage to measure injection site erythema and swelling/induration (hardness), and self-assessment for localized axillary swelling or tenderness on the same side as the injection arm.

Participants will record data into the eDiary starting approximately 1 hour after injection under supervision of the study site staff to ensure successful entry of assessments. The study site staff will perform any retraining as necessary. Participants will continue to record data in the eDiary after they leave the study site, preferably in the evening and at the same time each day, on the day of injection, and for 6 days following injection.

Participants will record the following data in the eDiary:

- Solicited local and systemic reactogenicity ARs, as defined in Section 8.4.3, that occur on the day of IP administration and during the 7 days after the IP administration (ie, the day of injection and 6 subsequent days). Any solicited AR that continues beyond Day 7 will be reported in the eDiary until it is no longer reported but only until 28 days after vaccination. Adverse reactions recorded in the eDiary beyond Day 7 should be reviewed by the study site staff either during the next scheduled telephone call or at the next study site visit.
- Passive and active surveillance of ILI (Phase 2 NH part only; Section 8.1.6). Participants will record symptoms of ILI that they may experience anytime from Day 1 through Day 181/EoS as part of the passive ILI surveillance. For active ILI surveillance, participants will be instructed to report whether ILI symptoms have been experienced, 3 to 4 times weekly from Day 1 through Day 29 and twice weekly from Day 30 through Day 181/EoS, via eDiary or telephone calls. If symptoms occur, participants will be directed to return to the study site as soon as possible, but no later than 72 hours after the onset of symptoms, for medical evaluation and an NP swab. Participants will be contacted by the study site, if they have missed reporting in the eDiary. If there is no response to an eDiary prompt for 2 consecutive entries, the study site staff will contact the participant by telephone (Section 8.1.6).
- Daily oral body temperature measurement should be performed at approximately the same time each day using the thermometer provided by the study site. If body temperature is taken more than once in a given day, only the highest temperature reading should be recorded.

- Measurements, as applicable, for solicited local ARs (injection site erythema and swelling/induration) will be performed using the ruler provided by the study site.
- Any medications taken to treat or prevent pain or fever on the day of injection or for the next 6 days.

The eDiary will be the only source document allowed for solicited systemic or local ARs (including body temperature measurements). Participants will be instructed to complete eDiary entries daily. Quantitative temperature recordings and measurement of any injection site erythema or swelling/induration reported on the following day may be excluded from the analyses of solicited ARs.

Study site staff will review eDiary data with participants at a visit 7 days after the injection.

8.1.1.1. Ancillary Supplies for Participant Use

Study sites will distribute Sponsor-provided oral thermometers and rulers for use by participants in assessing body temperature and injection site reactions, for recording solicited ARs in eDiaries. Based on availability, smartphone devices may be provided to those participants who do not have their own device to use for eDiary activities.

8.1.2. Safety Telephone Calls

A safety telephone call is a telephone call made to the participant by trained site personnel. This call will follow a script, which will facilitate the collection of relevant safety information. Safety telephone calls follow a schedule for each participant, as shown in the SoE (Table 1, Table 2, and Table 3). The participant will be interviewed according to the script about occurrence of AEs, MAAEs, SAEs, AESIs, AEs leading to withdrawal from study participation, concomitant medications associated with those events, and any nonstudy vaccinations (Section 8.4.9). All safety information collected from the telephone contact must be documented in source documents as described by the participant and not documented on the script used for the safety telephone contact. An unscheduled follow-up safety call may be triggered if an eDiary record results in identification of a relevant safety event.

8.1.3. Safety Laboratory Assessments

In the Phase 1/2 part of the study, planned blood sampling for safety laboratory assessments will occur as indicated in the SoE (Table 1). Tests will include WBC count, hemoglobin, platelets, ALT, AST, total bilirubin, alkaline phosphatase, and creatinine. Laboratory tests will be performed by the central laboratory, unless otherwise specified.

Pregnancy testing (applicable for all parts of the study) include the following:

- A point-of-care urine pregnancy test will be performed at the Screening Visit and before the study vaccination on Day 1. At any time, a pregnancy test either via blood or point-of-care urine can be performed, at the discretion of the Investigator.
- If not documented in a female participant's medical records, an FSH test may be performed at the Screening Visit, as necessary and at the discretion of the Investigator, to confirm postmenopausal status.

No scheduled safety laboratory assessments (except for pregnancy testing) will be conducted in the Phase 2 NH or Phase 2 Extension parts of the study.

8.1.4. Vital Sign Measurements

Vital sign measurements will include systolic and diastolic BP, heart rate, respiratory rate, and body temperature (preferred route is oral). The participant will be seated for at least 5 minutes before all measurements are taken. Vital signs will be measured at the time points indicated in the SoE (Table 1, Table 2, and Table 3). On the day of IP administration, vital sign measurements will be collected once before injection and approximately 1 hour after injection (before participants are discharged from the study site). Vital signs may be collected at other study visits in conjunction with a symptom-directed physical examination.

If any of the vital sign measurements meet the toxicity grading criteria for clinical abnormalities of Grade 3 or greater, the abnormal value and grade will be documented in the AE section of the eCRF (unless there is another known cause of the abnormality that would result in an AE classification). The Investigator will continue to monitor the participant with additional assessments until the vital sign value has reached the reference range, returns to the vital sign value at baseline, is considered stable, or until the Investigator determines that follow-up is no longer medically necessary.

Febrile participants on the day of IP administration (fever is defined as a body temperature ≥ 38.0°C/100.4°F) may be rescheduled within the relevant window periods. Afebrile participants with minor illnesses may be injected at the discretion of the Investigator.

When procedures overlap and are scheduled to occur at the same time point, the order of procedures should be vital sign measurements and then blood collection.

8.1.5. Physical Examinations

A full physical examination, including height and weight, will be performed at scheduled time points as indicated in the SoE (Table 1, Table 2, and Table 3). The full examination will include assessment of skin, head, ears, eyes, nose, throat, neck, thyroid, lungs, heart, cardiovascular

system, abdomen, lymph nodes, and musculoskeletal system and extremities. Any clinically significant finding identified during a study visit should be reported as an MAAE.

Symptom-directed physical examinations may be performed at other time points at the discretion of the Investigator. On the day of vaccination, prior to injection, both arms and associated lymph nodes should be examined, and any abnormalities should be documented.

Body mass index will be calculated at the Screening Visit only.

8.1.6. Assessments for Respiratory Viral Infections

Due to the ongoing COVID-19 pandemic, participants may get infected with SARS-CoV-2 or experience symptoms consistent with ILI during the study.

Phase 1/2

All participants will provide NP swab samples before the injection on Day 1 for assessment of asymptomatic infection with respiratory pathogens, including influenza virus and SARS-CoV-2, as influenza or COVID-19 symptoms may confound reactogenicity assessment (CDC 2020b).

A study site visit or a consultation will be arranged within 24 hours or as soon as possible to collect an NP swab sample if a participant experiences symptoms consistent with the CDC definition of ILI (see Section 8.4.6 for definition) and/or COVID-19 during the course of the study. The NP swab sample will be tested by multiplex RT-PCR for respiratory pathogens, including influenza viruses and SARS-CoV-2. Additionally, clinical information may be collected to evaluate the severity of the clinical case. All findings will be recorded in the eCRF.

If scheduled, a study site illness visit may include additional assessments such as medical history, physical examination, and blood sampling for clinical laboratory testing. Radiologic imaging studies may be conducted. Blood samples will be collected for potential future immunologic assessment of influenza and/or SARS-CoV-2 infection.

If participants are confirmed to have SARS-CoV-2 infection during the study and are asymptomatic, the Investigator will notify the participant's primary care physician and local health authority, per local regulations. If the participant had known exposure to COVID-19 (eg, exposure to someone with a confirmed case of COVID-19) prior to Day 1, it will be captured on the COVID-19 exposure form, and the IP administration for that participant will be delayed for at least 14 days.

All cases of RT-PCR-confirmed ILI and/or COVID-19 should be captured as MAAEs (unless the definition for an SAE is met) along with relevant concomitant medications and details about severity, seriousness, and outcome.

Phase 2 Northern Hemisphere

Participants in the Phase 2 NH part who manifest protocol-defined ILI (see Section 8.4.6 for definition) between Day 1 through Day 29 will be evaluated by real-time RT-PCR testing of an NP specimen for influenza (and other respiratory pathogens).

Both active and passive surveillance for ILI (see Section 8.4.6 for ILI definitions) will be conducted between Day 1 through Day 181/EoS. For active surveillance, participants will be instructed to report whether ILI symptoms have been experienced, 3 to 4 times weekly from Day 1 through Day 29, and twice weekly from Day 30 through Day 181/EoS, via eDiary or telephone calls.

If symptoms occur, participants will be directed to return to the study site as soon as possible, but no later than 72 hours after the onset of symptoms, for medical evaluation and an NP swab. Participants will be contacted by the study site, if they have missed reporting in the eDiary. If there is no response to an eDiary prompt for 2 consecutive entries, the study site staff will contact the study participant by telephone. For passive surveillance, participants will be instructed to report symptoms of ILI any time from Day 1 through Day 181/EoS.

Participants who manifest protocol-defined ILI (see Section 8.4.6 for definition) will have a study site visit or a consultation (unscheduled study visit) arranged within 72 hours after the onset of symptoms to collect an NP swab sample if participants experience symptoms consistent with the US CDC definition of ILI and/or COVID-19 during the course of the study.

Phase 2 Extension

For the Phase 2 Extension part, a study site visit or a consultation (unscheduled study visit) will be arranged within 72 hours after the onset of symptoms to collect an NP swab sample if participants experience symptoms consistent with the US CDC definition of ILI and/or COVID-19 during the course of the study.

8.2. Immunogenicity Assessments

Blood samples for immunogenicity assessments will be collected at the time points indicated in the SoE (Table 1, Table 2, and Table 3). The following assessments are planned:

- Serum antibody level as measured by HAI assay (primary, secondary, and exploratory endpoints).
- Serum neutralizing antibody level as measured by microneutralization assay or similar method (secondary [Phase 1/2 part only] and exploratory endpoints).

[redacted]

Sample aliquots will be designed to ensure that backup samples are available and that vial volumes are likely to be adequate for future testing needs. The actual time and date of each sample collected will be recorded in the eCRF, and unique sample identification will be utilized to maintain the blind at the laboratory at all times and to allow for automated sample tracking and housing. Handling and preparation of the samples for analysis, as well as shipping and storage requirements, will be provided in a separate study manual.

Measurement of antibody levels will be performed in a laboratory designated by the Sponsor.

According to the ICF (Section 11.1.6), serum from immunogenicity testing may be used for future research, which may be performed at the discretion of the Sponsor to further characterize the immune response to influenza viruses, additional assay development, and the immune response across influenza viruses.

The maximum amount of blood collected from each participant over the duration of the study, including any extra assessments that may be required, will not exceed blood limits specified by local regulations. Repeat or unscheduled samples may be taken for safety reasons or for technical issues with the samples. Further details are provided in both the ICF and Laboratory Reference Manual.

8.3. Efficacy Assessments

While the study will not be powered for efficacy assessments, [redacted] in the Phase 1/2, Phase 2 NH, and Phase 2 Extension parts of the study. [redacted]

8.4. Safety Definitions and Related Procedures

8.4.1. Adverse Event

An AE is defined as any untoward medical occurrence associated with the use of a drug/vaccine in humans, whether or not considered related to the drug/vaccine.

Events Meeting the Adverse Event Definition

- Exacerbation of a chronic or intermittent pre-existing condition, including an increase in frequency and/or intensity of the condition, after the IP injection.
- New conditions detected or diagnosed after the IP injection even though they may have been present before the start of the study.

Events NOT Meeting the Adverse Event Definition

- Procedures planned before study entry (eg, hospitalization for preplanned surgical procedure).
- Medical or surgical procedure (eg, endoscopy, appendectomy): the condition that leads to the procedure should be the AE.
- Situations in which an untoward medical occurrence did not occur (social and/or convenience admission to a hospital).

An AR is any AE for which there is a reasonable possibility that the vaccine caused the AE (Section 8.4.3). For the purposes of IND safety reporting, "reasonable possibility" means that there is evidence to suggest a causal relationship between the vaccine and the AE.

An unsolicited AE is any AE reported by the participant that is not specified as a solicited AR in the protocol or is specified as a solicited AR but starts outside the protocol-defined period for reporting solicited ARs (ie, 7 days after vaccine administration).

8.4.2. Serious Adverse Events

An AE (including an AR) is considered an SAE if, in the view of either the Investigator or Sponsor, it results in any of the following outcomes:

- **Death**
  A death that occurs during the study or that comes to the attention of the Investigator during the protocol-defined follow-up period must be reported to the Sponsor, whether or not it is considered related to the IP.
- **Is life-threatening**
  An AE is considered life-threatening if, in the view of either the Investigator or the Sponsor, its occurrence places the participant at immediate risk of death. It does not include an AE that, had it occurred in a more severe form, might have caused death.
- **Inpatient hospitalization or prolongation of existing hospitalization**
  In general, inpatient hospitalization indicates the participant was admitted to the hospital or emergency ward for at least 1 overnight stay for observation and/or

treatment that would not have been appropriate in the physician's office or outpatient setting. The hospital or emergency ward admission should be considered an SAE regardless of whether opinions differ as to the necessity of the admission. Complications that occur during inpatient hospitalization will be recorded as AEs; however, if a complication/AE prolongs hospitalization or otherwise fulfills SAE criteria, the complication/AE will be recorded as a separate SAE.

- **Persistent or significant incapacity or substantial disruption of the ability to conduct normal life functions**
  This definition is not intended to include experiences of relatively minor medical significance such as uncomplicated headache, nausea/vomiting, diarrhea, influenza, and accidental trauma (eg, sprained ankle) that may interfere with or prevent everyday life functions but do not constitute a substantial disruption.
- **Congenital anomaly or birth defect**
- **Medically important event**
  Medical judgment should be exercised in deciding whether SAE reporting is appropriate in other situations, such as important medical events that may not be immediately life-threatening or result in death or hospitalization but may jeopardize the participant or require medical or surgical intervention to prevent 1 of the other outcomes listed in the above definition. These events should usually be considered serious. Examples of such medical events include allergic bronchospasm requiring intensive treatment in an emergency room or at home, blood dyscrasias, or convulsions that do not result in inpatient hospitalization, or the development of drug dependency or drug abuse.

8.4.3. Solicited Adverse Reactions

The term "reactogenicity" refers to the occurrence and intensity of selected signs and symptoms (ARs) occurring after the IP injection. The eDiary will solicit daily participant reporting of ARs using a structured checklist (Section 8.1.1). Participants will record such occurrences in an eDiary on the day of injection and for the 6 days after the day of injection.

Severity grading of reactogenicity will occur automatically based on participant entries into the eDiary according to the grading scales presented in Table 13, which are modified from the toxicity grading scale for healthy adult and adolescent volunteers enrolled in preventive vaccine clinical trials (DHHS 2007b).

If a participant reported a solicited AR during the solicited period and did not record the event in the eDiary, the event should be recorded on the Reactogenicity page of the eCRF.

If the event starts during the solicited period, but continues beyond 7 days after dosing, the participant should notify the site to provide an end date to close out the event on the Reactogenicity page of the eCRF.

If the participant reported an event after the solicited period (ie, after Day 7), it should be recorded as an AE on the AE page of the eCRF.

All solicited ARs (local and systemic) will be considered causally related to study vaccination.

Table 13 Solicited Adverse Reactions and Grades

Reaction	Grade 1	Grade 2	Grade 3	Grade 4
Injection site pain	Does not interfere with activity	Repeated use of over-the-counter pain reliever > 24 hours or interferes with activity	Any use of prescription pain reliever or prevents daily activity	Requires emergency room visit or hospitalization
Injection site erythema (redness)	25 - 50 mm/ 2.5 - 5 cm	51 - 100 mm/ 5.1 - 10 cm	> 100 mm/ > 10 cm	Necrosis or exfoliative dermatitis
Injection site swelling/ induration (hardness)	25 - 50 mm/ 2.5 - 5 cm	51 - 100 mm/ 5.1 - 10 cm	> 100 mm/ > 10 cm	Necrosis
Axillary (underarm) swelling or tenderness ipsilateral to the side of injection	No interference with activity	Repeated use of over-the-counter (nonnarcotic) pain reliever > 24 hours or some interference with activity	Any use of prescription (narcotic) pain reliever or prevents daily activity	Emergency room visit or hospitalization
Headache	No interference with activity	Repeated use of over-the-counter pain reliever > 24 hours or some interference with activity	Significant; any use of prescription pain reliever or prevents daily activity	Requires emergency room visit or hospitalization
Fatigue	No interference with activity	Some interference with activity	Significant; prevents daily activity	Requires emergency room visit or hospitalization

Reaction	Grade 1	Grade 2	Grade 3	Grade 4
Myalgia (muscle aches all over body)	No interference with activity	Some interference with activity	Significant; prevents daily activity	Requires emergency room visit or hospitalization
Arthralgia (joint aches in several joints)	No interference with activity	Some interference with activity	Significant; prevents daily activity	Requires emergency room visit or hospitalization
Nausea/vomiting	No interference with activity or 1 or 2 episodes/24 hours	Some interference with activity or > 2 episodes/ 24 hours	Prevents daily activity, requires outpatient intravenous hydration	Requires emergency room visit or hospitalization for hypotensive shock
Chills	No interference with activity	Some interference with activity not requiring medical intervention	Prevents daily activity and requires medical intervention	Requires emergency room visit or hospitalization
Fever (oral)	38.0°C - 38.4°C<br>100.4°F - 101.1°F	38.5°C - 38.9°C<br>101.2°F - 102.0°F	39.0°C - 40.0°C<br>102.1°F - 104.0°F	> 40.0°C<br>> 104.0°F

Abbreviations: AE = adverse event; eCRF = electronic case report form,

Note: Events listed above but starting > 7 days post study injection will be recorded on the AE page of the eCRF. Causality for each event will be determined per assessment by the Investigator.

Any solicited AR that meets any of the following criteria must be entered into the participant's source document and must also be recorded by the study site staff in the solicited AR section of the participant's eCRF:

- Solicited local or systemic AR that results in a visit to a healthcare practitioner (HCP), to be recorded as an MAAE (Section 8.4.4).
- Solicited local or systemic AR leading to the participant withdrawing from the study or the participant being withdrawn from the study by the Investigator (AE leading to withdrawal).
- Solicited local or systemic AR lasting beyond 7 days after injection.
- Solicited local or systemic AR that otherwise meets the definition of an SAE.

8.4.4. Medically Attended Adverse Events

An MAAE is an AE that leads to an unscheduled visit to an HCP. This would include visits to a study site for unscheduled assessments (eg, abnormal laboratory follow-up, and/or COVID-19

[Section 8.1.6]) and visits to HCPs external to the study site (eg, urgent care, primary care physician). Investigators will review unsolicited AEs for the occurrence of any MAAEs. Unsolicited AEs will be recorded in the AE section of the eCRF.

All cases of RT-PCR-confirmed ILI and/or confirmed COVID-19 will be recorded as MAAEs.

8.4.5. Anaphylaxis

All suspected cases of anaphylaxis should be recorded as MAAEs and reported as an SAE, based on the criteria for a medically important event, unless the event meets other serious criteria. As an SAE, the event should be reported to the Sponsor or designee immediately and in all circumstances within 24 hours, per Section 8.4.13. The Investigator will submit any updated anaphylaxis case data to the Sponsor within 24 hours of it being available. For reporting purposes, a participant who displays signs or symptoms consistent with anaphylaxis (as described below) should be reported as a potential case of anaphylaxis. This is provided as general guidance for Investigators and is based on the Brighton Collaboration case definition (Rüggeberg et al 2007).

Anaphylaxis is an acute hypersensitivity reaction with multi-organ system involvement that can present as, or rapidly progress to, a severe life-threatening reaction. It may occur following exposure to allergens from a variety of sources.

Anaphylaxis is a clinical syndrome characterized by the following:

- Sudden onset AND
- Rapid progression of signs and symptoms AND
- Involves 2 or more organ systems, as follows:
  - **Skin/mucosal:** urticaria (hives), generalized erythema, angioedema, generalized pruritus with skin rash, generalized prickle sensation, red and itchy eyes.
  - **Cardiovascular:** measured hypotension, clinical diagnosis of uncompensated shock, loss of consciousness or decreased level of consciousness, evidence of reduced peripheral circulation.
  - **Respiratory:** bilateral wheeze (bronchospasm), difficulty breathing, stridor, upper airway swelling (lip, tongue, throat, uvula, or larynx), respiratory distress, persistent dry cough, hoarse voice, sensation of throat closure, sneezing, rhinorrhea.
  - **Gastrointestinal:** diarrhea, abdominal pain, nausea, vomiting.

8.4.6. Influenza-Like Illness

A protocol-defined ILI is determined by the occurrence of at least 1 respiratory illness symptom concurrently with at least 1 systemic symptom, or the occurrence of any 2 or more respiratory symptoms.

Respiratory Symptoms	Systemic Symptoms
1. Sore throat<br>2. Cough/rhinorrhea/nasal congestion (≥ 1 of the 3 symptoms count as 1 respiratory symptom)<br>3. Sputum production<br>4. Wheezing<br>5. Difficulty breathing	1. Body temperature > 37.2°C (> 99°F)<br>2. Chills<br>3. Tiredness<br>4. Headache<br>5. Myalgia<br>6. Nausea/Vomiting<br>7. Diarrhea

A CDC-defined ILI is defined as body temperature ≥ 37.8°C (100°F) accompanied by cough and/or sore throat.

An RT-PCR-confirmed ILI is defined as a positive influenza result by RT-PCR done at any setting during the study period.

8.4.7. Adverse Events of Special Interest

An AESI is an AE (serious or nonserious) of scientific and medical concern specific to the Sponsor's product or program, for which ongoing monitoring and immediate notification by the Investigator to the Sponsor are required. Such events may require further investigation to characterize and understand them.

Adverse events of special interest for this protocol are listed in Appendix 3 (Section 11.3).

All AESIs will be collected through the entire study period and must be reported to the Sponsor or designee immediately and in all circumstances within 24 hours of becoming aware of the event via the electronic data capture (EDC) system. If a site receives a report of a new AESI from a participant or receives updated information on a previously reported AESI at a time after the eCRF has been taken offline, then the site can report this information on a paper AESI form using the SAE Mailbox.

Myocarditis/Pericarditis

A case of suspected, probable, or confirmed myocarditis, pericarditis, or myopericarditis should be reported as an AESI, even if it does not meet criteria per the CDC case definition. The event

should also be reported as an SAE if it meets seriousness criteria (Section 8.4.2). The CDC case definition is provided in Section 11.4 as guidance.

8.4.8. Recording and Follow-Up of Pregnancy

Female participants who have a positive pregnancy test at screening should not be enrolled; participants who have a positive pregnancy test at Day 1 should not receive the IP.

Details of all pregnancies in female participants will be collected after the study vaccination on Day 1 and until the end of their participation in the study. If a pregnancy is reported, the Investigator should inform the Sponsor within 24 hours of learning of the pregnancy and should follow the procedures outlined in this section. Abnormal pregnancy outcomes (eg, spontaneous abortion, fetal death, stillbirth, congenital anomalies, ectopic pregnancy) are considered SAEs.

Pregnancies occurring in participants after enrollment must be reported to Sponsor or designee within 24 hours of the site learning of its occurrence, using the SAE Mailbox, the SAE Hotline, or the SAE Fax line (Section 8.4.13). If the participant agrees to submit this information, the pregnancy must be followed to determine the outcome, including spontaneous or voluntary termination, details of the birth, and the presence or absence of any birth defects, congenital abnormalities, or maternal and/or newborn complications. This follow-up should occur even if intended duration of the safety follow-up for the study has ended. Pregnancy report forms will be distributed to the study site to be used for this purpose. The Investigator must immediately (within 24 hours of awareness) report to the Sponsor any pregnancy resulting in an abnormal outcome according to the procedures described for SAEs.

8.4.9. Eliciting and Documenting Adverse Events

The Investigator is responsible for ensuring that all AEs and SAEs are recorded in the eCRF and reported to the Sponsor.

Solicited ARs will be collected from Day 1 through 7 days after the vaccine dose. Other (unsolicited) AEs will be collected from Day 1 through 28 days after the vaccine dose.

The MAAEs, SAEs, and AESIs will be collected from participants as specified in the SoE (Table 1, Table 2, and Table 3) until the end of their participation in the study. Any AEs occurring before receipt of the IP will be analyzed separately from AEs.

At every study site visit or telephone contact, participants will be asked a standard question to elicit any medically related changes in their well-being (including respiratory viral infection symptoms) according to the scripts provided. Participants will also be asked if they have been hospitalized, had any accidents, used any new medications, changed concomitant medication regimens (both prescription and over-the-counter medications), or had any nonstudy vaccinations.

In addition to participant observations, physical examination findings and other documents relevant to participant safety classified as an AE will be documented in the AE section of the eCRF.

After the initial AE/SAE report, the Investigator is required to proactively follow each participant at subsequent visits/contacts. All AEs and SAEs will be treated as medically appropriate and followed until resolution, stabilization, the event is otherwise explained, or the participant is LTFU (as defined in Section 7.4).

8.4.10. Assessment of Intensity

An event is defined as “serious” when it meets at least 1 of the predefined outcomes as described in the definition of an SAE (Section 8.4.2), NOT when it is rated as severe.

The severity (or intensity) of an AR or AE refers to the extent to which it affects the participant’s daily activities. The toxicity grading scale for healthy adult and adolescent volunteers enrolled in preventive vaccine clinical trials (DHHS 2007b) will be used to categorize local and systemic reactogenicity events (solicited ARs), clinical laboratory test results, and vital sign measurements observed during this study. Specific criteria for local and systemic reactogenicity events are presented in Section 8.4.3.

The determination of severity for all unsolicited AEs should be made by the Investigator based upon medical judgment and the definitions of severity as follows:

- Mild: These events do not interfere with the participant’s daily activities.
- Moderate: These events cause some interference with the participant’s daily activities and require limited or no medical intervention.
- Severe: These events prevent the participant’s daily activity and require intensive therapeutic intervention.

Study staff should elicit from the participant the impact of AEs on the participant’s activities of daily living to assess severity and document appropriately in the participant’s source documentation. Changes in the severity of an AE should be documented in the participant’s source documentation to allow an assessment of the duration of the event at each level of intensity to be performed. An AE characterized as intermittent requires documentation of onset and duration of each episode. An AE that fluctuates in severity during the course of the event is reported once in the eCRF at the highest severity observed.

8.4.11. Assessment of Causality

The Investigator’s assessment of an AE’s relationship to the IP is part of the documentation process but is not a factor in determining what is or is not reported in the study.

The Investigator will assess causality (ie, whether there is a reasonable possibility that the IP caused the event) for all AEs and SAEs. The relationship will be characterized using the following classification:

- **Not related:** There is not a reasonable possibility of a relationship to the IP. Participant did not receive the IP OR the temporal sequence of AE onset relative to administration of the IP is not reasonable OR the AE is more likely explained by another cause than the IP.
- **Related:** There is a reasonable possibility of a relationship to the IP. There is evidence of exposure to the IP. The temporal sequence of the AE onset relative to the administration of the IP is reasonable. The AE is more likely explained by the IP than by another cause.

8.4.12. Reporting Adverse Events

The Investigator is responsible for reporting all AEs that are observed or reported during the study regardless of their relationship to the IP or their clinical significance. If there is any doubt as to whether a clinical observation is an AE, the event should be reported.

All unsolicited AEs reported or observed during the study will be recorded in the AE section of the eCRF. Information to be collected includes type of event, time of onset, Investigator-specified assessment of severity (impact on activities of daily living) and relationship to the IP, time of resolution of the event, seriousness, any required treatment or evaluations, and outcome. The unsolicited AEs resulting from concurrent illnesses, reactions to concurrent illnesses, reactions to concurrent medications, or progression of disease states must also be reported. All AEs will be followed until they are resolved or stable or judged by the Investigator to be not clinically significant. The Medical Dictionary for Regulatory Activities (MedDRA) will be used to code all unsolicited AEs.

Any medical condition that is present at the time that the participant is screened but does not deteriorate should not be reported as an unsolicited AE. However, if it deteriorates at any time during the study, it should be recorded as an unsolicited AE.

8.4.13. Reporting Serious Adverse Events

Prompt notification by the Investigator to the Sponsor of an SAE is essential so that legal obligations and ethical responsibilities toward the safety of participants and the safety of a study intervention under clinical investigation are met.

Any AE considered serious by the Investigator or that meets SAE criteria (Section 8.4.2) must be reported to the Sponsor immediately (within 24 hours of becoming aware of the SAE) via the EDC

system. The Investigator will assess whether there is a reasonable possibility that the IP caused the SAE. The Sponsor will be responsible for notifying the relevant regulatory authorities of any SAE as outlined in 21 US CFR Parts 312 and 320. The Investigator is responsible for notifying the institutional review board (IRB) directly.

If the eCRF is unavailable at the time of the SAE, the following contact information is to be used for SAE reporting:

- SAE Mailbox: [redacted]
- SAE Hotline (USA): [redacted]
- SAE Fax Line (USA): [redacted]

Regulatory reporting requirements for SAEs are described in Section 8.4.18.

The Investigator and any qualified designees are responsible for detecting, documenting, and recording events that meet the definition of an AE, including SAEs, and remain responsible for following up AEs that are serious, considered related to the IP or study procedures, or that caused the participant to discontinue the study.

8.4.14. Reporting of Adverse Events of Special Interest

The following process for reporting an AESI ensures compliance with 21 CFR 312 and ICH GCP guidelines. After learning that a participant has experienced an AESI, the Investigator or designee is responsible for reporting the AESI to the Sponsor, regardless of relationship or expectedness, within 24 hours of becoming aware of the event. If the AESI meets the criteria for an SAE, the SAE reporting procedure should be followed.

8.4.15. Time Period and Frequency for Collecting Adverse Event and Serious Adverse Event Information

Medical occurrences that begin before the start of IP administration but after obtaining informed consent will be recorded in the Medical History/Current Medical Conditions section of the eCRF and not in the AE section; however, if the condition worsens at any time during the study, it will be recorded and reported as an AE.

Adverse events may be collected as follows:

- Observing the participant.
- Receiving an unsolicited complaint from the participant.
- Questioning the participant in an unbiased and nonleading manner.

Solicited ARs will be collected from the day of injection through 6 days after the injection. Other (unsolicited) AEs will be collected from the day of injection through 28 days after the injection.

Serious AEs will be collected from the start of IP administration until the last day of study participation.

All SAEs will be recorded and reported to the Sponsor or designee immediately and under no circumstance should this exceed 24 hours of becoming aware of the event via the EDC system. If a site receives a report of a new SAE from a study participant or receives updated data on a previously reported SAE and the eCRF has been taken offline, then the site can report this information on a paper SAE form using the SAE Mailbox, the SAE Hotline, or the SAE Fax line (Section 8.4.13).

An abnormal value or result from a clinical or laboratory evaluation can also indicate an AE if it is determined by the Investigator to be clinically significant (eg, meets any serious criteria). If this is the case, it must be recorded in the source document and as an AE on the appropriate AE form(s). The evaluation that produced the value or result should be repeated until that value or result returns to normal or is stabilized and the participant's safety is not at risk.

Investigators are not obligated to actively seek AEs or SAEs after EoS. However, if the Investigator learns of any SAE (including a death) at any time after a participant has withdrawn from or completed the study and the Investigator considers the event to be reasonably related to the IP or study participation, the Investigator must promptly notify the Sponsor.

Participants who develop ILI will be followed through 30 days from the onset of ILI even if Day 30 is beyond Day 181/EoS.

8.4.16. Method of Detecting Adverse Events and Serious Adverse Events

Electronic diaries have specifically been designed for this study by the Sponsor. The diaries will include prelisted AEs (solicited ARs) and intensity scales; they will also include blank space for the recording of information on other AEs (unsolicited AEs) and concomitant medications/vaccinations.

The Investigator is responsible for the documentation of AEs regardless of the vaccination group or suspected causal relationship to the IP. For all AEs, the Investigator must pursue and obtain information adequate to determine the outcome of the AE and to assess whether the AE meets the criteria for classification as an SAE, which requires immediate notification to the Sponsor or its designated representative.

Care will be taken not to introduce bias when detecting AEs and/or SAEs. Open-ended and nonleading verbal questioning of the participant is the preferred method to inquire about AE occurrences.

8.4.17. Follow-Up of Adverse Events and Serious Adverse Events

After the initial AE/SAE report, the Investigator is required to proactively follow each participant at subsequent visits and contacts.

All AEs and SAEs will be treated as medically appropriate and followed until resolution, stabilization, the event is otherwise explained, or the participant is LTFU, as defined in Section 7.4.

8.4.18. Regulatory Reporting Requirements for Serious Adverse Events

Prompt notification by the Investigator to the Sponsor of an SAE is essential so that legal obligations and ethical responsibilities towards the safety of participants and the safety of a study intervention under clinical investigation are met.

The Sponsor has a legal responsibility to notify both the local regulatory authority and other regulatory agencies about the safety of a study intervention under clinical investigation. The Sponsor will comply with country-specific regulatory requirements relating to safety reporting to the regulatory authority, IRBs, and Investigators.

Investigator safety reports must be prepared for suspected unexpected serious ARs according to local regulatory requirements and Sponsor policy and forwarded to Investigators as necessary.

An Investigator who receives an investigator safety report describing an SAE or other specific safety information (eg, summary or listing of SAEs) from the Sponsor will review and then file it along with the IB and will notify the IRB, if appropriate according to local requirements.

8.5. Safety Monitoring

The CRO's medical monitor, the Sponsor's medical monitor, and the individual Site Investigators will monitor safety throughout the study. The roles of the IST and DSMB are outlined in Section 11.1.11.

8.6. Treatment of Overdose

Because the IP is to be administered by an HCP, it is unlikely that an overdose will occur. Dose deviations will be tracked as protocol deviations (Section 11.1.8).

8.7. Pharmacokinetics

Pharmacokinetic parameters are not evaluated in this study.

8.8. Pharmacodynamics

Pharmacodynamic parameters are not evaluated in this study.

8.9. Biomarkers

Immunogenicity assessments are described in Section 8.2. Biomarkers are not evaluated in the Phase 1/2 part of the study outside of the described immunogenicity assessments. Biomarker assessments (to be determined) will be evaluated in the Phase 2 NH and Phase 2 Extension parts of the study, which may include genomic and transcriptomic studies.

8.10. Health Economics

Health economics are not evaluated in this study.

9. STATISTICAL ANALYSIS PLAN

This section summarizes the planned statistical analysis strategy and procedures for the study. The details of statistical analysis will be provided in the statistical analysis plan (SAP), which will be finalized before the clinical database lock for the study. If changes are made to primary and/or key secondary objectives or the related statistical methods after the study has begun but prior to any data unblinding, then the protocol will be amended (consistent with ICH Guideline E9). Changes to other secondary or exploratory analyses made after the protocol has been finalized, along with an explanation as to when and why they occurred, will be listed in the SAP or clinical study report (CSR) for the study. Ad hoc exploratory analyses, if any, will be clearly identified in the CSR.

9.1. Blinding and Responsibility for Analyses

This is an observer-blind study. The Investigator, study staff, study participants, site monitors, and Sponsor personnel (or its designees) will be blinded to the IP administered until the study database is locked and unblinded, with the following exceptions:

- Unblinded personnel (of limited number) will be assigned to vaccine accountability procedures and will prepare the IP for all participants. These personnel will have no study functions other than IP management, documentation, accountability, preparation, and administration. They will not be involved in participant evaluations and will not reveal the identity of the IP to either the participant or the blinded study site personnel involved in the conduct of the study unless this information is necessary in the case of an emergency.
- Unblinded medically qualified study site personnel will administer the IP. They will not be involved in assessments of any study endpoints.
- Unblinded site monitors, not involved in other aspects of monitoring, will be assigned as the IP accountability monitors. They will have responsibilities to ensure that sites are following all proper IP accountability, preparation, and administration procedures.
- An independent unblinded statistical and programming team will perform the preplanned IA (Section 9.6). Sponsor team members will be prespecified to be unblinded to the IA results and will not communicate the results to the blinded Investigators, study site staff, clinical monitors, or participants.
- The DSMB will review the IA data to safeguard the interests of clinical study participants and to help ensure the integrity of the study. The DSMB will review unblinded IA results provided by the independent unblinded statistician. The DSMB will also review unblinded statistical outputs for ad hoc safety reviews triggered by

pause rules, should this occur. Section 11.1.11 provides additional information on DSMB and safety review.

The dosing assignment will be concealed by having the unblinded pharmacy personnel prepare the IP in a secure location that is not accessible or visible to other study staff. An opaque sleeve over the syringe used for injection will maintain the blind at the time of injection, as the doses containing mRNA-1010 will look different from those of placebo/active comparator. Only delegated unblinded study site staff will conduct the injection procedure. Once the injection is completed, only the blinded study staff will perform further assessments and interact with the participants. Access to the randomization code will be strictly controlled at the pharmacy.

9.1.1. Breaking the Blind

Except in the case of medical necessity, a participant's vaccine assignment should not be unblinded without the approval of the Sponsor. If a participant becomes seriously ill or pregnant during the study, the blind will be broken only if knowledge of the vaccine assignment will affect that participant's clinical management. In the event of a medical emergency requiring identification of individual vaccine assignment, the Investigator will make every attempt to contact the CRO's medical monitor, preferably via electronic protocol inquiry platform, to explain the need for unblinding within 24 hours of opening the code. The Investigator will be responsible for documenting the time, date, reason for unblinding, and the names of the personnel involved. The Investigator (or designee) will have access to unblind participants within interactive response technology (IRT). All unblinding instances will be tracked via an audit trail in IRT and documented in the final study report.

In addition to the aforementioned situations where the blind may be broken, the data will also be unblinded to a statistical team at specified time point(s) for analysis as outlined in Section 9.6.

9.2. Statistical Hypotheses

No formal hypotheses will be tested.

9.3. Sample Size Determination

The sample size for this study is not driven by statistical assumptions for formal hypothesis testing. The number of proposed participants is considered sufficient to provide a descriptive summary of the safety and immunogenicity of different dose levels of mRNA-1010.

Phase 1/2

Approximately 180 participants will be randomly assigned in this part of the study, with approximately 45 participants in each vaccination group. Details regarding the number of participants in each vaccination group and the randomization ratio are presented in Section 6.2.1.

With 45 participants receiving an investigational vaccine, there is a probability of approximately 90% to observe at least 1 participant with an AE, if the true incidence rate of the AE is 5%; if the true incidence rate is 10%, then the probability to observe it will be approximately 99%.

Phase 2 Northern Hemisphere

Approximately 500 participants will be randomly assigned in a 3:3:3:1 ratio to the mRNA-1010 25, 50, 100 µg groups, or the Active Comparator Group. A total of 450 participants will receive the mRNA-1010 vaccine (150 participants at each dose level). A sample size of 150 participants at each dose level has at least a 95% probability to observe at least 1 participant with an AE, if the true incidence rate is 2%; if the true incidence rate is 3%, then the probably to observe it will be 99%.

Phase 2 Extension

Approximately 200 participants will be randomly assigned in a 1:1:1:1 ratio to the mRNA-1010 [redacted] groups, or the Active Comparator Group. A total of 150 participants will receive the mRNA-1010 vaccine (50 participants at each dose level). With 50 participants receiving an investigational vaccine, there is a probability of approximately 92% to observe at least 1 participant with an AE, if the true incidence rate of the AE is 5%; if the true incidence rate is 10%, then the probability to observe it will be approximately 99%.

9.4. Analysis Sets

The analysis sets are described in Table 14.

Table 14 Analysis Sets

Set	Description
Randomization Set	The Randomization Set consists of all participants who are randomly assigned.
Full Analysis Set (FAS)[1]	The FAS consists of all randomly assigned participants who receive the IP.
Per-Protocol (PP) Set[2]	The PP Set consists of all participants in the FAS who comply with the injection schedule, comply with the timings of immunogenicity blood sampling to have a baseline and at least 1 postinjection assessment, do not have influenza infection at baseline through Day 29 (as documented by PCR testing), and have no major protocol deviations that impact the immune response.
Safety Set[3]	The Safety Set consists of all randomly assigned participants who receive the IP.
Solicited Safety Set[4]	The Solicited Safety Set consists of all participants in the Safety Set who contribute any solicited AR data.

Abbreviations: AR = adverse reaction; IP = investigational product; PCR = polymerase chain reaction; RT-PCR = reverse transcription polymerase chain reaction.

1 For the FAS, participants will be analyzed according to the group to which they were randomized.

2 The PP Set will be used as the primary analysis set for analyses of immunogenicity unless otherwise specified. Participants will be analyzed according to the group to which they were randomized.

3 The Safety Set will be used for all analyses of safety, except for the solicited ARs. Participants will be included in the vaccination group corresponding to what that they actually received.

4 The Solicited Safety Set will be used for the analyses of solicited ARs and participants will be included in the vaccination group corresponding to what that they actually received.

9.5. Statistical Methods

All analyses and data summaries/displays will be provided by vaccination group and overall.

9.5.1. Baseline Characteristics and Demographics

Demographic variables (eg, age, gender, race, ethnicity, height, weight, and BMI) and baseline characteristics will be summarized by vaccination group and overall. Summary statistics (mean and standard deviation for continuous variables, and number and percentage for categorical variables) will be provided.

9.5.2. Safety Analyses

All safety analyses will be based on the Safety Set, except summaries of solicited ARs, which will be based on the Solicited Safety Set. All safety analyses will be provided by vaccination group.

Participants will be included in the vaccination group corresponding to what they actually received.

Safety and reactogenicity will be assessed by clinical review of all relevant parameters, including solicited ARs (local and systemic ARs), unsolicited AEs (including any clinical safety laboratory abnormalities [Phase 1/2 part only]), treatment-related AEs, severe AEs, SAEs, AESIs, MAAEs, AEs leading to withdrawal from study participation, vital sign measurements, and physical examination findings.

The number and percentage of participants with any solicited local AR, solicited systemic AR, and solicited AR during the 7-day follow-up period after the injection will be summarized. A 2-sided 95% exact confidence interval (CI) using the Clopper-Pearson method will also be provided for the percentage of participants with any solicited AR.

The number and percentage of participants with unsolicited AEs, treatment-related AEs, severe AEs, SAEs, MAAEs, AESIs, and AEs leading to withdrawal from study participation will be summarized. Unsolicited AEs will be coded according to the MedDRA for AR terminology and presented by MedDRA system organ class and preferred term.

Solicited ARs will be coded according to the MedDRA for AR terminology. The toxicity grading scale for healthy adult and adolescent volunteers enrolled in preventive vaccine clinical trials will be used in this study with modification for rash, solicited ARs, and vital signs (DHHS 2007b). Unsolicited AEs will be presented by MedDRA system organ class and preferred term.

The number of events of unsolicited AEs/SAEs, AESIs, and MAAEs will be reported in summary tables accordingly. For all other safety parameters, descriptive summary statistics will be provided.

Assessment of safety laboratory tests will be done for the Phase 1/2 part of the study only. The number and percentage of participants who have chemistry and hematology results below or above the normal laboratory ranges will be tabulated by time point. For treatment-emergent safety laboratory test results, the raw values and change from baseline values will be summarized by vaccination group and visit at each time point.

For all parts of the study, vital sign results will be presented.

Table 15 summarizes the analysis strategy for safety parameters. For all other safety parameters, descriptive summary statistics will be provided. Further details will be described in the SAP.

Table 15 Analysis Strategy for Safety Parameters

Safety Endpoint	Number and Percentage of Participants, Number of Events	95% CI
Any solicited AR (overall and by local, systemic)	X	X
Any unsolicited AE	X	–
Any SAE	X	–
Any unsolicited MAAE	X	–
Any unsolicited AESI	X	-
Any unsolicited treatment-related AE	X	–
Any treatment-related SAE	X	–
Any unsolicited AE leading to withdrawal from study participation	X	–
Any severe unsolicited AE	X	–
Any treatment-related severe unsolicited AE	X	–

Abbreviations: AE = adverse event; AESI = adverse event of special interest; AR = adverse reaction; CI = confidence interval; MAAE = medically attended adverse event; SAE = serious adverse event.
Note: 95% CI using the Clopper-Pearson method. X = results will be provided.

9.5.3. Immunogenicity Analyses

Immunogenicity analyses in each part of the study will be reported based on the PP Set and provided by vaccination group, unless otherwise specified.

Phase 1/2

For the immunogenicity endpoints, geometric mean of specific antibody titers with corresponding 95% CI at each time point and geometric mean fold rise (GMFR) of specific antibody titers with corresponding 95% CI at each postbaseline time point over preinjection baseline at Day 1 will be provided by vaccination group. The 95% CIs will be calculated based on the t-distribution of the log 2-transformed values and then back transformed to the original scale. Descriptive summary statistics, including median, minimum, and maximum, will also be provided.

For calculation of geometric mean titers (GMTs), antibody titers reported as below the lower limit of quantification (LLOQ) will be replaced by 0.5 × LLOQ. Values that are greater than the upper limit of quantification (ULOQ) will be converted to the ULOQ.

Seroconversion rate from baseline will be provided with a 2-sided 95% CI using the Clopper-Pearson method at each postbaseline time point. Rate of seroconversion is defined as the proportion of participants with either a prevaccination HAI titer < 1:10 and a postvaccination HAI titer ≥ 1:40 or a prevaccination HAI titer ≥ 1:10 and a minimum 4-fold rise in postvaccination HAI antibody titer.

Further details will be described in the SAP.

Phase 2 Northern Hemisphere

Immune responses, as measured by GMT and seroconversion rate in the mRNA-1010 groups based on Day 29 HAI titers, will be compared with that of participants receiving active comparator for all 4 strains.

An analysis of covariance model will be carried out with HAI titers at Day 29 as a dependent variable and a group variable as the fixed variable. The GMT of the mRNA-1010 at Day 29 will be estimated by the geometric least square mean (GLSM) from the model. The geometric mean ratio (GMTr; ratio of GMT) will be estimated by the ratio of GLSM from the model. The corresponding 2-sided 95% CI will be provided to assess the difference in immune response between the mRNA-1010 compared with the active comparator at Day 29.

The number and percentage of participants with seroconversion due to vaccination will be provided with 2-sided 95% CI using the Clopper-Pearson method at Day 29. The seroconversion rate difference with 95% CI at Day 29 will be provided.

In addition, the GMT of HAI titers with corresponding 95% CI will be provided at each time point. The 95% CIs will be calculated based on the t-distribution of the log-transformed values and then back transformed to the original scale. The GMFR of HAI titers with corresponding 95% CI at each postbaseline time point over baseline will be provided. Descriptive summary statistics, including median, minimum, and maximum, will also be provided.

For summarizations of GMTs, antibody titers reported as LLOQ will be replaced by 0.5 × LLOQ. Values that are greater than ULOQ will be converted to the ULOQ.

Rate of seroconversion is defined as (1) if LLOQ is 1:10, the proportion of participants with either a prevaccination HAI titer < 1:10 and a postvaccination HAI titer ≥ 1:40 or a prevaccination HAI titer ≥ 1:10 and a minimum 4-fold rise in the postvaccination HAI antibody titer; or (2) if LLOQ is greater than 1:10, the proportion of participants with either a prevaccination HAI titer < LLOQ and a postvaccination HAI ≥ 4 times of LLOQ, or a prevaccination HAI ≥ LLOQ and a minimum 4-fold rise in the postvaccination HAI .

The HAI titers at Day 29, GMTr at Day 29, and the difference in seroconversion rate at Day 29 may also be performed with the Full Analysis Set as supplementary analyses.

Phase 2 Extension

Immune responses, as measured by GMT and seroconversion rate in the mRNA-1010 groups based on Day 29 HAI titers, will be compared with that of participants receiving active comparator for all 4 strains.

The number and percentage of participants with seroconversion due to vaccination will be provided with 2-sided 95% CI using the Clopper-Pearson method at Day 29. The seroconversion rate difference with 95% CI at Day 29 will be provided.

For summarizations of GMTs, antibody titers reported as LLOQ will be replaced by 0.5 × LLOQ. Values that are greater than ULOQ will be converted to the ULOQ. The definition of rate of seroconversion will be the same definition as the rate of seroconversion in Phase 2 NH.

9.5.4. Exploratory Analyses

Exploratory analyses not addressed in Section 9.5.3 will be described in the SAP before database lock.

9.5.5. Subgroup Analyses

The protocol does not define any formal subgroup analyses, and the study is not adequately powered to perform subgroup analyses.

9.6. Study Analyses

A Day 29 IA is planned in this study for the Phase 1/2, Phase 2 NH, and Phase 2 Extension parts separately. The IA for the Phase 1/2 part will be performed after all participants have completed the Day 29 Visit. All relevant data to the IA will be cleaned (ie, data that are as clean as possible).

For the Phase 2 NH and Phase 2 Extension parts, an IA of safety (reactogenicity and immunogenicity as applicable) will be performed after participants have completed the Day 29 Visit for each part of the study. All data relevant to the IA through the Day 29 Visit will be cleaned (ie, data that are as clean as possible). An interim study report may be generated, in which case a formal database lock will be performed to support the interim study report.

The IA will be performed by a separate team of unblinded programmers and statisticians. The analysis will be presented by vaccination groups. Except for a limited number of Sponsor and CRO personnel who will be unblinded to perform the IA, the study site staff, Investigators, study monitors, and participants will remain blinded until after the final database lock for final analysis.

The final analysis of safety, reactogenicity, and immunogenicity will be performed after all participants have completed all planned study procedures. The results of this analysis will be presented in a final CSR, including individual listings.

Further details can be found in the SAP.

10. REFERENCES

Aretz HT, Billingham ME, Edwards WD, Factor SM, Fallon JT, Fenoglio JJ Jr, et al. Myocarditis. A histopathologic definition and classification. Am J Cardiovasc Pathol 1987; 1:3–14

Baden LR, El Sahly HM, Essink B, Kotloff K, Frey S, Novak R, et al; COVE Study Group. Efficacy and safety of the mRNA-1273 SARS-CoV-2 vaccine. N Engl J Med. 2021;384(5):403-16.

Centers for Disease Control and Prevention (CDC). CDC seasonal flu vaccine effectiveness studies [Internet]. Atlanta (GA). 2020a Dec 11 (cited 2021 Mar 17). Available from: https://www.cdc.gov/flu/vaccines-work/effectiveness-studies.htm.

Centers for Disease Control and Prevention (CDC). Coronavirus disease 2019 (COVID-19): 2020 interim case definition [Internet]. Atlanta (GA). 2020b Aug 5 (cited 2021 Mar 17). Available from: https://ndc.services.cdc.gov/case-definitions/coronavirus-disease-2019-2020-08-05/.

Centers for Disease Control and Prevention (CDC). Influenza virus testing methods [Internet]. 2020c Aug 10 (cited 2021 June 22). Available from: https://www.cdc.gov/flu/professionals/diagnosis/table-testing-methods.htm

Centers for Disease Control and Prevention (CDC). COVID-19: appendix A – glossary of key terms: close contact [Internet]. Atlanta (GA). 2021a Mar 11 (cited 2021 Mar 17). Available from: https://www.cdc.gov/coronavirus/2019-ncov/php/contact-tracing/contact-tracing-plan/appendix.html#contact.

Centers for Disease Control and Prevention (CDC). COVID-19: Interim guidance on ending isolation and precautions for adults with COVID-19 [Internet]. Atlanta (GA). 2021b Feb 13 (cited 2021 Jul 09). Available from: https://www.cdc.gov/coronavirus/2019-ncov/hcp/duration-isolation.html?CDC_AA_refVal=https%3A%2F%2Fwww.cdc.gov%2Fcoronavirus%2F2019-ncov%2Fcommunity%2Fstrategy-discontinue-isolation.html#Annex.

ClinicalTrials.gov [Internet]. Bethesda (MD): National Library of Medicine (US). Identifier NCT03382405: Safety, reactogenicity, and immunogenicity of cytomegalovirus vaccines mRNA-1647 and mRNA-1443 in healthy adults. 2017 Dec 22 (updated 2021 Jan 15; cited 2021 Mar 17). Retrieved from: https://clinicaltrials.gov/ct2/show/NCT03382405.

ClinicalTrials.gov [Internet]. Bethesda (MD): National Library of Medicine (US). Identifier NCT04064905: Safety, tolerability, and immunogenicity of Zika vaccine mRNA-1893 in healthy flavivirus seropositive and seronegative adults. 2019 Aug 22 (updated 2021 Mar 5; cited 2021 Mar 17). Retrieved from: https://clinicaltrials.gov/ct2/show/NCT04064905.

ClinicalTrials.gov [Internet]. Bethesda (MD): National Library of Medicine (US). Identifier NCT03392389: Safety, reactogenicity, and immunogenicity of mRNA-1653 in healthy adults. 2018 Jan 8 (updated 2020 Feb 6; cited 2021 Mar 26). Retrieved from: https://clinicaltrials.gov/ct2/show/study/NCT03392389.

ClinicalTrials.gov [Internet]. Bethesda (MD): National Library of Medicine (US). Identifier NCT04283461: Safety and immunogenicity study of 2019-nCov vaccine (mRNA-1273) for prophylaxis of SARS-CoV-2 infection (COVID-19). 2020 Feb 21 (updated 2021 Apr 9; cited 2021 Mar 17). Retrieved from: https://clinicaltrials.gov/ct2/keydates/NCT04283461.

ClinicalTrials.gov [Internet]. Bethesda (MD): National Library of Medicine (US). Identifier NCT04405076: Dose-confirmation study to evaluate the safety, reactogenicity, and immunogenicity of mRNA-1273 COVID-19 vaccine in adults aged 18 years and older. 2020 May 28 (updated 2021 Mar 21; cited 2021 Apr 10). Retrieved from: https://clinicaltrials.gov/ct2/show/NCT04405076.

ClinicalTrials.gov [Internet]. Bethesda (MD): National Library of Medicine (US). Identifier NCT04470427: A study to evaluate efficacy, safety, and immunogenicity of mRNA-1273 vaccine in adults aged 18 years and older to prevent COVID-19. 2020 Jul 14 (updated 2021 Feb 25; cited 2021 Apr 10). Retrieved from: https://clinicaltrials.gov/ct2/show/NCT04470427.

Department of Health and Human Services (DHHS), Food and Drug Administration, Center for Biologics Evaluation and Research (US). Guidance for industry: Clinical data needed to support the licensure of seasonal inactivated influenza vaccines. May 2007a [cited 2021 Mar 18] [17 screens]. Available from:
https://www.fda.gov/files/vaccines%2C%20blood%20%26%20biologics/published/Guidance-for-Industry--Clinical-Data-Needed-to-Support-the-Licensure-of-Seasonal-Inactivated-Influenza-Vaccines.pdf.

Department of Health and Human Services (DHHS), Food and Drug Administration, Center for Biologics Evaluation and Research (US). Guidance for industry: Toxicity grading scale for healthy adult and adolescent volunteers enrolled in preventive vaccine clinical trials. September 2007b [cited 2021 Mar 18] [10 screens]. Available from:
https://www.fda.gov/downloads/BiologicsBloodVaccines/GuidanceComplianceRegulatoryInformation/Guidances/Vaccines/ucm091977.pdf.

Department of Health and Human Services (DHHS), Food and Drug Administration (US). Guidance for industry, investigators, and institutional review boards: Conduct of clinical trials of medical products during the COVID-19 public health emergency. March 2020 [updated 2021 Jan 27; cited 2021 Mar 18] [38 screens]. Available from: https://www.fda.gov/media/136238/download.

Dunning AJ, DiazGranados CA, Voloshen T, Hu B, Landolfi VA, Talbot HK. Correlates of protection against influenza in the elderly: results from an influenza vaccine efficacy trial. Clin Vaccine Immunol. 2016;23(3):228-35.

European Medicines Agency (EMA). Committee for Medicinal Products for Human Use. Guideline on influenza vaccines: non-clinical and clinical module. London, 21 July 2016. EMA/CHMP/VWP/457259/2014 [cited 2021 Mar 18] [31 screens]. Available from: https://www.ema.europa.eu/en/documents/scientific-guideline/influenza-vaccines-non-clinical-clinical-module_en.pdf.

Feldman RA, Fuhr R, Smolenov I, Ribeiro AM, Panther L, Watson M, et al. mRNA vaccines against H10N8 and H7N9 influenza viruses of pandemic potential are immunogenic and well tolerated in healthy adults in phase 1 randomized clinical trials. Vaccine. 2019;37(25):3326-34.

Gargano JW, Wallace M, Hadler SC, Langley G, Su JR, Oster ME, et al. Use of mRNA COVID-19 vaccine after reports of myocarditis among vaccine recipients: update from the Advisory Committee on Immunization Practices – United States, June 2021. MMWR Morb Mortal Wkly Rep. 2021;70(27):977-82.

Jackson LA, Anderson EJ, Rouphael NG, Roberts PC, Makhene M, Coler RN, et al; mRNA-1273 Study Group. An mRNA vaccine against SARS-CoV-2 – preliminary report. N Engl J Med. 2020;383(20):1920-31.

Monto AS. Reflections on the Global Influenza Surveillance and Response System (GISRS) at 65 years: an expanding framework for influenza detection, prevention, and control. Influenza Other Respir Viruses. 2018;12(1):10-2.

Polack FP, Thomas SJ, Kitchin N, Absalon J, Gurtman A, Lockhart S, et al; C4591001 Clinical Trial Group. Safety and efficacy of the BNT162b2 mRNA COVID-19 vaccine. N Engl J Med 2020;383(27):2603-15.

Riedel S, Hobden JA, Miller S, Morse SA, Mietzner TA, Detrick B, et al. Jawetz, Melnick & Adelberg's Medical Microbiology. 28th ed. New York: McGraw-Hill Education; 2019. Chapter 39, Orthomyxoviruses (Influenza Viruses); p. 581-94.

Rockman S, Laurie KL, Parkes S, Wheatley A, Barr IG. New technologies for influenza vaccines. Microorganisms. 2020;8(11):1745.

Rüggeberg JU, Gold MS, Bayas JM, Blum MD, Bonhoeffer J, Friedlander S, et al; Brighton Collaboration Anaphylaxis Working Group. Anaphylaxis: case definition and guidelines for data collection, analysis, and presentation of immunization safety data. Vaccine. 2007;25(31):5675-84.

Shimabukuro TT, Cole M, Su JR. Reports of anaphylaxis after receipt of mRNA COVID-19 vaccines in the US—December 14, 2020-January 18, 2021. JAMA. 2021;325(11):1101-2.

World Health Organization (WHO). Influenza (seasonal): Ask the expert: influenza Q&A [Internet]. 2018 Nov 6 (cited 2021 Mar 17. Available from: https://www.who.int/news-room/fact-sheets/detail/influenza-(seasonal).

Zent O, Arras-Reiter C, Broeker M, Hennig R. Immediate allergic reactions after vaccinations – a post-marketing surveillance review. Eur J Pediatr. 2002;161(1):21-5.

11. SUPPORTING DOCUMENTATION AND OPERATIONAL CONSIDERATIONS

11.1. APPENDIX 1: Study Governance Considerations

11.1.1. Regulatory and Ethical Considerations

This study will be conducted in accordance with the protocol and with the following:

- Consensus ethical principles derived from international guidelines, including the Declaration of Helsinki and Council for International Organizations of Medical Sciences International Ethical Guidelines.
- Applicable ICH GCP guidelines.
- Applicable laws and regulatory requirements.
- The protocol, protocol amendments, ICF, IB, and other relevant documents (eg, advertisements) must be submitted to an IRB by the Investigator and reviewed and approved by the IRB before the study is initiated.
- Any amendments to the protocol will require IRB approval before implementation of changes made to the study design, except for changes necessary to eliminate an immediate hazard to study participants.
- The Investigator will be responsible for the following:
  - Providing written summaries of the status of the study to the IRB annually or more frequently in accordance with the requirements, policies, and procedures established by the IRB.
  - Notifying the IRB of SAEs or other significant safety findings as required by IRB procedures.
  - Providing oversight of the conduct of the study at the site and adherence to requirements of 21 CFR, ICH GCP guidelines, the IRB, and all other applicable local regulations.

11.1.2. Study Monitoring

Before an investigational study site can enter a participant into the study, the Sponsor or its representatives will visit the study site for the following:

- Determine the adequacy of the facilities.
- Discuss with the Investigator(s) and other personnel their responsibilities with regard to protocol adherence, and the responsibilities of the Sponsor or its representatives. This will be documented in a Clinical Study Agreement between the Sponsor, the designated CRO, and the Investigator.

According to ICH GCP guidelines, the Sponsor of the study is responsible for ensuring the proper conduct of the study with regard to protocol adherence and validity of data recorded on the eCRFs. The study monitor's duties are to aid the Investigator and the Sponsor in the maintenance of complete, accurate, legible, well-organized, and easily retrievable data. The study monitor will advise the Investigator of the regulatory necessity for study-related monitoring, audits, IRB review, and inspection by providing direct access to the source data and/or documents. In addition, the study monitor will explain to and interpret for the Investigator all regulations applicable to the clinical evaluation of IP as documented in ICH guidelines.

It is the study monitor's responsibility to inspect the eCRFs and source documentation throughout the study to protect the rights of the participants; to verify adherence to the protocol; to verify completeness, accuracy, and consistency of the data; and to confirm adherence of study conduct to any local regulations. Details will be outlined in the clinical monitoring plan. During the study, a monitor from the Sponsor or a representative will have regular contacts with the study site, for the following purposes:

- Provide information and support to the Investigator(s).
- Confirm that facilities remain acceptable.
- Confirm that the investigational team is adhering to the protocol, that the data are being accurately recorded in the eCRFs, and that IP accountability checks are being performed.
- Perform source data verification. This includes a comparison of the data in the eCRFs with the participant's medical records at the hospital or practice and other records relevant to the study. This will require direct access to all original records for each participant (eg, clinical charts or electronic medical record system).
- Record and report any protocol deviations not previously sent.
- Confirm that AEs and SAEs have been properly documented on eCRFs, that any SAEs have been forwarded to the SAE Hotline, and that those SAEs that meet criteria for reporting have been forwarded to the IRB.

The monitor will be available between visits if the Investigator(s) or other staff needs information or advice.

11.1.3. Audits and Inspections

The Sponsor, their designee(s), the IRB, or regulatory authorities will be allowed to conduct site visits to the investigational facilities for the purpose of monitoring or inspecting any aspect of the study. The Investigator agrees to allow the Sponsor, their designee(s), the IRB, or regulatory

authorities to inspect the IP storage area, IP stocks, IP records, participant charts, and study source documents, and other records relative to study conduct.

Authorized representatives of the Sponsor, a regulatory authority, and the IRB may visit the study site to perform audits or inspections, including source data verification. The purpose of a Sponsor audit or inspection is to systematically and independently examine all study-related activities and documents to determine whether these activities were conducted and whether data were recorded, analyzed, and accurately reported according to the protocol, ICH GCP (R2), and any applicable regulatory requirements. The Investigator should contact the Sponsor immediately if contacted by a regulatory agency about an inspection.

The Principal Investigator must obtain IRB approval for the investigation. Initial IRB approval and all materials approved by the IRB for this study, including the participant ICF and recruitment materials must be maintained by the Investigator and made available for inspection.

11.1.4. Financial Disclosure

The Investigator is required to provide financial disclosure information to allow the Sponsor to submit the complete and accurate certification or disclosure statements required under 21 CFR 54. In addition, the Investigator must provide the Sponsor with a commitment to promptly update this information if any relevant changes occur during the course of the investigation and for 1 year following the completion of the study.

The Sponsor, the CRO, and the study site are not financially responsible for further testing or treatment of any medical condition that may be detected during the screening process. In addition, in the absence of specific arrangements, the Sponsor, the CRO, and the study site are not financially responsible for further treatment of the disease under study.

11.1.5. Recruitment Procedures

Advertisements to be used for the recruitment of study participants and any other written information regarding this study to be provided to the participant should be submitted to the Sponsor for approval. All documents must be approved by the IRB.

11.1.6. Informed Consent Process

The informed consent document(s) must meet the requirements of 21 CFR 50, local regulations, ICH guidelines, Health Insurance Portability and Accountability Act requirements, where applicable, and the IRB or study site. All consent documents will be approved by the appropriate IRB. The actual ICF used at each study site may differ, depending on local regulations and IRB requirements. However, all versions must contain the standard information found in the sample

ICF provided by the Sponsor. Any change to the content of the ICF must be approved by the Sponsor and the IRB prior to the form being used.

If new information becomes available that may be relevant to the participant's willingness to continue participation in the study, this will be communicated to him/her in a timely manner. Such information will be provided via a revised ICF or an addendum to the original ICF.

The Investigator or his/her representative will explain the nature of the study to the participant and answer all questions regarding the study.

The Investigator is responsible for ensuring that the participant fully understands the nature and purpose of the study. Information should be given in both oral and written form whenever possible.

No participant should be obliged to participate in the study. The participant must be informed that participation is voluntary. Participants, their relatives, guardians, or (if applicable) legal representatives must be given ample opportunity to inquire about details of the study. The information must make clear that refusal to participate in the study or withdrawal from the study at any stage is without any prejudice to the participant's subsequent care.

The participant must be allowed sufficient time to decide whether they wish to participate.

The participant must be made aware of and give consent to direct access to his/her source medical records by study monitors, auditors, the IRB, and regulatory authorities. The participant should be informed that such access will not violate participant confidentiality or any applicable regulations. The participant should also be informed that he/she is authorizing such access by signing the ICF.

A copy of the ICF(s) must be provided to the participant.

A participant who is rescreened is not required to sign another ICF if the rescreening occurs within 28 days from the previous ICF signature date (within the initial screening period).

The ICF will also explain that excess serum from immunogenicity testing may be used for future research, which may be performed at the discretion of the Sponsor to further characterize the immune response to influenza viruses, for additional assay development, and to examine the immune response across influenza viruses.

11.1.7. Protocol Amendments

No change or amendment to this protocol may be made by the Investigator or the Sponsor after the protocol has been agreed to and signed by all parties unless such change(s) or amendment(s) has (have) been agreed upon by the Investigator or the Sponsor. Any change agreed upon will be recorded in writing, and the written amendment will be signed by the Investigator and the Sponsor. The IRB approval is required prior to the implementation of an amendment, unless overriding safety reasons warrant immediate action, in which case the IRB(s) will be promptly notified.

Any modifications to the protocol or the ICF that may impact the conduct of the study or the potential benefit of the study or may affect participant safety, including changes of study objectives, study design, participant population, sample sizes, study procedures, or significant administrative aspects will require a formal amendment to the protocol. Such amendment will be released by the Sponsor, agreed to by the Investigator(s), and approved by the relevant IRB(s) prior to implementation. A signed and dated statement that the protocol, any subsequent relevant amended documents, and the ICF have been approved by relevant IRB(s) must be provided to the Sponsor before the study is initiated.

Administrative changes to the protocol are minor corrections and/or clarifications that have no effect on the way the study is to be conducted. These administrative changes will be released by the Sponsor and agreed to by the Investigators, and the IRB(s) will be notified.

11.1.8. Protocol Deviations

Noncompliance may be on the part of the participant, the Investigator, or the study site staff. As a result of deviations, corrective actions are to be developed by the site and implemented promptly.

It is the responsibility of the Site Investigator to use continuous vigilance to identify and report deviations to the Sponsor or its designee in a timely manner. All deviations must be addressed in study source documents and reported to the study monitor. Protocol deviations must be sent to the reviewing IRB per their policies. The Site Investigator is responsible for knowing and adhering to the reviewing IRB requirements.

11.1.9. Data Protection

Participants will be assigned a unique identifier by the Sponsor. Any participant records or datasets that are transferred to the Sponsor will contain the identifier only; participant names or any information that would make the participant identifiable will not be transferred.

The participant must be informed that his/her personal study-related data will be used by the Sponsor in accordance with local data protection law. The level of disclosure must also be explained to the participant.

The participant must be informed that his/her medical records may be examined by Clinical Quality Assurance (QA) auditors or other authorized personnel appointed by the Sponsor, by appropriate IRB members, and by inspectors from regulatory authorities.

Individual participant medical information obtained as a result of this study is considered confidential, and disclosure to third parties is prohibited. Information will be accessible to authorized parties or personnel only. Medical information may be given to the participant's physician or to other appropriate medical personnel responsible for the participant's well-being.

Each participant will be asked to complete a form allowing the Investigator to notify the participant's primary HCP of his/her participation in this study.

All laboratory specimens, evaluation forms, reports, and other records will be identified in a manner designed to maintain participant confidentiality. All records will be kept in a secure storage area with limited access. Clinical information will not be released without the written permission of the participant, except as necessary for monitoring and auditing by the Sponsor, its designee, the relevant regulatory authority, or the IRB.

The Investigator and all employees and coworkers involved with this study may not disclose or use for any purpose other than performance of the study, any data, record, or other unpublished, confidential information disclosed to those individuals for the purpose of the study. Prior written agreement from the Sponsor or its designee must be obtained for the disclosure of any confidential information to other parties.

11.1.10. Sample Retention and Future Biomedical Research

The Sponsor may store laboratory samples for the time frame specified in the ICF to address study objectives or further scientific questions related to mRNA-1010 or anti-respiratory viral immune response. In addition, identifiable samples can be destroyed at any time at the request of the participant. During the study or during the retention period, in addition to the analysis outlined in the study endpoints, [redacted] In the Phase 2 NH and Phase 2 Extension parts of the study, these analyses will extend the search for other potentially relevant biomarkers to investigate the effect of mRNA-1010 as well as to determine how changes in biomarkers may relate to exposure and clinical outcomes. A decision to perform such exploratory research may arise from new scientific findings related to the vaccine class, as well as reagents for biomarker assay development.

11.1.11. Safety Oversight

Safety monitoring for this study will include the blinded study team members, inclusive of, at a minimum, the Sponsor's medical monitor, CRO's medical monitor, a blinded IST, and an unblinded DSMB. The study team will conduct ongoing blinded safety reviews during the study and will be responsible for notifying the IST and DSMB of potential safety signal events or the triggering of pause rules.

The IST will consist of at least 2 Sponsor physicians who will be voting members not directly involved in the clinical development of the study. For the initial stage in the Phase 1/2 part of the study, the IST will conduct a blinded review of all available safety data after at least 36 participants

(9 participants per vaccination group; Table 6) have completed their Day 8 Visit. Vaccination of the remaining participants in the expansion stage will continue in each vaccination group if no pause rules have been met and the safety and tolerability are acceptable. Prior to the start of the Phase 2 NH part of the study, the IST will also conduct a blinded review of available safety data from the Phase 1/2 part of the study. Finally, the IST will also conduct ad hoc reviews as requested by the study medical monitor and the study team.

An independent unblinded DSMB will be used throughout the conduct of this study. This committee will be composed of independent members with relevant therapeutic and/or biostatistical expertise to allow for the ongoing unblinded review of safety data from this study population. Safety data will be reviewed according to intervals defined in the DSMB charter and will also occur as needed when study stopping or pausing criteria are met, or as otherwise requested by the study team and/or IST.

Details regarding the IST and DSMB composition, responsibilities, procedures, and frequency of data review will be defined in their respective charters.

11.1.12. Dissemination of Clinical Study Data

The Sponsor shares information about clinical studies and results on publicly accessible websites, based on international and local legal and regulatory requirements, and other clinical study disclosure commitments established by pharmaceutical industry associations. These websites include clinicaltrials.gov, European Union clinical trial register (eu.ctr), and some national registries.

11.1.13. Data Quality Assurance and Quality Control

Data collection is the responsibility of the clinical study staff at the site under the supervision of the Site Investigator. The Investigator is responsible for ensuring the accuracy, completeness, legibility, and timeliness of the data reported.

- All participant data relating to the study will be recorded in the eCRF unless transmitted to the Sponsor or designee electronically (eg, laboratory data). The Investigator is responsible for verifying that data entries are accurate and correct by physically or electronically signing the eCRF.
- The Investigator must maintain accurate documentation (source data) that supports the information entered in the eCRF.
- The Investigator must permit study-related monitoring, audits, IRB review, and regulatory agency inspections and provide direct access to source data documents.

- Monitoring details describing strategy (eg, risk-based initiatives in operations and quality such as risk management and mitigation strategies and analytical risk-based monitoring), methods, responsibilities and requirements, including handling of noncompliance issues and monitoring techniques (central, remote, or onsite monitoring) are provided in the clinical monitoring plan.
- The Sponsor or designee is responsible for the data management of this study, including quality checking of the data.
- The Sponsor assumes accountability for actions delegated to other individuals (eg, CROs).
- Study monitors will perform ongoing source data verification to confirm that data entered into the eCRF by authorized site personnel are accurate, complete, and verifiable from source documents; that the safety and rights of participants are being protected; and that the study is being conducted in accordance with the currently approved protocol and any other study agreements, ICH GCP, and all applicable regulatory requirements.
- Records and documents, including signed ICFs, pertaining to the conduct of this study must be retained by the Investigator for a period of at least 2 years after the last marketing application approval or, if not approved, 2 years following the discontinuance of the test article for investigation. If this requirement differs from any local regulations, the local regulations will take precedence unless the local retention policy is less than 2 years. No records may be destroyed during the retention period without the written approval of the Sponsor. No records may be transferred to another location or party without written notification to the Sponsor.

Quality assurance includes all the planned and systematic actions that are established to ensure that the clinical study is performed, and the data are generated, documented (recorded), and reported according to ICH GCP and local/regional regulatory standards.

A QA representative from the Sponsor or qualified designee, who is independent of and separated from routine monitoring, may periodically arrange inspections/audits of the clinical study by reviewing the data obtained and procedural aspects. These inspections may include onsite inspections/audits and source data checks. Direct access to source documents is required for the purpose of these periodic inspections/audits.

11.1.14. Data Collection and Management

This study will be conducted in compliance with ICH GCP guidelines. This study will also be conducted in accordance with the most recent version of the Declaration of Helsinki.

This study will use electronic data collection to collect data directly from the study site using eCRFs. The Investigator is responsible for ensuring that all sections of each eCRF are completed promptly and correctly and that entries can be verified against any source data.

Study monitors will perform source document verification to identify inconsistencies between the eCRFs and source documents. Discrepancies will be resolved in accordance with the principles of GCP. Detailed study monitoring procedures are provided in the clinical monitoring plan.

Adverse events will be coded with MedDRA. Concomitant medications will be coded using WHODrug Global.

11.1.15. Source Documents

Source documents are original documents or certified copies and include, but are not limited to, eDiaries, medical and hospital records, screening logs, ICFs, telephone contact logs, and worksheets. Source documents provide evidence for the existence of the participant and substantiate the integrity of the data collected. Source documents are filed at the Investigator's site.

Data reported on the case report form or entered in the eCRF that are transcribed from source documents must be consistent with the source documents or the discrepancies must be explained. The Investigator may need to request previous medical records or transfer records, depending on the study. Also, current medical records must be available.

The Sponsor or its designee requires that the Investigator prepare and maintain adequate and accurate records for each participant treated with the IP. Source documents such as any hospital, study site, or office charts and the signed ICFs are to be included in the Investigator's files with the participant's study records.

11.1.16. Retention of Records

The Principal Investigator must maintain all documentation relating to the study for a period of at least 2 years after the last marketing application approval or, if not approved, 2 years following the discontinuance of the test article for investigation. If this requirement differs from any local regulations, the local regulations will take precedence unless the local retention policy is less than 2 years.

If it becomes necessary for the Sponsor or the regulatory authority to review any documentation relating to the study, the Investigator must permit access to such records. No records will be destroyed without the written consent of the Sponsor, if applicable. It is the responsibility of the Sponsor to inform the Investigator when these documents no longer need to be retained.

11.1.17. Study and Site Closure

If the study is prematurely terminated or suspended, the Sponsor shall promptly inform the Investigators, the IRBs, the regulatory authorities, and any CRO(s) used in the study of the reason for termination or suspension, as specified by the applicable regulatory requirements. The Investigator shall promptly inform the participant and should ensure appropriate participant therapy and/or follow-up.

The Sponsor or designee reserves the right to close the study site or terminate the study at any time for any reason at the sole discretion of the Sponsor.

The Investigator may initiate study site closure at any time, provided there is reasonable cause and sufficient notice is given in advance of the intended termination.

Reasons for the early closure of a study site by the Sponsor or Investigator may include but are not limited to the following:

- Continuation of the study represents a significant medical risk to participants.
- Failure of the Investigator to comply with the protocol, the requirements of the IRB or local health authorities, the Sponsor's procedures, or ICH GCP guidelines.
- Inadequate recruitment of participants by the Investigator.
- Discontinuation of further mRNA-1010 development.

Study sites will be closed upon study completion. A study site is considered closed when all required documents and study supplies have been collected and a study site closure visit has been performed.

11.1.18. Publication Policy

The results of this study may be published or presented at scientific meetings. If this is foreseen, the Investigator agrees to submit all manuscripts or abstracts to the Sponsor before submission. This allows the Sponsor to protect proprietary information and to provide comments.

The Sponsor will comply with the requirements for publication of study results. In accordance with standard editorial and ethical practice, the Sponsor will generally support publication of multicenter studies only in their entirety and not as individual site data. In this case, a Coordinating Investigator will be designated by mutual agreement.

Authorship will be determined by mutual agreement and in line with International Committee of Medical Journal Editors authorship requirements.

The clinical study plan and the results of the study will be published on www.ClinicalTrials.gov in accordance with 21 CFR 50.25(c). The results of and data from this study belong to the Sponsor.

11.2. APPENDIX 2: Contraceptive Guidance

Definitions: Woman of Childbearing Potential

Females of childbearing potential are those who are considered fertile following menarche and until becoming postmenopausal unless permanently sterile (as follows). If fertility is unclear (eg, amenorrhea in adolescents or athletes) and a menstrual cycle cannot be confirmed before study vaccination, additional evaluation should be considered.

Females in the following categories are not considered females of childbearing potential:

1. Premenarchal
2. Premenopausal surgically sterile female with 1 of the following:
   a. Documented complete hysterectomy
   b. Documented surgical sterilization

   For individuals with permanent infertility due to an alternate medical cause other than the above (eg, Müllerian agenesis, androgen insensitivity), Investigator's discretion should be applied when determining study entry.

   Note: Documentation can come from the site personnel's review of the participant's medical records, medical examination, or medical history interview.
3. Postmenopausal female
   - A postmenopausal state is defined as no menses for 12 months without an alternative medical cause. The following age-specific requirements apply:
     - Females < 50 years of age would be considered postmenopausal if they have been amenorrheic for 12 months or more following cessation of exogenous hormonal treatments and if they have luteinizing hormone and FSH levels in the postmenopausal range for the institution.
     - Females ≥ 50 years of age would be considered postmenopausal if they have been amenorrheic for 12 months or more, had radiation-induced menopause with last menses > 1 year ago, or had chemotherapy-induced menopause with last menses > 1 year ago.
   - A high FSH level in the postmenopausal range may be used to confirm a postmenopausal state in women not using hormone replacement therapy (HRT).
   - Females on HRT and whose menopausal status is in doubt will be required to use 1 of the nonestrogen hormonal highly effective contraception methods if they wish to

continue their HRT during the study. Otherwise, they must discontinue HRT to allow confirmation of postmenopausal status before study enrollment.

Contraception Guidance:

Adequate female contraception is defined as consistent and correct use of an FDA-approved contraceptive method in accordance with the product label. The following are examples:

- Barrier method (such as condoms, diaphragm, or cervical cap) used in conjunction with spermicide.
- Intrauterine device.
- Prescription hormonal contraceptive taken or administered via oral (pill), transdermal (patch), subdermal, or IM route.
- Sterilization of a female participant's monogamous male partner prior to entry into the study.

Note that periodic abstinence (eg, calendar, ovulation, symptothermal, postovulation methods) and withdrawal are not acceptable methods of contraception.

11.3. APPENDIX 3: Adverse Events of Special Interest Terms

Investigators should report all AEs, which fall into the following categories presented in Table 16 as an AESI per the reporting processes specified in Section 8.4.14. The following AESIs are medical concepts that are generally of interest in vaccine safety surveillance per the Brighton Collaboration and Safety Platform for Emergency Vaccines.

Table 16 Adverse Events of Special Interest

Adverse Event of Special Interest	Notes
Thrombocytopenia	• Platelet counts < 150 × 10^9<br>• Including but not limited to immune thrombocytopenia, platelet production decreased, thrombocytopenia, thrombocytopenic purpura, thrombotic thrombocytopenic purpura, or HELLP syndrome
New onset of or worsening of the following neurologic diseases	• Guillain-Barre Syndrome<br>• Acute disseminated encephalomyelitis<br>• Idiopathic peripheral facial nerve palsy (Bell's palsy)<br>• Seizures including, but not limited to, febrile seizures and/or generalized seizures/convulsions
Anaphylaxis	• Anaphylaxis as defined in Section 8.4.5<br>• Following reporting procedures as specified in Section 8.4.14
Myocarditis/Pericarditis	• Myocarditis<br>• Pericarditis<br>• Myopericarditis

Abbreviation: HELLP = hemolysis, elevated liver enzymes, low platelet count.

11.4. APPENDIX 4: CDC Working Case Definition of Pericarditis, Myocarditis, and Myopericarditis Occurring After Receipt of COVID-19 mRNA Vaccines

Table 17 Case Definitions of Probable and Confirmed Myocarditis, Pericarditis, and Myopericarditis

Condition	Definition	
**Acute myocarditis**	**Probable case**	**Confirmed Case**
	Presence of ≥ 1 new or worsening of the following clinical symptoms:*	Presence of ≥ 1 new or worsening of the following clinical symptoms:*
	• chest pain, pressure, or discomfort	• chest pain, pressure, or discomfort
	• dyspnea, shortness of breath, or pain with breathing	• dyspnea, shortness of breath, or pain with breathing
	• palpitations	• palpitations
	• syncope	• syncope
	**OR,** infants and children aged < 12 years might instead have ≥ 2 of the following symptoms:	**OR,** infants and children aged < 12 years might instead have ≥ 2 of the following symptoms:
	• irritability	• irritability
	• vomiting	• vomiting
	• poor feeding	• poor feeding
	• tachypnea	• tachypnea
	• lethargy	• lethargy
	**AND**	**AND**
	≥ 1 new finding of	≥ 1 new finding of
	• troponin level above upper limit of normal (any type of troponin)	• Histopathologic confirmation of myocarditis†
	• abnormal electrocardiogram (ECG or EKG) or rhythm monitoring findings consistent with myocarditis§	
	• abnormal cardiac function or wall motion abnormalities on echocardiogram	• cMRI findings consistent with myocarditis in the presence of troponin level above upper limit of normal (any type of troponin)
	• cMRI findings consistent with myocarditis	
	**AND**	**AND**
	• No other identifiable cause of the symptoms and findings	• No other identifiable cause of the symptoms and findings

Condition	Definition
**Acute pericarditis****	Presence of ≥ 2 new or worsening of the following clinical features:
	• acute chest pain††
	• pericardial rub on exam
	• new ST-elevation or PR-depression on EKG
	• new or worsening pericardial effusion on echocardiogram or MRI
**Myopericarditis**	This term may be used for patients who meet criteria for both myocarditis and pericarditis.

Abbreviations: AV = atrioventricular; cMRI = cardiac magnetic resonance imaging; ECG or EKG = electrocardiogram; MRI = magnetic resonance imaging.

Note: An independent Cardiac Event Adjudication Committee (CEAC) comprised of medically qualified personnel, including cardiologists, will review suspected cases of myocarditis, pericarditis, and myopericarditis to determine if they meet Center for Disease Control and Prevention criteria for "probable" or "confirmed" events, (Gargano et al 2021), and provide the assessment to the Sponsor. The CEAC members will be blinded to study treatment. Details regarding the CEAC composition, responsibilities, procedures, and frequency of data review will be defined in the CEAC charter.

* Persons who lack the listed symptoms but who meet other criteria may be classified as subclinical myocarditis (probable or confirmed).

† Using the Dallas criteria (Aretz et al 1987). Autopsy cases may be classified as confirmed clinical myocarditis on the basis of meeting histopathologic criteria if no other identifiable cause.

§ To meet the ECG or rhythm monitoring criterion, a probable case must include at least one of 1) ST-segment or T-wave abnormalities; 2) Paroxysmal or sustained atrial, supraventricular, or ventricular arrhythmias; or 3) AV nodal conduction delays or intraventricular conduction defects.

Using either the original or the revised Lake Louise criteria.
https://www.sciencedirect.com/science/article/pii/S0735109718388430?via%3Dihubexternal icon

** https://academic.oup.com/eurheartj/article/36/42/2921/2293375external icon

†† Typically described as pain made worse by lying down, deep inspiration, or cough, and relieved by sitting up or leaning forward, although other types of chest pain might occur.

Reference: (Gargano et al 2021).

11.5. APPENDIX 5: Protocol Amendment History

The document history table for this protocol and the Protocol Amendment Summary of Changes table for the current Amendment 4 is located directly before Section 1.

Description of Amendments 1, 2, and 3 are presented in this appendix.

Amendment 1, 22 Jul 2021

Main Rationale for the Amendment:

The main purpose of this amendment is to incorporate a new part (Phase 2 Northern Hemisphere [NH]) into the study. By incorporating the Phase 2 NH part, the study intends to increase the size of the safety database for each dose level of the mRNA-1010 vaccine tested in the originally planned (ie, Phase 1/2 part) study. In addition, the Phase 2 NH part of the study will include a composition change of mRNA-1010 (strain update for the 2021-2022 NH season), as well as the inclusion of a licensed active comparator.

The summary of changes table provided here describes the major changes made in Amendment 1 relative to the original protocol, including the sections modified and the corresponding rationales. As applicable, the synopsis of Amendment 1 has been modified to correspond to changes in the body of the protocol.

Summary of Major Changes From Original Protocol to Protocol Amendment 1:

Section # and Name	Description of Change	Brief Rationale
Section 1.2 (Schema) and Section 1.3 (Schedule of Events)	• In the SoE for the Phase 1/2 part, the row specifying the assessment "Recording of SAEs and concomitant medications relevant to or for the treatment of the SAE" has been updated to include AESIs.<br>• In the SoE for the Phase 1/2 part, the row specifying "study completion" has been updated to change the wording to "Phase 1/2 completion" in line with the nomenclature used to identify the 2 study parts.<br>• Added an instruction in footnote 4 regarding vital sign measurements in the Phase 1/2 part SoE that the participants will be seated for at least 5 minutes before all measurements are taken.	• AESIs have been added to the Phase 1/2 part SoE as 1 of the safety assessments to be performed.<br>• In the Phase 1/2 part SoE, added an instruction in footnote 4 for vital sign measurements to take measurements only after the participants have been rested in a seating position for at least 5 minutes; number of participants in footnote 7 regarding assessment of [redacted] samples has been updated; and added clarification in footnote 8 for NP swab collection that the NP swab samples may be collected at home if needed in lieu of a study site visit.

Section # and Name	Description of Change	Brief Rationale
	• In the Phase 1/2 part SoE, footnote 7 has been updated to specify that [redacted]<br>• Added in footnote 8 regarding NP swab specimen in the Phase 1/2 part that the swabs may be collected as part of a home visit in lieu of a study site visit.<br>• Added study design for the Phase 2 NH part. Added SoE planned for the Phase 2 NH part.	• To provide the time points for the evaluation of assessments planned for the Phase 2 NH part.
Section 2.2.1 (mRNA-1010), Section 2.3.1 (Known Potential Benefits), and Section 2.3.3 (Overall Benefit/Risk Conclusion)	• Text has been updated to include information on the Phase 2 NH part of the study.	• Addition of a Phase 2 NH part to the originally planned study does not affect the potential benefits or risks associated with study participation for participants.
Section 3 (Objectives and Endpoints)	• New section (Section 3.2: Phase 2 NH) has been created to provide the objectives and endpoints of the newly added Phase 2 NH part of the study.<br>• The following exploratory objective for the Phase 1/2 part of the study [redacted]<br>• The primary safety endpoint of reporting of any SAEs and MAAEs from Day 1 through Day 181/EoS for the Phase 1/2 part has been updated to include AESIs as well.	• Provided the objectives and endpoints of the Phase 2 NH part of the study.<br>• Included recording of AESIs from Day 1 through Day 181/EoS in the primary safety endpoint for the Phase 1/2 part.

Section # and Name	Description of Change	Brief Rationale
	• Section 3 now includes 2 subsections to separate objectives for the 2 study parts (Phase 1/2 and Phase 2 NH).<br>• The originally planned objectives and endpoints (for the Phase 1/2 part) are now tabulated under Section 3.1 (Phase 1/2).	
Section 4.1 (General Design) and Section 4.2 (Scientific Rationale for Study Design)	• Added design, duration, randomization, vaccination groups, and dosing, assessment procedures for the Phase 2 NH part of the study.<br>• The age group "18 to 49 years" used as a stratification factor for randomization in the expansion stage of the Phase 1/2 part has been updated to "18 to < 50 years."	• Provided information on the specific procedures for the Phase 2 NH part of the study.<br>• Provided rationale for the general design elements of the Phase 2 NH part of the study.<br>• Updated the age stratification randomization factor for the expansion stage in the Phase 1/2 part of the study.
Section 4.3 (End of Study Definition)	• EoS definition has been updated to "The study will be considered ended at the completion of the last visit of the last participant in the Phase 2 NH part of the study or the last scheduled procedure as shown in the SoE (Table 2) for the last participant in the Phase 2 NH part of the study."	• Addition of the Phase 2 NH part resulted in updating the EoS definition as the study can be considered ended (ie, complete) only when the last participant's last visit in the Phase 2 NH part is completed.
Section 5 (Study Population)	• Added inclusion and exclusion criteria for the Phase 2 NH part in Section 5.2.1 and Section 5.2.2, respectively.<br>• Section 5 now includes 4 subsections to separate the inclusion/exclusion criteria for each part (Phase 1/2 and Phase 2 NH). The inclusion/exclusion criteria for the Phase 1/2 part are now listed in Section 5.1.1 and Section 5.1.2, respectively.<br>• Exclusion criterion No. 5 for the Phase 1/2 part has been updated to clarify that the participants may be rescreened if they are not medically stable at the Screening Visit.	• Provided a list of the inclusion and exclusion criteria for participant eligibility for Phase 2 NH part.<br>• Specified the allowance for rescreening of participants in the Phase 1/2 part for those who are not medically stable at screening.

Section # and Name	Description of Change	Brief Rationale
Section 5.3 (Lifestyle Restrictions)	• Text has been updated to clarify that only when participants choose to receive an available seasonal influenza vaccine, will they be requested to defer that vaccination while in the study.	• To clarify when participants will be requested to defer receiving commercially available seasonal influenza vaccination administered outside the study if they choose to receive it.
Section 6.1 (Investigational Products Administered), Section 6.2 (Randomization), Section 6.3 (Preparation/Handling/Storage/ Accountability) and subsections	• Text has been updated to include information on the IPs administered in the Phase 2 NH part of the study.<br>• The age group "18 to 49 years" used as a stratification factor for randomization in the expansion stage of the Phase 1/2 part has been updated to "18 to < 50 years."<br>• Name of the "mRNA-1010 Pharmacy Manual" has been updated to "Pharmacy Manual."	• Provided information on the IPs administered in the Phase 2 NH part and related instructions on storage, handling, preparation, processing, and accountability of the products administered.<br>• Updated the age stratification randomization factor for the expansion stage in the Phase 1/2 part of the study.<br>• Name of the Pharmacy Manual has been updated because of the restrictive wording. The manual will include information on the active comparator to be administered in the Phase 2 NH part in addition to the originally added details on mRNA-1010.
Section 6.5.2 (Concomitant Medications and Therapies)	• Revised language for 1 of the concomitant medications/therapies information criterion to specify that information of any seasonal influenza vaccine administered since September 2020 will be collected from participants and added in the eCRF.<br>• Added that the study staff should also ask the participants whether they have received any authorized or investigational COVID-19 vaccine at any time before the IP injection. If yes, the information should be recorded in the eCRF.	• Clarified text to avoid ambiguity regarding collection of previous seasonal influenza vaccine(s) received by the participants.<br>• Added a new data collection criterion regarding concomitant/prior medications received by the participant.

Section # and Name	Description of Change	Brief Rationale
Section 7.1 (Pause Rules)	• New section (Section 7.1.2: Pause Rules for the Phase 2 NH Part of the Study) has been created to provide the study pause rules of the newly added Phase 2 NH part of the study.<br>• Footnote b in Table 9 (Phase 1/2 Pause Rule Criteria, Events, and Thresholds - Proportion of Participants) was updated to include the following text: The calculation for ≥ 20% of participants in the expansion stage includes participants in the initial stage and the expansion stage as the total number of participants in the denominator.<br>• Section 7.1 now includes 2 main subsections to separate pause rules for the 2 study parts (Phase 1/2 [Section 7.1.1] and Phase 2 NH [Section 7.1.2]).	• Included explicit criteria as to when the Phase 2 NH part of the study should be paused.<br>• Footnote b in Phase 1/2 Pause Rule Criteria, Events, and Thresholds - Proportion of Participants) was updated to clarify text regarding calculation of proportion of participants for pause rule implementation if needed for the Phase 1/2 part.
Section 7.2 (Criteria for Delay or Withholding of Study Vaccination)	• The timing of receipt of COVID-19 vaccination before the planned Day 1 study vaccination that constitutes a delay in the receipt of study vaccination has been updated from "within 14 days" to "within 7 to 14 days."	• Updated the criterion pertaining to the receipt of COVID-19 vaccination that constitute a delay or withholding of study vaccination.
Section 8.1 (Safety Assessments and Procedures), Section 8.1.1 (Use of Electronic Diaries), Section 8.1.3 (Safety Laboratory Assessments), Section 8.1.4 (Vital Sign Measurements), Section 8.1.5 (Physical Examination), Section 8.1.6 (Assessments for Respiratory Viral Infections), Section 8.2 (Immunogenicity Assessments), and Section 8.3 (Efficacy Assessments)	• Text has been updated to include safety, immunogenicity, and efficacy assessments and procedures to be performed for Phase 2 NH part of the study.<br>• Clarified that the safety laboratory assessments will be performed only in the Phase 1/2 part of the study.<br>• Efficacy assessments as exploratory endpoints have been described in more detail.	• Provided text on the assessments and procedures planned for the Phase 2 NH part of the study.
Section 8.2 (Immunogenicity Assessments)	• Total maximum blood volume collected from the participant during his/her study participation has been updated from "250 mL" to "450 mL."	• Total maximum blood volume to be collected from a participant in the study has been updated.

Section # and Name	Description of Change	Brief Rationale
Section 8.4.4 (Medically Attended Adverse Events)	• Text has been updated to clarify that there will not be a separate MAAE section in the eCRF. Any MAAEs will be recorded under unsolicited AEs in the AE section of the eCRF.	• Clarified the precise section to record MAAEs in the eCRF.
Section 8.4.5 (Anaphylaxis)	• A new section has been added by incorporating the information about anaphylaxis previously added to the section of MAAEs (Section 8.4.4).	• All information related to anaphylaxis is now incorporated in a new separate section.
Section 8.4.6 (Influenza-Like Illness)	• Definitions have been added for ILI (protocol-defined, CDC-defined, and RT-PCR-confirmed).	• Added definitions for ILI.
Section 8.4.7 (Adverse Events of Special Interest), Section 8.4.14 (Reporting of Adverse Events of Special Interest), and Section 11.3 (Appendix 3: Adverse Events of Special Interest Terms); and Throughout the Protocol as Applicable for Phase 1/2 Part	• Added text defining AESIs for this protocol, reporting requirements, and the event terms to be considered as AESIs.<br>• AESIs also have been added as a safety assessment for the Phase 1/2 part of the study. Therefore, in addition to SAEs and MAAEs, AESIs will be collected from Day 1 through Day 181/EoS in the Phase 1/2 part.	• The Sponsor has identified AESIs for this protocol, which will be assessed from Day 1 through Day 181/EoS in both study parts (Phase 1/2 and Phase 2 NH). Text has been added to provide information on such events.
Section 8.9 (Biomarkers)	• Added text to specify that for the Phase 2 NH part, biomarker assessments are yet to be determined and possibly may include genomic and transcriptomic studies.<br>• Added text to specify that biomarker assessments will not be performed for the Phase 1/2 part of the study outside of the described immunogenicity assessments.	• Added text about biomarker assessments to be performed in the study.
Section 9.3 (Sample Size Determination)	• A sample size of 500 participants (150 participants in each of the 3 mRNA-1010 groups and 50 participants in the Active Comparator Group) is planned for the Phase 2 NH part and corresponding text has been added.	• Text added to justify the planned sample size for Phase 2 NH part of the study.
Section 9.5.2 (Safety Analyses) and Section 9.5.3 (Immunogenicity Analyses)	• Added analysis methods for safety and immunogenicity assessments for the Phase 2 NH part.	• Provided text for the statistical methodology of the assessments planned for the Phase 2 NH part.

Section # and Name	Description of Change	Brief Rationale
	• Text for safety analyses has been updated to include AESI assessment for the Phase 1/2 part as well.	• Text for safety analyses has been updated to include assessment of AESIs for the Phase 1/2 part.
Section 9.6 (Study Analyses)	• Added text for the planned IA for the Phase 2 NH part of the study.	• For the Phase 2 NH part, an IA of safety (and immunogenicity as applicable) will be performed after participants have completed the Day 29 Visit. In addition, an interim study report may be generated, in which case a formal database lock will be performed to support the interim study report.
Section 10 (References)	• Included the CDC reference for the exclusion criterion No. 10 applicable for the Phase 2 NH part.	• Added reference related to the exclusion criterion for the Phase 2 NH part.
Section 11.1.11 (Safety Oversight) and Section 4.1 (Study Design)	• Added text for the IST and DSMB responsibility for the Phase 2 NH data review.<br>• Added text to specify that for the initial stage in the Phase 1/2 part of the study, the IST will conduct a blinded review of all available safety data after at least 36 participants (9 participants per vaccination group) have completed their Day 8 Visit.<br>• Clarified that vaccination of the remaining participants in the expansion stage will continue in each vaccination group if no pause rules have been met and the safety and tolerability are acceptable.	• Added that prior to the start of the Phase 2 NH part of the study, the IST will also conduct a blinded review of available safety data from the Phase 1/2 part of the study.<br>• Text added to specify the IST review of available safety data from the initial stage of the Phase 1/2 part of the study.
Throughout the Protocol	• Minor formatting and edits were made for consistency.	• Minor formatting and edits were made for consistency and clarity.

Abbreviations: AE = adverse event; AESI = adverse event of special interest; CDC = Centers for Disease Control and Prevention; COVID-19 = coronavirus disease 2019; DSMB = Data and Safety Monitoring Board; eCRF = electronic case report form; EoS = end of study; IA = interim analysis; ILI = influenza-like illness; IP = investigational product; IST = internal safety team; MAAE = medically attended adverse event; NH = Northern Hemisphere; NP = nasopharyngeal swab; RT-PCR = reverse transcription polymerase chain reaction; SAE = serious adverse event; SoE = schedule of events.

Amendment 2, 19 Aug 2021

Main Rationale for the Amendment:

The purpose of this amendment is to describe the observation of myocarditis/pericarditis following COVID-19 mRNA vaccines.

The summary of changes table provided here describes the major changes made in Amendment 2 relative to Amendment 1, including the sections modified and the corresponding rationales. As applicable, the synopsis of Amendment 2 has been modified to correspond to changes in the body of the protocol.

Summary of Major Changes From Protocol Amendment 1 to Protocol Amendment 2:

Section # and Name	Description of Change	Brief Rationale
Section 2.3.2 (Risks From Study Participation and Their Mitigation)	• Added description of myocarditis and pericarditis following COVID-19 mRNA vaccines.	• To add information on very rare reports of myocarditis and pericarditis after vaccination with COVID-19 mRNA vaccines.
Section 4.1 (General Design) and Section 9.6 (Study Analyses)	• Clarified that the Phase 2 NH part will also access reactogenicity.	• Revised for consistency of presentation.
Section 8.4.3 (Solicited Adverse Reactions)	• Clarified documentation of solicited adverse reaction collection.	• Revised for consistency of documentation.
Section 8.4.7 (Adverse Events of Special Interest)	• Added information on myocarditis/pericarditis events.	• To provide guidance to the Investigators regarding reporting myocarditis and pericarditis for this study population.
Section 10 (References)	• Updated reference list.	• To support the text added to the protocol for myocarditis and pericarditis.
Section 11.4.(APPENDIX 4: CDC Working Case Definition of Pericarditis, Myocarditis, and Myopericarditis Occurring After Receipt of COVID-19 mRNA Vaccines)	• Added CDC case definitions for myocarditis and pericarditis.<br>• Added description of independent CEAC as footnote to the table.	• To provide guidance to the Investigators regarding assessing and reporting myocarditis and pericarditis for this study population.<br>• To add mechanism for review of suspected cases of myocarditis and pericarditis.

Abbreviations: AESI = adverse event of special interest; CDC = Centers for Disease Control and Prevention; CEAC = Cardiac Event Adjudication Committee COVID-19 = coronavirus disease 2019; mRNA = messenger RNA; NH = Northern Hemisphere.

Amendment 3, 21 Oct 2021

Main Rationale for the Amendment:

The purpose of this amendment is to update the Phase 2 NH part of the study to test 3 dose levels of mRNA-1010 (25, 50, and 100 µg total mRNA; Vaccination Groups 1 to 3).

The summary of changes table provided here describes the major changes made in Amendment 3 relative to Amendment 2, including the sections modified and the corresponding rationales. As applicable, the synopsis of Amendment 3 has been modified to correspond to changes in the body of the protocol. Minor editorial and/or formatting changes are not provided in this summary table.

Summary of Major Changes From Protocol Amendment 2 to Protocol Amendment 3:

Section # and Name	Description of Change	Brief Rationale
Title page: Sponsor Contact and Medical Monitor	• Name and contact info of the person responsible updated	• To indicate the change in personnel
Synopsis: Overall Study Design; Section 4.1. (General Design); Section 4.3. (Choice of Vaccine Dose); Section 6.1. (Investigational Products Administered); Section 6.2.2. (Phase 2 Northern Hemisphere); Section 6.3.1. (Preparation of Investigational Product); Section 9.3. (Sample Size Determination)	• Updated the Phase 2 NH part of the study to test 3 dose levels of mRNA-1010 (25, 50, and 100 µg total mRNA)	• Based on interim analysis results of the Phase 1/2 study, the 200 µg dose of mRNA-1010 did not meet the Sponsor's desired reactogenicity profile. The Sponsor intends to test a lower dose level of 25 µg in lieu of 200 µg, keeping the previous dose levels of 50 and 100 µg unchanged.
Table 1: Phase 1/2 - Schedule of Events; Table 2: Phase 2 Northern Hemisphere - Schedule of Events; Section 6.5.2. Concomitant Medications and Therapies	• Specified that all nonstudy vaccinations will be recorded throughout the study	• To specify that nonstudy vaccinations should be reported if received at any point during the study.
Figure 2: Phase 2 Northern Hemisphere – Study Schema	• Updated the Phase 2 NH part of the study to include 3 dose levels of mRNA-1010 (25, 50, and 100 µg total mRNA)	• Revised for consistency of presentation.
Section 2.3.3. (Overall Benefit/Risk Conclusion)	• Explanation of the differences in dosing between Phase 1/2 and Phase 2 NH	• Revised for consistency of presentation and to provide context.
Table 5: Vaccination Groups and Dose Levels	• Table split into Table 5a presenting dosing in Phase 1/2 and Table 5b presenting dosing in Phase 2.	• Revised for consistency of presentation.

Section # and Name	Description of Change	Brief Rationale
Section 8.1.6. (Assessments for Respiratory Viral Infections); Section 8.4.4. (Medically Attended Adverse Events)	• Clarified that RT-PCR confirmed ILI and/or COVID-19 should be captured as MAAEs.	• To distinguish the diagnosis criteria to be used

Abbreviations: COVID-19 = coronavirus disease 2019; ILI = influenza-like illness; MAAE = medically attended adverse event; mRNA = messenger RNA; NH = Northern Hemisphere; RT-PCR = reverse transcription polymerase chain reaction.

Signature Page for VV-CLIN-003485 v3.0

Approval	[redacted]<br>04-Feb-2022 20:38:37 GMT+0000

Signature Page for VV-CLIN-003485 v3.0

ModernaTX, Inc.

Protocol mRNA-1010-P101

A Phase 1/2, Randomized, Stratified, Observer-Blind, Dose-Ranging Study to Evaluate the Safety, Reactogenicity, and Immunogenicity of mRNA-1010 Seasonal Influenza Vaccine in Healthy Adults 18 Years and Older

Statistical Analysis Plan

SAP Version 3.0
Version Date of SAP: 22-April-2022

Prepared by:

PPD
3575 Quakerbridge Road
Suite 201
Hamilton, NJ 08619

TABLE OF CONTENTS

LIST OF ABBREVIATIONS V

1 INTRODUCTION 7

2 STUDY OBJECTIVES 7

2.1 PHASE 1/2 7
2.1.1 Primary Objectives 7
2.1.2 Secondary Objectives 8
2.1.3 Exploratory Objectives 8
2.2 PHASE 2 NORTHERN HEMISPHERE AND PHASE 2 EXTENSION 8
2.2.1 Primary Objectives 8
2.2.2 Secondary Objectives 9
2.2.3 Exploratory Objectives 9

3 STUDY ENDPOINTS 9

3.1 PHASE 1/2 9
3.1.1 Primary Endpoints 9
3.1.2 Secondary Endpoints 10
3.1.3 Exploratory Endpoints 10
3.2 PHASE 2 NORTHERN HEMISPHERE 10
3.2.1 Primary Endpoints 10
3.2.2 Secondary Endpoints 11
3.2.3 Exploratory Endpoints 11
3.3 PHASE 2 EXTENSION .... **ERROR! BOOKMARK NOT DEFINED.**

4 STUDY DESIGN 12

4.1 OVERALL STUDY DESIGN 12
4.1.1 Phase 1/2 13
4.1.1.1 Stage 1, Initial Stage 14
4.1.1.2 Stage 2, Expansion Stage 14
4.1.2 Phase 2 Northern Hemisphere 15
4.1.3 Phase 2 Extension 16
4.2 STATISTICAL HYPOTHESES 17
4.3 SAMPLE SIZE AND POWER 17
4.3.1 Phase 1/2 18
4.3.2 Phase 2 Northern Hemisphere 18
4.3.3 Phase 2 Extension 18
4.4 RANDOMIZATION 18
4.4.1 Phase 1/2 18
4.4.2 Phase 2 Northern Hemisphere 19
4.4.3 Phase 2 Northern Hemisphere 19
4.5 BLINDING AND UNBLINDING 19

5 ANALYSIS POPULATIONS 20

5.1 RANDOMIZATION SET 20
5.2 FULL ANALYSIS SET (FAS) 20

5.3 PER-PROTOCOL (PP) SET 20
5.4 SAFETY SET 22
5.5 SOLICITED SAFETY SET 23

6 STATISTICAL ANALYSIS 23

6.1 GENERAL CONSIDERATIONS 23
6.1.1 Phase 1/2 25
6.1.2 Phase 2 Northern Hemisphere 26
6.1.3 Phase 2 Extension 27
6.2 BACKGROUND CHARACTERISTICS 28
6.2.1 Participant Disposition 28
6.2.2 Demographics 29
6.2.3 Medical History 29
6.2.4 Prior and Concomitant Medications 30
6.2.5 Study Exposure 31
6.2.6 Major Protocol Deviations 31
6.2.7 COVID-19 Impact 31
6.3 SAFETY ANALYSIS 31
6.3.1 Adverse Events 32
6.3.1.1 Incidence of Adverse Events 33
6.3.1.2 TEAEs by System Organ Class and Preferred Term 33
6.3.1.2 TEAEs by Preferred Term 34
6.3.1.3 TEAEs by System Organ Class, Preferred Term, and Severity 34
6.3.2 Solicited Adverse Reactions 34
6.3.3 Clinical Laboratory Evaluations 36
6.3.4 Vital Sign Measurements 36
6.4 IMMUNOGENICITY ANALYSIS 37
6.4.1 Immunogenicity Assessments 38
6.4.2 Phase 1/2 38
6.4.2.1 Primary Analysis of Immunogenicity Endpoints 38
6.4.2.2 Secondary Analysis of Immunogenicity Endpoints 39
6.4.2.3 Exploratory Analysis of Immunogenicity 39
6.4.3 Phase 2 Northern Hemisphere and Phase 2 Extension 40
6.4.3.1 Primary Analysis of Immunogenicity Endpoints 40
6.4.3.2 Secondary Analysis of Immunogenicity Endpoints 40
6.4.3.3 Exploratory Analysis of Immunogenicity Endpoints 41
6.5 ANALYSIS OF INFLUENZA INFECTION 41
6.6 INTERIM ANALYSIS 42

7 CHANGES FROM PLANNED ANALYSES IN PROTOCOL 43

8 REFERENCES 43

9 LIST OF APPENDICES 44

9.1 APPENDIX A STANDARDS FOR SAFETY AND IMMUNOGENICITY VARIABLE DISPLAY IN TFLS 44
9.2 APPENDIX B ANALYSIS VISIT WINDOWS FOR SAFETY AND IMMUNOGENICITY ANALYSIS44

9.3 APPENDIX C IMPUTATION RULES FOR MISSING PRIOR/CONCOMITANT MEDICATIONS AND NON-STUDY VACCINATIONS....45
9.4 APPENDIX D IMPUTATION RULES FOR MISSING AE DATES....46
9.5 APPENDIX E SCHEDULE OF EVENTS....48
9.5.1 Phase 1/2 - Schedule of Events....48
9.5.2 Phase 2 NH - Schedule of Events....51
9.5.3 Phase 2 Extension - Schedule of Events....54
9.6 APPENDIX F SEVERITY GRADING OF LABORATORY ABNORMALITIES....57
9.7 APPENDIX G SEVERITY GRADING OF VITAL SIGN ABNORMALITIES....59

List of Abbreviations

Abbreviation	Definition
AE	adverse event
AESI	adverse event of special interest
ANCOVA	analysis of covariance
AR	adverse reaction
BMI	body mass index
BUN	blood urea nitrogen
BP	blood pressure
CDC	United States Centers for Disease Control and Prevention
CI	confidence interval
COVID-19	coronavirus disease 2019
CRO	contract research organization
CSP	clinical study protocol
CSR	clinical study report
DBP	data blinding plan
DHHS	Department of Health and Human Services
DSMB	Data and Safety Monitoring Board
eCRF	electronic case report form
eDiary	electronic diary
EoS	end of study
FAS	Full Analysis Set
Fc	fragment crystallizable
FDA	Food and Drug Administration
FIH	first-in-human
FSH	follicle stimulating hormone
GM	geometric mean
GMFR	geometric mean fold rise
GMT	geometric mean titer
GMTr	ratio of geometric mean titer
HA	hemagglutinin
HAI	hemagglutination inhibition
IA	interim analysis
ILI	influenza-like illness
IP	investigational product
IST	internal safety team
LLOQ	lower limit of quantification
MAAE	medically attended adverse event
max	maximum
MedDRA	Medical Dictionary for Regulatory Activities
min	minimum
MN	microneutralization
mRNA	messenger ribonucleic acid
NH	Northern Hemisphere

Abbreviation	Definition
NP	nasopharyngeal
PP	per-protocol
PT	preferred term
RT-PCR	reverse transcriptase polymerase chain reaction
SAE	serious adverse event
SAP	statistical analysis plan
SAS	Statistical Analysis System
SD	standard deviation
SoE	schedule of events
SOC	system organ class
TEAE	treatment-emergent adverse event
ULOQ	upper limit of quantification
WHO	World Health Organization
WHODrug Global	World Health Organization drug dictionary

1 Introduction

This statistical analysis plan (SAP), which describes the planned analyses for Study mRNA-1010-P101, is based on the most recent approved clinical study protocol (CSP), Amendment 4, dated 03-Feb-2022, and the most recent approved electronic case report form (eCRF), dated 01-Mar-2022.

In addition to the information presented in the statistical analysis plan section of the protocol (Section 9), which provides the principal features of analyses for this study, this SAP provides statistical analysis details/data derivations. It also documents modifications or additions to the analysis plan that are not “principal” in nature and result from information that was not available at the time of protocol finalization.

Study mRNA-1010-P101 is a Phase 1/2, randomized, stratified, observer-blind, dose-ranging study to evaluate the safety, reactogenicity, and immunogenicity of messenger ribonucleic acid (mRNA)-1010 seasonal influenza vaccine in healthy adults 18 years and older. Study design and analyses for Phase 1/2, and Phase 2 Northern Hemisphere, and Phase 2 Extension parts of the study will be described separately in the SAP, unless otherwise specified.

PPD Biostatistics and Programming team, designee of Moderna Biostatistics and Programming department, will perform the statistical analysis of the safety, reactogenicity, and immunogenicity data; Statistical Analysis System (SAS) Version 9.4 or higher will be used to generate all statistical outputs (tables, figures, listings, and datasets). The SAP will be finalized and approved prior to the primary analysis clinical database lock and treatment unblinding for the study. If the methods in this SAP differ from the methods described in the protocol, the SAP will prevail.

In this document, study vaccination, injection of investigational product (IP)/ investigational vaccine, and injection are used interchangeably. The Phase 2 Northern Hemisphere part of the study will be referred to as Phase 2 NH, and previous flu vaccination status will be referred to as vaccination status throughout the SAP.

2 Study Objectives

2.1 Phase 1/2

2.1.1 Primary Objectives

The primary objectives are the following:

- To evaluate the safety and reactogenicity of 3 dose levels (50, 100, and 200 µg) of mRNA-1010 vaccine administered as a single dose.
- To evaluate the humoral immunogenicity of 3 dose levels (50, 100, and 200 µg) of mRNA-1010 vaccine administered as a single dose against vaccine-matched influenza A and B strains at Day 29.

2.1.2 Secondary Objectives

The secondary objective is the following:

- To evaluate the humoral immunogenicity of 3 dose levels (50, 100, and 200 µg) of mRNA-1010 vaccine administered as a single dose against vaccine-matched influenza A and B strains at all evaluable humoral immunogenicity time points.

2.1.3 Exploratory Objectives

[redacted]

2.2 Phase 2 Northern Hemisphere and Phase 2 Extension

2.2.1 Primary Objectives

The primary objectives are the following:

- To evaluate the humoral immunogenicity of mRNA-1010 vaccine relative to that of an active comparator against vaccine-matched influenza A and B strains at Day 29.
- To evaluate the safety and reactogenicity of mRNA-1010 vaccine.

2.2.2 Secondary Objectives

The secondary objective is the following:

- To evaluate the humoral immunogenicity of each vaccine group against vaccine-matched influenza A and B strains at Day 29.

2.2.3 Exploratory Objectives

[redacted]

3 Study Endpoints

3.1 Phase 1/2

3.1.1 Primary Endpoints

The primary safety objectives will be evaluated by the following safety endpoints:

- Frequency and grade of each solicited local and systemic reactogenicity adverse reaction (AR) during a 7-day follow-up period after vaccination.
- Frequency and severity of any unsolicited adverse events (AEs) during the 28-day follow-up period after vaccination.
- Frequency of any serious adverse events (SAEs), AE of special interest (AESI), and medically attended adverse events (MAAEs) from Day 1 to Day 181/end of study (EoS).
- Safety laboratory abnormalities through 7 days after vaccination.

The primary immunogenicity objectives will be evaluated by:

- Geometric mean titer (GMT) and geometric mean fold rise (GMFR) at Day 29 compared with Day 1 (baseline) and percentage of participants with seroconversion, defined as a Day 29 titer ≥ 1:40 if baseline is < 1:10 or a 4-fold or greater rise if baseline is ≥ 1:10 in anti-hemagglutinin (HA) antibodies measured by hemagglutination inhibition (HAI) assay.

3.1.2 Secondary Endpoints

The secondary objective will be evaluated by the following endpoints:

- GMT and GMFR of anti-HA antibodies as measured by HAI or microneutralization (MN) assays at all evaluable humoral immunogenicity time points compared with Day 1 (baseline).

3.1.3 Exploratory Endpoints

[redacted]

3.2 Phase 2 Northern Hemisphere and Phase 2 Extension

3.2.1 Primary Endpoints

The primary safety objectives will be evaluated by the following safety endpoints:

- Frequency and grade of each solicited local and systemic reactogenicity ARs during a 7-day follow-up period after vaccination.
- Frequency and severity of any unsolicited AEs during the 28-day follow-up period after vaccination.

- Frequency of any SAEs, AESIs, and MAAEs from Day 1 through Day 181/EoS.

The primary immunogenicity objectives will be evaluated by:

- GMT of anti-HA antibodies at Day 29 as measured by HAI assay.
- Proportion of participants reaching seroconversion at Day 29 as measured by HAI assay. Seroconversion is defined as:
    1. If LLOQ is 1:10, a Day 29 titer ≥ 1:40 if baseline is < 1:10 or a 4-fold or greater rise if baseline is ≥ 1:10 in anti- HA antibodies measured by HAI assay.
    2. If LLOQ is greater than 1:10 (e.g. 1:14), a Day 29 titer ≥ 4 times of LLOQ if baseline is < LLOQ or a 4-fold or greater rise if baseline is ≥ LLOQ in anti- HA antibodies measured by HAI assay.

3.2.2 Secondary Endpoints

The secondary objective will be evaluated by the following endpoints:

- The frequency of participants with anti-HA antibodies seroconversion as measured by HAI assay, and the frequency of participants with an anti-HA antibodies titer ≥ 1:40 as measured by HAI assay at Day 29.
- GMFR of anti-HA antibodies comparing Day 29 to Day 1 (baseline) as measured by HAI assay.

3.2.3 Exploratory Endpoints

[redacted]

- GMT, GMFR of anti-HA antibodies, and frequency of participants with anti-HA antibodies titers ≥ 1:40 as measured by assays such as MN assays or alternative methods at Days 91 and 181/EoS.
- Frequency, specificities, or other endpoints to be determined for further characterization of immune responses.

4 Study Design

4.1 Overall Study Design

This is a first-in-human (FIH), Phase 1/2, randomized, observer-blind, dose-ranging study in healthy adult participants ≥ 18 years of age. The study comprises 3 parts: Phase 1/2, Phase 2 NH, and Phase 2 Extension. The study will screen and enroll healthy (Phase 1/2) and medically stable (Phase 2 NH and Phase 2 Extension) adults ≥ 18 years of age. All participants will participate in a screening period (up to 28 days before Day 1), treatment period (single dose of vaccine on Day 1), and follow-up period (up to 6 months after vaccination). The vaccination groups and dose level that will be evaluated in each part of the study is are presented in Table 1.

Table 1 Vaccination Groups and Dose Levels

	Vaccination Group	Investigational Product	mRNA or HA (µg)	Total Dose (µg)	Number of Participants: Initial Stage	Number of Participants: Expansion Stage	Number of Participants: Total
**Phase 1/2**	1	mRNA-1010	12.5	50	9	36	45
	2	mRNA-1010	25	100	9	36	45
	3	mRNA-1010	50	200	9	36	45
	4	Placebo	–	–	9	36	45
	**Total**				**36**	**144**	**180**
**Phase 2 NH**	**Vaccination Group**	**Investigational Product**	**mRNA or HA (µg)**	**Total Dose (µg)**	**Number of Participants**		
	1	mRNA-1010	6.25	25	150		
	2	mRNA-1010	12.5	50	150		
	3	mRNA-1010	25	100	150		
	4	Active comparator	15	60	50		
	**Total**				**500**		

	Vaccination Group	Investigational Product	Total Dose (µg)[1]	Number of Participants
Phase 2 Extension	1	mRNA-1010	[redacted]	50
	2	mRNA-1010	[redacted]	50
	3	mRNA-1010	[redacted]	50
	4	Active comparator	60	50
	Total			200

Abbreviations: HA = hemagglutinin; mRNA = messenger RNA; NA = not applicable; NH = Northern Hemisphere.
[1] For Phase 2 Extension, mass percent in mRNA-1010 is 25% for each of the 4 HAs.

4.1.1 Phase 1/2

The Phase 1/2 part of the study will be a randomized, observer-blind, dose-ranging, placebo-controlled study to evaluate the safety, reactogenicity, and immunogenicity of mRNA-1010 vaccine in healthy adult participants ≥ 18 years of age.

The mRNA-1010 vaccine to be tested includes mRNAs that encode for the surface glycoprotein HA of the following influenza virus strains recommended by the World Health Organization (WHO) for 2021 Southern Hemisphere cell- or recombinant-based vaccines:

- A/Wisconsin/588/2019(H1N1)pdm09
- A/Hong Kong/45/2019(H3N2)
- B/Washington/02/2019 (B/Victoria lineage)
- B/Phuket/3073/2013 (B/Yamagata lineage)

Three dose levels (50, 100, and 200 µg) of mRNA-1010 vaccine (vaccination groups 1 through 3) will be evaluated. Vaccination group 4 will receive placebo. Overall, approximately 180 participants will be randomized in a 1:1:1:1 ratio to receive mRNA-1010 50 µg, mRNA-1010 100 µg, mRNA-1010 200 µg, or placebo, with approximately 45 participants randomized to each vaccination group. The vaccination groups and corresponding dose levels are presented in Table 1. Each vaccination group will have 2 stages: initial stage and expansion stage. The study schema is illustrated in Figure 1.

4.1.1.1 Stage 1, Initial Stage

A total of 36 participants (9 participants in each vaccination group) will be randomly assigned in the initial stage of the study. The internal safety team (IST) will perform a blinded review of all safety data up to 7 days after vaccination from the 36 participants in the initial stage.

4.1.1.2 Stage 2, Expansion Stage

After the IST confirms that no pause rules have been met in the 36 participants in the initial stage, enrollment will begin in the expansion stage. A total of 144 participants (36 participants in each vaccination group) will be randomly assigned in the expansion stage of the study. Randomization in the expansion stage will be stratified by age (18 to 49 years versus ≥ 50 years) and will be balanced across the 2 age groups within each vaccination group.

Figure 1 Phase 1/2 Study Schema

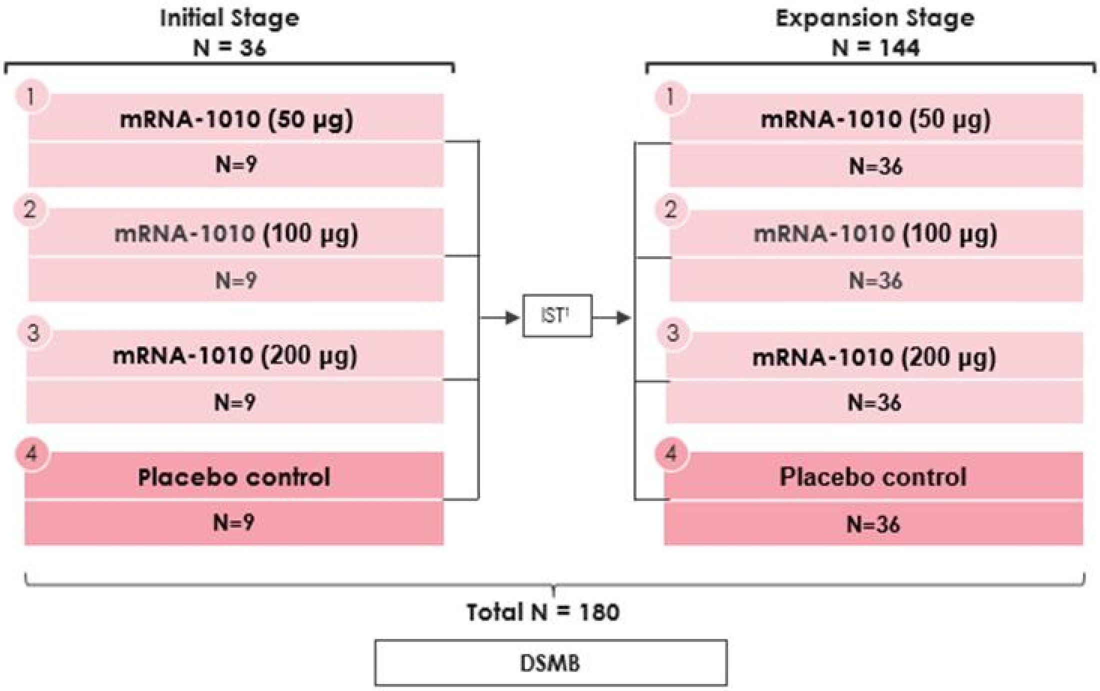

Abbreviations: DSMB = Data and Safety Monitoring Board; IST = internal safety team; N = number of participants.
[1] Expansion from initial stage to expansion stage is triggered by the IST.

4.1.2 Phase 2 Northern Hemisphere

The Phase 2 NH part of the study will be a randomized, observer-blind, dose-ranging, active-controlled study to evaluate the immunogenicity, reactogenicity, and safety of mRNA-1010 vaccine in medically stable adults 18 years and older.

The mRNA-1010 vaccine to be tested, includes mRNAs that encode for the surface glycoprotein HAs of the following influenza virus strains recommended by the WHO for 2021-2022 NH cell- or recombinant-based vaccines:

- A/Wisconsin/588/2019(H1N1)pdm09
- A/Cambodia/e0826360/2020 (H3N2)
- B/Washington/02/2019 (B/Victoria lineage)
- B/Phuket/3073/2013 (B/Yamagata lineage) I

Three dose levels (25, 50, and 100 µg) of mRNA-1010 vaccine (vaccination groups 1 through 3) will be evaluated. Vaccination group 4 will receive an active comparator (licensed quadrivalent seasonal influenza vaccine). Phase 2 NH part of the study will enroll and randomize approximately 500 participants in a 3:3:3:1 ratio to 1 of 4 vaccination groups to receive a single dose of mRNA-1010 at different dose levels (25, 50, 100 µg total mRNA) or a single dose of an active comparator. Randomization will be stratified by age categories (18 to < 50 years, 50 to < 65 years, or ≥ 65 years) and vaccination status in the previous flu season (received or not received). The study schema is illustrated in Figure 2.

Figure 2 Phase 2 Northern Hemisphere Study Schema

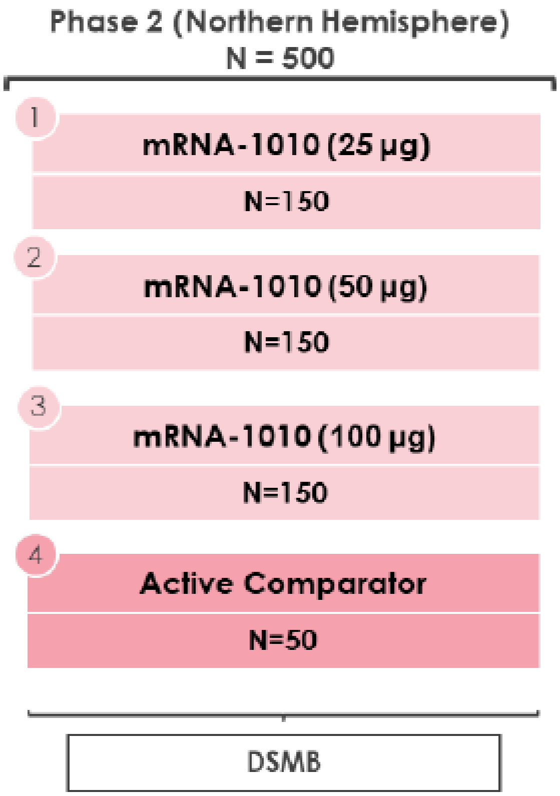

Abbreviations: DSMB = Data and Safety Monitoring Board; N = number of participants.

4.1.3 Phase 2 Extension

The Phase 2 Extension part of the study follows a similar design as Phase 2 NH, and the same influenza virus strains recommended by the WHO for 2021-2022 NH cell- or recombinant-based vaccines will be tested:

- A/Wisconsin/588/2019(H1N1)pdm09
- A/Cambodia/e0826360/2020 (H3N2)
- B/Washington/02/2019 (B/Victoria lineage)
- B/Phuket/3073/2013 (B/Yamagata lineage) I

Three dose levels [redacted] of mRNA-1010 vaccine (vaccination groups 1 through 3) will be evaluated. Vaccination group 4 will receive an active comparator (licensed quadrivalent seasonal influenza vaccine). Phase 2 Extension part of the study will enroll and randomize approximately 200 participants in a 1:1:1:1 ratio to 1 of 4 vaccination

groups to receive a single dose of mRNA-1010 at different dose levels [redacted] or a single dose of an active comparator. Randomization will be stratified by age categories (18 to < 50 years or ≥ 50 years). The study schema is illustrated in Figure 3.

Figure 3 Phase 2 Extension Study Schema

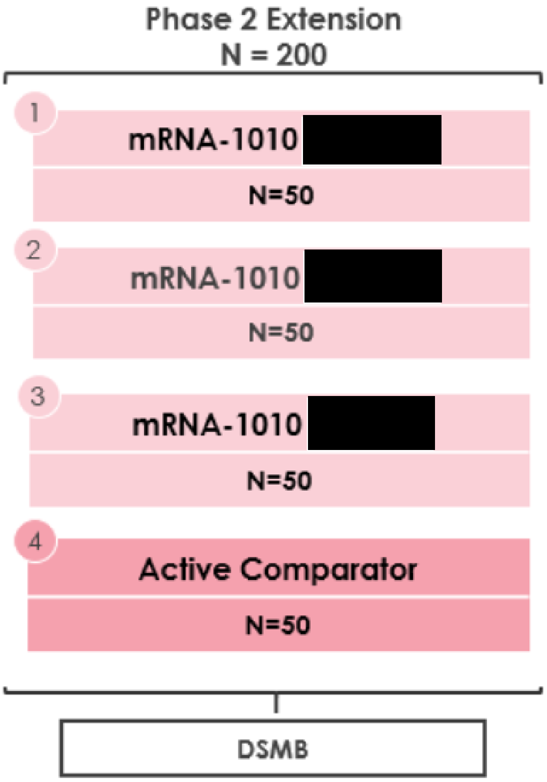

Abbreviations: DSMB = Data and Safety Monitoring Board; N = number of participants

4.2 Statistical Hypotheses

No formal hypotheses will be tested for Phase 1/2, Phase 2 NH, and Phase 2 Extension.

4.3 Sample Size and Power

The sample size for this trial is not driven by statistical assumptions for formal hypothesis testing. The number of proposed participants is considered sufficient to provide a descriptive summary of the safety and immunogenicity of different dose levels of mRNA-1010.

4.3.1 Phase 1/2

Approximately 180 participants will be randomized to 4 vaccination groups in a 1:1:1:1 ratio as specified in Table 1. With 45 participants receiving an investigational vaccine, there is a probability of approximately 90% to observe at least 1 participant with an AE, if the true incidence rate of the AE is 5%; if the true incidence rate is 10%, then the probability to observe it will be approximately 99%.

4.3.2 Phase 2 Northern Hemisphere

Approximately 500 participants will be randomly assigned in a 3:3:3:1 ratio to the mRNA-1010 25, 50, 100 µg groups, or the active comparator group as specified in Table 1. A total of 450 participants will receive the mRNA-1010 vaccine (150 participants at each dose level). A sample size of 150 participants at each dose level has at least a 95% (or 99%) probability to observe at least 1 participant with an AE at a true 2% (or 3%) AE rate.

4.3.3 Phase 2 Extension

Approximately 200 participants will be randomly assigned in a 1:1:1:1 ratio to the mRNA-1010 [redacted] groups, or the active comparator Group. A total of 150 participants will receive the mRNA-1010 vaccine (50 participants at each dose level). With 50 participants receiving an investigational vaccine, there is a probability of approximately 92% to observe at least 1 participant with an AE, if the true incidence rate of the AE is 5%; if the true incidence rate is 10%, then the probability to observe it will be approximately 99%.

4.4 Randomization

4.4.1 Phase 1/2

Approximately 180 participants will be randomized in a 1:1:1:1 ratio to receive either mRNA-1010 50 µg, mRNA-1010 100 µg, mRNA-1010 200 µg, or placebo, with approximately 45 participants randomized to each vaccination group. A total of 36 participants (9 participants in each vaccination group) will be randomly assigned in the initial stage of the study. A total of 144 participants (36 participants in each vaccination group) will be randomly assigned in the expansion stage of the study. Randomization in the expansion stage will be stratified by age (18 to 49 years versus ≥ 50 years) and will be balanced across the 2 age groups within each vaccination group.

4.4.2 Phase 2 Northern Hemisphere

Approximately 500 participants will be randomized in a 3:3:3:1 ratio to receive either mRNA-1010 25 µg, mRNA-1010 50 µg, mRNA-1010 100 µg, , or a single dose of a licensed quadrivalent seasonal influenza vaccine (active comparator). Randomization to individual vaccination groups will proceed with parallel randomization among the 3 dose levels of mRNA-1010 (25, 50, and 100 µg total mRNA; vaccination groups 1 to 3) and the active comparator (vaccination group 4). Randomization will be stratified by age categories (18 to < 50 years, 50 to < 65 years, or ≥ 65 years) and vaccination status in the previous flu season (received or not received).

4.4.3 Phase 2 Extension

Approximately 200 participants will be randomly assigned in a 1:1:1:1 ratio to receive either [redacted], or licensed quadrivalent seasonal influenza vaccine (active comparator), with approximately 50 participants randomly assigned to each mRNA-1010 vaccination group and 50 participants to the Active Comparator Group. Randomization will be stratified by age (18 to < 50 years or ≥ 50 years), with at least 30% of participants in each dose group being ≥ 50 years of age.

4.5 Blinding and Unblinding

This study is an observer-blind study. The investigator, study staff, study participants, site monitors, and sponsor personnel (or its designees) will be blinded to the IP administered until the study database is locked and unblinded, with certain exceptions please refer to Section 9.1 of the protocol for details.

A Day 29 IA is planned in this study for each of the Phase 1/2, Phase 2 NH, and Phase 2 Extension parts, separately. All relevant data to each IA will be cleaned (i.e., data that are as clean as possible).

The IA will be performed by a separate team of unblinded programmers and statisticians. Except for a limited number of Sponsor and contract research organization (CRO) personnel who will be unblinded to perform the IA, the study site staffs, investigators, study monitors, and participants will remain blinded until after the final database lock for final analysis. Please refer to Section 6.6 and the data blinding plan (DBP) for more details on the IA.

The unblinded IA results will be shared with the Data and Safety Monitoring Board (DSMB).

5 Analysis Populations

For Phase 1/2, Phase 2 NH, and Phase 2 Extension, the following analysis sets are defined: Randomization Set, Full Analysis Set (FAS), Per-protocol (PP) Set, Safety Set, and Solicited Safety Set.

5.1 Randomization Set

The Randomization Set consists of all participants who are randomly assigned. Participants will be analyzed according to the vaccination group to which they were randomized.

5.2 Full Analysis Set (FAS)

The FAS consists of all randomly assigned participants who receive the IP. Participants will be analyzed according to the vaccination group to which they were randomized.

5.3 Per-protocol (PP) Set

The PP Set consists of all participants in the FAS who comply with the injection schedule, comply with the timing of immunogenicity blood sampling (see definition of criterion below), do not have influenza infection at baseline through Day 29 (as documented by RT-PCR testing), and have no major protocol deviations that impact the immune response. The PP Set will be used as the primary analysis set for analyses of immunogenicity unless otherwise specified. Participants will be analyzed according to the vaccination group to which they were randomized.

Subjects that comply with the timing of immunogenicity blood sampling are required to have a baseline assessment, have at least one post-injection assessment, and have a Day 29 assessment that is within 22 days to 36 days after injection (+7 or -7 days of Day 29).

Subjects with dosing error will be considered as having a protocol deviation. However, the determination of whether to include/exclude subjects from the PP Set due to dosing error will be based on the dosage difference (in µg) between the actual dose received and the randomized dose. The PP Set exclusion criteria for subjects with dosing error are described in Table 2. For Phase 2 NH and Phase 2 Extension, subjects that are randomized to the

active comparator arm but received less than 40 µg (< 40 µg) of the active comparator or any amount of mRNA-1010 will be excluded from the PP Set.

Table 2 PP Set Exclusion Criteria for Dosing Errors

		Randomized Dose			
Phase 1/2	**Actual Dose Received**	50 µg	100 µg	200 µg	Placebo (0 µg)
	50 µg		Y	Y	Y
	100 µg	Y		Y	Y
	200 µg	Y	Y		Y
	> 0 - 25 µg	Y	Y	Y	Y
	> 25 - 50 µg		Y	Y	Y
	> 50- 75 µg		Y	Y	Y
	> 75 - 100 µg	Y		Y	Y
	> 100 - 125 µg	Y		Y	Y
	> 125 -150 µg	Y	Y	Y	Y
	> 150 – 175 µg	Y	Y	Y	Y
	> 175 -200 µg	Y	Y		Y
	> 200 -225 µg	Y	Y		Y
	> 225 µg	Y	Y	Y	Y
	Placebo (0 µg)	Y	Y	Y	
Phase 2 NH		**Randomized Dose**			
	**Actual Dose Received**	25 µg	50 µg	100 µg	Active Comparator[1]
	25 µg		Y	Y	Y
	50 µg	Y		Y	Y
	100 µg	Y	Y		Y
	> 0 - 25 µg		Y	Y	Y
	> 25 - 37.5 µg		Y	Y	Y
	> 37.5 - 50 µg	Y		Y	Y
	> 50- 75 µg	Y		Y	Y
	> 75 - 100 µg	Y	Y		Y
	> 100 - 125 µg	Y	Y		Y
	> 125 µg	Y	Y	Y	Y
	Active Comparator[1]	Y	Y	Y	
Phase 2 Extension		**Randomized Dose**			
	**Actual Dose Received**	[redacted]	[redacted]	[redacted]	Active Comparator[1]
	[redacted]		Y	Y	Y
	[redacted]	Y		Y	Y

	[redacted]	Y	Y		Y
	[redacted]		Y	Y	Y
	[redacted]		Y	Y	Y
	[redacted]	Y		Y	Y
	[redacted]	Y		Y	Y
	[redacted]	Y	Y		Y
	[redacted]	Y	Y		Y
	[redacted]	Y	Y	Y	Y
	Active Comparator[1]	Y	Y	Y	

Note: Y = Excluded from PP Set.
[1] Subjects who are randomized to receive active comparator should receive at least 40 µg (≥ 40 µg) of the active comparator and 0 µg of mRNA-1010 to be included in the PP Set. Subjects in the active comparator arm that received < 40 µg or any amount of mRNA-1010 will be excluded from the PP Set.

5.4 Safety Set

The Safety Set consists of all randomly assigned participants who receive the IP. The Safety Set will be used for analysis of safety except for the solicited ARs. Participants will be included in the vaccination group corresponding to the vaccination they actually received according to the following rules:

For Phase 1/2:

- mRNA-1010 50 µg group: If the received dose of mRNA-1010 is > 0 µg and ≤ 75 µg
- mRNA-1010 100 µg group: If the received dose of mRNA-1010 is > 75 µg and ≤ 150 µg
- mRNA-1010 200 µg group: If the received dose of mRNA-1010 is > 150 µg
- Placebo group: If the received dose of mRNA-1010 is 0 µg

For Phase 2 NH:

- mRNA-1010 25 µg group: If the received dose of mRNA-1010 is > 0 µg and ≤ 37.5 µg
- mRNA-1010 50 µg group: If the received dose of mRNA-1010 is > 37.5 µg and ≤ 75 µg
- mRNA-1010 100 µg group: If the received dose of mRNA-1010 is > 75 µg

- Active comparator group: If the received dose of active comparator is ≥ 40 µg and mRNA-1010 is 0 µg .

For Phase 2 Extension

- mRNA-1010 [redacted] group: If the received dose of mRNA-1010 is [redacted]
- mRNA-1010 [redacted] group: If the received dose of mRNA-1010 is [redacted]
- mRNA-1010 [redacted] group: If the received dose of mRNA-1010 is [redacted]
- Active comparator group: If the received dose of active comparator is ≥ 40 µg and mRNA-1010 is 0 µg .

5.5 Solicited Safety Set

The Solicited Safety Set consists of all participants in the Safety Set who contribute any solicited AR data, i.e., have at least one post-baseline solicited safety assessment. The Solicited Safety Set will be used for the analyses of solicited ARs and participants will be included in the vaccination group corresponding to the study injection they actually received.

6 Statistical Analysis

6.1 General Considerations

For Phase 1/2, Phase 2 NH, and Phase 2 Extension, the Schedules of Events (SoE) are provided in Appendix E.

General considerations for analyses will be applied to Phase 1/2, Phase 2 NH, and Phase 2 Extension, unless otherwise specified. All analyses will be conducted using SAS Version 9.4 or higher. Statistical outputs (tables, figures, listings, and datasets) will refer study participants as subjects and will use injection of IP and injection interchangeably.

For the summary statistics of all numerical variables unless otherwise specified, the display precision will follow programming standards. Please see Appendix A for variable display standards.

When count data are presented, the percentage will be suppressed when the count is zero in order to draw attention to the non-zero counts. A row denoted “Missing” will be included in count tabulations where specified on the shells to account for dropouts and missing values. The denominator for all percentages will be the number of participants in that vaccination group within the analysis set of interest, unless otherwise specified.

Continuous variables will be summarized using the following descriptive summary statistics: the number of participants (n), mean, standard deviation (SD), median, minimum (min), and maximum (max).

Categorical variables will be summarized using counts and percentages.

Baseline value is defined as the most recent non-missing measurement (scheduled or unscheduled) collected before injection, unless otherwise specified. For immunogenicity tests and nasopharyngeal (NP) swab tests, the baseline is defined as the most recent non-missing result/measurement (scheduled or unscheduled) collected before or on the date (and time, if available) of injection (Day 1).

Study day relative to injection will be calculated as below:

- Study day prior to injection will be calculated as: date of assessment/event – date of injection.
- Study day on or after the date of injection will be calculated as: date of assessment/event – date of injection + 1.

For calculation regarding antibody levels/titers, antibody values reported as below lower limit of quantification (LLOQ) will be replaced by 0.5 × LLOQ. Values that are greater than the upper limit of quantification (ULOQ) will be converted to the ULOQ. Missing results will not be imputed.

The following **analysis periods for safety analyses** will be used in this study:

- 7 days following vaccination: this period includes the day of vaccination and 6 subsequent days. This analysis period will be used for solicited local and systemic AR that occur during this time.
- Up to 28 days following vaccination: this period starts from the day of vaccination and spans 28 days to include the day of vaccination and 27 subsequent days. This

analysis period will be used as the primary analysis period for safety analyses including unsolicited AE, except for solicited AR, unless specified otherwise.

- Overall period: this analysis period starts on Day 1 and continues through the earliest of the following: study completion, discontinuation from the study, or death.

Unscheduled visits: Unscheduled visit measurements will be included in analysis as follows:

- In scheduled visit windows per specified visit windowing rules.
- In the derivation of baseline/last on-treatment values.
- In the derivation of max/min on-treatment values and max/min change from baseline values for safety analyses.
- In individual participant data listings as appropriate.

Visit windowing rules: The analysis visit windows for protocol-defined visits are provided in Appendix B.

Incomplete/missing data:

- Imputation rules for missing prior/concomitant medications, non-study vaccinations and procedures are provided in Appendix C.
- Imputation rules for missing AE dates are provided in Appendix D.
- For laboratory assessments (Phase 1/2 only), if majority of results are indefinite, imputation of these values will be considered. If the laboratory results are reported as below the LLOQ (e.g., < 0.1), the numeric values will be imputed by 0.5 × LLOQ in the summary. If the laboratory results are reported as greater than the ULOQ (e.g., > 3000), the numeric values will be imputed by ULOQ in the summary.
- Other incomplete/missing data will not be imputed, unless otherwise specified.

6.1.1 Phase 1/2

Age groups:

The following age groups will be used for summary purposes:

- Overall
- Age: ≥ 18 to < 50 years
- Age ≥ 50 years

Vaccination groups:

The following vaccination groups will be used for summary purposes:

- mRNA-1010 50 µg
- mRNA-1010 100 µg
- mRNA-1010 200 µg
- Placebo

For Phase 1/2, all analyses and data summaries/displays will be provided by vaccination group (and then by age group) using appropriate analysis population, unless otherwise specified. Summaries may also contain a display for all mRNA-1010 vaccination groups (Total mRNA-1010 group) or all vaccination groups, including placebo (Overall group).

6.1.2 Phase 2 Northern Hemisphere

Age groups:

The following age groups will be used for summary purposes:

- Overall
- Age: ≥ 18 to < 50 years
- Age ≥ 50 to < 65 years
- Age ≥ 65 years

Vaccination status:

The following previous flu vaccination status may be used for summary purposes:

- Received season flu vaccine in the previous season
- Not received season flu vaccine in the previous season

Vaccination groups:

The following vaccination groups will be used for summary purposes:

- mRNA-1010 25 µg
- mRNA-1010 50 µg
- mRNA-1010 100 µg
- Active comparator

For Phase 2 NH, all analyses and data summaries/displays will be provided by vaccination group (and then by age group) using appropriate analysis population, unless otherwise specified. Summaries may also contain a display for all mRNA-1010 vaccination groups (Total mRNA-1010 group) or all vaccination groups, including active comparator (Overall group). Additional summaries/displays based on vaccination status will be provided for immunogenicity analyses only.

6.1.3 Phase 2 Extension

Age groups:

The following age groups will be used for summary purposes:

- Overall
- Age: ≥ 18 to < 50 years
- Age ≥ 50 years

Vaccination groups:

The following vaccination groups will be used for summary purposes:

- mRNA-1010 [redacted]
- mRNA-1010 [redacted]
- mRNA-1010 [redacted]
- Active comparator

For Phase 2 Extension, all analyses and data summaries/displays will be provided by vaccination group (and then by age group) using appropriate analysis population, unless otherwise specified. Summaries may also contain a display for all mRNA-1010 vaccination groups (Total mRNA-1010 group) or all vaccination groups, including active comparator (Overall group).

6.2 Background Characteristics

The following description of background characteristics analyses will be applied to Phase 1/2, Phase 2 NH, and Phase 2 Extension, unless otherwise specified.

6.2.1 Participant Disposition

The number and percentage of participants in the following categories will be summarized by vaccination group and then by age group) as defined in Section 6.1 based on Randomization Set:

- Randomization Set
- FAS
- PP Set
- Safety Set
- Solicited Safety Set

The percentage will be based on participants in that vaccination group within the Randomization Set (as randomized).

A summary of reasons for participants who are in the Randomization Set but excluded from PP Set will also be provided.

The number of participants in the following categories will be summarized based on participants screened:

- Number of participants screened
- Number and percentage of screen failure participants and the reason for screen failure

The percentage of participants who screen failed will be based on the number of participants screened. The percentage of participants for each reason for screen failure will be based on the number of participants who screen failed.

The number and percentage of participants in each of the following disposition categories will be summarized by vaccination group (and then by age group) based on the Randomization Set:

- Randomized by site
- Received injection

- Completed study
- Prematurely discontinued the study and the reason for discontinuation

The denominator for all percentages will be the number of participants in the vaccination groups within the Randomized Set.

A participant disposition listing will be provided, including informed consent, participants who completed the study injection schedule, participants who completed study, participants who discontinued from participation in the study, with reasons for discontinuation. A separate listing will be provided for screen failure participants with reasons for screen failure.

For all study parts, a participant who completed the final visit on Day 181 (Month 6), defined as 6 months after the study vaccination on Day 1, is considered to have completed the study.

6.2.2 Demographics

Descriptive statistics will be calculated for the following continuous demographic and baseline characteristics: age (years), weight (kg), height (cm), and body mass index (BMI) (kg/m^2). Number and percentage of participants will be provided for categorical variables such as sex, race, and ethnicity. The summaries will be presented by vaccination group (and then by age group) as defined in Section 6.1 based on the FAS, PP Set, and Safety Set. If the Safety Set differs from the Randomization Set (e.g., participants randomized but not received any study vaccination or participants received study vaccination other than the vaccination group they were randomized to), the analysis will also be conducted using the Randomization Set.

For screened failure participants, age (years), as well as sex, race, and ethnicity will be presented in a listing.

In addition, participants who are in the Randomization Set but with any inclusion and exclusion criteria deviation will also be provided in a listing.

6.2.3 Medical History

Medical history data will be coded by system organ class (SOC) and preferred term (PT) using the Medical Dictionary for Regulatory Activities (MedDRA).

The number and percentage of participants with any medical history will be summarized by SOC and PT based on the Safety Set. A participant will be counted only once for multiple events within each SOC and PT. SOCs will be displayed in internationally agreed order. PTs will be displayed in descending order of frequency of Total mRNA-1010 group/Overall group and then alphabetically within SOC.

Medical history data will be presented in a listing.

6.2.4 Prior and Concomitant Medications

Prior and concomitant medications and non-study vaccination will be coded using the World Health Organization drug dictionary (WHODrug Global). The summary of concomitant medications will be based on the Safety Set. Categorization of prior, concomitant, and post medications is summarized in Appendix C Table 5.

The number and percentage of participants using concomitant medications and non-study vaccination during the 7-day follow-up period (i.e., on the day of injection and the 6 subsequent days) and during the 28-day follow-up period after the injection (i.e., on the day of injection and the 27 subsequent days) will be summarized by vaccination groups (as defined in Section 6.1) as follows:

- Any concomitant medications and non-study vaccination within 7 days post-injection
- Any concomitant medications and non-study vaccination within 28 days post-injection
- Seasonal influenza vaccine within 28 days post-injection
- Antipyretic or analgesic medication within 28 days post-injection

A summary table of concomitant medications and non-study vaccination that continued or newly received on the date of injection through 28 days post-injection will be provided by PT in descending frequency in the Total mRNA-1010 group.

Medications taken to prevent or treat pain or fever will be collected in the electronic diary (eDiary), and summaries will be provided based on the Solicited Safety Set by vaccination group (and then by age group) as defined in Section 6.1, including within 7 days after injection, beyond 7 days after injection, and any time after injection.

Prior, concomitant, post medications, and non-study vaccination will be presented in a listing. Medications taken to prevent or treat pain or fever will also be presented in a listing.

Concomitant procedures will be presented in a listing.

6.2.5 Study Exposure

Study IP administration data as well as subjects with dosing error will be presented in a listing.

Study duration will be summarized since randomization and since the injection.

6.2.6 Major Protocol Deviations

Major protocol deviations are a subset of protocol deviations that may significantly impact the completeness, accuracy, or reliability of the study data or that may significantly affect a participant's rights, safety, or well-being. Major protocol deviations rules will be developed and finalized before database lock.

The number and percentage of the participants with each major protocol deviation type will be provided by vaccination group (and then by age group) as defined in Section 6.1 based on the Randomization Set.

Major protocol deviations will be presented in a listing.

6.2.7 COVID-19 Impact

A listing will be provided for the impact of coronavirus disease 2019 (COVID-19) on the execution of the study.

6.3 Safety Analysis

The following description of safety analyses will be applied to Phase 1/2, Phase 2 NH, and Phase 2 Extension, unless otherwise specified.
Safety and reactogenicity will be assessed by clinical review of all relevant parameters including solicited ARs (local and systemic), unsolicited AEs (including any clinical safety laboratory abnormalities for Phase 1/2), treatment-related AEs, severe AEs, SAEs, AESIs, MAAEs, AEs leading to withdrawal from study vaccine and/or study participation, vital signs measurement, and physical examination findings. Solicited ARs and unsolicited AEs will be coded by SOC and PT according to MedDRA. AESIs are defined and described in detail in the protocol. The Toxicity Grading Scale for Healthy Adult and Adolescent

Volunteers Enrolled in Preventative Vaccine Clinical Trials (Department of Health and Human Services [DHHS] 2007) is used in this study for solicited ARs as presented in the protocol.

All safety analyses will be based on the Safety Set, except summaries of solicited ARs, which will be based on the Solicited Safety Set. All safety analyses will be provided by vaccination group (and then by age group) unless otherwise specified.

6.3.1 Adverse Events

A treatment-emergent AE (TEAE) is defined as any event occurring during the study not before exposure to study vaccine or any event already present that worsens after exposure to study vaccine. Worsening of a pre-existing condition after vaccination will be reported as a new AE.

AEs will also be evaluated by the investigator for the coexistence of MAAE and/or AESI. MAAE is defined as an AE that leads to an unscheduled visit to a healthcare practitioner. AESI is an AE (serious or nonserious) of scientific and medical concern specific to the Sponsor's product or program, for which ongoing monitoring and immediate notification by the Investigator to the Sponsor are required. All AEs will be coded by SOC and PT using MedDRA.

Unsolicited AEs within 28 days after vaccination will be summarized by vaccination group. SAE, AESI, MAAE, and AE leading to discontinuation from the study up to 28 days after vaccination and overall period will be summarized by vaccination group. See Section 6.1 for definitions of analysis groups and analysis period.

All summary tables (except for the overall summary of AEs) for unsolicited TEAEs will be presented by SOC and/or PT with counts of participants included. SOCs will be displayed in internationally agreed order. PTs will be displayed in descending order of frequency of Total mRNA-1010 group and then alphabetically within SOC. When summarizing the number and percentage of participants with an event, participants with multiple occurrences of the same AE or a continuing AE will be counted once. Participants will be presented according to the highest severity in the summaries by severity, if participants reported multiple events under the same SOC and/or PT.

Percentages will be based upon the number of participants in the Safety Set within each vaccination group.

6.3.1.1 Incidence of Adverse Events

An overall summary of unsolicited TEAEs including the number and percentage of participants who experience the following will be presented:

- Any unsolicited TEAEs
- Any unsolicited serious TEAEs
- Any unsolicited fatal TEAEs
- Any unsolicited medically attended TEAEs
- Any unsolicited treatment-emergent AESI
- Any unsolicited TEAEs leading to discontinuation from participation in the study
- Any unsolicited severe TEAEs

The table will also include number and percentage of participants with unsolicited TEAEs that are treatment-related in each of the above categories.

In addition, separate listings containing individual participant AE data for unsolicited AEs, unsolicited TEAEs leading to discontinuation from participation in the study, SAEs, AESI, and medically attended TEAEs will be provided separately.

6.3.1.2 TEAEs by System Organ Class and Preferred Term

The following summary tables of TEAEs will be provided by SOC and PT using frequency counts and percentages (i.e., number and percentage of participants with an event):

- All unsolicited TEAEs
- All unsolicited TEAEs that are treatment-related
- All unsolicited serious TEAEs
- All unsolicited serious TEAEs that are treatment-related
- All unsolicited TEAEs leading to discontinuation from participation in the study
- All unsolicited severe TEAEs
- All unsolicited severe TEAEs that are treatment-related
- All unsolicited treatment-emergent AESI

- All unsolicited medically attended TEAEs
- All unsolicited medically attended TEAEs that are treatment-related

6.3.1.2 TEAEs by Preferred Term

Summary tables of all unsolicited TEAEs within 28 days after vaccination will be provided. PTs will be sorted in a descending order according to the frequency in Total mRNA-1010 group.

6.3.1.3 TEAEs by System Organ Class, Preferred Term, and Severity/Toxicity

The following summary tables of TEAEs within 28 days after vaccination will be provided by SOC, PT, and maximum severity (mild, moderate, severe) or toxicity (Grade 1 through Grade 5) using frequency counts and percentages:

- All unsolicited TEAEs
- All unsolicited TEAEs that are treatment-related

6.3.2 Solicited Adverse Reactions

An AR is any AE for which there is a reasonable possibility that the IP caused the AE. The term “Solicited Adverse Reactions” refers to selected signs and symptoms occurring after injection administration during a specified post-injection follow-up period (day of injection and 6 subsequent days). The solicited ARs are recorded by the participant in the eDiary. Any new safety information reported during safety telephone calls or at site visits (including a solicited reaction) that is not already captured in the eDiary will be described in the source documents as a verbally reported event. Any AR reported in this manner must be entered on the Reactogenicity eCRF and will be included in the evaluation of solicited ARs in addition to the eDiary data.

The occurrence and intensity of selected signs and symptoms are actively solicited from participants during a specified post-injection follow-up period (day of injection and 6 subsequent days), using a pre-defined checklist (i.e., solicited ARs). If a solicited local or systemic AR continues beyond 7 days post injection, the participant will be prompted daily to capture solicited local or systemic AR in the eDiary until no longer reported, not to exceed 28 days after vaccination.

The following local ARs will be solicited: injection site pain, injection site erythema (redness), injection site swelling/induration (hardness), and axillary (underarm) swelling or tenderness ipsilateral to the side of injection.

The following systemic ARs will be solicited: fever (an oral temperature greater than or equal to 38.0°C/100.4°F), headache, fatigue, myalgia (muscle aches all over the body), arthralgia (joint aches in several joints), nausea/vomiting, and chills.

The solicited ARs will be graded based on the grading scales presented in the protocol, modified from the Toxicity Grading Scale for Healthy Adult and Adolescent Volunteers Enrolled in Preventative Vaccine Clinical Trials (DHHS 2007). The investigator will assess Grade 4 (with exception of fever) solicited ARs recorded in eDiary or Reactogenicity CRF.

Analyses of solicited ARs will be provided by vaccination group (and then by age group) based on the Solicited Safety Set, unless otherwise specified. All solicited ARs (overall, local, and systemic) reported during the 7-day follow-up period after injection will be summarized. All solicited ARs will be considered causally related to injection.

The number and percentage of participants who reported each individual solicited local AR (has a severity grade of Grade 1 or greater) and solicited systemic AR (has a severity grade of Grade 1 or greater) during the 7-day follow-up period after injection or persisting beyond 7 days after injection will be tabulated by vaccination group and toxicity grade. The number and percentage of participants who reported each individual solicited AR will also be summarized by vaccination group, toxicity grade, and day of reporting based on the eDiary and Reactogenicity CRF. Analyses will be displayed for each age group.

A two-sided 95% exact confidence interval (CI) using the Clopper-Pearson method will be provided for the percentage of participants who reported or any solicited AR, any solicited local AR, or any solicited systemic AR.

The onset of individual solicited AR is defined as the time point after injection at which the respective solicited AR first occurred. The number and percentage of participants with onset of individual solicited AR within 7 days will be summarized by vaccination group and onset day relative to injection day (Day 1). Analyses will be displayed for each age group.

The number of days reporting each solicited AR will be summarized descriptively and categorically for the following time windows (1-2 days, 3-4 days, 5-6 days, and ≥7 days) by vaccination group (and then by age group). The number of days will be calculated as

the day(s) the solicited AR is reported within 7 days of injection including the day of injection, no matter if it is intermittent or continued. If the solicited AR continues beyond 7 days, the days a solicited AR is reported after 7 days will be included (e.g., an event that lasted 5 days in the first 7 days post injection and 3 days beyond 7 days post injection, the duration will be reported as 8 (5 + 3) days). In addition, the duration for each solicited AR that started within 7 days of injection will be summarized descriptively. The duration of the solicited AR will be calculated as the last day – the first day + 1.

Solicited ARs collected in the eDiary and those collected in the reactogenicity eCRF will be provided in a listing, and the maximum toxicity grade from eDiary and reactogenicity eCRF will be presented. All solicited ARs that continue beyond 7 days post injection will be presented in separate data listings.

6.3.3 Clinical Laboratory Evaluations

Safety laboratory testing will include hematology and serum chemistry (Phase 1/2 only).

All laboratory test results will be presented in the data listings. The results that are outside the reference ranges will be flagged in the data listings. The abnormalities meeting the toxicity grading criteria (Grade 3 or higher) in Appendix F in any safety laboratory (hematology and serum chemistry) will be listed separately.

For continuous hematology and serum chemistry parameters, the observed values and changes from baseline will be summarized at each visit by vaccination group as defined in Section 6.1. Hematology and chemistry toxicity grades will be summarized using a shift table from baseline to Day 8 by vaccination group.

A pregnancy test will be performed on all female participants of childbearing potential at the Screening Visit and before vaccine administration on Day 1 via point-of-care urine, and as needed at unscheduled visits (urine or blood pregnancy test based on the investigator's discretion). A follicle stimulating hormone (FSH) test may be performed at the Screening Visit (Day 0), as necessary and at the discretion of the investigator, to confirm postmenopausal status.

6.3.4 Vital Sign Measurements

Vital sign measurements, including systolic and diastolic blood pressures (BP), heart rate, respiratory rate, and body temperature, will be presented in a data listing. The values that are outside the reference ranges will be flagged in the data listing. The abnormalities

meeting the toxicity grading criteria (Grade 3 or higher) in Appendix G in any vital sign measurement will be listed separately.

Observed values and changes from pre-injection to post-injection at Day 1 for all vital sign measurements will be summarized by vaccination group as defined in Section 6.1. Shift from baseline in the toxicity grades to post-injection result at Day 1 will also be summarized by vaccination group.

6.4 Immunogenicity Analysis

The following description of immunogenicity analyses will be applied to Phase 1/2, Phase 2 NH, and Phase 2 Extension. Phase 2 NH and Phase 2 Extension will share the same analyses, unless otherwise specified.

The analyses of immunogenicity will be based on the PP Set and will be performed by vaccination group as defined in Section 6.1. If the number of participants in the FAS and PP Set differs (defined as the difference divided by the total number of participants in the PP Set) by more than 10% for each age group, supportive analyses of immunogenicity may be conducted using the FAS. The supportive analysis is required if the condition is met.

The GMT and geometric mean (GM) level will be calculated using the following formula:

$$2^{\left\{\frac{\sum_{i=1}^{n} log_2(t_i)}{n}\right\}}$$

where $t_1, t_2, \ldots, t_n$ are n observed immunogenicity titers or levels.

The GMFR measures the changes in immunogenicity titers or levels within participants. The GMFR will be calculated using the following formula:

$$2^{\left\{\frac{\sum_{i=1}^{n} log_2\left(v_{ij}/v_{ik}\right)}{n}\right\}} = 2^{\left\{\frac{\sum_{i=1}^{n} log_2(v_{ij}) - log_2(v_{ik})}{n}\right\}}$$

where, for n participants, v_{ij} and v_{ik} are observed immunogenicity titers or levels for participant i at time points j and k, $j \neq k$.

The 95% CIs for GMT and GMFR will be calculated based on the t distribution of the log-transformed values then back transformed to the original scale for presentation, unless otherwise specified.

6.4.1 Immunogenicity Assessments

For Phase 1/2, there will be two types of humoral immunogenicity assessments:

- Anti-HA antibodies against vaccine-matched strains as measured by HAI or MN assays for primary and secondary analysis.

[redacted]

For Phase 2 NH and Phase 2 Extension, there will be two types of humoral immunogenicity assessments:

- Anti-HA antibodies against vaccine-matched strains as measured by HAI assay for primary and secondary analysis.

[redacted]

6.4.2 Phase 1/2

6.4.2.1 Primary Analysis of Immunogenicity Endpoints

For each vaccination group, the following evaluations will be performed at Day 29.

- GMT of vaccine-matched strain-specific anti-HA antibodies with corresponding 95% CI will be provided at Day 29. The following descriptive statistics will also be provided at Day 29: the number of participants (n), median, min, and max.
- GMFR of vaccine-matched strain-specific anti-HA antibodies with corresponding 95% CI will be provided at Day 29 over pre-injection baseline at Day 1. The following descriptive statistics will also be provided at Day 29: the number of participants (n), median, min, and max.
- Proportion of participants with seroconversion (defined as a Day 29 titer ≥ 1:40 if baseline is < 1:10 or a 4-fold or greater rise if baseline is ≥ 1:10 in anti-HA antibodies measured by HAI assay) will be tabulated with 2-sided 95% Clopper Pearson CIs.

6.4.2.2 Secondary Analysis of Immunogenicity Endpoints

For each vaccination group, the following evaluations will be performed at all evaluable humoral immunogenicity time points (unless otherwise specified).

- GMT of vaccine-matched strain-specific anti-HA antibodies with corresponding 95% CI will be provided at each time point. GM level and corresponding 95% CI will be plotted at each time point. The following descriptive statistics will also be provided at each time point: the number of participants (n), median, min, and max.
- GMFR of vaccine-matched strain-specific anti-HA antibodies with corresponding 95% CI will be provided at each post-baseline time point over pre-injection baseline at Day 1. GMFR and corresponding 95% CI will be plotted at each time point. The following descriptive statistics will also be provided at each time point: the number of participants (n), median, min, and max.
- Proportion of participants with seroconversion at each visit following Day 29. Summaries will be tabulated with 2-sided 95% Clopper Pearson CIs.

6.4.2.3 Exploratory Analysis of Immunogenicity

[redacted]

6.4.3 Phase 2 Northern Hemisphere and Phase 2 Extension

6.4.3.1 Primary Analysis of Immunogenicity Endpoints

Immune responses, as measured by GMT and seroconversion rate in the mRNA-1010 groups based on Day 29 anti-HA antibodies titers as measured by HAI assay, will be compared with that of participants receiving active comparator for all 4 strains.

For Phase 2 NH and Phase 2 Extension, the following evaluations will be performed at Day 29:

- An analysis of covariance (ANCOVA) model will be carried out with anti-HA antibodies titers at Day 29 as a dependent variable and vaccination group as an independent variable. This model will be adjusted for the baseline antibody titer. For Phase 2 NH, age group and vaccination status will be included as covariates. For Phase 2 Extension, age group will be included as a covariate. The GMT of vaccine-matched influenza A and B strains at Day 29 will be estimated by the geometric least square mean (GLSM) from the model (model-based GMT). The ratio of GMT (GMTr) will be estimated by the ratio of GLSM from the model. The corresponding 2 sided 95% CI will be provided to assess the difference in immune response between the mRNA 1010 compared with the active comparator at Day 29. The model will be conducted based on the log transformed values then back transformed to the original scale for presentation.

- The number and proportion of participants with seroconversion (defined in Section 3.2.1) at Day 29 will be provided with 2-sided 95% CI using Clopper Pearson method for each vaccination group. To compare the seroconversion rates between the vaccination groups, the Miettinen-Nurminen's method will be used to calculate the 95% CI for the difference in the seroconversion rates.

The analyses above may also be performed by vaccination status.

6.4.3.2 Secondary Analysis of Immunogenicity Endpoints

- GMFR of vaccine-matched influenza A and B strains (as measured by HAI) at Day 29 with corresponding 95% CI using Clopper Pearson method for each vaccination group will be provided

- The number and proportion of participants with an anti-HA antibodies titer ≥ 1:40 at Day 29 will be provided with 2-sided 95% CI using Clopper Pearson method for each vaccination group. The Miettinen-Nurminen's method will be used to

calculate the 95% CI of the difference in the rates of anti-HA antibodies titer ≥ 1:40 at Day 29 between the vaccination groups.

6.4.3.3 Exploratory Analysis of Immunogenicity Endpoints

[redacted]

6.5 Analysis of Influenza Infection

For Phase 1/2, Phase 2 NH, and Phase 2 Extension, [redacted], and the analyses of influenza infection by RT-PCR test may be performed using the FAS. Additionally, ILIs will be further identified in Phase 2 NH and Phase 2 Extension:

1. A protocol-defined ILI is determined by the occurrence of at least 1 respiratory illness symptom concurrently with at least 1 systemic symptom, or the occurrence of any 2 or more respiratory symptoms defined below:

    - Sore throat
    - Cough/rhinorrhea/nasal congestion (≥ 1 of the 3 symptoms count as 1 respiratory symptom)
    - Sputum production
    - Wheezing

- Difficulty breathing

2. A CDC defined ILI is defined as body temperature ≥ 37.8°C (100°F) accompanied by cough and/or sore throat.

3. An RT-PCR confirmed ILI is defined as a positive influenza result by RT-PCR done at any setting during the study period.

Summaries below will be applied to Phase 1/2, Phase 2 NH, and Phase 2 Extension, unless otherwise specified.

- RT-PCR test results at baseline will be summarized by test and vaccination group. Participants with positive influenza results at baseline will be presented in a listing.
- The number and percentage of participants with at least one positive influenza result at post-baseline will be provided by vaccination group. Participants with at least one positive influenza result at post-baseline will be presented in a listing.
- For Phase 2 NH and Phase 2 Extension, the number of participants with RT-PCR confirmed ILI, protocol-defined ILI, or CDC-defined ILI that begin at least 14 days after vaccination through Day 181/EoS will be summarized by vaccination group. Participants with RT-PCR confirmed ILI, protocol-defined ILI, or CDC-defined ILI will be presented in a listing.

6.6 Interim Analysis

A Day 29 IA is planned in this study for the Phase 1/2, Phase 2 NH, and Phase 2 Extension. The IA for Phase 1/2 will be performed after all participants have completed the Day 29 visit. This interim analysis will be focused on providing insight on the primary and secondary study objectives using the available data. The IA for Phase 2 NH and Phase 2 Extension will be on safety (reactogenicity and immunogenicity as applicable) and will be performed after participants have completed the Day 29 visit. An interim study report may be generated, in which case a formal database lock will be performed to support the interim study report.

All relevant data for the Phase 1/2, Phase 2 NH, and Phase 2 Extension IA will be cleaned (i.e., data that are as clean as possible).

A limited number of Sponsor and CRO personnel will be unblinded to perform the IAs. Please refer to the DBP for more details on the blinding of IA.

An independent, unblinded statistics team will carry out the IA. The unblinded statistics team will not be involved in either study design or the regular study conduct. The study site staffs, investigators, study monitors, and participants will remain blinded until after the final database lock for final analysis.

The final analysis of all applicable endpoints will be performed after all participants have completed all planned study procedures. Results of this analysis will be presented in a final clinical study report (CSR), including individual listings.

6.7 Data Safety Monitoring Board

An independent DSMB, composed of independent members with relevant therapeutic and/or biostatistical expertise, will conduct unblinded reviews of safety data on an ad hoc basis if any pause rule is met or as requested by the safety oversight and/or the internal safety team. The DSMB will also review unblinded IA results provided by the independent unblinded statistician. Refer to the DSMB charter for more details.

7 Changes from Planned Analyses in Protocol

Not applicable.

8 References

Department of Health and Human Services (DHHS), Food and Drug Administration (FDA), Center for Biologics Evaluation and Research (US). Guidance for industry: Toxicity grading scale for healthy adult and adolescent volunteers enrolled in preventative vaccine clinical trials. September 2007

Available from:
https://www.fda.gov/downloads/BiologicsBloodVaccines/GuidanceComplianceRegulatoryInformation/Guidances/Vaccines/ucm091977.pdf.

9 List of Appendices

9.1 Appendix A Standards for Safety and Immunogenicity Variable Display in TFLs

Continuous Variables: The precision for continuous variables will be based on the precision of the data itself. The mean and median will be presented to one decimal place more than the original results; the SD will be presented to two decimal places more than the original results; the minimum and maximum will be presented to the same precision as the original results.

Categorical Variables: Percentages will be presented to one decimal place.

9.2 Appendix B Analysis Visit Windows for Safety and Immunogenicity Analysis

Safety and Immunogenicity Analysis will be summarized using the following analysis visit window for post injection assessments:

Step 1: If the safety and immunogenicity assessments are collected at scheduled visit, i.e., nominal scheduled visit, the data collected at scheduled visit will be used.

Step 2: If the safety and immunogenicity assessments are not collected at the scheduled visit, assessments collected at unscheduled visit will be used using the analysis visit windows described in Table 3 (Phase 1/2) and Table 4 (Phase 2 NH and Phase 2 Extension) below.

If a participant has multiple assessments within the same analysis visit, the following rule will be used:

- If multiple assessments occur within a given analysis visit, the assessment closest to the target study day will be used.
- If there are 2 or more assessments equal distance to the target study day, the last assessment will be used.

Table 3 Phase 1/2 Visit Windows

Visit	Target Study Day	Visit Window in Study Day
**Labs**		
Day 8	8	[6, 9]
**Immunogenicity**		
Day 1	1 (Date of Injection)	1, Pre-vaccination
Day 8	8	[2, 18]

Day 29	29	[19, 105]
Day 181	181	≥ 106

Table 4 Phase 2 NH and Phase 2 Extension Visit Windows

**Visit**	**Target Study Day**	**Visit Window in Study Day**
**Immunogenicity**		
Day 1	1 (Date of Injection)	1, Pre-vaccination
Day 29	29	[2, 60]
Day 91	91	[61, 136]
Day 181	181	≥ 137

9.3 Appendix C Imputation Rules for Missing Prior/Concomitant Medications and Non-Study Vaccinations

Imputation rules for missing or partial medication start/end dates are defined below:

1. Missing or partial medication start date:
   - If only Day is missing, use the first day of the month, unless:
     - The medication end date is after the date of injection or is missing AND the start month and year of the medication coincide with the start month and year of the injection. In this case, use the date of injection.
   - If Day and Month are both missing, use the first day of the year, unless:
     - The medication end date is after the date of injection or is missing AND the start year of the medication coincide with the start year of the injection. In this case, use the date of injection
   - If Day, Month, and Year are all missing, the date will not be imputed, but the medication will be treated as though it began prior to the injection for purposes of determining if status as prior or concomitant.
2. Missing or partial medication end date:
   - If only Day is missing, use the earliest date of (last day of the month, study completion, discontinuation from the study, or death).
   - If Day and Month are both missing, use the earliest date of (last day of the year, study completion, discontinuation from the study, or death).

- If Day, Month, and Year are all missing, the date will not be imputed, but the medication will be flagged as a continuing medication.

In summary, the prior, concomitant, or post categorization of a medication is described in Table 5 below.

Table 5 Prior, Concomitant, and Post Categorization of Medications and Non-Study Vaccinations

	Medication End Date		
**Medication Start Date**	**< Injection Date of IP**	**≥ Injection Date and ≤ Injection Day + 27 Days After Injection**	**> 28 Days After Injection [1]**
< Injection date of IP [2]	P	P, C	P, C, A
≥ Injection date and ≤ 28 days after injection	-	C	C, A
> 28 days after injection	-	-	A

A = Post; C = Concomitant; P = Prior
[1] includes medications with completely missing end date
[2] includes medications with completely missing start date

9.4 Appendix A Imputation Rules for Missing AE Dates

Imputation rules for missing or partial AE start dates and end dates are defined below:

1. Missing or partial AE start date:

- If only Day is missing, use the first day of the month, unless:
  - o The AE end date is after the date of the injection or is missing AND the start month and year of the AE coincide with the start month and year of the injection. In this case, use the date and time of the injection, even if time is collected.
- If Day and Month are both missing, use the first day of the year, unless:
  - o The AE end date is after the date of the injection or is missing AND the start year of the AE coincides with the start year of the injection. In this case, use the date of the injection

- If Day, Month and Year are all missing, the date will not be imputed. However, if the AE end date is prior to the date of the injection, then the AE will be considered a pre-treatment AE. Otherwise, the AE will be considered treatment-emergent.

2. Missing or partial AE end dates will not be imputed.

9.5 Appendix E Schedule of Events

9.5.1 Phase 1/2 - Schedule of Events

Visit Number	SCRN[1]	1	2	3	4	5, 6, and 7	8	Unscheduled
**Type of Visit**	**C**	**C**	**C**	**C**	**C**	**SC**	**C**	**C**
**Month Time Point**	**NA**	**M0**	**M0**	**M1**	**M2**	**M3-M5**	**M6**	**M0-M6**
**Study Visit Day**	**-**	**D1 (Baseline)**	**D8**	**D29**	**D57**	**D91, D121, D151**	**D181/EoS**	**NA**
**Window Allowance (Days)**	**-28**	**-**	**–2 or +1**	**±2**	**±5**	**±5**	**±14**	**NA**
ICF, demographics, concomitant medications, medical history	X	-	-	-	-	-	-	-
Inclusion/exclusion criteria	X	X	-	-	-	-	-	-
Blood collection for safety laboratory samples[2]	X	-	X	-	-	-	-	-
Physical examination[3]	X	-	-	-	-	-	-	-
Vital signs[4]	X	X	-	-	-	-	-	-
Pregnancy testing[5]	X	X	-	-	-	-	-	-
Randomization	-	X	-	-	-	-	-	-
Study vaccination (including 60-minute postdosing observation period)	-	X[6]	-	-	-	-	-	-
Blood collection for humoral immunogenicity[7]	-	X	X	X	-	-	X	-
[redacted]								
NP swab for virus detection[8]	-	X	-	-	-	-	-	X
eDiary activation for recording solicited ARs (7 days)[9]	-	X	-	-	-	-	-	-
Review of eDiary	-	-	X	-	-	-	-	-
Follow-up safety calls[10]	-	-	-	-	-	X	-	-
Recording of unsolicited AEs	-	X	X	X	-	-	-	-

Visit Number	SCRN[1]	1	2	3	4	5, 6, and 7	8	Unscheduled
**Type of Visit**	**C**	**C**	**C**	**C**	**C**	**SC**	**C**	**C**
**Month Time Point**	**NA**	**M0**	**M0**	**M1**	**M2**	**M3-M5**	**M6**	**M0-M6**
**Study Visit Day**	**-**	**D1 (Baseline)**	**D8**	**D29**	**D57**	**D91, D121, D151**	**D181/EoS**	**NA**
**Window Allowance (Days)**	**-28**	**-**	**–2 or +1**	**±2**	**±5**	**±5**	**±14**	**NA**
Recording of MAAEs and concomitant medications relevant to or for the treatment of the MAAE[11]	-	X	X	X	X	X	X	X
Recording of SAEs, AESIs, and concomitant medications relevant to or for the treatment of the SAE and/or AESI[11]	-	X	X	X	X	X	X	X
Recording of concomitant medications and nonstudy vaccinations[11]	X	X	X	X	X	X	X	X
Phase 1/2 completion	-	-	-	-	-	-	X	-

Abbreviations: AE = adverse event; AESI = adverse event of special interest; ALT = alanine aminotransferase; AR = adverse reaction; AST = aspartate aminotransferase; C = clinic visit (ie, study site visit); COVID-19 = coronavirus disease 2019; D = day; eCRF = electronic case report form; eDiary = electronic diary; EoS = end of study; FSH = follicle-stimulating hormone; ICF = informed consent form; ILI = influenza-like illness; IM = intramuscular; IP = investigational product; M = month; MAAE = medically attended adverse event; NA = not applicable; NP = nasopharyngeal; RT-PCR = reverse transcription polymerase chain reaction; SAE = serious adverse event; SARS-CoV-2 = severe acute respiratory syndrome coronavirus 2; SC = safety call (phone or contact by electronic means); SCRN = screening; WBC = white blood cell.

[1] The Screening Visit and Day 1 Visit cannot be completed on the same day. Additionally, the Screening Visit may be performed over multiple visits if within the 28-day screening window.

[2] Safety laboratory tests: WBC count, hemoglobin, platelets, ALT, AST, total bilirubin, alkaline phosphatase, and creatinine.

[3] Physical examination: A full physical examination, including height and weight, will be performed at screening. Symptom-directed physical examinations may be performed at other time points at the discretion of the Investigator. Any clinically significant finding identified during a study visit should be reported as an MAAE.

[4] Vital sign measurement: systolic and diastolic blood pressure, heart rate, respiratory rate, and body temperature. Participants will be seated for at least 5 minutes before all measurements are taken. Vital signs will be collected at the Screening Visit and on the day of vaccination, once before and at least 1 hour after the vaccination. Vital signs will be collected at other study site visits only in conjunction with a symptom-directed physical examination. The preferred route of temperature assessment is oral. Participants must not eat or drink anything hot or cold within 10 minutes before oral temperature is taken. If any of the vital sign measurements meet the toxicity grading criteria for clinical abnormalities of Grade 3 or greater, the abnormal value and grade will be documented in the AE section of the eCRF (unless there is another known cause of the abnormality that would result in an AE classification). The Investigator will continue to monitor the participant with additional assessments until the vital sign value has reached the reference range, returns to the vital sign value at baseline, is considered stable or until the Investigator determines that follow-up is no longer medically necessary.

5 A point-of-care urine pregnancy test will be performed at the Screening Visit and before the vaccine dose on Day 1. At the discretion of the Investigator, a pregnancy test either via blood or point-of-care urine can be performed at any time. The FSH level may be measured at the Screening Visit as necessary and at the discretion of the Investigator to confirm postmenopausal status.

6 A 50-µg dose of mRNA-1010 will be administered for Vaccination Group 1, a 100-µg dose of mRNA-1010 will be administered for Vaccination Group 2, a 200-µg dose of mRNA-1010 will be administered for Vaccination Group 3, and placebo will be administered for Vaccination Group 4. All participants will receive a single 0.5-mL IM injection of the IP.

7 Samples for humoral and [redacted] immunogenicity (and safety laboratory samples) must be collected prior to receipt of vaccination on Day 1. [redacted]

8 An NP swab specimen will be collected on Day 1 prior to vaccination to assess for asymptomatic infection with respiratory pathogens, including influenza virus and SARS-CoV-2. An NP swab will also be collected at any time during the study through study completion if any signs or symptoms or an MAAE suggesting COVID-19 or ILI occur, to confirm the diagnosis by RT-PCR. For signs or symptoms during the study, a participant will be instructed to contact the study site to have an NP swab collected for testing for respiratory pathogens at an unscheduled visit. The NP swabs may be collected as part of a home visit in lieu of a study site visit.

9 The eDiary entries will be recorded by the participant at approximately 1 hour after vaccination while at the study site under the supervision of the study site staff. Participants will continue to record data in the eDiary for solicited AEs each day after they leave the study site, preferably in the evening and at the same time each day, on the day of injection, and for 6 days following injection. Any solicited AR that continues beyond Day 7 will be reported until it is no longer reported but only until 28 days after vaccination. Adverse reactions recorded in diaries beyond Day 7 should be reviewed either during the next scheduled telephone call or at the next study site visit.

10 Trained study personnel will call all participants to collect information relating to any AEs, MAAEs, AEs leading to withdrawal from study participation, SAEs, AESIs, information on concomitant medications associated with those events, and any nonstudy vaccinations.

11 All concomitant medications and nonstudy vaccinations will be recorded through 28 days after vaccination; all concomitant medications relevant to or for the treatment of an SAE, AESI, or MAAE will be recorded from Day 1 through Day 181/EoS.

9.5.2 Phase 2 NH - Schedule of Events

Visit Number	SCRN[1]	1	2	3	4	5	6	Unscheduled
Type of Visit/Contact	C	C	SC	SC	C	C	C	C
Month Time Point	NA	M0	M0	M0	M1	M3	M6	M0-M6
Study Visit Day	-	D1 (Baseline)	D8	D15	D29	D91	D181/EoS	NA
Window Allowance (Days)	-28	-	±3	±3	-7 to +3	±5	±14	NA
ICF, demographics, concomitant medications, medical history[2]	X	-	-	-	-	-	-	-
Inclusion/exclusion criteria	X	X	-	-	-	-	-	-
Physical examination[3]	X	-	-	-	-	-	-	-
Vital signs[4]	X	X	-	-	-	-	-	-
Pregnancy testing[5]	X	X	-	-	-	-	-	-
Randomization	-	X	-	-	-	-	-	-
Study vaccination (including 60-minute postdosing observation period)	-	X[6]	-	-	-	-	-	-
Blood collection for humoral immunogenicity (and exploratory assessments)[7]	-	X	-	-	X	X	X	-
Optional blood collection for pharmacogenomics	-	X	-	-	-	-	-	-
NP swab for virus detection[8]	-	-	-	-	-	-	-	X
eDiary activation for recording solicited ARs (7 days)[9]	-	X	-	-	-	-	-	-
Review of eDiary for solicited ARs	-	-	X	-	-	-	-	-
eDiary activation for active and passive surveillance[10]	-	X	-	-	-	-	-	-
eDiary active surveillance collection of symptoms of ILI[11]	-	3 to 4 times weekly				Twice weekly		-

**Visit Number**	**SCRN[1]**	**1**	**2**	**3**	**4**	**5**	**6**	**Unscheduled**
**Type of Visit/Contact**	**C**	**C**	**SC**	**SC**	**C**	**C**	**C**	**C**
**Month Time Point**	**NA**	**M0**	**M0**	**M0**	**M1**	**M3**	**M6**	**M0-M6**
**Study Visit Day**	**-**	**D1 (Baseline)**	**D8**	**D15**	**D29**	**D91**	**D181/EoS**	**NA**
**Window Allowance (Days)**	**-28**	**-**	**±3**	**±3**	**-7 to +3**	**±5**	**±14**	**NA**
eDiary passive surveillance collection of symptoms of ILI[11]	-	X						-
Review of eDiary[12]	-	Review participant-recorded ILI starting on Day 1 through Day 181/EoS						
Follow-up safety call	-	-	X	X	-	-	-	-
Recording of unsolicited AEs	-	X	X	X	X	-	-	-
Recording of MAAEs and concomitant medications relevant to or for the treatment of the MAAE[13]	-	X	X	X	X	X	X	X
Recording of SAEs, AESIs, and concomitant medications relevant to or for the treatment of the SAE and/or AESI[13]	-	X	X	X	X	X	X	X
Recording of concomitant medications and nonstudy vaccinations[14]	X	X	X	X	X	X	X	X
Study completion	-	-	-	-	-	-	X[15]	-

Abbreviations: AE = adverse event; AESI = adverse event of special interest; AR = adverse reaction; C = clinic visit (ie, study site visit); COVID-19 = coronavirus disease 2019; D = day; eCRF = electronic case report; form; eDiary = electronic diary; EoS = end of study; FSH = follicle-stimulating hormone; ICF = informed consent form; ILI = influenza-like illness; IM = intramuscular; M = month; MAAE = medically attended adverse event; NA = not applicable; NP = nasopharyngeal swab; SAE = serious adverse event; SARS-CoV-2 = severe acute respiratory syndrome coronavirus 2; SC = safety call (phone or contact by electronic means); SCRN = screening.

[1] The Screening Visit and Day 1 Visit may be performed on the same day or a different day. Additionally, the Screening Visit may be performed over multiple visits if within the 28-day screening window.

[2] Verbal confirmation of medical history is acceptable.

[3] Physical examination: A full physical examination, including height and weight, will be performed at screening; symptom-directed physical examinations will be performed at all other clinic visits (ie, study site visits). Interim physical examinations will be performed at the discretion of the Investigator. Any clinically significant finding identified by a healthcare professional during study visits should be reported as an MAAE.

[4] Vital sign measurement: Systolic and diastolic blood pressure, heart rate, respiratory rate, and body temperature. The preferred route of temperature assessment is oral. Participants must not eat or drink anything hot or cold within 10 minutes before oral temperature is taken. Participants will be seated for at least 5 minutes before all measurements are taken. Vital signs will be collected on the day of vaccination, once before vaccination and approximately 1 hour after vaccination. Vital signs may be collected at other study site visits in conjunction with a symptom-directed physical examination. If any of the vital sign measurements meet the toxicity grading criteria for clinical abnormalities of Grade 3 or greater, the abnormal value and grade will be documented in the AE section of the eCRF (unless there is another known cause of the abnormality that would result in an AE classification). The Investigator will continue to monitor the participant with additional assessments until the vital sign value has reached the reference range, returns to the vital sign value at baseline, is considered stable or until the Investigator determines that follow-up is no longer medically necessary.

[5] A point-of-care urine pregnancy test will be performed at the Screening Visit (and before the vaccine dose on Day 1 if Day 1 is not on the same day as the Screening Visit). At the discretion of the Investigator, a pregnancy test either via blood or point-of-care urine can be performed at any time. The FSH level may be measured at the Screening Visit as necessary and at the discretion of the Investigator, to confirm postmenopausal status.

[6] See Table 1 for dose levels and vaccination groups. All participants will be randomized to receive a single 0.5-mL IM injection.

[7] Samples for humoral immunogenicity (and exploratory assessments) must be collected prior to receipt of vaccination on Day 1. A subset of samples collected will be assessed for humoral immunogenicity (and exploratory assessments).

[8] An NP swab specimen for pathogens, including influenza virus and SARS-CoV-2 will be collected any time from Day 1 through Day 181/EoS if participants have protocol-defined ILI or symptoms suggestive of COVID-19 or other upper or lower respiratory infection at the Investigator's discretion. If participants experience these symptoms/signs, they will be instructed to contact the study site to have an NP swab collected for testing. The NP swabs may be collected as part of a home visit in lieu of a study site visit.

[9] The eDiary entries will be recorded by all participants at approximately 1 hour after vaccination while at the study site under the supervision of the study site staff. Participants will continue to record data in the eDiary for solicited ARs each day after they leave the study site, preferably in the evening and at the same time each day, on the day of vaccination, and the 6 days following vaccination. Any solicited AR that is ongoing beyond Day 7 will be reported until it is no longer reported but only until 28 days after vaccination. Adverse reactions recorded in the eDiary beyond Day 7 should be reviewed either during the next scheduled call or study site visit, or during an unscheduled visit.

[10] The eDiary will be activated for collection of ILI symptoms.

[11] Passive Surveillance: Participants will be instructed to report symptoms of ILI any time from Day 1 through Day 181/EoS; Active Surveillance: Participants will be instructed to report whether ILI symptoms have been experienced, 3 to 4 times weekly from Day 1 through Day 29 and twice weekly from Day 30 through Day 181/EoS, via eDiary or telephone calls. If symptoms occur, participants will be directed to return to the study site as soon as possible, but no later than 72 hours after the onset of symptoms for medical evaluation and an NP swab.

[12] Review of eDiary for recording of symptoms of ILI.

[13] Trained study personnel will call all participants to collect information relating to any MAAEs, AEs leading to study discontinuation, SAEs, AESIs, information on concomitant medications associated with those events, and any nonstudy vaccinations.

[14] All concomitant medications and nonstudy vaccinations will be recorded through 28 days after vaccination; all concomitant medications relevant to or for the treatment of an SAE, AESI, or MAAE will be recorded from Day 1 through Day 181/EoS.

[13] Participants who develop ILI will be followed through 30 days from the onset of ILI even if Day 30 is beyond Day 181/EoS.

9.5.3 Phase 2 Extension - Schedule of Events

**Visit Number**	**SCRN[1]**	**1**	**2**	**3**	**4**	**5**	**6**	**7**	**Unscheduled**
**Type of Visit/Contact**	**C**	**C**	**C**	**SC**	**SC**	**C**	**C**	**C**	**C**
**Month Time Point**	**NA**	**M0**	**M0**	**M0**	**M0**	**M1**	**M3**	**M6**	**M0-M6**
**Study Visit Day**	**-**	**D1 (Baseline)**	**D4**	**D8**	**D15**	**D29**	**D91**	**D181/EoS**	**NA**
**Window Allowance (Days)**	**-28**	**-**	**-2**	**±3**	**±3**	**-7 to +3**	**±5**	**±14**	**NA**
ICF, demographics, concomitant medications, medical history[2]	X	-	-	-	-	-	-	-	-
Inclusion/exclusion criteria	X	X	-	-	-	-	-	-	-
Physical examination[3]	X	-	-	-	-	-	-	-	-
Vital signs[4]	X	X	-	-	-	-	-	-	-
Pregnancy testing[5]	X	X	-	-	-	-	-	-	-
Randomization	-	X	-	-	-	-	-	-	-
Study vaccination (including 60-minute postdosing observation period)	-	X[6]	-	-	-	-	-	-	-
Blood collection for humoral immunogenicity (and exploratory assessments)[7]	-	X	-	-	-	X	X	X	-
Optional blood collection for pharmacogenomics	-	X	-	-	-	-	-	-	-
Optional blood collection for potential biomarker analysis[8]			X						
NP swab for virus detection[9]	-	-	-	-	-	-	-	-	X
eDiary activation for recording solicited ARs (7 days)[10]	-	X	-	-	-	-	-	-	-
Review of eDiary for solicited ARs	-	-	-	X	-	-	-	-	-
Follow-up safety call	-	-	-	X	X	-	-	-	-
Recording of unsolicited AEs	-	X	-	X	X	X	-	-	-

Visit Number	SCRN[1]	1	2	3	4	5	6	7	Unscheduled
**Type of Visit/Contact**	**C**	**C**	**C**	**SC**	**SC**	**C**	**C**	**C**	**C**
**Month Time Point**	**NA**	**M0**	**M0**	**M0**	**M0**	**M1**	**M3**	**M6**	**M0-M6**
**Study Visit Day**	**-**	**D1 (Baseline)**	**D4**	**D8**	**D15**	**D29**	**D91**	**D181/EoS**	**NA**
**Window Allowance (Days)**	**-28**	**-**	**-2**	**±3**	**±3**	**-7 to +3**	**±5**	**±14**	**NA**
Recording of MAAEs and concomitant medications relevant to or for the treatment of the MAAE[11]	-	X	-	X	X	X	X	X	X
Recording of SAEs, AESIs, and concomitant medications relevant to or for the treatment of the SAE and/or AESI[11]	-	X	-	X	X	X	X	X	X
Recording of concomitant medications and nonstudy vaccinations[12]	X	X	-	X	X	X	X	X	X
Study completion	-	-	-	-	-	-	-	X[13]	-

Abbreviations: AE = adverse event; AESI = adverse event of special interest; AR = adverse reaction; C = clinic visit (ie, study site visit); COVID-19 = coronavirus disease 2019; D = day; eCRF = electronic case report; form; eDiary = electronic diary; EoS = end of study; FSH = follicle-stimulating hormone; ICF = informed consent form; ILI = influenza-like illness; IM = intramuscular; M = month; MAAE = medically attended adverse event; NA = not applicable; NP = nasopharyngeal swab; SAE = serious adverse event; SARS-CoV-2 = severe acute respiratory syndrome coronavirus 2; SC = safety call (phone or contact by electronic means); SCRN = screening.

[1] The Screening Visit and Day 1 Visit may be performed on the same day or a different day. Additionally, the Screening Visit may be performed over multiple visits if within the 28-day screening window.

[2] Verbal confirmation of medical history is acceptable.

[3] Physical examination: A full physical examination, including height and weight, will be performed at screening; symptom-directed physical examinations will be performed at all other clinic visits (ie, study site visits). Interim physical examinations will be performed at the discretion of the Investigator. Any clinically significant finding identified by a healthcare professional during study visits should be reported as an MAAE.

[4] Vital sign measurement: Systolic and diastolic blood pressure, heart rate, respiratory rate, and body temperature. The preferred route of temperature assessment is oral. Participants must not eat or drink anything hot or cold within 10 minutes before oral temperature is taken. Participants will be seated for at least 5 minutes before all measurements are taken. Vital signs will be collected on the day of vaccination, once before vaccination and approximately 1 hour after vaccination. Vital signs may be collected at other study site visits in conjunction with a symptom-directed physical examination. If any of the vital sign measurements meet the toxicity grading criteria for clinical abnormalities of Grade 3 or greater, the abnormal value and grade will be documented in the AE section of the eCRF (unless there is another known cause of the abnormality that would result in an AE classification). The Investigator will continue to monitor the participant with additional assessments until the vital sign value has reached the reference range, returns to the vital sign value at baseline, is considered stable or until the Investigator determines that follow-up is no longer medically necessary.

[5] A point-of-care urine pregnancy test will be performed at the Screening Visit (and before the vaccine dose on Day 1 if Day 1 is not on the same day as the Screening Visit). At the discretion of the Investigator, a pregnancy test either via blood or point-of-care urine can be performed at any time. The FSH level may be measured at the Screening Visit as necessary and at the discretion of the Investigator, to confirm postmenopausal status.
[6] See Table 1 for dose levels and vaccination groups. All participants will be randomized to receive a single 0.5-mL IM injection.
[7] Samples for humoral immunogenicity (and exploratory assessments) must be collected prior to receipt of vaccination on Day 1. A subset of samples collected will be assessed for humoral immunogenicity (and exploratory assessments).
[8] Biomarker plasma and biomarker serum samples will be stored for potential future biomarker assessment
[9] An NP swab specimen for pathogens, including influenza virus and SARS-CoV-2 will be collected any time from Day 1 through Day 181/EoS if participants have protocol-defined ILI or symptoms suggestive of COVID-19 or other upper or lower respiratory infection at the Investigator's discretion. If participants experience these symptoms/signs, they will be instructed to contact the study site to have an NP swab collected for testing. The NP swabs may be collected as part of a home visit in lieu of a study site visit.
[10] The eDiary entries will be recorded by all participants at approximately 1 hour after vaccination while at the study site under the supervision of the study site staff. Participants will continue to record data in the eDiary for solicited ARs each day after they leave the study site, preferably in the evening and at the same time each day, on the day of vaccination, and the 6 days following vaccination. Any solicited AR that is ongoing beyond Day 7 will be reported until it is no longer reported but only until 28 days after vaccination. Adverse reactions recorded in the eDiary beyond Day 7 should be reviewed either during the next scheduled call or study site visit, or during an unscheduled visit.
[11] The eDiary will be activated for collection of ILI symptoms.
[12] Passive Surveillance: Participants will be instructed to report symptoms of ILI any time from Day 1 through Day 181/EoS; Active Surveillance: Participants will be instructed to report whether ILI symptoms have been experienced, 3 to 4 times weekly from Day 1 through Day 29 and twice weekly from Day 30 through Day 181/EoS, via eDiary or telephone calls. If symptoms occur, participants will be directed to return to the study site as soon as possible, but no later than 72 hours after the onset of symptoms for medical evaluation and an NP swab.
[13] Review of eDiary for recording of symptoms of ILI.
[11] Trained study personnel will call all participants to collect information relating to any MAAEs, AEs leading to study discontinuation, SAEs, AESIs, information on concomitant medications associated with those events, and any nonstudy vaccinations.
[12] All nonstudy vaccinations will be recorded through Day 181/EoS. All concomitant medications will be recorded through 28 days after vaccination; all concomitant medications relevant to or for the treatment of an SAE, AESI, or MAAE will be recorded from Day 1 through Day 181/EoS.
[13] Participants who develop ILI will be followed through 30 days from the onset of ILI even if Day 30 is beyond Day 181/EoS.

9.6 Appendix F Severity Grading of Laboratory Abnormalities

**Serum***	**Mild (Grade 1)**	**Moderate (Grade 2)**	**Severe (Grade 3)**	**Potentially Life Threatening (Grade 4)****
Sodium – Hyponatremia mEq/L	132 – 134	130 – 131	125 – 129	< 125
Sodium – Hypernatremia mEq/L	144 – 145	146 – 147	148 – 150	> 150
Potassium – Hyperkalemia mEq/L	5.1 – 5.2	5.3 – 5.4	5.5 – 5.6	> 5.6
Potassium – Hypokalemia mEq/L	3.5 – 3.6	3.3 – 3.4	3.1 – 3.2	< 3.1
Glucose – Hypoglycemia mg/dL	65 – 69	55 – 64	45 – 54	< 45
Glucose – Hyperglycemia<br>Fasting – mg/dL<br>Random – mg/dL	100 – 110<br>110 – 125	111 – 125<br>126 – 200	>125<br>>200	Insulin requirements or hyperosmolar coma
Blood Urea Nitrogen (BUN) mg/dL	23 – 26	27 – 31	> 31	Requires dialysis
Creatinine – mg/dL	1.5 – 1.7	1.8 – 2.0	2.1 – 2.5	> 2.5 or requires dialysis
Calcium – hypocalcemia mg/dL	8.0 – 8.4	7.5 – 7.9	7.0 – 7.4	< 7.0
Calcium – hypercalcemia mg/dL	10.5 – 11.0	11.1 – 11.5	11.6 – 12.0	> 12.0
Magnesium – hypomagnesemia mg/dL	1.3 – 1.5	1.1 – 1.2	0.9 – 1.0	< 0.9
Phosphorous – hypophosphatemia mg/dL	2.3 – 2.5	2.0 – 2.2	1.6 – 1.9	< 1.6
CPK – mg/dL	1.25 – 1.5 x ULN***	1.6 – 3.0 x ULN	3.1 – 10 x ULN	> 10 x ULN
Albumin – Hypoalbuminemia g/dL	2.8 – 3.1	2.5 – 2.7	< 2.5	--
Total Protein – Hypoproteinemia g/dL	5.5 – 6.0	5.0 – 5.4	< 5.0	--
Alkaline phosphate – increase by factor	1.1 – 2.0 x ULN	2.1 – 3.0 x ULN	3.1 – 10 x ULN	> 10 x ULN
Liver Function Tests – ALT, AST increase by factor	1.1 – 2.5 x ULN	2.6 – 5.0 x ULN	5.1 – 10 x ULN	> 10 x ULN
Bilirubin – when accompanied by any increase in Liver Function Test increase by factor	1.1 – 1.25 x ULN	1.26 – 1.5 x ULN	1.51 – 1.75 x ULN	> 1.75 x ULN
Bilirubin – when Liver Function Test is normal; increase by factor	1.1 – 1.5 x ULN	1.6 – 2.0 x ULN	2.0 – 3.0 x ULN	> 3.0 x ULN
Cholesterol	201 – 210	211 – 225	> 226	---

Pancreatic enzymes – amylase, lipase	1.1 – 1.5 x ULN	1.6 – 2.0 x ULN	2.1 – 5.0 x ULN	> 5.0 x ULN

* The laboratory values provided in the tables serve as guidelines and are dependent upon institutional normal parameters. Institutional normal reference ranges should be provided to demonstrate that they are appropriate.
** The clinical signs or symptoms associated with laboratory abnormalities might result in characterization of the laboratory abnormalities as Potentially Life Threatening (Grade 4). For example. a low sodium value that falls within a Grade 3 parameter (125-129 mE/L) should be recorded as a Grade 4 hyponatremia event if the participant had a new seizure associated with the low sodium value.
***ULN" is the upper limit of the normal range.

**Hematology ***	**Mild (Grade 1)**	**Moderate (Grade 2)**	**Severe (Grade 3)**	**Potentially Life Threatening (Grade 4)**
Hemoglobin (Female) - mg/dL	11.0 – 12.0	9.5 – 10.9	8.0 – 9.4	< 8.0
Hemoglobin (Female) change from baseline value - mg/dL	Any decrease – 1.5	1.6 – 2.0	2.1 – 5.0	> 5.0
Hemoglobin (Male) - mg/dL	12.5 – 13.5	10.5 – 12.4	8.5 – 10.4	< 8.5
Hemoglobin (Male) change from baseline value – mg/dL	Any decrease – 1.5	1.6 – 2.0	2.1 – 5.0	> 5.0
WBC Increase - cell/mm^3	10,800 – 15,000	15,001 – 20,000	20,001 – 25,000	> 25,000
WBC Decrease - cell/mm^3	2,500 – 3,500	1,500 – 2,499	1,000 – 1,499	< 1,000
Lymphocytes Decrease - cell/mm^3	750 – 1,000	500 – 749	250 – 499	< 250
Neutrophils Decrease - cell/mm^3	1,500 – 2,000	1,000 – 1,499	500 – 999	< 500
Eosinophils - cell/mm^3	650 – 1500	1501 - 5000	> 5000	Hypereosinophilic
Platelets Decreased - cell/mm^3	125,000 – 140,000	100,000 – 124,000	25,000 – 99,000	< 25,000
PT – increase by factor (prothrombin time)	1.0 – 1.10 x ULN**	1.11 – 1.20 x ULN	1.21 – 1.25 x ULN	> 1.25 ULN
PTT – increase by factor (partial thromboplastin time)	1.0 – 1.2 x ULN	1.21 – 1.4 x ULN	1.41 – 1.5 x ULN	> 1.5 x ULN
Fibrinogen increase - mg/dL	400 – 500	501 – 600	> 600	--
Fibrinogen decrease - mg/dL	150 – 200	125 – 149	100 – 124	< 100 or associated with gross bleeding or disseminated intravascular coagulation (DIC)

* The laboratory values provided in the tables serve as guidelines and are dependent upon institutional normal parameters. Institutional normal reference ranges should be provided to demonstrate that they are appropriate.
** "ULN" is the upper limit of the normal range.

9.7 Appendix G Severity Grading of Vital Sign Abnormalities

**Vital Signs***	**Mild (Grade 1)**	**Moderate (Grade 2)**	**Severe (Grade 3)**	**Potentially Life Threatening (Grade 4)**
Fever (°C)**<br>(°F)**	38.0 – 38.4<br>100.4 – 101.1	38.5 – 38.9<br>101.2 – 102.0	39.0 – 40<br>102.1 – 104	> 40<br>> 104
Tachycardia - beats per minute	101 – 115	116 – 130	> 130	ER visit or hospitalization for arrhythmia
Bradycardia - beats per minute***	50 – 54	45 – 49	< 45	ER visit or hospitalization for arrhythmia
Hypertension (systolic) - mm Hg	141 – 150	151 – 155	> 155	ER visit or hospitalization for malignant hypertension
Hypertension (diastolic) - mm Hg	91 – 95	96 – 100	> 100	ER visit or hospitalization for malignant hypertension
Hypotension (systolic) – mm Hg	85 – 89	80 – 84	< 80	ER visit or hospitalization for hypotensive shock
Respiratory Rate – breaths per minute	17 – 20	21 – 25	> 25	Intubation

* Participant should be at rest for all vital sign measurements.
** Oral temperature; no recent hot or cold beverages or smoking.
*** When resting heart rate is between 60 – 100 beats per minute. Use clinical judgement when characterizing bradycardia among some healthy participant populations, for example, conditioned athletes.
