# Supplementary material for: Safety and immunogenicity of a phase 1/2 randomized clinical trial of a quadrivalent, mRNA-based seasonal influenza vaccine (mRNA-1010) in healthy adults: interim analysis
Source: Nat Commun. 2023 Jun 19;14:3631. doi: 10.1038/s41467-023-39376-7 (PMC10279702; doi:10.1038/s41467-023-39376-7)
Supplement: Supplementary file 3 — Reporting Summary [file 41467_2023_39376_MOESM3_ESM.pdf]

nature portfolio

Corresponding author(s): Raffael Nachbagauer and Robert Paris

Last updated by author(s): Apr 24, 2023

Reporting Summary

Nature Portfolio wishes to improve the reproducibility of the work that we publish. This form provides structure for consistency and transparency in reporting. For further information on Nature Portfolio policies, see our Editorial Policies and the Editorial Policy Checklist.

Statistics

For all statistical analyses, confirm that the following items are present in the figure legend, table legend, main text, or Methods section.

n/a	Confirmed	
☐	☒	The exact sample size (*n*) for each experimental group/condition, given as a discrete number and unit of measurement
☐	☒	A statement on whether measurements were taken from distinct samples or whether the same sample was measured repeatedly
☒	☐	The statistical test(s) used AND whether they are one- or two-sided<br>*Only common tests should be described solely by name; describe more complex techniques in the Methods section.*
☒	☐	A description of all covariates tested
☒	☐	A description of any assumptions or corrections, such as tests of normality and adjustment for multiple comparisons
☐	☒	A full description of the statistical parameters including central tendency (e.g. means) or other basic estimates (e.g. regression coefficient) AND variation (e.g. standard deviation) or associated estimates of uncertainty (e.g. confidence intervals)
☒	☐	For null hypothesis testing, the test statistic (e.g. *F*, *t*, *r*) with confidence intervals, effect sizes, degrees of freedom and *P* value noted<br>*Give P values as exact values whenever suitable.*
☒	☐	For Bayesian analysis, information on the choice of priors and Markov chain Monte Carlo settings
☒	☐	For hierarchical and complex designs, identification of the appropriate level for tests and full reporting of outcomes
☒	☐	Estimates of effect sizes (e.g. Cohen's *d*, Pearson's *r*), indicating how they were calculated

Our web collection on statistics for biologists contains articles on many of the points above.

Software and code

Policy information about availability of computer code

Data collection	Statistical analyses were performed using SAS version 9.4.
Data analysis	Statistical analyses were performed using SAS version 9.4.

For manuscripts utilizing custom algorithms or software that are central to the research but not yet described in published literature, software must be made available to editors and reviewers. We strongly encourage code deposition in a community repository (e.g. GitHub). See the Nature Portfolio guidelines for submitting code & software for further information.

Data

Policy information about availability of data

All manuscripts must include a data availability statement. This statement should provide the following information, where applicable:

- Accession codes, unique identifiers, or web links for publicly available datasets
- A description of any restrictions on data availability
- For clinical datasets or third party data, please ensure that the statement adheres to our policy

The ‘Data Availability’ section has been updated to now state “Access to patient-level data presented in this article (antibody assays, safety, and reactogenicity) and supporting clinical documents with external researchers who provide methodologically sound scientific proposals will be available upon reasonable request and subject to review from 2 years after study completion. Such requests can be made to Moderna Inc., 200 Technology Square, Cambridge, MA 02139. A materials transfer and/or data access agreement with the sponsor will be required for accessing shared data. All other relevant data are presented in the paper. The protocol is available as online supplementary material to this article. ClinicalTrials.gov: NCT04956575.”

Human research participants

Policy information about studies involving human research participants and Sex and Gender in Research.

Reporting on sex and gender	The research findings presented within this manuscript do not apply to only one sex or gender. As the study design of this phase 1/2 trial did not allow for sex and gender-based analysis and this analysis was not prespecified, disaggregated findings for sex and gender were not included. Participant gender was determined by self-reporting.
Population characteristics	In Part 1 of the study, a total of 180 participants aged ≥18 years were randomly assigned to receive placebo or 1 dose of mRNA-1010 (50 µg, 100 µg, or 200 µg). The majority of participants were White and non-Hispanic or Latino; in the mRNA-1010 groups, the median age was 51.0 years and 57.0% were female; in the placebo group, the median age was 50.0 years and 51.1% were female. In Part 2 of the trial, a total of 501 participants aged ≥18 years were randomly assigned to receive a single dose of Afluria (n=53) or mRNA-1010 (25 µg, n=152; 50 µg, n=149; 100 µg, n=147). Most participants in Part 2 were White and non-Hispanic or Latino. In the mRNA-1010 groups, the median age was 52.0 years and 57.3% were female; in the Afluria group, the median age was 53.0 years and 54.7% were female.
Recruitment	This is a first-in-human, phase 1/2, randomized, observer-blinded study at 20 sites in the United States to evaluate the safety, reactogenicity, and immunogenicity of mRNA-1010 in adults ≥18 years of age (NCT04956575).<br><br>The initial stage of Part 1 planned for approximately 36 participants (9 participants in each group) to be randomly assigned (1:1:1:1) to receive a single dose of mRNA-1010 (50 µg, 100 µg, or 200 µg) or placebo. Safety data up to 7 days after vaccination were reviewed by a blinded internal safety team; after confirmation that no study pause rules were met, the remaining participants (approximately 36 in each group for a total of 144 participants) were randomly assigned in the Part 1 expansion stage. Randomization at this stage was stratified by age (18-49 years and ≥50 years) and was balanced within each group.<br><br>Approximately 500 participants were planned to be randomly assigned (3:3:3:1) to receive a single dose of mRNA-1010 (25 µg, 50 µg, or 100 µg) or a licensed quadrivalent seasonal influenza vaccine (Afluria®; Seqirus Pty Ltd, Parkville, Victoria, Australia). Randomization was performed in parallel among the 4 vaccine groups, and participants were stratified by age (18-49 years, 50-64 years, or ≥65 years) and vaccination status in the previous influenza season (received or not received).
Ethics oversight	The study was conducted in accordance with the protocol, applicable laws, and regulatory requirements, as well as International Council for Harmonization Good Clinical Practice guidelines, and the consensus ethical principles derived from international guidelines, including the Declaration of Helsinki and Council for International Organizations of Medical Sciences International Ethical Guidelines. The protocol was approved by the central institutional review board (Advarra, Inc.; Columbia, MD) prior to study initiation, and written informed consent was obtained from all participants before enrollment.

Note that full information on the approval of the study protocol must also be provided in the manuscript.

Field-specific reporting

Please select the one below that is the best fit for your research. If you are not sure, read the appropriate sections before making your selection.

☒ Life sciences ☐ Behavioural & social sciences ☐ Ecological, evolutionary & environmental sciences

For a reference copy of the document with all sections, see nature.com/documents/nr-reporting-summary-flat.pdf

Life sciences study design

All studies must disclose on these points even when the disclosure is negative.

Sample size	The sample sizes for Part 1 and Part 2 were not based on formal statistical hypothesis testing. In Part 1, it was expected that with 45 participants in each vaccine group, there was an approximately 90% probability to observe at least 1 participant with an adverse event (AE; if the true incidence rate of AEs was 5%) or an approximately 99% probability if the true incidence rate was 10%. For Part 2, a sample size of 150 participants in each vaccine group had at least a 95% probability to observe at least 1 participant with an AE if the true incidence rate was 2%; if the true incidence rate was 3%, then the probability would be 99%.
Data exclusions	No data were excluded from this analysis.
Replication	In Part 1 of the study, a total of 180 participants aged ≥18 years were randomly assigned to receive placebo or 1 dose of 3 different dose levels of mRNA-1010 (n=21-24 participants per group). In Part 2 of the trial, a total of 501 participants aged ≥18 years were randomly assigned to receive a single dose of Afluria or mRNA-1010 (n=13-21 for Afluria; n=31-60 for mRNA-1010 groups). Qualified assays were used in this study and multiple participants were assessed in each assay, which makes these findings likely reproducible.
Randomization	The initial stage of Part 1 planned for approximately 36 participants (9 participants in each group) to be randomly assigned (1:1:1:1:) to receive a single dose of mRNA-1010 (50 µg, 100 µg, or 200 µg) or placebo. Safety data up to 7 days after vaccination were reviewed by a blinded internal safety team; after confirmation that no study pause rules were met, the remaining participants (approximately 36 in each group for a total of 144 participants) were randomly assigned in the Part 1 expansion stage. Randomization at this stage was stratified by age (18-49 years and ≥50 years) and was balanced within each group. In Part 2, approximately 500 participants were planned to be randomly assigned (3:3:3:1) to receive a single dose of mRNA-1010 (25 µg, 50 µg, or 100 µg) or a licensed quadrivalent seasonal influenza vaccine (Afluria®; Seqirus Pty Ltd, Parkville, Victoria, Australia). Randomization was performed in parallel among the 4 vaccine groups, and participants were stratified by age (18-49 years, 50-64 years, or ≥65 years) and vaccination status in the previous influenza season (received or not received).
Blinding	This was an observer-blind study, with the Investigator, study staff, study participants, site monitors, and Sponsor personnel blinded to the vaccine administered until the study database was locked and unblinded. A limited number of unblinded personnel were assigned to vaccine accountability procedures and prepared the study vaccines, while an unblinded medically qualified study site personnel administered vaccination. Unblinded site monitors were assigned responsibilities to ensure sites were following all proper vaccination accountability, preparation, and administration procedures. The planned interim analysis was performed by an independent, unblinded statistical and programming team and did not communicate results to blinded Investigators, study site staff, clinical monitors, or participants. The Data Safety Monitoring Board reviewed unblinded interim analysis data provided by the independent unblinded statistician to safeguard the interests of the study participants.

Reporting for specific materials, systems and methods

We require information from authors about some types of materials, experimental systems and methods used in many studies. Here, indicate whether each material, system or method listed is relevant to your study. If you are not sure if a list item applies to your research, read the appropriate section before selecting a response.

Materials & experimental systems

n/a	Involved in the study	
☒	☐	Antibodies
☒	☐	Eukaryotic cell lines
☒	☐	Palaeontology and archaeology
☒	☐	Animals and other organisms
☐	☒	Clinical data
☒	☐	Dual use research of concern

Methods

n/a	Involved in the study	
☒	☐	ChIP-seq
☒	☐	Flow cytometry
☒	☐	MRI-based neuroimaging

Clinical data

Policy information about clinical studies

All manuscripts should comply with the ICMJE guidelines for publication of clinical research and a completed CONSORT checklist must be included with all submissions.

Clinical trial registration	NCT04956575
Study protocol	Study protocol has been provided as a supplementary file.
Data collection	This is a first-in-human, phase 1/2, randomized, observer-blinded study at 20 sites in the United States to evaluate the safety, reactogenicity, and immunogenicity of mRNA-1010 in adults ≥18 years of age. Part 1 enrollment occurred between July 6, 2021, and August 18, 2021; Part 2 enrollment occurred between November 10, 2021, and November 15, 2021. Exact study sites are provided online at https://clinicaltrials.gov/ct2/show/NCT04956575
Outcomes	The predefined primary objectives of Part 1 were to evaluate the safety and reactogenicity of a single dose of mRNA-1010 (50 µg, 100 µg, and 200 µg) versus placebo and to evaluate the humoral immunogenicity of a single dose of mRNA-1010 against vaccine-matched influenza A and B strains at Day 29. The predefined objectives of Part 2 were to evaluate the safety and reactogenicity of mRNA-1010 (25 µg, 50 µg, and 100 µg; primary) and to evaluate the humoral immunogenicity of mRNA-1010 and active comparator against vaccine-matched influenza A and B strains at Day 29 (primary and secondary).<br><br>Safety endpoints included solicited local and systemic adverse reactions (ARs) for 7 days after vaccination, safety laboratory abnormalities (Part 1 only), unsolicited adverse events (AEs) for 28 days after vaccination, as well as serious AEs (SAEs), AEs of special interest (AESIs), and medically attended AEs (MAAEs) through the end of the study (Day 181). Interim safety data through Day 29 are included in this report. Participants used an electronic diary to record local ARs (ie, injection site pain, injection site redness, injection site hardness, or axillary swelling/tenderness ipsilateral to the side of injection), or systemic ARs (ie, headache, fatigue, myalgia, arthralgia, nausea/vomiting, chills, and fever). Safety laboratory assessments in Part 1 (at baseline and Day 8) included white blood cell count, hemoglobin, platelets, alanine aminotransferase, aspartate aminotransferase, total bilirubin, alkaline phosphatase, and creatinine. Solicited ARs were assessed in all participants in the safety population who contributed any solicited AR data (solicited safety population). The number of events of unsolicited AEs, SAEs, AESIs, and MAAEs were summarized, while descriptive summary statistics were provided for all other safety analyses.<br><br>Blood samples for immunogenicity assessments were collected on Days 1 (baseline), 8 (Part 1 only), 29, and 181 (end of study). This report summarizes immunogenicity assessments at baseline and Day 29. Immunogenicity endpoints included geometric mean titers (GMTs) at Day 1 and 29, geometric mean fold rises (GMFRs) at Day 29 over Day 1 (baseline), and percentage of participants with seroconversion at Day 29 of serum anti-HA antibodies against vaccine-matched influenza A and B strains as measured by hemagglutination inhibition (HAI) assay using red blood cells from guinea pig and cell-grown viruses. The geometric mean of specific antibody titers with corresponding 95% confidence intervals (CIs) at Day 29 and GMFRs of specific antibody titers with corresponding 95% CI at Day 29 over Day 1 (baseline) were calculated for each vaccination group; 95% CIs were calculated based on the t distribution of the log 2-transformed values and then back transformed to the original scale. The seroconversion rate from baseline was determined along with 2-sided 95% CIs using the Clopper-Pearson method. The rate of seroconversion was defined as the percentage of participants with either a prevaccination HAI titer <1:10 and a postvaccination HAI titer ≥1:40 or a prevaccination HAI titer ≥1:10 and a minimum 4-fold rise in postvaccination HAI antibody titer. In Part 2, GMTs and seroconversion rates at Day 29 were compared in the mRNA-1010 groups with the active comparator group.
